# Adiposity, metabolomic biomarkers, and risk of nonalcoholic fatty liver disease: a case-cohort study

**DOI:** 10.1093/ajcn/nqab392

**Published:** 2021-12-13

**Authors:** Yuanjie Pang, Christiana Kartsonaki, Jun Lv, Iona Y Millwood, Zammy Fairhurst-Hunter, Iain Turnbull, Fiona Bragg, Michael R Hill, Canqing Yu, Yu Guo, Yiping Chen, Ling Yang, Robert Clarke, Robin G Walters, Ming Wu, Junshi Chen, Liming Li, Zhengming Chen, Michael V Holmes

**Affiliations:** Department of Epidemiology and Biostatistics, School of Public Health, Peking University, Beijing, China; Clinical Trial Service Unit and Epidemiological Studies Unit (CTSU), Nuffield Department of Population Health, University of Oxford, Oxford, United Kingdom; Medical Research Council Population Health Research Unit (MRC PHRU) at the University of Oxford, Nuffield Department of Population Health, University of Oxford, Oxford, United Kingdom; Department of Epidemiology and Biostatistics, School of Public Health, Peking University, Beijing, China; Peking University Center for Public Health and Epidemic Preparedness and Response (PKU-PHEPR), Peking University, Beijing, China; Clinical Trial Service Unit and Epidemiological Studies Unit (CTSU), Nuffield Department of Population Health, University of Oxford, Oxford, United Kingdom; Medical Research Council Population Health Research Unit (MRC PHRU) at the University of Oxford, Nuffield Department of Population Health, University of Oxford, Oxford, United Kingdom; Clinical Trial Service Unit and Epidemiological Studies Unit (CTSU), Nuffield Department of Population Health, University of Oxford, Oxford, United Kingdom; Clinical Trial Service Unit and Epidemiological Studies Unit (CTSU), Nuffield Department of Population Health, University of Oxford, Oxford, United Kingdom; Clinical Trial Service Unit and Epidemiological Studies Unit (CTSU), Nuffield Department of Population Health, University of Oxford, Oxford, United Kingdom; Medical Research Council Population Health Research Unit (MRC PHRU) at the University of Oxford, Nuffield Department of Population Health, University of Oxford, Oxford, United Kingdom; Clinical Trial Service Unit and Epidemiological Studies Unit (CTSU), Nuffield Department of Population Health, University of Oxford, Oxford, United Kingdom; Department of Epidemiology and Biostatistics, School of Public Health, Peking University, Beijing, China; Peking University Center for Public Health and Epidemic Preparedness and Response (PKU-PHEPR), Peking University, Beijing, China; Chinese Academy of Medical Sciences, Beijing, China; Clinical Trial Service Unit and Epidemiological Studies Unit (CTSU), Nuffield Department of Population Health, University of Oxford, Oxford, United Kingdom; Medical Research Council Population Health Research Unit (MRC PHRU) at the University of Oxford, Nuffield Department of Population Health, University of Oxford, Oxford, United Kingdom; Clinical Trial Service Unit and Epidemiological Studies Unit (CTSU), Nuffield Department of Population Health, University of Oxford, Oxford, United Kingdom; Medical Research Council Population Health Research Unit (MRC PHRU) at the University of Oxford, Nuffield Department of Population Health, University of Oxford, Oxford, United Kingdom; Clinical Trial Service Unit and Epidemiological Studies Unit (CTSU), Nuffield Department of Population Health, University of Oxford, Oxford, United Kingdom; Clinical Trial Service Unit and Epidemiological Studies Unit (CTSU), Nuffield Department of Population Health, University of Oxford, Oxford, United Kingdom; Medical Research Council Population Health Research Unit (MRC PHRU) at the University of Oxford, Nuffield Department of Population Health, University of Oxford, Oxford, United Kingdom; Jiangsu Center for Disease Control and Prevention, Nanjing, China; National Center for Food Safety Risk Assessment, Beijing, China; Department of Epidemiology and Biostatistics, School of Public Health, Peking University, Beijing, China; Peking University Center for Public Health and Epidemic Preparedness and Response (PKU-PHEPR), Peking University, Beijing, China; Clinical Trial Service Unit and Epidemiological Studies Unit (CTSU), Nuffield Department of Population Health, University of Oxford, Oxford, United Kingdom; Medical Research Council Population Health Research Unit (MRC PHRU) at the University of Oxford, Nuffield Department of Population Health, University of Oxford, Oxford, United Kingdom; Clinical Trial Service Unit and Epidemiological Studies Unit (CTSU), Nuffield Department of Population Health, University of Oxford, Oxford, United Kingdom; Medical Research Council Population Health Research Unit (MRC PHRU) at the University of Oxford, Nuffield Department of Population Health, University of Oxford, Oxford, United Kingdom; National Institute for Health Research Oxford Biomedical Research Centre, Oxford University Hospital, Oxford, United Kingdom

**Keywords:** adiposity, nonalcoholic fatty liver disease, metabolomics, Mendelian randomization, Chinese

## Abstract

**Background:**

Globally, the burden of obesity and associated nonalcoholic fatty liver disease (NAFLD) are rising, but little is known about the role that circulating metabolomic biomarkers play in mediating their association.

**Objectives:**

We aimed to examine the observational and genetic associations of adiposity with metabolomic biomarkers and the observational associations of metabolomic biomarkers with incident NAFLD.

**Methods:**

A case-subcohort study within the prospective China Kadoorie Biobank included 176 NAFLD cases and 180 subcohort individuals and measured 1208 metabolites in stored baseline plasma using a Metabolon assay. In the subcohort the observational and genetic associations of BMI with biomarkers were assessed using linear regression, with adjustment for multiple testing. Cox regression was used to estimate adjusted HRs for NAFLD associated with biomarkers.

**Results:**

In observational analyses, BMI (kg/m^2^; mean: 23.9 in the subcohort) was associated with 199 metabolites at a 5% false discovery rate. The effects of genetically elevated BMI with specific metabolites were directionally consistent with the observational associations. Overall, 35 metabolites were associated with NAFLD risk, of which 15 were also associated with BMI, including glutamate (HR per 1-SD higher metabolite: 1.95; 95% CI: 1.48, 2.56), cysteine-glutathione disulfide (0.44; 0.31, 0.62), diaclyglycerol (C32:1) (1.71; 1.24, 2.35), behenoyl dihydrosphingomyelin (C40:0) (1.92; 1.42, 2.59), butyrylcarnitine (C4) (1.91; 1.38, 2.35), 2-hydroxybehenate (1.81; 1.34, 2.45), and 4-cholesten-3-one (1.79; 1.27, 2.54). The discriminatory performance of known risk factors was increased when 28 metabolites were also considered simultaneously in the model (weighted C-statistic: 0.84 to 0.90; *P*  < 0.001).

**Conclusions:**

Among relatively lean Chinese adults, a range of metabolomic biomarkers are associated with NAFLD risk and these biomarkers may lie on the pathway between adiposity and NAFLD.

See corresponding editorial on page 603.

## Introduction

Globally, obesity affects more than 670 million adults, with rapidly rising prevalence and associated disease burden in many low- and middle-income countries, including China (1). In China, approximately 30% of the adult population have nonalcoholic fatty liver disease (NAFLD) diagnosed by ultrasound or computed tomography (CT) (2), despite having relatively low BMI (3). Individuals with NAFLD have higher risks of cirrhosis, liver cancer, cardiometabolic disease, and all-cause mortality (2, 4, 5). Of all established risk factors, adiposity is the strongest determinant of NAFLD risk both in Western countries and in China (2, 4, 5). Several mechanisms have been proposed to explain the strong association between adiposity and NAFLD, including chronic inflammation, oxidative stress, and insulin resistance (2, 5, 6). However, there is still limited understanding of the pathophysiology of NAFLD, as well as of the metabolic derangements associated with adiposity and their role in the development of NAFLD. Metabolomics quantifies a broad range of small-molecule metabolites at the cellular level and has emerged as a powerful technique that might be of utility in shedding light on biomarkers associated with NAFLD (7).

Over 20 studies have assessed the associations of metabolomics with NAFLD or liver fat content (7). These studies have identified several pathways underlying the development of NAFLD, including glutamate, branched-chain amino acids (BCAAs), glycolysis, sex hormones, fatty acids, and very low density lipoproteins (8–17). However, the majority of these studies were cross-sectional, involved a small number of cases (typically <50), and measured a limited set of metabolomic biomarkers (typically <100). More importantly, no studies have assessed simultaneously associations of adiposity with metabolomics and the role of metabolomics in potentially mediating the association between adiposity and NAFLD. Reliable assessment of any apparent mediating effects of metabolomic biomarkers should improve the understanding of pathways linking adiposity and metabolic liver diseases and could inform the development of potential therapeutic opportunities.

The causal effects of adiposity on metabolites associated with NAFLD have yet to be fully characterized but can be established by Mendelian randomization (MR) (18, 19). MR utilizes the random assortment of genes from parents to offspring at conception, and uses gene variants associated with the exposure of interest as unconfounded markers, thus providing an approach for assessing causation (18, 19). Assessing the genetic associations of adiposity with metabolomics can inform the selection of biomarkers associated with NAFLD and circumvent reverse causality, where the presence of NAFLD could cause changes in the metabolite profile.

Using a case-subcohort study embedded within the prospective China Kadoorie Biobank (CKB), this study aims to examine *1*) the observational and genetic associations of adiposity with blood-based metabolites, *2*) the observational associations of circulating metabolites with incident NAFLD risk, and *3*) the role of metabolomics in predicting incident NAFLD risk.

## Methods

### Study population and design

The CKB is a prospective cohort study of 512,891 adults aged 30–79 y, recruited in 2004–2008 from 10 (5 urban and 5 rural) geographically defined areas in China. Details of the CKB design, survey methods, and long-term follow-up have been previously described (20). At the baseline survey, and subsequent periodic resurveys in a random subset of participants, participants completed an interviewer-administered laptop-based questionnaire on sociodemographic characteristics, smoking, alcohol consumption, diet, physical activity, and medical history, and underwent a range of physical measurements, including height, weight, hip and waist circumference, bioimpedance, lung function, blood pressure, and heart rate. All participants provided a 10-mL nonfasting (with time since last meal recorded) blood sample for immediate on-site testing of random plasma glucose (RPG) and long-term storage. The study was approved by the ethics committee and research council of the Chinese Centre for Disease Control and Prevention and the Oxford Tropical Research Ethics Committee at the University of Oxford. All participants provided written informed consent.

For the present case-subcohort study, a random sample of 192 NAFLD cases [International Classification of Diseases, 10th Revision (ICD-10), K76.0] out of a total of 961 cases accumulated until 1 January 2016 were included, as well as a subcohort of 192 participants who were randomly sampled from the baseline cohort with genotyping data available as part of a random sample with genotyping. All NAFLD cases were ascertained from medical records and 93% of cases had ultrasound or CT (21).

### Assessment of adiposity

All anthropometric measurements were taken by trained technicians while participants were wearing light clothes and no shoes, usually to the nearest 0.1 cm or 0.1 kg. Standing height was measured using a stadiometer. Weight was measured using a body-composition analyzer (TANITA-TBF-300GS; Tanita Corporation), with subtraction of weight of clothing according to season (ranging from 0.5 kg in summer to 2.0–2.5 kg in winter). Waist circumference (WC) and hip circumference (HC) were measured using a soft nonstretchable tape, with HC measured at the maximum circumference around the buttocks. BMI was calculated as the measured weight in kilograms divided by the square of the measured height in meters.

### Metabolomics assay

The Metabolon platform was used to quantify levels of 1208 metabolites in baseline blood samples (**Supplemental Table 1**). The metabolites were grouped into 8 chemical classes (amino acids, carbohydrates, cofactors and vitamins, energy metabolites, lipids, nucleotide metabolites, peptides, and xenobiotics), which were further classified into 9 super-pathways and 105 sub-pathways (Supplemental Table 1). The Metabolon Discovery HD4 platform used a single nontargeted extraction with methanol and recovered a diverse set of metabolomic biomarkers by protein precipitation (22). Samples were then analyzed using ultra HPLC and GC coupled with tandem MS and MS. The mass spectra peaks were compared with a chemical reference library generated from 2500 standards to identify individual metabolomic biomarkers. In-house peak detection and integration software was used whose data output was a list of *m*/*z* ratios, retention indices, and AUC values. Metabolite peak intensities were run-day normalized. Values below the limit of detection were assigned the minimum observed value for that metabolite in the dataset. There were 1208 metabolites of known and unknown identity measured at detectable levels (Supplemental Table 1). Metabolites were excluded if missing in >95% of participants, leaving 1153 metabolites for the present analysis (Supplemental Table 1).

### Genotyping

**FIGURE 1:**
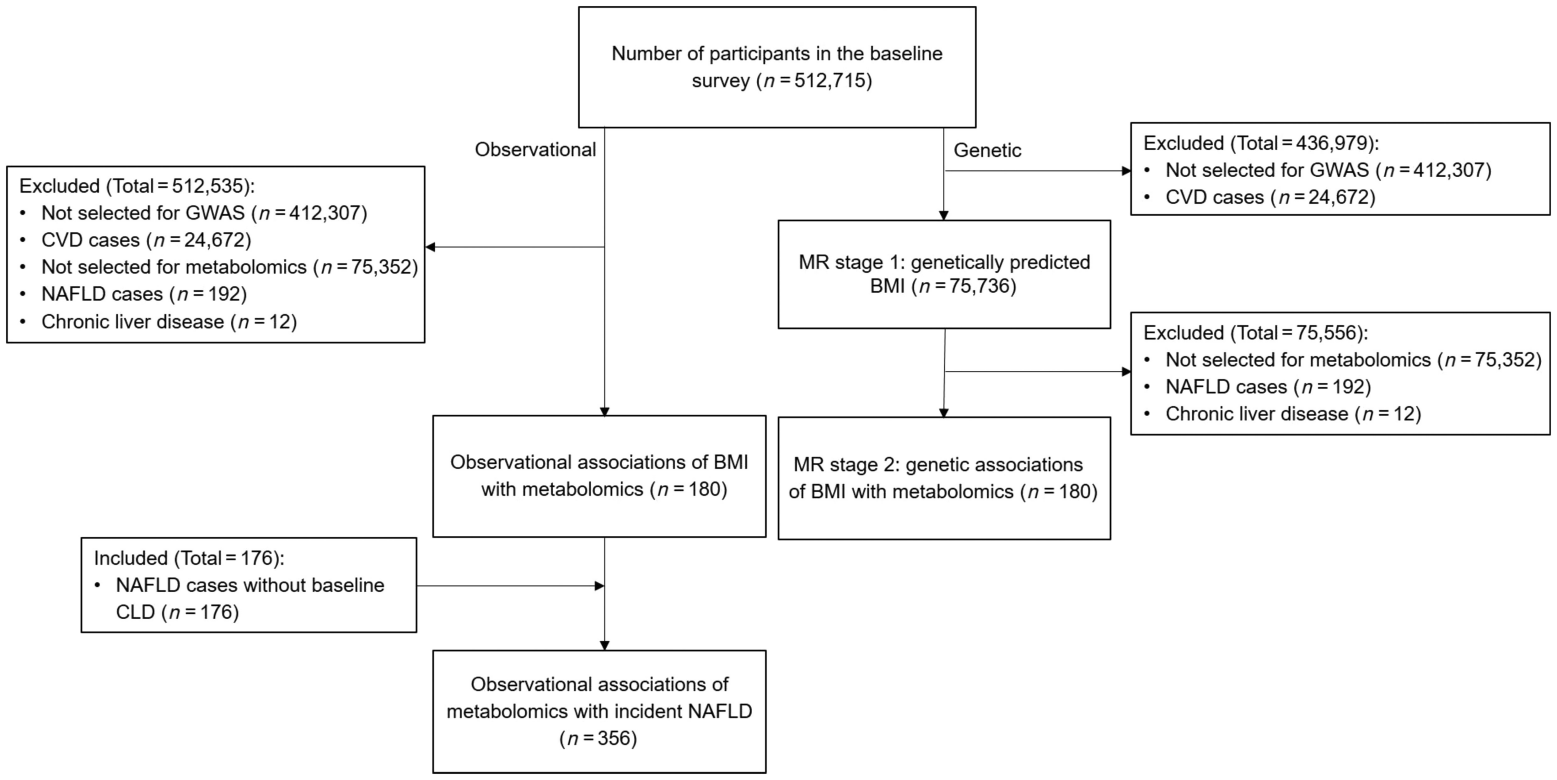
Flow diagram of the study design. CLD, chronic liver disease; CVD, cardiovascular disease; GWAS, genome-wide association study; MR, Mendelian randomization; NAFLD, nonalcoholic fatty liver disease.

Genotyping was conducted using a custom-designed 800K-SNP (single nucleotide polymorphism) array (Axiom; Affymetrix) with imputation to 1000 Genomes Phase 3. In CKB release 15, genotyping data were available for 100,408 participants with quality control (overall call rate >99.97% across all variants), including 75,736 randomly selected participants from the CKB cohort and 24,672 selected for various nested case-control studies of incident cardiovascular disease (CVD) or chronic obstructive pulmonary disease. To avoid potential selection bias, the present study only used genotyping data from a population-based random sample (**Figure 1**).

### Genetic risk score for BMI

We selected 670 independent SNPs (*r*^2^ ≤0.01 in Europeans) as instrumental variables for BMI based on a meta-analysis of UK Biobank and the Genetic Investigation of Anthropometric Traits (GIANT) consortium (23). After excluding 84 SNPs with low minor allele frequency (MAF <1%) in CKB, 586 SNPs remained for the BMI genetic score (Figure 1 and **Supplemental Table 2**). We constructed an externally weighted BMI genetic score by summing the number of effect alleles carried by each participant (SD difference in BMI per effect allele), weighted by the reported effect size of each variant on BMI reported in Biobank Japan (BBJ) (24). After excluding 15 SNPs with low MAF (<1%) in BBJ, 571 SNPs remained for the weighted score. Five of the 571 SNPs were unavailable in BBJ and proxy SNPs were selected [*R*^2^ ≥0.8 using the linkage disequilibrium structure in CEPH (Centre d'Etude du Polymorphisme Humain) from Utah (CEU) (1000 Genomes Project)].

### Long-term follow-up and ascertainment of NAFLD

The vital status of each participant was determined periodically through China CDC's Disease Surveillance Points system, supplemented by regular checks against local residential records and health insurance records and by annual active confirmation through street committees or village administrators (25). Additional information about major diseases and any episodes of hospitalization was collected through linkages, via each participant's unique national identification number, with disease registries [for cancer, coronary heart disease (CHD), stroke, and diabetes] and national health insurance claims databases (for any disease), which has almost universal coverage in the study areas. ICD-10 code (K76.0) and Chinese keywords were used to identify and allow standardization of NAFLD cases in the health insurance database. NAFLD was diagnosed according to the Chinese guidelines and was defined as the presence of hepatic steatosis on ultrasound without significant alcohol consumption (>40 g/d or 280 g/wk for women; 60 g/d or 420 g/wk for men) and/or viral hepatitis (hepatitis B virus or hepatitis C virus) (26). We additionally excluded participants who reported excessive alcohol consumption (>40 g/d for women and 60 g/d for men) and tested positive for hepatitis B surface antigen (HBsAg) at study baseline. All events were coded using the ICD-10 by trained staff who were blinded to baseline information.

### Statistical analysis

We excluded individuals with a prior history of cancer, cirrhosis, or hepatitis at baseline, leaving 176 incident NAFLD cases and 180 subcohort participants for the main analysis (Figure 1). The primary outcome was NAFLD, and the secondary outcomes were metabolomic biomarkers. All metabolomic biomarkers were log-transformed and then standardized to have an SD of 1. Mean values and prevalence of baseline characteristics were calculated for the subcohort and NAFLD cases, standardized to age (in 5-year groups), sex, and area structure (10 regions) of the CKB population.

#### Observational analysis

The observational associations of adiposity with metabolites were examined in the subcohort using linear regression, adjusting for age at baseline (continuous), sex, region (10 regions), education (4 groups: no formal school, primary school, middle/high school, or college/university), smoking (3 groups: never regular, former regular, or current regular), and fasting time (continuous). For WC, we additionally adjusted for BMI in a separate analysis to assess whether central adiposity was associated with metabolites beyond BMI. For each biomarker, the adjusted SD differences and 95% CIs associated with 1-SD higher adiposity were estimated. The observational associations of metabolites with NAFLD risk were examined in all 356 participants using Cox regression fitted using the Prentice pseudo-partial likelihood (27), using time in study as the time scale and adjusting for the same covariates as in the adiposity-metabolomics analysis. For both analyses, we calculated false discovery rate (FDR)–corrected *P* values using the Benjamini-Hochberg method (28).

#### MR analysis

In MR analysis, the potential causal effects of BMI on metabolites were assessed by the 2-stage least-squares method using individual participant–level data (IPD). In the first stage, the associations between the externally weighted BMI genetic score and BMI were examined in 75,736 participants in the genome-wide association study (GWAS) population subset using linear regression, adjusting for age, age squared, sex, area, the first 12 principal components, education, smoking, and alcohol. To account for population structure, covariates for genetic analyses included 12 principal components derived using 72,473 unrelated participants and a linkage disequilibrium (LD)-pruned set of 140,830 variants. In the second stage, the associations of the resulting predicted BMI values with metabolomics were examined in the subcohort of 180 individuals using linear regression with the same adjustments. We calculated effect estimates per 3.4 kg/m^2^ higher genetically elevated BMI (corresponding to 1-SD baseline BMI in CKB) on metabolomic biomarkers to allow comparison with the estimates for observational BMI. Unadjusted *P* values were reported for the genetic associations of BMI with metabolomic biomarkers to avoid over-correction. The genetic associations were compared with the corresponding observational associations using Cochran's *Q* test.

#### Multivariable models

We fitted multivariable models with metabolomic biomarkers using the method developed by Cox and Battey (29), without transformations or interactions of variables. The 1153 markers were laid on a 11 × 11 × 10 cuboid, with the remaining 21 positions filled with normally distributed random variables (mean zero and variance 1). A regression was fitted with each set of explanatory variables indexed by each dimension of the cuboid, adjusting for age at baseline, sex, region, education, smoking, and fasting time. The metabolomic biomarkers showing the strongest associations with NAFLD risk were kept from each regression, defined as *1*) those with *z* >2.5, *2*) the 2 most significant, or *3*) the 3 most significant. Then, for each of the 3 criteria, biomarkers identified as such 3 times were selected and included in a model, adjusting for the covariates above. After the first round of selection, 122 metabolomic biomarkers were selected by at least 1 of the 3 criteria. We repeated the selection procedure with these 122 metabolomic biomarkers, retaining 15, 17, and 27 metabolomic biomarkers for each of the 3 criteria.

We then examined whether including selected metabolomic biomarkers adds to the discriminatory ability of a model with established risk factors by fitting 4 models: *1*) model 1 included age, age squared, sex, region, education, household income, smoking, total physical activity, BMI, and diabetes, which have been shown to be associated with NAFLD in Chinese (21); *2*) model 2 additionally included the metabolomic biomarkers identified from the multivariable models as being most associated with risk of NAFLD—that is, variables selected 3 times from the Cox–Battey procedure when keeping those with *z* >2.5 (i.e., model 1 plus 15 metabolic biomarkers by criterion 1); *3*) model 3 additionally included metabolomic biomarkers selected when keeping the 2 most significant (i.e., model 1 plus 21 metabolomic biomarkers by criteria 1 and 2); and *4*) model 4 additionally including those selected when keeping the 3 most significant (i.e., model 1 plus 28 metabolomic biomarkers by criteria 1–3), such that models were nested. Discrimination of risk prediction models was assessed using a weighted C-index (30, 31).

#### Sensitivity analyses

In sensitivity analyses, the observational associations of BMI with metabolomic biomarkers were additionally adjusted for dietary factors, prevalent diabetes, systolic blood pressure (SBP), and alcohol. We also conducted MR-Egger and weighted median MR for the genetic associations of BMI with metabolomic biomarkers, using 2-sample MR analysis, with the same adjustments as the IPD analysis. Statistical analyses were done using R version 4.0.2 and package “MendelianRandomization” (R Foundation for Statistical Computing).

## Results

Compared with the subcohort, NAFLD cases were older, more likely to be female and from rural areas, and had higher educational levels (**Table 1**). NAFLD cases and subcohort participants had similar levels of total physical activity and sedentary leisure time, but NAFLD cases were more likely to smoke and drink alcohol. High SBP, RPG, and adiposity, as well as hypertension, diabetes, and CHD, were more prevalent among cases than subcohort members (Table 1).

**TABLE 1:** Baseline characteristics of subcohort participants and NAFLD cases^1^

	Subcohort	NAFLD cases
Variable	(*n* = 180)	(*n* = 176)
Age (±SD), y	50.6 ± 10.1	51.1 ± 9.1
Female, %	62.8	64.5
Socioeconomic and lifestyle factors, %		
Urban residency	48.6	25.1
≥9 years of education	21.6	28.0
Household income ≥35 000 RMB/y	19.5	21.4
Ever regular smoking, %		
Male	65.1	69.1
Female	5.9	9.2
Weekly alcohol drinking, %		
Male	31.6	45.2
Female	4.5	4.6
Total physical activity (±SD), MET-h/d	21.8 ± 14.6	21.2 ± 13.4
Sedentary leisure time (±SD), h/d	3.1 ± 1.5	3.0 ± 1.9
Blood pressure and anthropometry		
SBP (±SD), mmHg	131.2 ± 19.9	133.0 ± 19.7
RPG (±SD), mmol/L	5.9 ± 2.7	6.4 ± 2.5
BMI (±SD), kg/m^2^	23.9 ± 3.5	26.1 ± 3.5
Waist circumference (±SD), cm	79.6 ± 10.0	85.7 ± 9.9
Hip circumference (±SD), cm	90.5 ± 7.3	94.0 ± 7.2
Waist-to-hip ratio (±SD)	0.88 ± 0.07	0.91 ± 0.08
Percentage body fat (±SD), %	29.0 ± 8.2	33.4 ± 9.2
Standing height (±SD), cm	158.2 ± 7.9	158.7 ± 9.1
Prior disease history, %		
Diabetes	5.8	6.5
Coronary heart disease	3.1	5.9
Stroke or TIA	2.9	0
Hypertension	10.9	13.7
Family history of diabetes	5.3	5.3
Family history of cancer	18.5	18.6

1 Results by BMI categories are standardized by age, sex, and region, whereas for age they were adjusted for sex and region. Values are means unless otherwise stated. MET, metabolic equivalent of task; NAFLD, nonalcoholic fatty liver disease; RMB, Renminbi; RPG, random plasma glucose; SBP, systolic blood pressure; TIA, transient ischemic attack.

For the 1153 metabolomic markers, the majority were approximately normally distributed, while the distributions of several metabolites were right skewed (phenyllactate, hydantoin-5-propionate) and left skewed (glutamine, tryptophan) (**Supplemental Figure 1**). There were low to moderate correlations between metabolomic biomarkers [median (IQR) of pairwise Pearson correlation coefficients: −0.036 (−0.19, 0.084)].

### MR assumptions

The first MR assumption is that the BMI score is associated with BMI, and this is supported by the data (F-statistic 1071, variance explained 1.1%). The second MR assumption is that the BMI score is associated with metabolomics only through BMI. We showed that MR-Egger estimates were consistent with the inverse variance weighted (IVW) estimates (Pearson correlation coefficient *r* = 0.95; **Supplemental Figure 2**). The third MR assumption is that the BMI score is not associated with traits that can confound the associations between BMI and metabolomics. The BMI genetic score was not associated with potential confounders (**Supplemental Table 3**).

### Observational associations of adiposity, metabolomic biomarkers, and NAFLD

**FIGURE 2:**
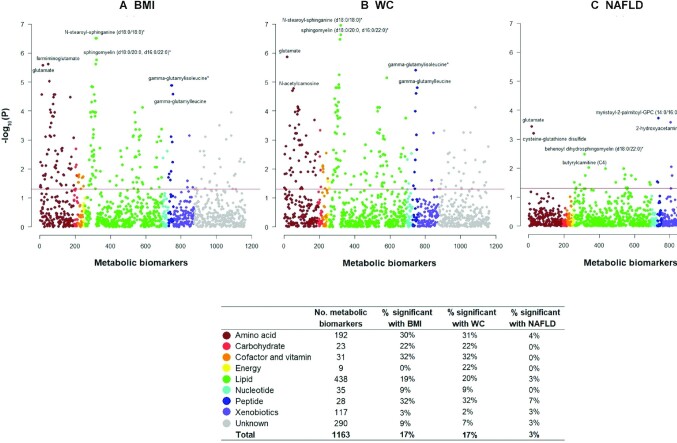
Manhattan plot showing the *P* values for observational associations of adiposity, metabolomic biomarkers, and risk of NAFLD. Observational associations of BMI and WC with all metabolomic biomarkers (A, B) and of metabolomic biomarkers with NAFLD risk (C) are shown. Metabolomic biomarkers are the dependent variables in A and B, whereas NAFLD is the dependent variable in C. The *x* axis shows the numeric order of metabolomic biomarkers shown in “Compound ID” Supplemental Table 1 within each super-pathway. The *y* axis shows log-transformed FDR-corrected *P* values. The horizontal line denotes −log(0.05). The table shows the percentage of metabolomic biomarkers that passed the FDR threshold of 0.05 by super-pathways. The estimates were adjusted for age, age squared, sex, area, smoking, education, and fasting time. FDR, false discovery rate; NAFLD, nonalcoholic fatty liver disease; WC, waist circumference. An asterisk denotes that the metabolic biomarker is a mixture of isoforms (with the same number of carbons and carbon-carbon double bonds).

Overall, 199 of the 1153 metabolomic biomarkers were associated with BMI after FDR correction, including predominantly lipids (*n* = 84) and amino acids (*n* = 58) (**Figure 2**). The associations were positive for 172 metabolites and inverse for 27 metabolites, with mean SD differences of metabolites ranging from −0.82 to 0.50 per 1-SD higher BMI (**Supplemental Table 4**). For central adiposity, 201 of the 1153 metabolomic biomarkers were associated (at FDR <5%) with WC (**Supplemental Figure 3**), with a Pearson correlation coefficient of 0.96 between the 2 sets of estimates (BMI-metabolomic biomarkers and WC-metabolomic biomarkers). When additionally adjusting for BMI, the associations of WC with metabolomic biomarkers tended to attenuate towards the null and became nonsignificant (FDR-corrected *P* values, Supplemental Table 4 and Supplemental Figure 3).

**FIGURE 3:**
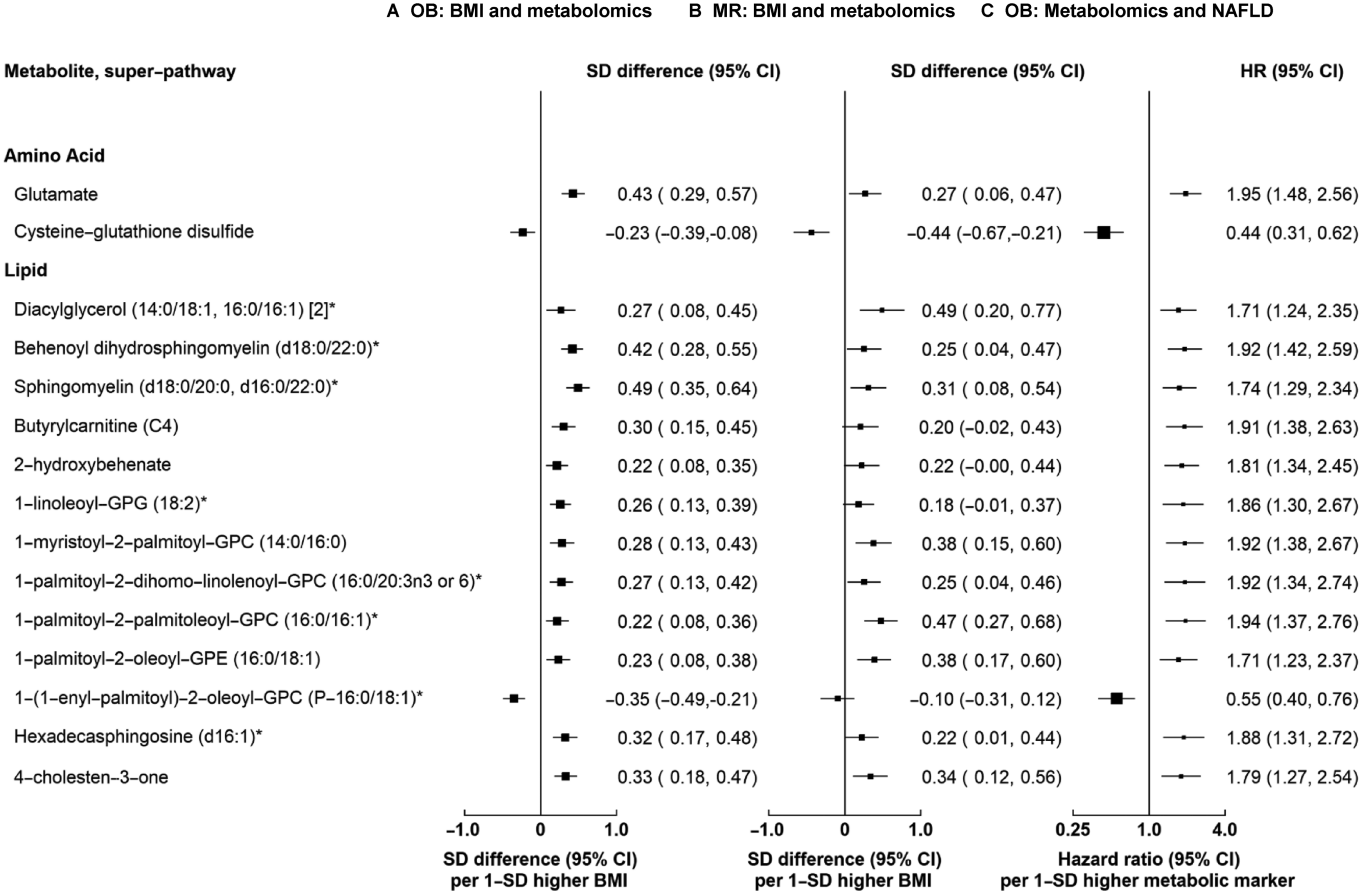
Associations of BMI, metabolomic biomarkers, and risk of NAFLD for 15 metabolomic biomarkers associated with both BMI and NAFLD at 5% FDR. Column A shows adjusted SD differences (95% CI) of metabolomic biomarkers per 1-SD higher observational BMI for 15 metabolomic biomarkers associated with both BMI and NAFLD with FDR-corrected *P* values <0.05. Column B shows corresponding estimates per 1-SD higher genetically elevated BMI. The observational estimates were adjusted for age, age squared, sex, area, smoking, education, and fasting time. The MR estimates were adjusted for age, age squared, sex, area, the first 12 principal components, education, and smoking. The SD for BMI in the whole CKB cohort was 3.4 kg/m^2^. Column C shows adjusted HR (95% CI) of NAFLD per 1-SD higher metabolomic biomarkers. An asterisk denotes that the metabolic biomarker is a mixture of isoforms (with the same number of carbons and carbon-carbon double bonds). FDR, false discovery rate; GPG, glycerophosphoglycerol; GPC, glycerophosphocholine; OB, observational; MR, Mendelian randomization; NAFLD, nonalcoholic fatty liver disease.

At an FDR <5%, 35 of the 1153 metabolomic biomarkers were associated with NAFLD risk, mostly lipids (*n* = 23) (**Supplemental Table 5**), with positive associations for 26 biomarkers and inverse associations for 12 biomarkers. Of these 35 biomarkers, 15 were also associated with BMI (FDR-corrected *P* < 0.05; **Figure 3**). There was a concordant pattern between the associations of BMI with metabolomic biomarkers and of metabolomic biomarkers with NAFLD risk (Figure 3).

### Genetic associations of adiposity with metabolomic biomarkers

For the 199 metabolomic biomarkers associated with both BMI and NAFLD risk, there was general concordance between the associations of observational BMI and of genetically elevated BMI with metabolomic biomarkers, with greater magnitudes of genetic than observational associations (Pearson correlation coefficient *r* = 0.58; **Supplemental Figure 4, Supplemental Table 6**). Cochran's *Q* test showed no evidence that the genetic estimates differed from the observational estimates, with the exception of 3 biomarkers. The genetic associations were stronger for 2 biomarkers (1,5-anhydroglucitol and N-acetylglycine), whereas the genetic association was opposite to the observational association for glucuronide of C10H18O2 (a partially characterized molecule). For the 15 metabolomic biomarkers associated with both BMI and NAFLD risk, the associations between genetically predicted BMI with metabolomic biomarkers were of a consistent direction in all cases and nominally significant for 11 biomarkers (Figure 3).

### Multivariable analysis

The metabolomic biomarkers associated with NAFLD identified using the Cox–Battey method largely overlapped with the biomarkers identified in the univariable analyses (**Supplemental Table 7**), with similar coefficients. The following metabolomic biomarkers were identified by all 3 criteria (*z* >2.5, the 2 most significant, and the 3 most significant): 1H-indole-7-acetic acid, 3-hydroxysebacate, 3b-hydroxy-5-cholenoic acid, ɑ-tocopherol, cysteine-glutathione disulfide, glutamate, indolin-2-one, saccharin, X-13729, X-17676, and X-21785. The addition of metabolomic biomarkers to a model with established risk factors yielded small increases in the discriminatory ability of the model (**Table 2**). The weighted C statistic increased from 0.84 (base model, 95% CI: 0.80, 0.88) to 0.88 (0.85, 0.92) when adding 15 biomarkers (criterion 1), to 0.89 (0.86, 0.93) when adding 21 biomarkers (criteria 1–2), and to 0.90 (0.87, 0.93) when adding 28 biomarkers (criteria 1–3).

**TABLE 2:** Exploratory investigation of metabolomic biomarkers as predictors of incident diagnosis of NAFLD^1^

Variables included in model	Weighted C statistic	95% CI	*P*
Base model	0.84	(0.80, 0.88)	
+ 15 metabolic biomarkers (criterion 1)	0.88	(0.85, 0.92)	0.005
+ 21 metabolic biomarkers (criteria 1, 2)	0.89	(0.86, 0.93)	0.002
+ 28 metabolic biomarkers (criteria 1–3)	0.90	(0.87, 0.93)	<0.001

^1^Base model includes age, age squared, sex, region, education, household income, smoking, total physical activity, BMI, and diabetes. Discrimination of models was assessed using a weighted C-index.

The criterion was each defined as *1*) those with *z* >2.5, *2*) the 2 most significant, or *3*) the 3 most significant. *P* values are for comparison with the base model. NAFLD, nonalcoholic fatty liver disease.

### Subgroup and sensitivity analyses

In the observational analyses, the associations of BMI with metabolomic biomarkers were similar for subgroups defined by age, sex, region, and smoking (**Supplemental Figure 5**). Similar associations were evident when further adjusting for dietary factors, SBP, and diabetes (**Supplemental Figure 6**). In the genetic analyses, MR-Egger and weighted median estimates were consistent with the IVW estimates (Pearson correlation coefficients *r* = 0.95 and 0.88; Supplemental Figure 2), whereas MR-Egger estimates were more imprecise (Supplemental Table 6).

## Discussion

**FIGURE 4:**
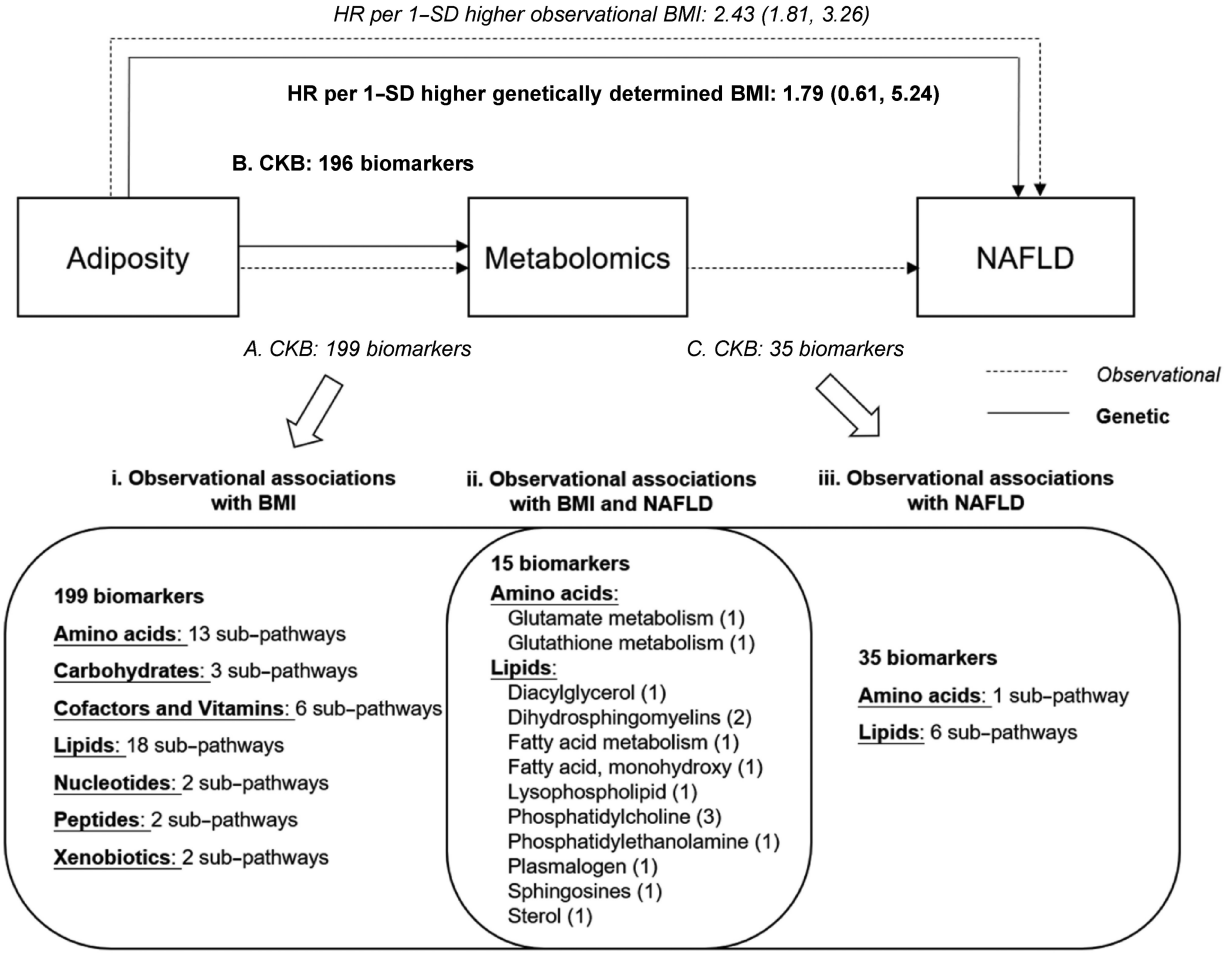
Central illustration of BMI, metabolomic biomarkers, and risk of NAFLD. We assessed the associations of BMI with ∼1200 metabolomic biomarkers and of these metabolomic biomarkers with risk of NAFLD. Previous reports in CKB showed observational and genetic associations between BMI and risk of NAFLD (21, 62). For the associations between BMI and metabolomic biomarkers, this study showed that measured BMI was observationally associated with 199 metabolomic biomarkers (amino acids, carbohydrates, cofactors and vitamins, lipids, nucleotides, peptides, and xenobiotics), with general concordance between the observational and genetic associations (except for 3 biomarkers—i.e., 1,5-anhydroglucitol, N-acetylglycine, and C10H18O2). This study also showed that 35 metabolomic biomarkers were associated with NAFLD risk. The lower panel illustrates the observational associations of BMI with metabolomics and of metabolomics with NAFLD by sub-pathways. There were 15 metabolomic biomarkers that were associated with BMI and NAFLD risk. CKB, China Kadoorie Biobank; HR, hazard ratio; NAFLD, nonalcoholic fatty liver disease.

In this relatively lean Chinese population, BMI was associated with a broad range of metabolomic biomarkers, which covered amino acids, lipids, peptides, carbohydrates, cofactors and vitamins, and xenobiotics. MR analyses demonstrated directionally concordant relations of observational and genetically elevated BMI with metabolomic biomarkers. Some of the BMI-associated metabolomic biomarkers (e.g., glutamate, butyrylcarnitine, 2-hydroxybehenate) were associated with NAFLD risk, suggesting they may act as potential mediators linking adiposity and NAFLD (**Figure 4**). The discriminatory ability of a model with known risk factors was increased when 28 metabolomic biomarkers identified by multivariable models were added. Although our study suggests that several metabolomic biomarkers might lie on the causal pathway between BMI and NAFLD, further investigations are warranted to identify whether these metabolomic biomarkers are causally related to NAFLD and to further evaluate their utility in risk prediction.

The associations of metabolomic biomarkers with NAFLD risk in the current study are generally consistent with previous cross-sectional or case-control studies on amino acids, lipids, and other metabolites (carnitines and urate) (**Supplemental Table 8** and **Supplemental Figures 7** and **8**). For amino acids, our results are consistent with previous studies, particularly for glutamate, cysteine-glutathione, γ-glutamyl dipeptide, and BCAAs (8, 9, 14, 15, 32, 33). For BCAAs, prospective cohort studies have shown positive associations with insulin resistance and subsequent type 2 diabetes (34, 35), whereas case-control studies and prospective cohort studies have shown consistently positive associations with NAFLD, including leucine, isoleucine, and valine (12–16). Although the associations of BCAAs with NAFLD were nominally significant in CKB, there was agreement of the associations of BMI with BCAAs and of BCAAs with NAFLD. Alterations in circulating concentrations of these amino acids and peptides may be due to the following: *1*) increased glutathione turnover as a result of oxidative stress (35, 36), *2*) increased conversion from intermediates to amino acids in the tricarboxylic acid (TCA) cycle (8), and *3*) increased transamination of amino acids being degraded in the liver and skeletal muscle (15).

For phosphatidylcholines di-acyl (PCs aa) and lyso-phosphatidylcholines (LPCs), cross-sectional studies showed positive associations between BMI and PC aa from C30 to C40, whereas the associations of BMI with PC aa >C40 and LPC varied by the degree of saturation and length of fatty acid chain (37, 38). Similarly, the associations of PC aa and LPC with hyperglycemia, type 2 diabetes, and NAFLD differed according to the degree of saturation and length of fatty acid chain (10, 17, 39, 40). In addition to PC and LPC, case-control studies have reported that several sphingomyelins (SMs) differed between NAFLD cases and healthy controls (10, 17). Consistent with previous studies, our study suggested that the associations of BMI, SM, and NAFLD risk differed by the length of fatty acid chain. Diacylglycerol (DAG) is derived from lipogenesis and membrane phospholipid (41). Membrane PC may contribute to the observed increase in DAG along with lipogenesis (41). A previous review paper has suggested that DAG might serve as the link between NAFLD and hepatic insulin resistance (42).

Recent studies have suggested sex differences in NAFLD (43, 44). A recent meta-analysis involving 62,239 participants identified women to have a 19% lower risk of NAFLD than men but had a higher risk of progression (45). The sex differences in the development and progression of NAFLD might be modified by estrogen (43, 44). In our study, we conducted sex-specific analyses and found overall consistent patterns for BMI and metabolomics as well as metabolomics and NAFLD in men and women (Supplemental Figure 5). Nonetheless, the lack of sex differences might be due to the small sample size. Future larger studies are warranted to assess the sex-specific associations of metabolomics with NAFLD. Apart from sex differences, NAFLD is a heterogenous disease (46, 47). Recent development of ’omics technology enables the characterization of disease sub-phenotypes, which can inform better understanding of disease natural history and response to therapy (48). These molecular phenotypes can improve our understanding of NAFLD heterogeneity, which serves as the basis of personalized medicine.

Our study showed that both general and central adiposity was associated with metabolomic biomarkers in Chinese. However, the associations between adiposity and metabolomics might differ in Europeans because of different distribution of adiposity in general populations (49). In addition, previous studies have shown that metabolically healthy obesity (MHO) and metabolically unhealthy obesity (MUO) both have higher risk of developing NAFLD and nonalcoholic steato hepatitis (NASH) compared with healthy individuals with normal weight, with stronger associations in MHO than MUO (50, 51).

Apart from showing consistency for the observational associations, we found that altered concentrations of several metabolomic biomarkers that BMI is genetically associated with were also observationally associated with NAFLD. The general concordance between the observational and genetic associations can improve understanding of the causes of NAFLD. Although previous studies suggested genetic associations of BCAAs with insulin resistance and diabetes (52, 53), there is limited evidence on the genetic associations between metabolomic biomarkers with NAFLD. A possible limitation may be the lack of available instruments for metabolomic biomarkers, particularly in East Asians (54).

Several prospective studies have constructed prediction models for imaging-diagnosed NAFLD using biomarker data and achieved good prediction ability (55–59). A European cohort study involving 1514 adults with MRI-derived liver fat content reported that ’omics data (metabolomics, proteomics, genetics, and transcriptomics) in combination with clinical variables had better prediction ability than clinical variables alone (AUC: 0.84 and 0.82) (55). Of note, this study identified PCs, glycerophospholipids, and valine among the highest-ranked metabolites, which were also associated with NAFLD risk in the current study. Previous prospective studies conducted in the Chinese population, involving 577 to 8226 participants and ascertaining NAFLD with ultrasound, showed that clinical variables (BMI, lipids, and liver enzymes) could be used to identify individuals at high risk of NAFLD, achieving an AUC between 0.72 and 0.93 (55–58). Consistent with previous studies, the current study constructed a model for incident diagnosis of NAFLD and found that combining metabolomic biomarkers with known risk factors (demographic and clinical variables) achieved good prediction performance (weighted C statistic: 0.90). Such a model may be applicable to the general population in identifying individuals at higher risk of NAFLD, who may benefit from additional investigations.

The strengths of the current study included measurement of a broad range of blood-based metabolomic biomarkers involved in multiple biological pathways, assessment of different adiposity measures, and use of MR to assess likely causal associations of adiposity, metabolites, and NAFLD risk in the same study population. Our study also had several limitations. First, our study relied on hospital records to capture NAFLD. However, we ascertained all NAFLD cases diagnosed between 2013 and 2015 and showed that 93% of all hospitalized NAFLD cases were diagnosed by ultrasound or CT. In addition, the metabolomic signatures of NAFLD observed in our study were consistent with previous studies of biopsy-ascertained NAFLD (Supplemental Table 8). Likewise, our previous reports on the associations of metabolic risk factors (i.e., adiposity and diabetes) with NAFLD in CKB were consistent with those in previous prospective studies using imaging or biopsy to ascertain NAFLD (**Supplemental Table 9**). Second, another limitation is the lack of validation of findings on metabolomics and NAFLD using more accurate diagnostic tools, such as biopsy. However, we showed consistent patterns of metabolomic biomarkers associated with NAFLD compared with hospital-based case-control studies of biopsy-diagnosed NAFLD (Supplemental Table 8). Although biopsy is unfeasible in large-scale cohort studies, future studies warrant adopting noninvasive tools to diagnose NAFLD with greater accuracy, such as controlled attenuation parameter and magnetic resonance spectroscopy (60). Third, individuals hospitalized for NAFLD may have more cardiometabolic comorbidities including CVD and diabetes. However, we found similar associations of adiposity with metabolites and of metabolites with NAFLD among participants without and with cardiometabolic comorbidities at baseline (Supplemental Figure 5). Fourth, we were not able to assess the effects of subclinical NAFLD developed at baseline on metabolic profiles, nor histological changes after NAFLD diagnosis and their associations with repeated measures of blood metabolomics due to lack of histological assessment. A previous study has suggested that histological progression in NAFLD patients is associated with metabolic changes, particularly lipidomics (61). Fifth, the sample size in the current study is relatively small because of the exploratory nature of the study.

In summary, our study in a relatively lean Chinese population shows that adiposity was associated with a range of metabolomic biomarkers, with concordant associations for both observational and genetic estimates. Some of the BMI-associated metabolomic biomarkers were also associated with risk of NAFLD. By integrating genomics and metabolomics, the current study findings suggest that several metabolites might lie on the causal pathway between BMI and NAFLD. Our findings provide insights into the metabolomic disturbances and pathophysiological mechanisms linking adiposity and NAFLD. Additional investigations are warranted to further characterize the relation of these metabolites with NAFLD and to evaluate their potential utility in risk prediction.

## Funding

The CKB baseline survey and the first re-survey were supported by a grant from the Kadoorie Charitable Foundation in Hong Kong. The long-term follow-up is supported by grants from the UK Wellcome Trust (212946/Z/18/Z, 202922/Z/16/Z, 104085/Z/14/Z, 088158/Z/09/Z), National Natural Science Foundation of China (91846303, 91843302, 81390540, 81390541, 81390544), and Chinese Ministry of Science and Technology (2011BAI09B01). This work was supported by grants (2016YFC0900500, 2016YFC0900501, 2016YFC0900504, 2016YFC1303904) from the National Key R&D Program of China. Dr Holmes is supported by a British Heart Foundation Intermediate Clinical Research Fellowship (FS/18/23/33512) and the National Institute for Health Research Oxford Biomedical Research Centre. Dr Pang acknowledges support from the China Postdoctoral Science Foundation (2019TQ0008, 2020M670071). The metabolomics analysis was funded by a NDPH Pump Priming Award. The funders had no role in the study design, data collection, data analysis and interpretation, writing of the report, or the decision to submit the article for publication.

## Supplementary Material

nqab392_Supplemental_File

## Data Availability

Data described in the article, code book, and analytic code will be made available from CKB upon request (https://www.ckbiobank.org/site/Data+Access) upon request pending application and approval. The datasets generated and/or analyzed during the current study are not publicly available but are available from the corresponding author on reasonable request.

## Notes

The China Kadoorie Biobank baseline survey and the first re-survey were supported by a grant from the Kadoorie Charitable Foundation in Hong Kong. The long-term follow-up is supported by grants from the UK Wellcome Trust (212946/Z/18/Z, 202922/Z/16/Z, 104085/Z/14/Z, 088158/Z/09/Z), the National Natural Science Foundation of China (91846303, 91843302, 81390540, 81390541, 81390544), and the Chinese Ministry of Science and Technology (2011BAI09B01). This work was supported by grants (2016YFC0900500, 2016YFC0900501, 2016YFC0900504, 2016YFC1303904) from the National Key R&D Program of China. MVH is supported by a British Heart Foundation Intermediate Clinical Research Fellowship (FS/18/23/33512) and the National Institute for Health Research Oxford Biomedical Research Centre. YP acknowledges support from the China Postdoctoral Science Foundation (2019TQ0008, 2020M670071). The metabolomics analysis was funded by a Nuffield Department of Population Health Pump Priming Award. The funders had no role in the study design, data collection, data analysis and interpretation, writing of the report, or the decision to submit the article for publication.

LL, ZC, and MVH are joint senior authors.

Supplemental Tables 1–9 and Supplemental Figures 1–8 are available from the “Supplementary data” link in the online posting of the article at https://academic.oup.com/ajcn/.

Abbreviations used: BBJ, Biobank Japan; BCAA, branched-chain amino acid; CHD, coronary heart disease; CKB, China Kadoorie Biobank; CT, computed tomography; CVD, cardiovascular disease; DAG, diacylglycerol; FDR, false discovery rate; HC, hip circumference; ICD-10, International Classification of Diseases, 10th Revision; IPD, individual participant–level data; LPC, lyso-phosphatidylcholine; MAF, minor allele frequency; MHO, metabolically healthy obesity; MUO, metabolically unhealthy obesity; NAFLD, nonalcoholic fatty liver disease; PC, phosphatidylcholine; RPG, random plasma glucose; SBP, systolic blood pressure; SM, sphingomyelin; SNP, single nucleotide polymorphism; WC, waist circumference.
