# Supplementary material for: Adiposity, metabolomic biomarkers, and risk of nonalcoholic fatty liver disease: a case-cohort study
Source: Am J Clin Nutr. 2021 Dec 13;115(3):799–810. doi: 10.1093/ajcn/nqab392 (PMC8895224; doi:10.1093/ajcn/nqab392)
Supplement: nqab392_Supplemental_File [file nqab392_supplemental_file.pdf]

On-line Supplementary Materials

Adiposity, metabolomic biomarkers and risk of non-alcoholic fatty liver disease: a case-cohort study

American Journal of Clinical Nutrition

Yuanjie Pang, DPhil[1], Christiana Kartsonaki, DPhil[2,3], Jun Lv, PhD[1,4], Iona Y. Millwood, DPhil[2,3], Zammy Fairhurst-Hunter, DPhil[2], Iain Turnbull, MRCP[2], Fiona Bragg, DPhil[2,3], Michael R. Hill, DPhil[2], Canqing Yu, PhD[1,4], Yu Guo, MSc[5], Yiping Chen, DPhil[2,3], Ling Yang, PhD[2,3] Robert Clarke, FRCP, MD[2], Robin G. Walters, PhD[2,3], Ming Wu, PhD[6], Junshi Chen, MD[7], Liming Li, MD[1,4*], Zhengming Chen, DPhil[2,3*], Michael V. Holmes, PhD[2,3,8*]

* Joint senior authors

1. Department of Epidemiology and Biostatistics, School of Public Health, Peking University, 38 Xueyuan Road, Beijing 100191, China
2. Clinical Trial Service Unit & Epidemiological Studies Unit (CTSU), Nuffield Department of Population Health, Big Data Institute Building, Roosevelt Drive, University of Oxford, UK
3. Medical Research Council Population Health Research Unit (MRC PHRU) at the University of Oxford, Nuffield Department of Population Health, University of Oxford, UK
4. Peking University Center for Public Health and Epidemic Preparedness & Response (PKU-PHEPR), Peking University, 38 Xueyuan Road, Beijing 100191, China
5. Chinese Academy of Medical Sciences, 9 Dongdan San Tiao, Beijing 100730, China
6. Jiangsu Center for Disease Control and Prevention, 172 Jiangsu Road, Nanjing 210009, China
7. National Center for Food Safety Risk Assessment, 37 Guangqu Road, Beijing 100021, China
8. National Institute for Health Research Oxford Biomedical Research Centre, Oxford University Hospital, Old Road, Oxford OX3 7LE, UK

Address for correspondence:

Dr Christiana Kartsonaki
Medical Research Council
Population Health Research Unit
University of Oxford
Big Data Institute
Oxford, OX3 7LF, UK
Tel: 44-1865-743644
Fax: 44-1865-743985
christiana.kartsonaki@ndph.ox.ac.uk

Table of Content

Supplementary Figure 1. Histograms of log-transformed distribution of individual metabolites........ 3

Supplementary Figure 2. Sensitivity analyses comparing different MR analytic approaches 15

Supplementary Figure 3. Associations of BMI and WCadjBMI with metabolic biomarkers 16

Supplementary Figure 4. Observational and genetic associations of BMI with selected metabolic biomarkers........ 17

Supplementary Figure 5. Subgroup analyses of the observational associations of BMI with metabolomics and metabolomics with NAFLD........ 18

Supplementary Figure 6. Observational associations of BMI with metabolomics and metabolomics with NAFLD, with additional adjustment........ 19

Supplementary Figure 7. Associations of BMI, metabolomics, and NAFLD risk in CKB for selected metabolic biomarkers identified by previous studies........ 20

Supplementary Figure 8. Associations of BMI, metabolomics, and NAFLD risk in CKB for selected metabolic biomarkers identified by previous studies........ 21

Supplementary Table 1. Information of 1208 metabolic biomarkers measured by Metabolon 22

Supplementary Table 2. Genetic variants associated with BMI in the GIANT consortium. 63

Supplementary Table 3. Associations of potential confounders with BMI GRS........ 84

Supplementary Table 4. Observational associations of adiposity with metabolic biomarkers 85

Supplementary Table 5. Observational associations of metabolic biomarkers with NAFLD 121

Supplementary Table 6. Genetic associations of BMI with metabolic biomarkers........ 157

Supplementary Table 7. Multivariable analyses of the associations between metabolic biomarkers with risk of NAFLD........ 166

Supplementary Table 8. Previous studies on metabolic biomarkers with NAFLD........ 167

Supplementary Table 9. Previous prospective studies on metabolic risk factors with NAFLD 169

Supplementary Figure 1. Histograms of log-transformed distribution of individual metabolites

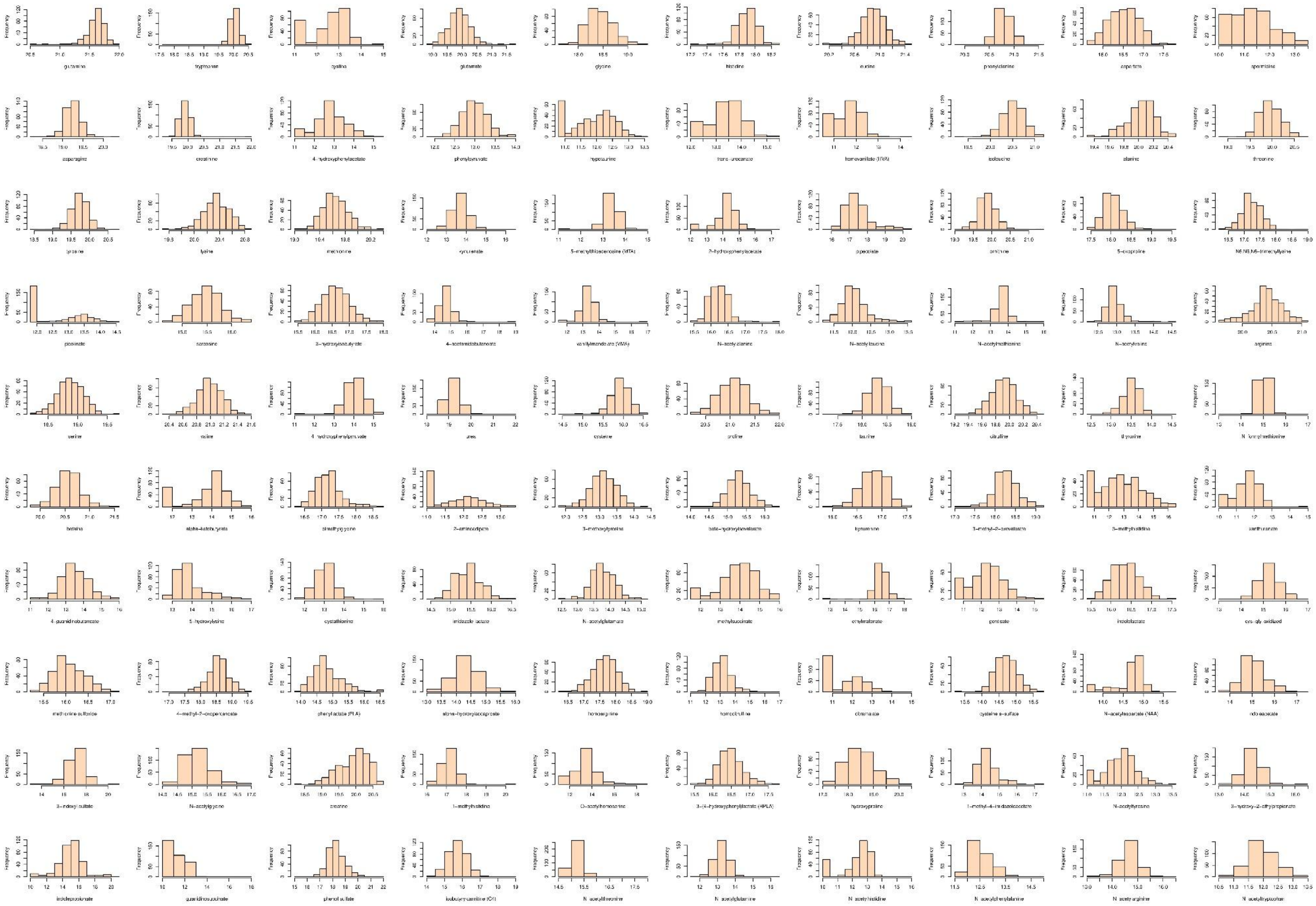

Supplementary Figure 1. continued

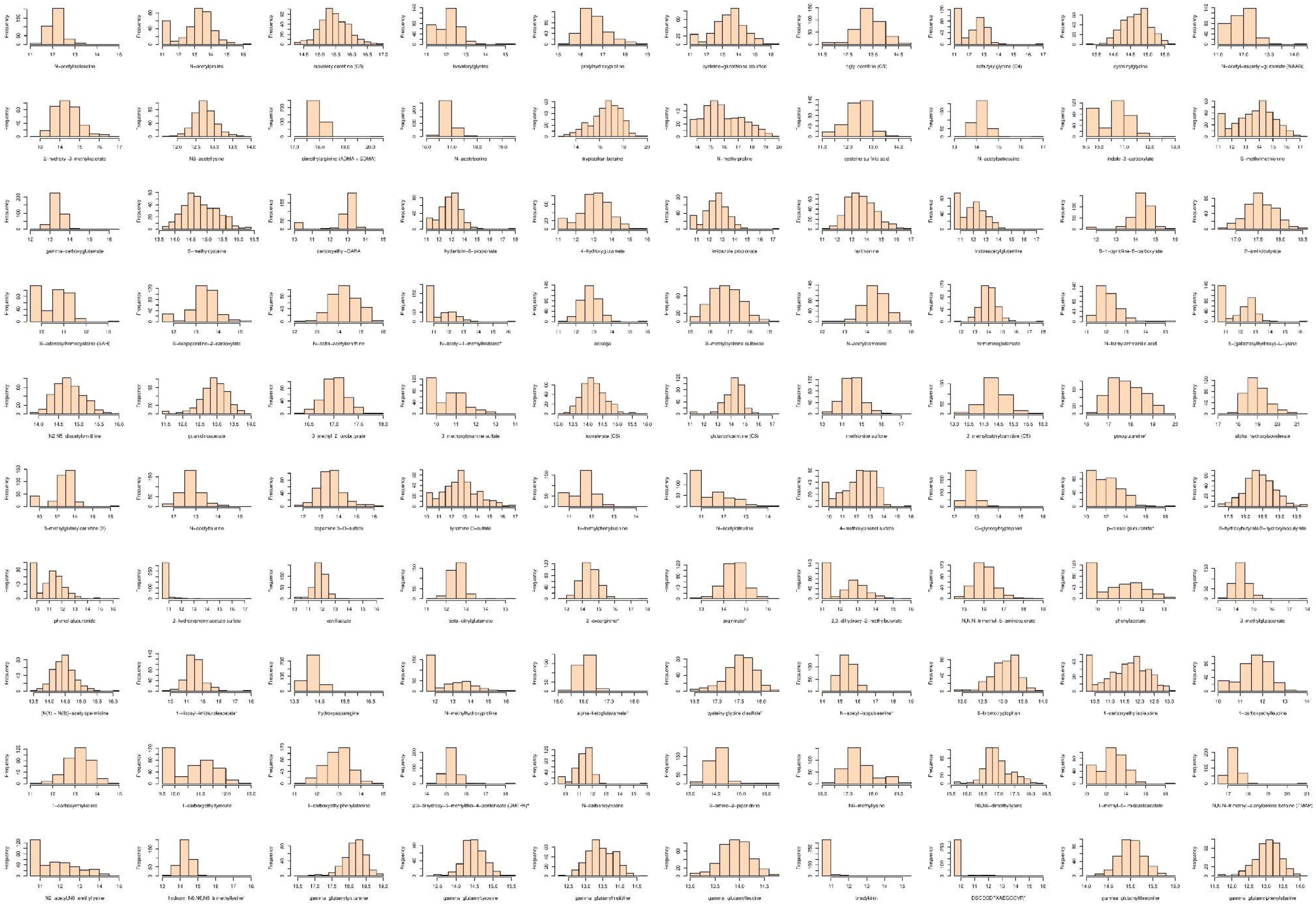

Supplementary Figure 1. continued

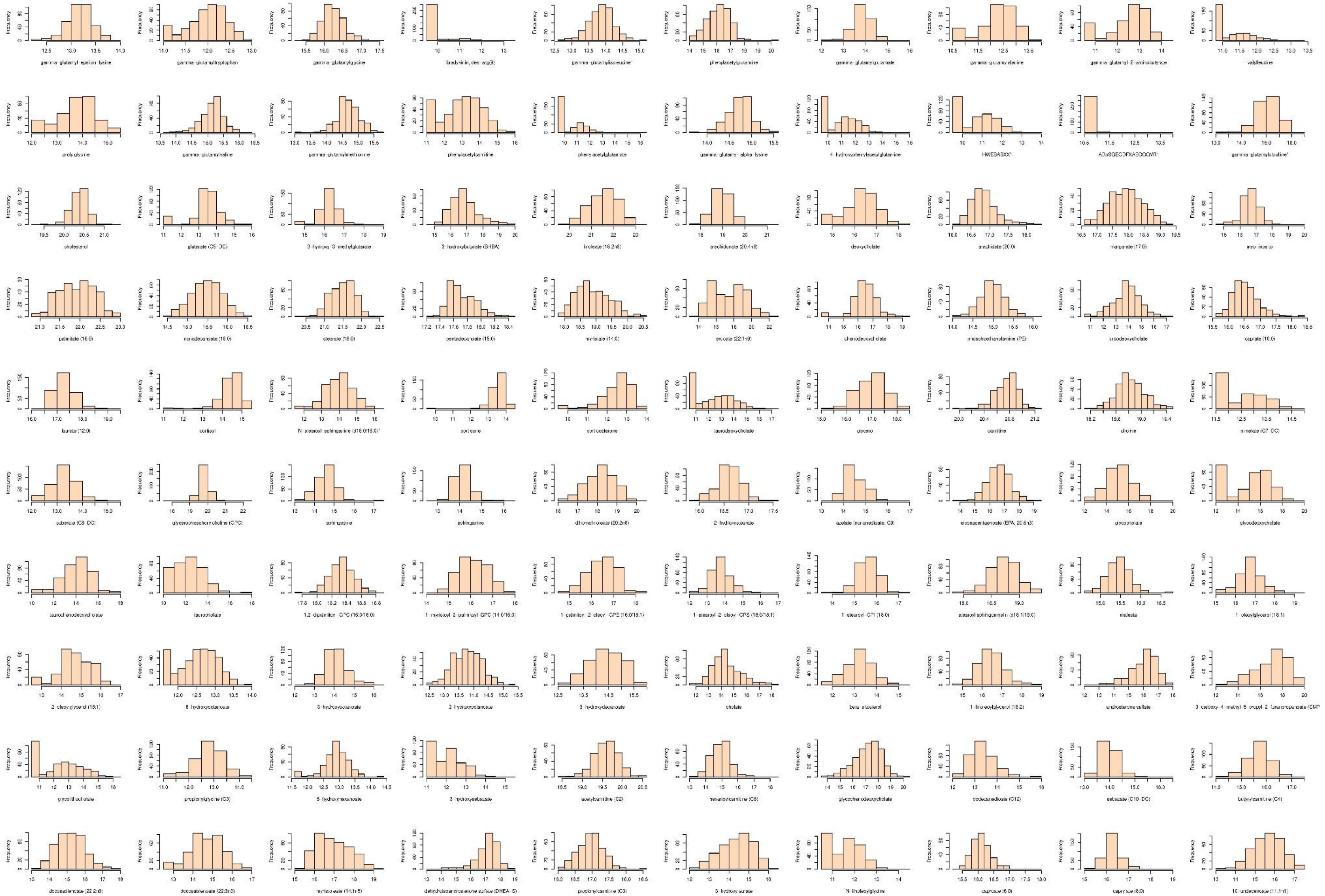

Supplementary Figure 1. continued

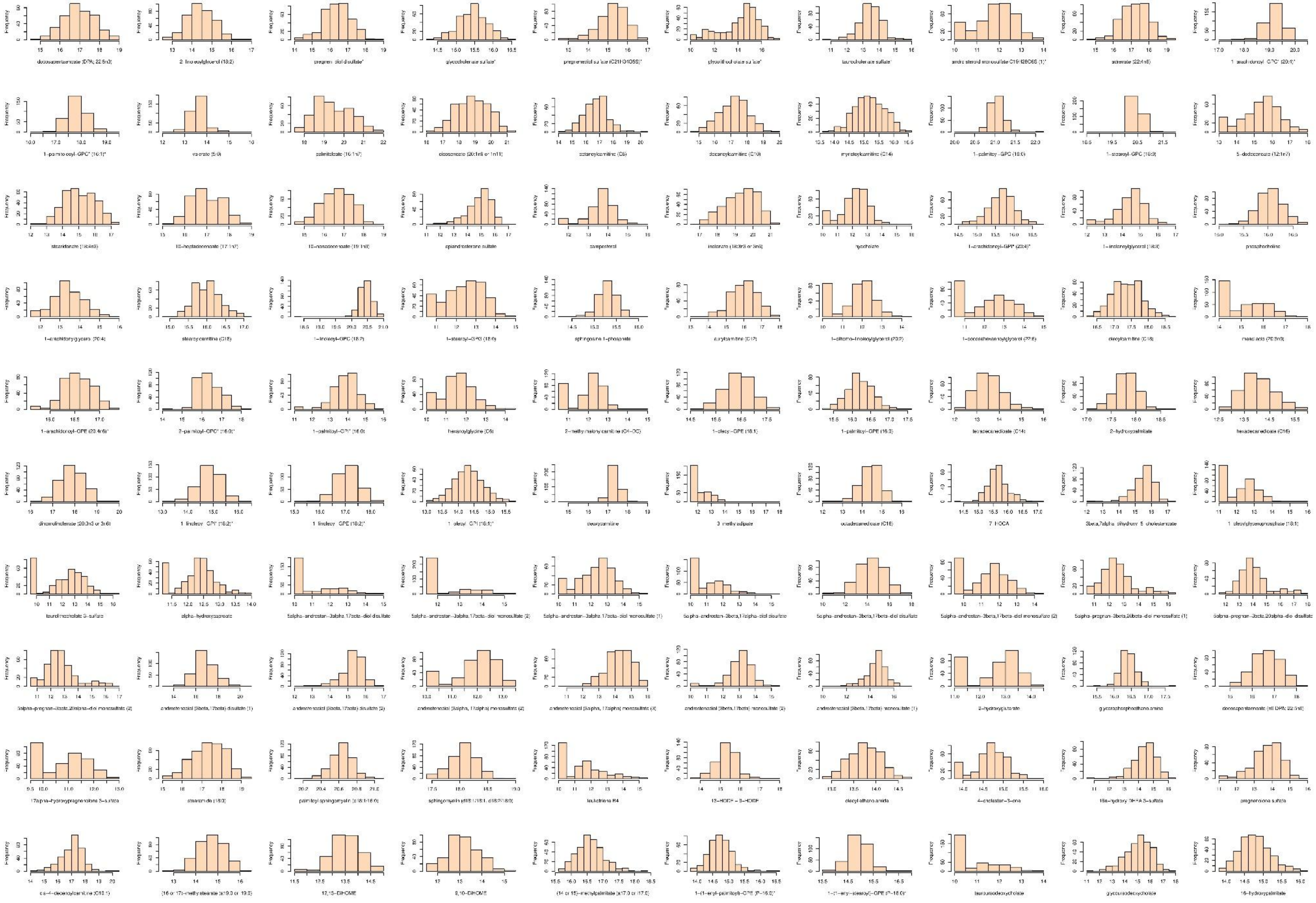

Supplementary Figure 1. continued

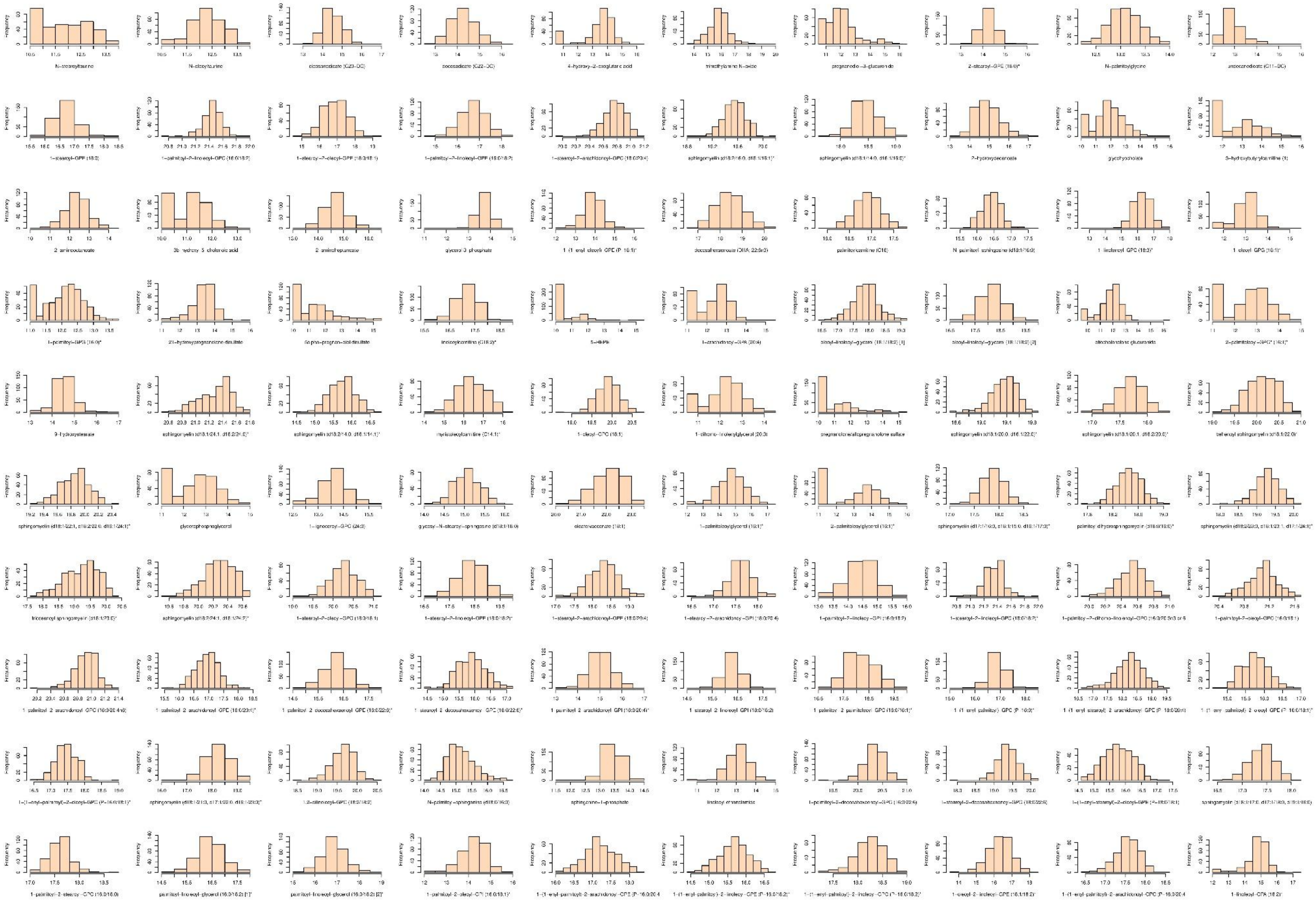

Supplementary Figure 1. continued

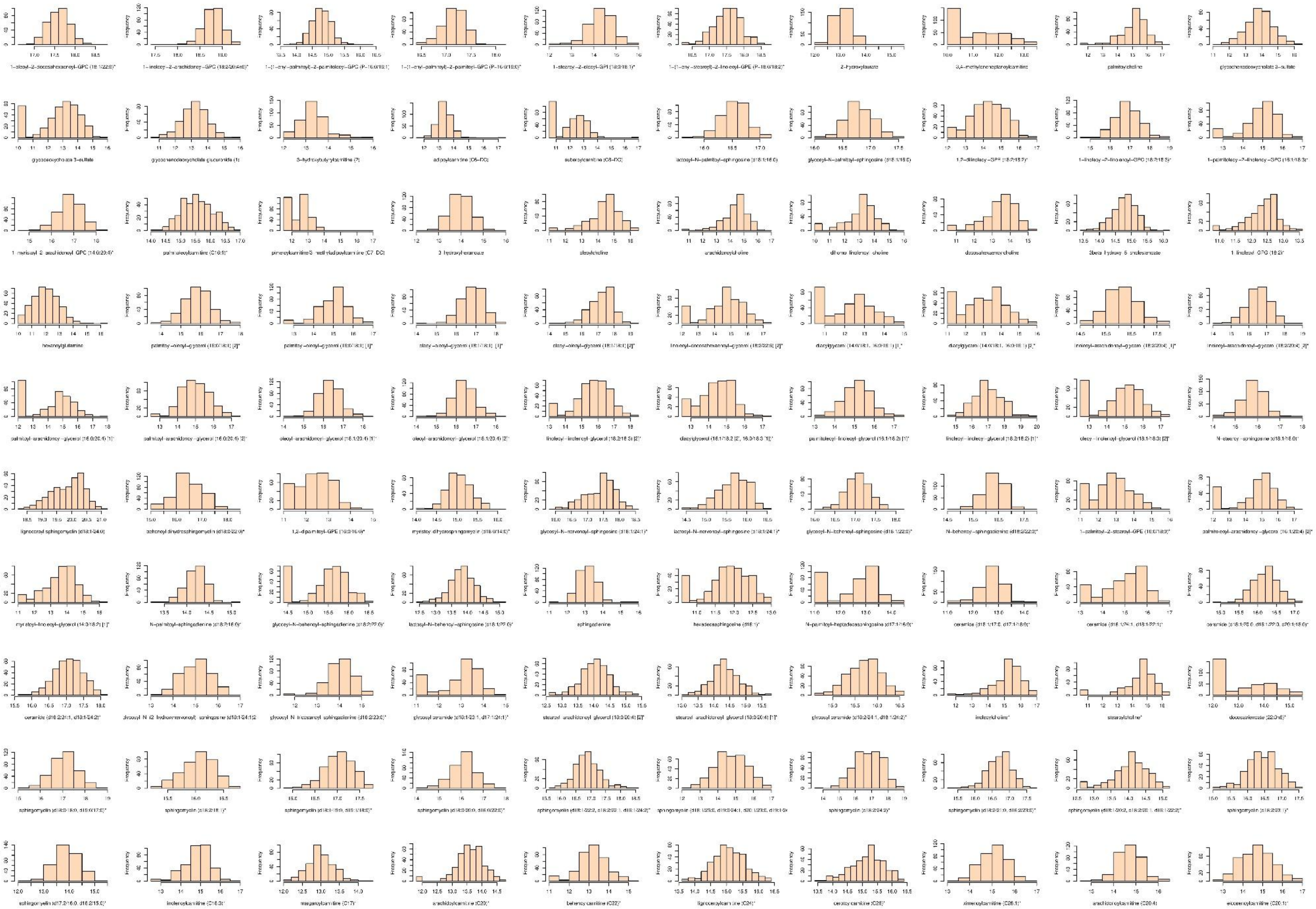

Supplementary Figure 1. continued

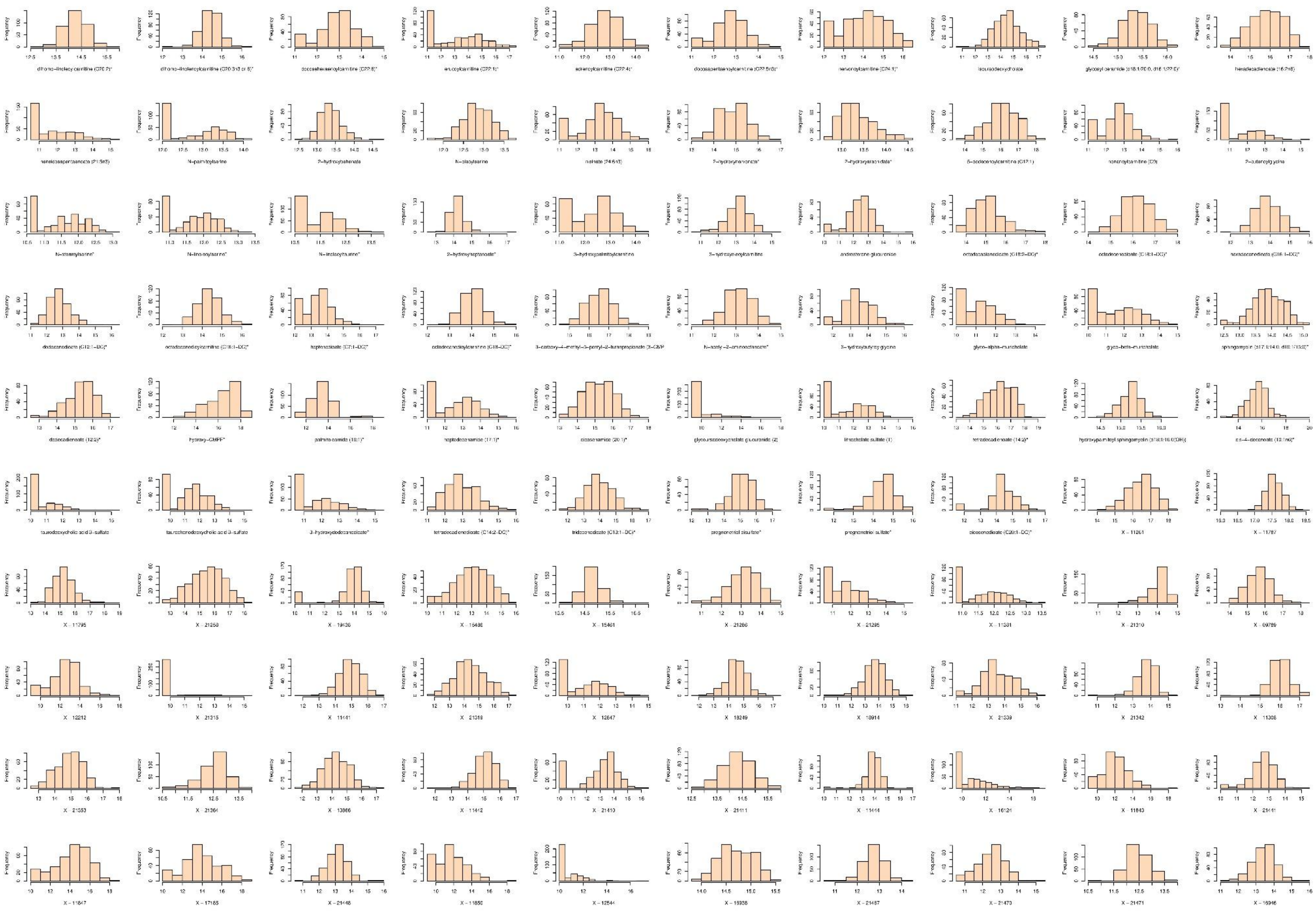

Supplementary Figure 1. continued

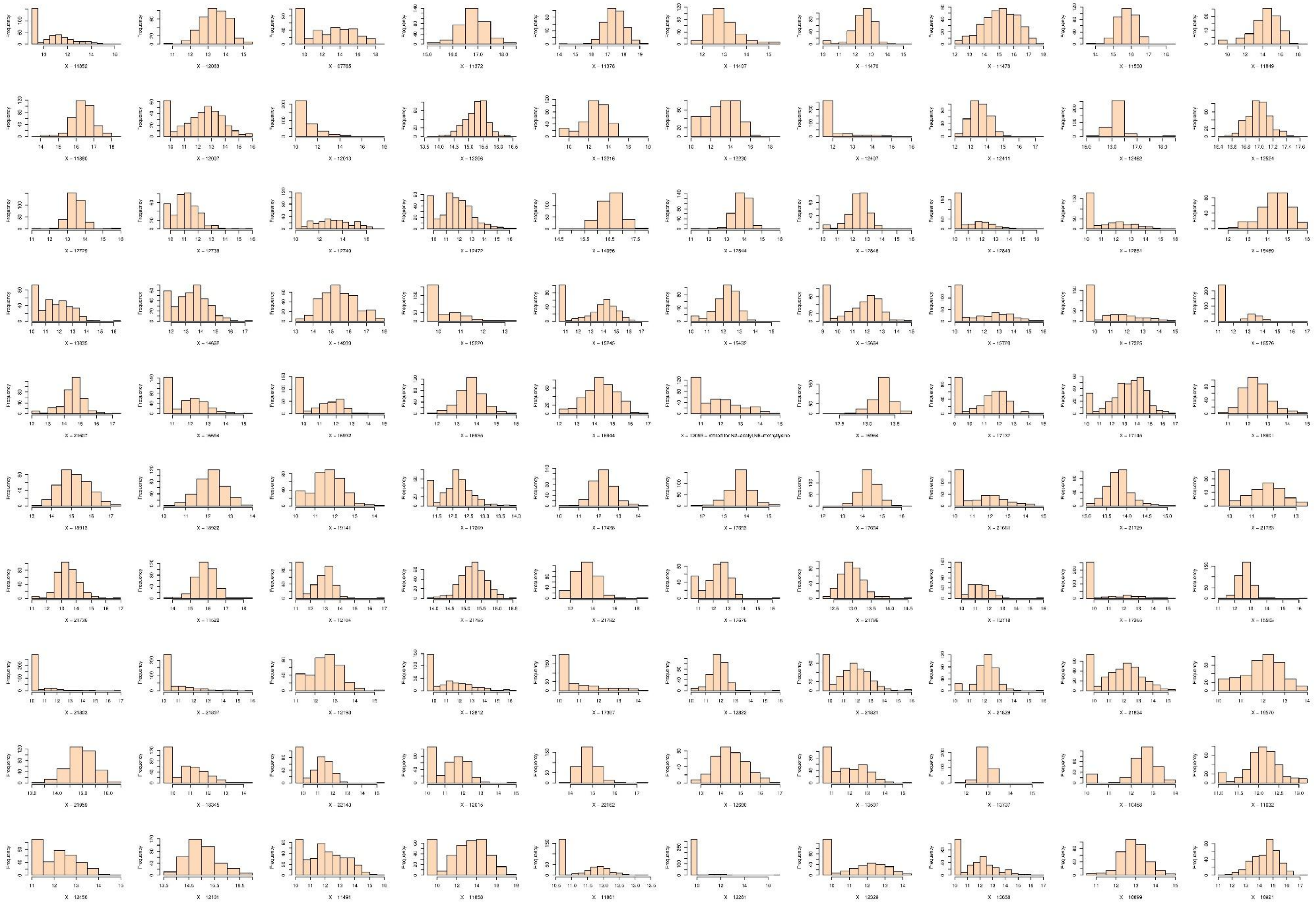

Supplementary Figure 1. continued

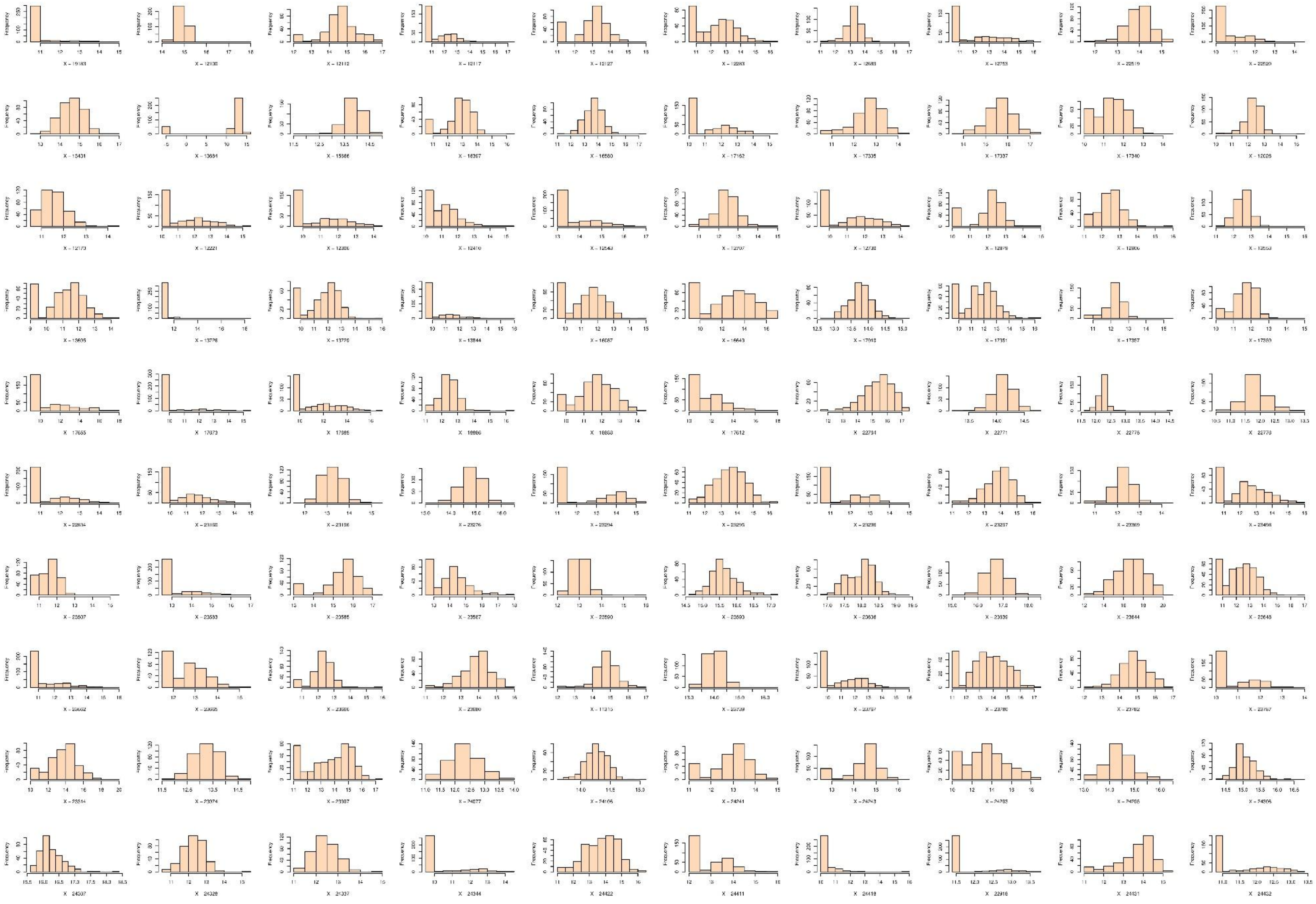

Supplementary Figure 1. continued

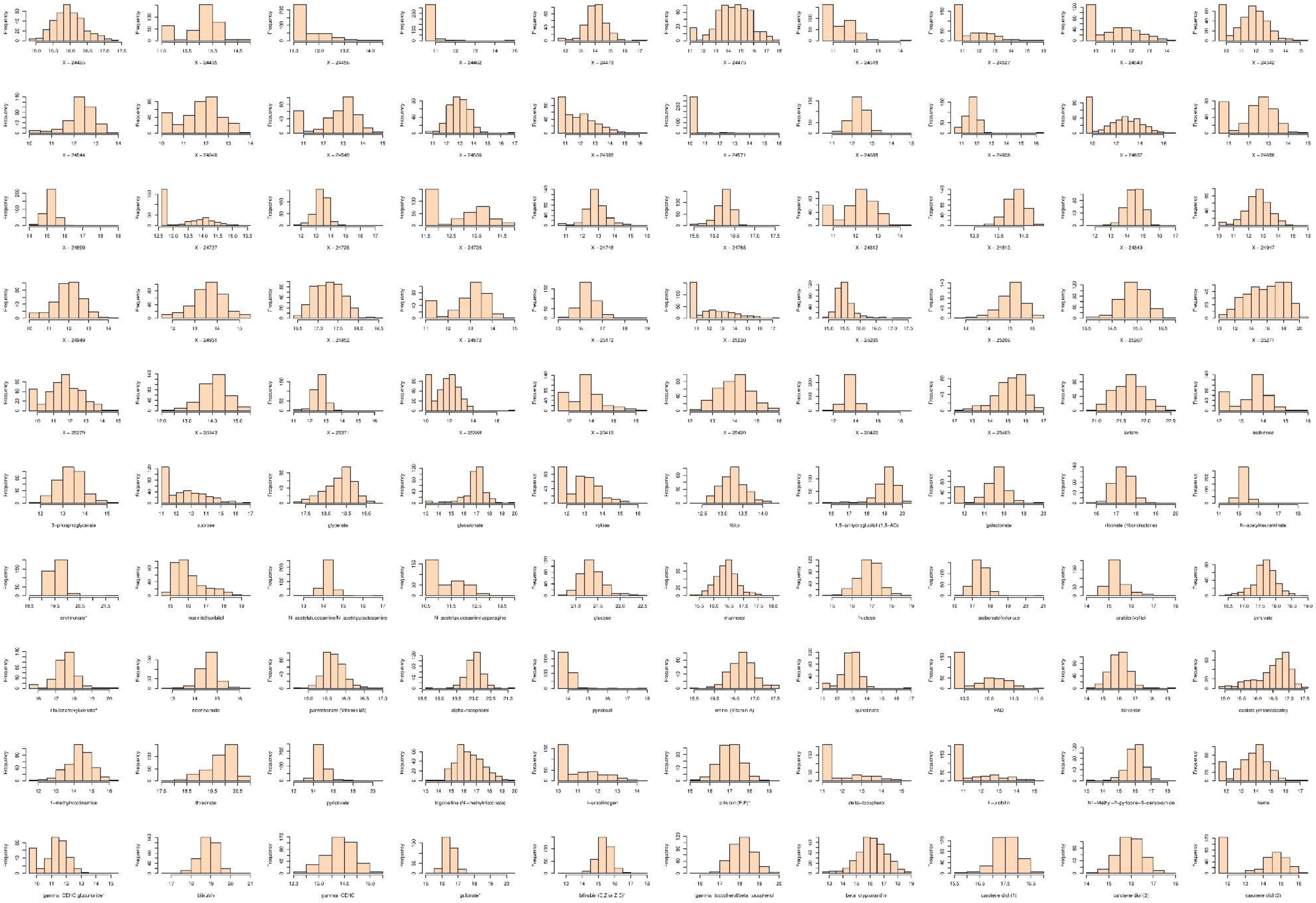

Supplementary Figure 1. continued

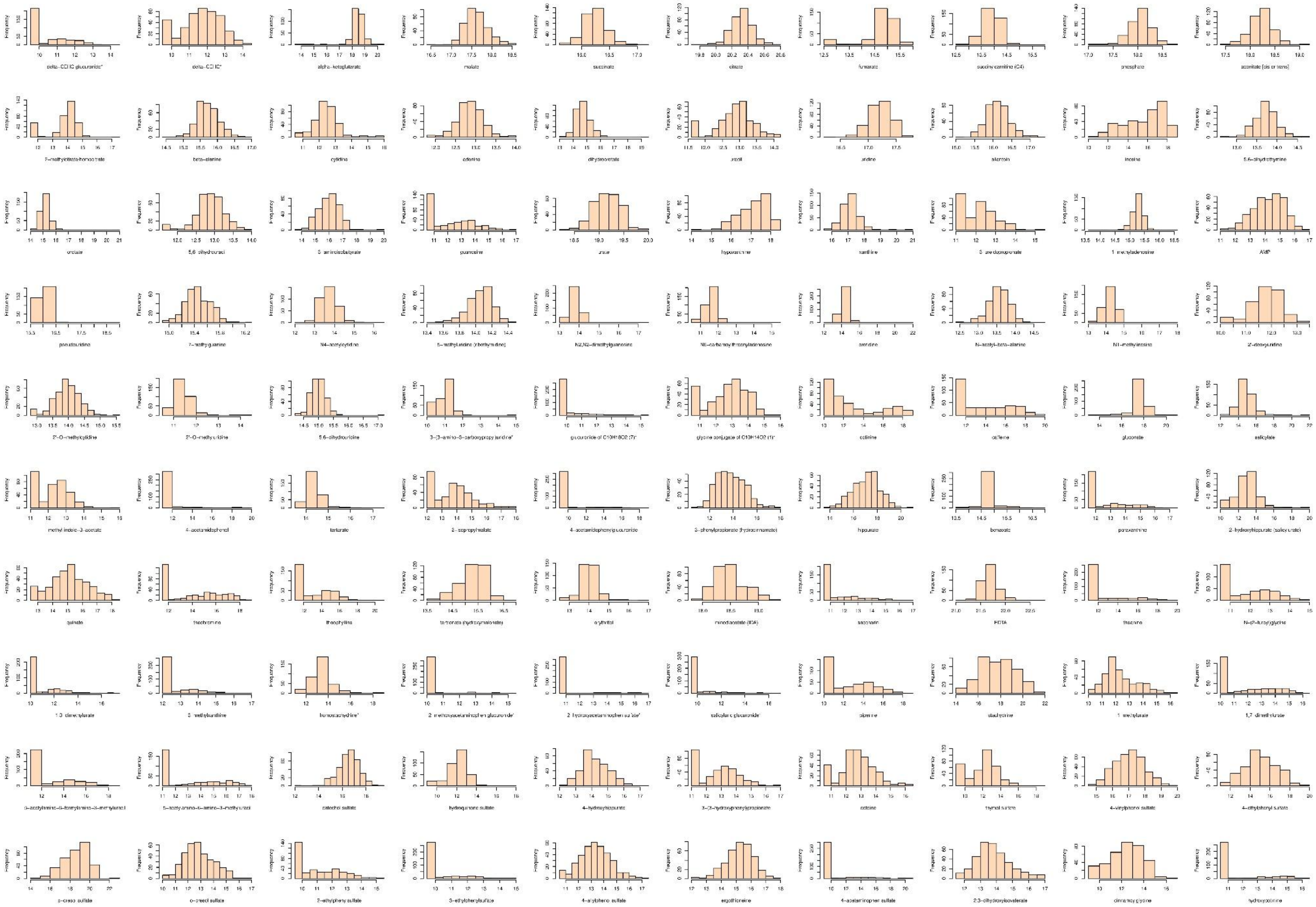

Supplementary Figure 1. continued

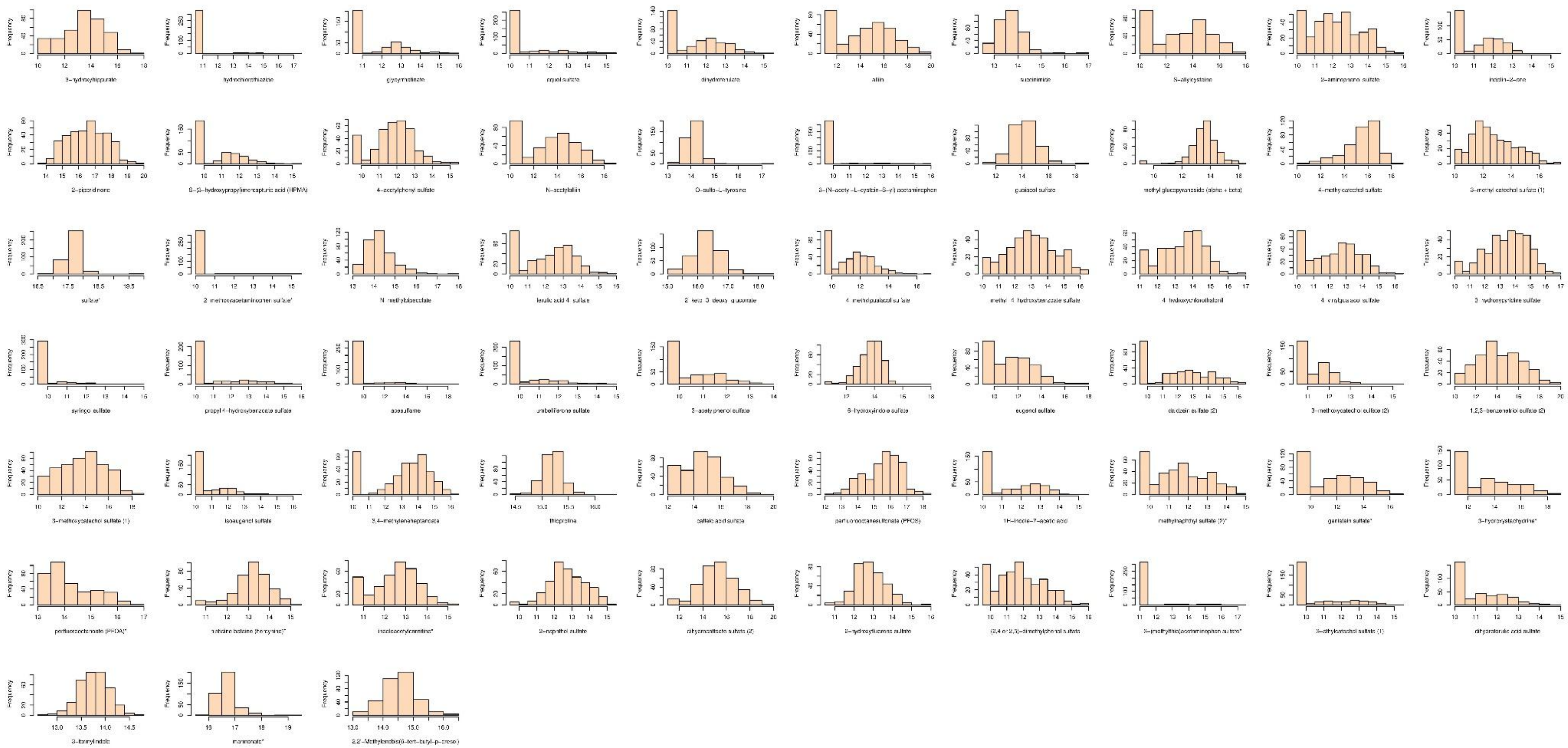

Supplementary Figure 2. Sensitivity analyses comparing different MR analytic approaches

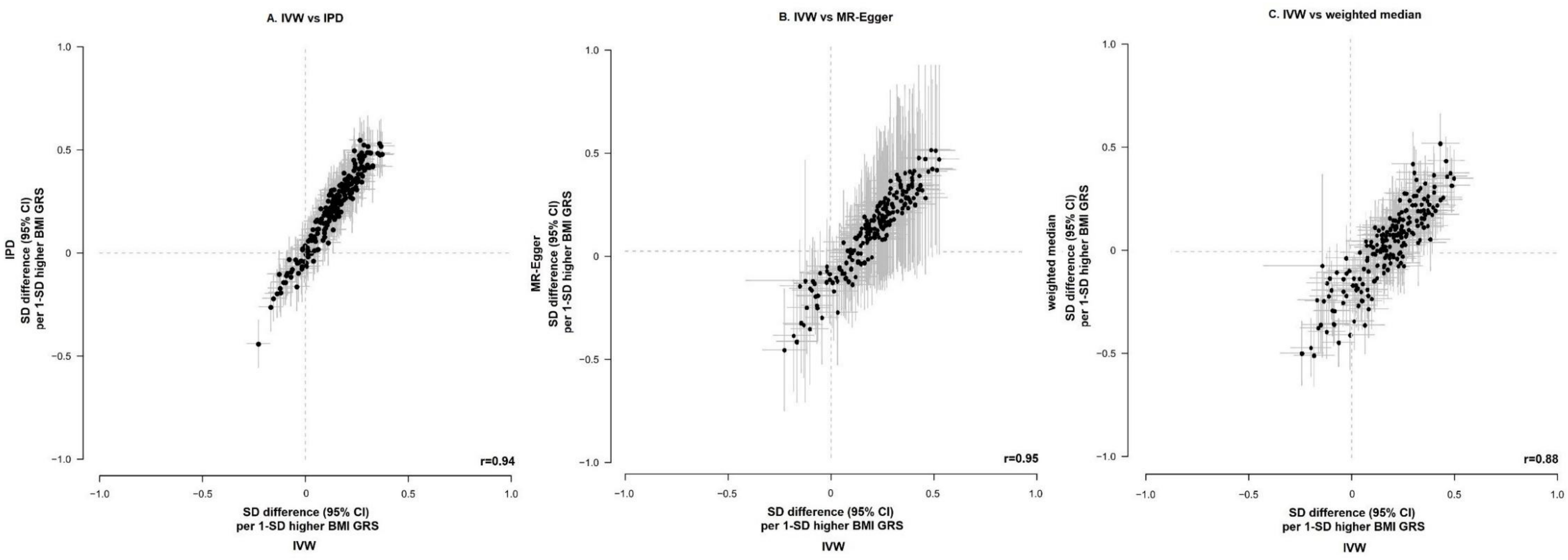

Supplementary Figure 3. Associations of BMI and WCadjBMI with metabolic biomarkers

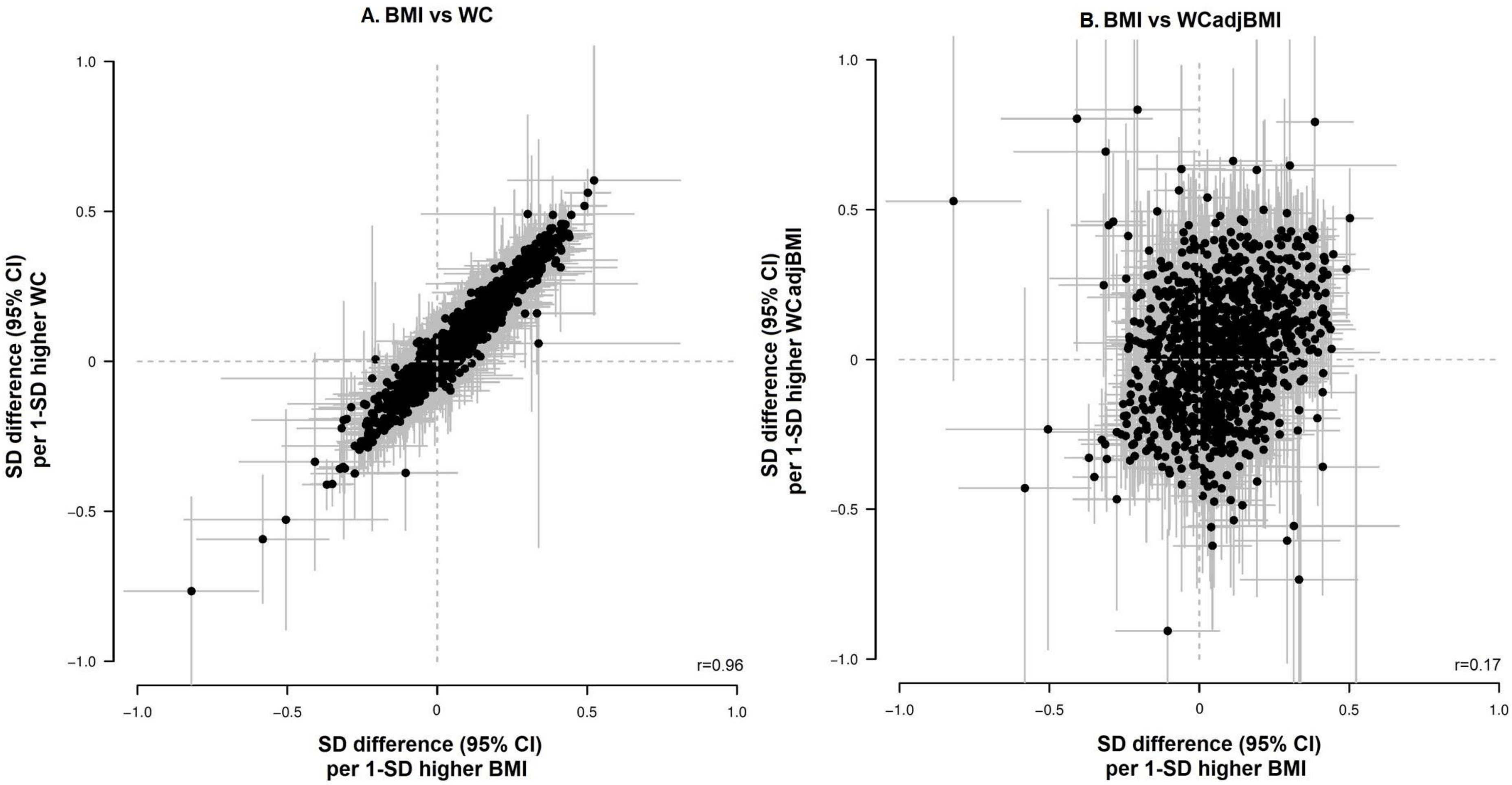

Supplementary Figure 4. Observational and genetic associations of BMI with selected metabolic biomarkers

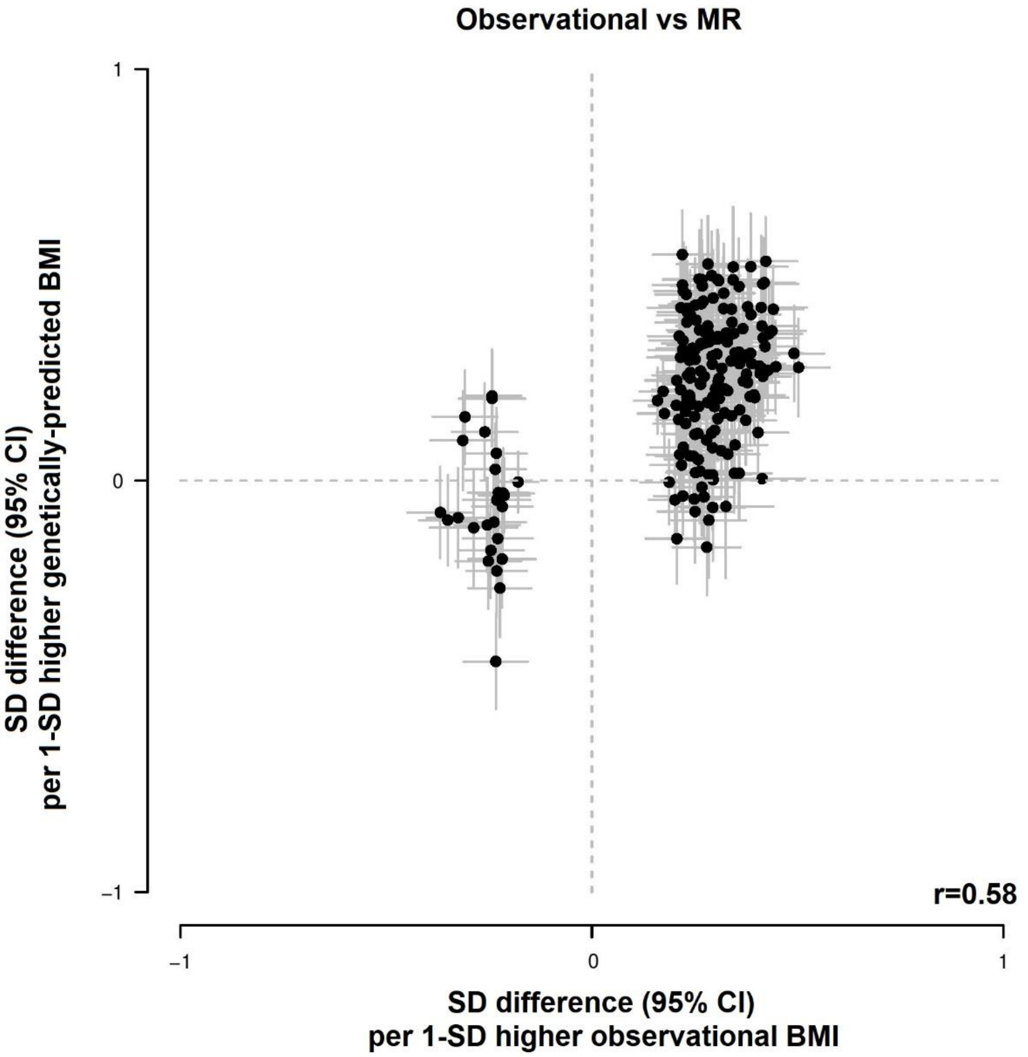

Supplementary Figure 5. Subgroup analyses of the observational associations of BMI with metabolomics and metabolomics with NAFLD

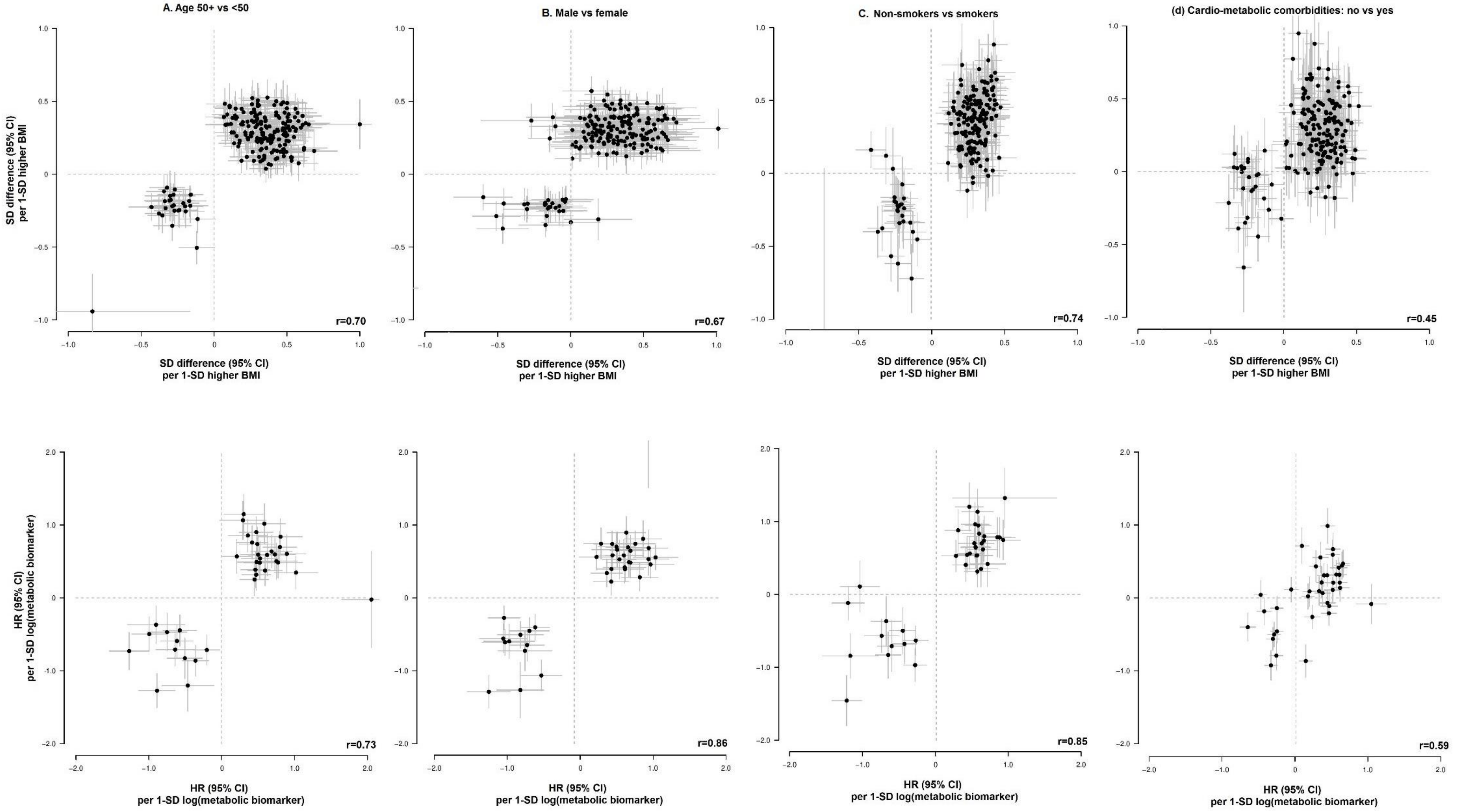

Supplementary Figure 6. Observational associations of BMI with metabolomics and metabolomics with NAFLD, with additional adjustment

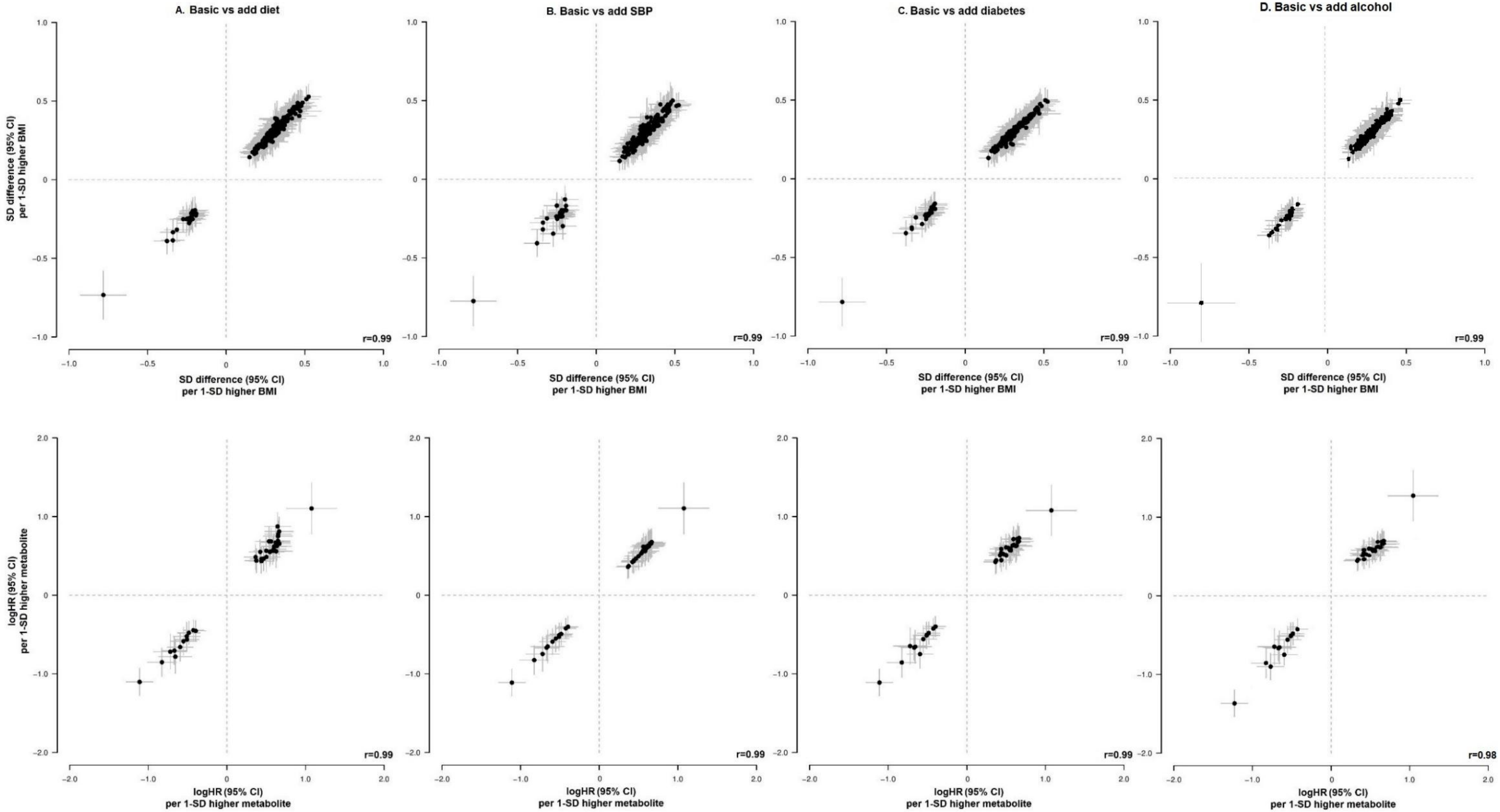

Supplementary Figure 7. Associations of BMI, metabolomics, and NAFLD risk in CKB for selected metabolic biomarkers identified by previous studies

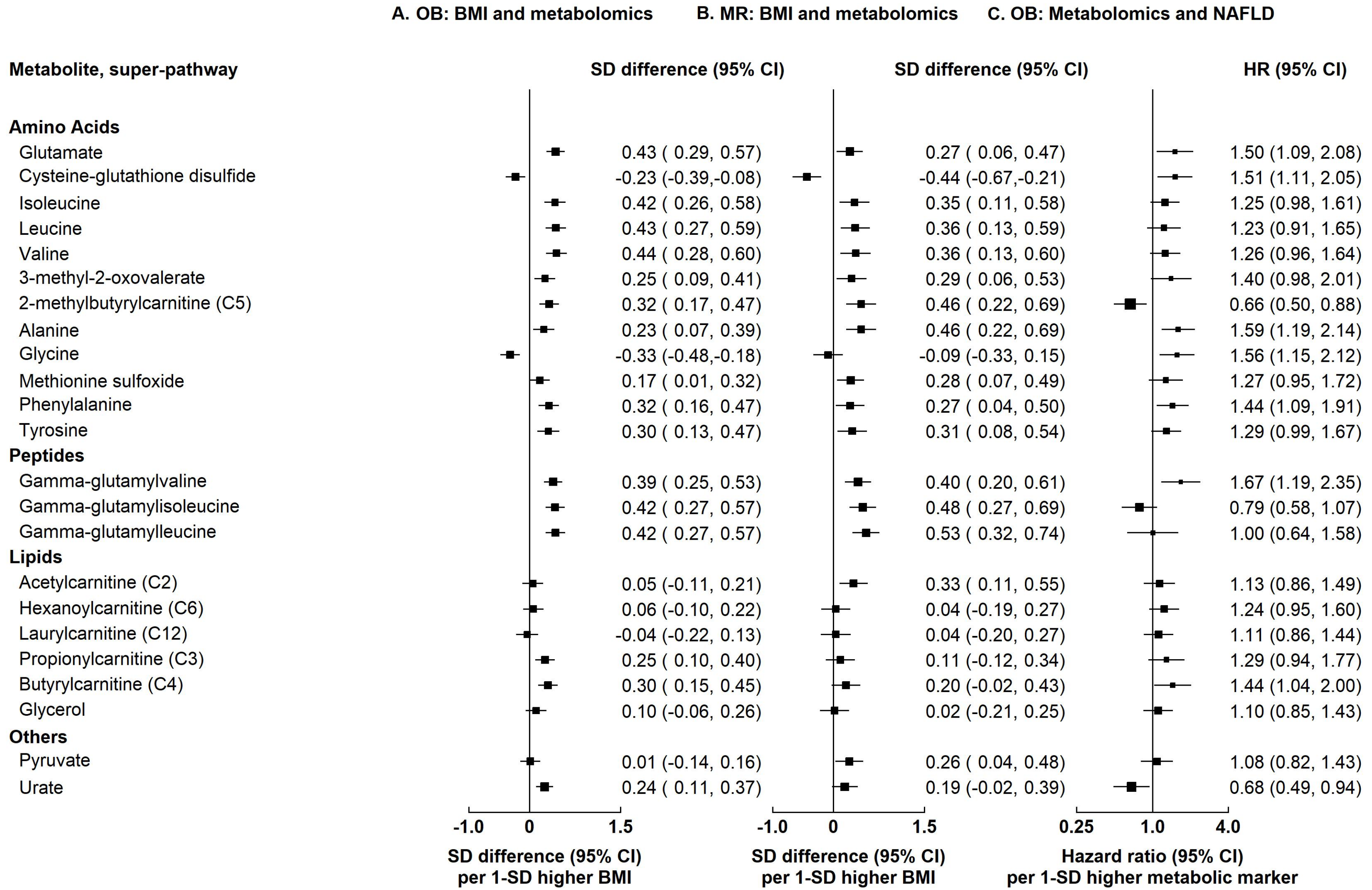

Supplementary Figure 8. Associations of BMI, metabolomics, and NAFLD risk in CKB for selected metabolic biomarkers identified by previous studies

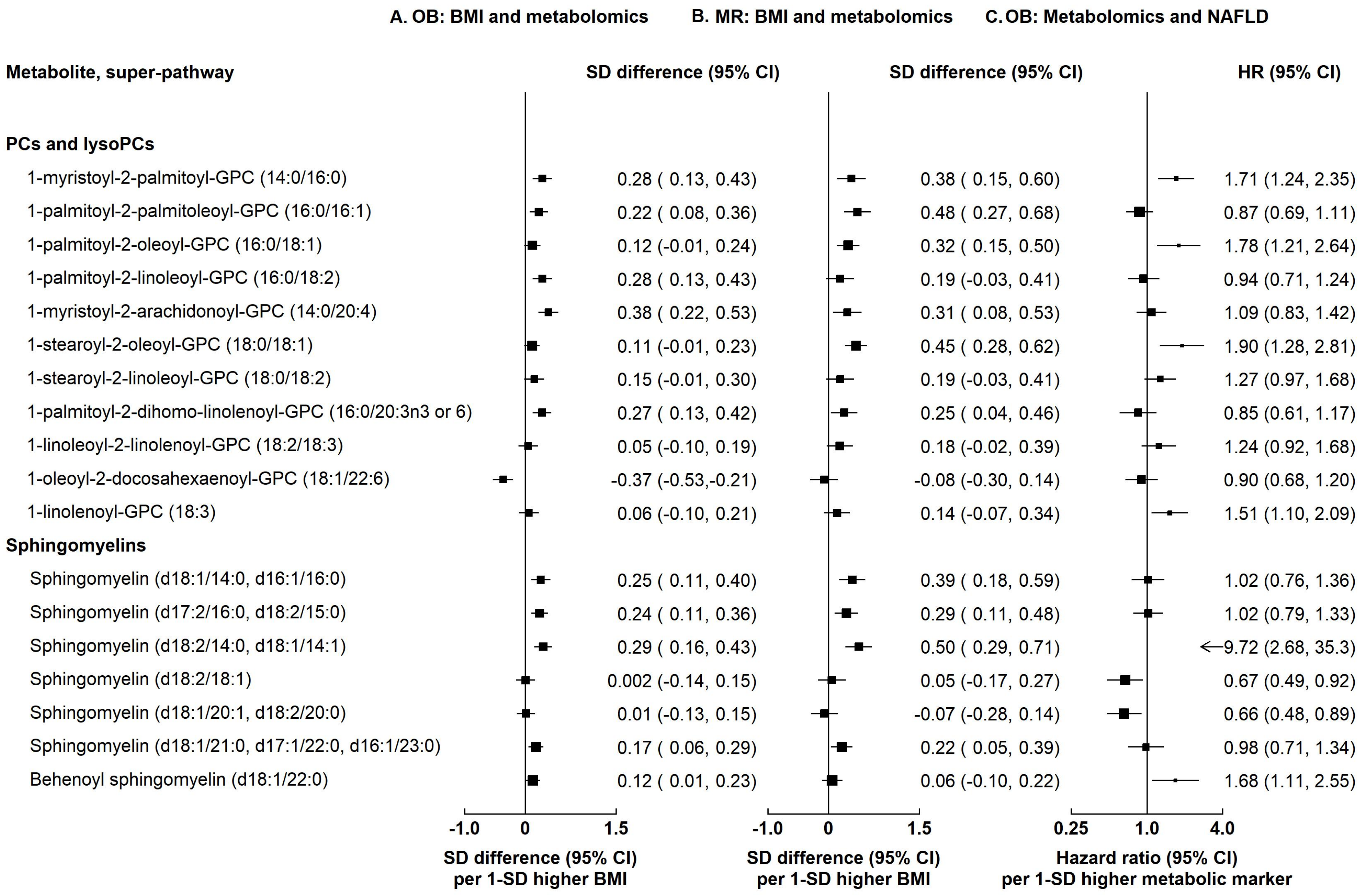

Supplementary Table 1. Information of 1208 metabolic biomarkers measured by Metabolon

Compound ID	Biochemical	Super-pathway	Sub-pathway	PuChem	CAS	Group HMDB
38768	(14 or 15)-methylpalmitate (a17:0 or i17:0)	Lipid	Fatty Acid, Branched	17903417		
38296	(16 or 17)-methylstearate (a19:0 or i19:0)	Lipid	Fatty Acid, Branched	3083779	2724-59-6	HMDB37397
62533	(2,4 or 2,5)-dimethylphenol sulfate	Xenobiotics	Food Component/Plant			
57814	(N(1) + N(8))-acetylspermidine	Amino Acid	Polyamine Metabolism			
44621	1-(1-enyl-oleoyl)-GPE (P-18:1)*	Lipid	Lysoplasmalogen			
52689	1-(1-enyl-palmitoyl)-2-arachidonoyl-GPC (P-16:0/20:4)*	Lipid	Plasmalogen			HMDB11220
52673	1-(1-enyl-palmitoyl)-2-arachidonoyl-GPE (P-16:0/20:4)*	Lipid	Plasmalogen			HMDB11352
52682	1-(1-enyl-palmitoyl)-2-linoleoyl-GPC (P-16:0/18:2)*	Lipid	Plasmalogen			HMDB11211
52677	1-(1-enyl-palmitoyl)-2-linoleoyl-GPE (P-16:0/18:2)*	Lipid	Plasmalogen			HMDB11343
52478	1-(1-enyl-palmitoyl)-2-oleoyl-GPC (P-16:0/18:1)*	Lipid	Plasmalogen			
52477	1-(1-enyl-palmitoyl)-2-oleoyl-GPE (P-16:0/18:1)*	Lipid	Plasmalogen			HMDB11342
52713	1-(1-enyl-palmitoyl)-2-palmitoleoyl-GPC (P-16:0/16:1)*	Lipid	Plasmalogen			HMDB11207
52716	1-(1-enyl-palmitoyl)-2-palmitoyl-GPC (P-16:0/16:0)*	Lipid	Plasmalogen	11146967		HMDB11206
52474	1-(1-enyl-palmitoyl)-GPC (P-16:0)*	Lipid	Lysoplasmalogen	10917802		HMDB10407
39270	1-(1-enyl-palmitoyl)-GPE (P-16:0)*	Lipid	Lysoplasmalogen			
52475	1-(1-enyl-stearoyl)-2-arachidonoyl-GPE (P-18:0/20:4)*	Lipid	Plasmalogen	9547058		HMDB05779
52748	1-(1-enyl-stearoyl)-2-linoleoyl-GPE (P-18:0/18:2)*	Lipid	Plasmalogen			HMDB11376
52614	1-(1-enyl-stearoyl)-2-oleoyl-GPE (P-18:0/18:1)	Lipid	Plasmalogen		144371-68-6	HMDB11375
39271	1-(1-enyl-stearoyl)-GPE (P-18:0)*	Lipid	Lysoplasmalogen			
48762	1,2,3-benzenetriol sulfate (2)	Xenobiotics	Chemical			
52603	1,2-dilinoleoyl-GPC (18:2/18:2)	Lipid	Phosphatidylcholine (PC)	5288075	998-06-1	HMDB08138
53174	1,2-dilinoleoyl-GPE (18:2/18:2)*	Lipid	Phosphatidylethanolamine (PE)	9546812		HMDB09093
19130	1,2-dipalmitoyl-GPC (16:0/16:0)	Lipid	Phosphatidylcholine (PC)	452110	63-89-8	HMDB00564
57341	1,2-dipalmitoyl-GPE (16:0/16:0)*	Lipid	Phosphatidylethanolamine (PE)	445468		HMDB08923
32391	1,3-dimethylurate	Xenobiotics	Xanthine Metabolism	70346	944-73-0	HMDB01857
20675	1,5-anhydroglucitol (1,5-AG)	Carbohydrate	Glycolysis, Gluconeogenesis, and Pyruvate Metabolism	64960	154-58-5	HMDB02712
34400	1,7-dimethylurate	Xenobiotics	Xanthine Metabolism	91611	33868-03-0	HMDB11103
33971	10-heptadecenoate (17:1n7)	Lipid	Long Chain Fatty Acid	5312435	29743-97-3	HMDB60038

33972	10-nonadecenoate (19:1n9)	Lipid	Long Chain Fatty Acid	5312513	73033-09-7	HMDB13622
32497	10-undecenoate (11:1n1)	Lipid	Medium Chain Fatty Acid	14891	1333-28-4	HMDB33724
47135	11-ketoetiocholanolone glucuronide	Lipid	Androgenic Steroids		17181-16-7	
38395	12,13-DiHOME	Lipid	Fatty Acid, Dihydroxy	10236635	263399-35-5	HMDB04705
37752	13-HODE + 9-HODE	Lipid	Fatty Acid, Monohydroxy	43013		
38168	16a-hydroxy DHEA 3-sulfate	Lipid	Androgenic Steroids			
39609	16-hydroxypalmitate	Lipid	Fatty Acid, Monohydroxy	10466	506-13-8	HMDB06294
37482	17alpha-hydroxypregnenolone 3-sulfate	Lipid	Pregnenolone Steroids	152971	28901-70-4	HMDB00416
46325	1-arachidonoyl-GPA (20:4)	Lipid	Lysophospholipid		799268-65-8	
33228	1-arachidonoyl-GPC* (20:4)*	Lipid	Lysophospholipid			HMDB10395
35186	1-arachidonoyl-GPE (20:4n6)*	Lipid	Lysophospholipid	42607465		HMDB11517
34214	1-arachidonoyl-GPI* (20:4)*	Lipid	Lysophospholipid			HMDB61690
34397	1-arachidonylglycerol (20:4)	Lipid	Monoacylglycerol	5282281	35474-99-8	HMDB11549
62558	1-carboxyethylisoleucine	Amino Acid	Leucine, Isoleucine and Valine Metabolism			
62559	1-carboxyethylleucine	Amino Acid	Leucine, Isoleucine and Valine Metabolism			
62566	1-carboxyethylphenylalanine	Amino Acid	Phenylalanine Metabolism			
62564	1-carboxyethyltyrosine	Amino Acid	Tyrosine Metabolism			
62562	1-carboxyethylvaline	Amino Acid	Leucine, Isoleucine and Valine Metabolism			
48341	1-dihomo-linolenylglycerol (20:3)	Lipid	Monoacylglycerol			
35103	1-dihomo-linoleoylglycerol (20:2)	Lipid	Monoacylglycerol			
35153	1-docosahexaenoylglycerol (22:6)	Lipid	Monoacylglycerol			HMDB11587
57783	1H-indole-7-acetic acid	Xenobiotics	Bacterial/Fungal		39689-63-9	
49617	1-lignoceroyl-GPC (24:0)	Lipid	Lysophospholipid		325171-59-3	HMDB10405
34393	1-linolenoylglycerol (18:3)	Lipid	Monoacylglycerol	53480978	124151-74-2	HMDB11569
45951	1-linolenoyl-GPC (18:3)*	Lipid	Lysophospholipid			HMDB10388
52710	1-linoleoyl-2-arachidonoyl-GPC (18:2/20:4n6)*	Lipid	Phosphatidylcholine (PC)			HMDB08147
53176	1-linoleoyl-2-linolenoyl-GPC (18:2/18:3)*	Lipid	Phosphatidylcholine (PC)			HMDB08141
27447	1-linoleoylglycerol (18:2)	Lipid	Monoacylglycerol	5283469	2277-28-3	
52690	1-linoleoyl-GPA (18:2)*	Lipid	Lysophospholipid			HMDB07856
34419	1-linoleoyl-GPC (18:2)	Lipid	Lysophospholipid	11988421		HMDB10386
36600	1-linoleoyl-GPE (18:2)*	Lipid	Lysophospholipid	52925130		HMDB11507
54885	1-linoleoyl-GPG (18:2)*	Lipid	Lysophospholipid			

36594	1-linoleoyl-GPI* (18:2)*	Lipid	Lysophospholipid			
32350	1-methyl-4-imidazoleacetate	Amino Acid	Histidine Metabolism	75810	2625-49-2	HMDB02820
62946	1-methyl-5-imidazoleacetate	Amino Acid	Histidine Metabolism	6451814	4200-48-0	HMDB04988
30460	1-methylhistidine	Amino Acid	Histidine Metabolism	92105	332-80-9	HMDB00001
27665	1-methylnicotinamide	Cofactors and Vitamins	Nicotinate and Nicotinamide Metabolism	10129985	1005-24-9	HMDB00699
34395	1-methylurate	Xenobiotics	Xanthine Metabolism	69726	708-79-2	HMDB03099
53195	1-myristoyl-2-arachidonoyl-GPC (14:0/20:4)*	Lipid	Phosphatidylcholine (PC)			HMDB07883
19258	1-myristoyl-2-palmitoyl-GPC (14:0/16:0)	Lipid	Phosphatidylcholine (PC)	129657	69525-80-0	HMDB07869
52697	1-oleoyl-2-docosahexaenoyl-GPC (18:1/22:6)*	Lipid	Phosphatidylcholine (PC)			HMDB08123
52687	1-oleoyl-2-linoleoyl-GPE (18:1/18:2)*	Lipid	Phosphatidylethanolamine (PE)	9546753		HMDB05349
21184	1-oleoylglycerol (18:1)	Lipid	Monoacylglycerol	5283468	111-03-5	HMDB11567
36812	1-oleoylglycerophosphate (18:1)	Lipid	Lysophospholipid	5497152	325465-93-8	
48258	1-oleoyl-GPC (18:1)	Lipid	Lysophospholipid	16081932	19420-56-5	HMDB02815
35628	1-oleoyl-GPE (18:1)	Lipid	Lysophospholipid	9547071	89576-29-4	HMDB11506
45968	1-oleoyl-GPG (18:1)*	Lipid	Lysophospholipid			
36602	1-oleoyl-GPI (18:1)*	Lipid	Lysophospholipid			
53180	1-palmitoleoyl-2-linolenoyl-GPC (16:1/18:3)*	Lipid	Phosphatidylcholine (PC)			HMDB08008
52431	1-palmitoleoylglycerol (16:1)*	Lipid	Monoacylglycerol			HMDB11565
33230	1-palmitoleoyl-GPC* (16:1)*	Lipid	Lysophospholipid	24779461		HMDB10383
52462	1-palmitoyl-2-arachidonoyl-GPC (16:0/20:4n6)	Lipid	Phosphatidylcholine (PC)	10747814	35418-58-7	HMDB07982
52464	1-palmitoyl-2-arachidonoyl-GPE (16:0/20:4)*	Lipid	Phosphatidylethanolamine (PE)	9546800		HMDB05323
52467	1-palmitoyl-2-arachidonoyl-GPI (16:0/20:4)*	Lipid	Phosphatidylinositol (PI)			HMDB09789
52454	1-palmitoyl-2-dihomo-linolenoyl-GPC (16:0/20:3n3 or 6)*	Lipid	Phosphatidylcholine (PC)			
52610	1-palmitoyl-2-docosahexaenoyl-GPC (16:0/22:6)	Lipid	Phosphatidylcholine (PC)	6441886	59403-54-2	HMDB07991
52465	1-palmitoyl-2-docosahexaenoyl-GPE (16:0/22:6)*	Lipid	Phosphatidylethanolamine (PE)	9546799		HMDB05324
42446	1-palmitoyl-2-linoleoyl-GPC (16:0/18:2)	Lipid	Phosphatidylcholine (PC)	5287971	40811-94-7	HMDB07973
42449	1-palmitoyl-2-linoleoyl-GPE (16:0/18:2)	Lipid	Phosphatidylethanolamine (PE)	9546747		HMDB05322
52450	1-palmitoyl-2-linoleoyl-GPI (16:0/18:2)	Lipid	Phosphatidylinositol (PI)			HMDB09784
52461	1-palmitoyl-2-oleoyl-GPC (16:0/18:1)	Lipid	Phosphatidylcholine (PC)	6436017	26853-31-6;26853-31-6	HMDB07972
19263	1-palmitoyl-2-oleoyl-GPE (16:0/18:1)	Lipid	Phosphatidylethanolamine (PE)	5283496	26662-94-2	HMDB05320
52669	1-palmitoyl-2-oleoyl-GPI (16:0/18:1)*	Lipid	Phosphatidylinositol (PI)			HMDB09783
52470	1-palmitoyl-2-palmitoleoyl-GPC (16:0/16:1)*	Lipid	Phosphatidylcholine (PC)			HMDB07969

52616	1-palmitoyl-2-stearoyl-GPC (16:0/18:0)	Lipid	Phosphatidylcholine (PC)		59403-51-9	HMDB07970
57388	1-palmitoyl-2-stearoyl-GPE (16:0/18:0)*	Lipid	Phosphatidylethanolamine (PE)	5326793		HMDB08925
33955	1-palmitoyl-GPC (16:0)	Lipid	Lysophospholipid	86554	17364-16-8	HMDB10382
35631	1-palmitoyl-GPE (16:0)	Lipid	Lysophospholipid	9547069	53862-35-4	HMDB11503
45970	1-palmitoyl-GPG (16:0)*	Lipid	Lysophospholipid	3300276		
35305	1-palmitoyl-GPI* (16:0)	Lipid	Lysophospholipid			HMDB61695
61868	1-ribosyl-imidazoleacetate*	Amino Acid	Histidine Metabolism	5117448	29605-99-0	HMDB02331
42450	1-stearoyl-2-arachidonoyl-GPC (18:0/20:4)	Lipid	Phosphatidylcholine (PC)	16219824	35418-59-8	HMDB08048
52447	1-stearoyl-2-arachidonoyl-GPE (18:0/20:4)	Lipid	Phosphatidylethanolamine (PE)	5289133		HMDB09003
52449	1-stearoyl-2-arachidonoyl-GPI (18:0/20:4)	Lipid	Phosphatidylinositol (PI)		383907-33-3	HMDB09815
52611	1-stearoyl-2-docosahexaenoyl-GPC (18:0/22:6)	Lipid	Phosphatidylcholine (PC)		59403-52-0	HMDB08057
52466	1-stearoyl-2-docosahexaenoyl-GPE (18:0/22:6)*	Lipid	Phosphatidylethanolamine (PE)	9546798		HMDB05334
52452	1-stearoyl-2-linoleoyl-GPC (18:0/18:2)*	Lipid	Phosphatidylcholine (PC)			HMDB08039
52446	1-stearoyl-2-linoleoyl-GPE (18:0/18:2)*	Lipid	Phosphatidylethanolamine (PE)	9546749		HMDB08994
52468	1-stearoyl-2-linoleoyl-GPI (18:0/18:2)	Lipid	Phosphatidylinositol (PI)			HMDB09809
52438	1-stearoyl-2-oleoyl-GPC (18:0/18:1)	Lipid	Phosphatidylcholine (PC)		56421-10-4	HMDB08038
42448	1-stearoyl-2-oleoyl-GPE (18:0/18:1)	Lipid	Phosphatidylethanolamine (PE)			HMDB08993
52726	1-stearoyl-2-oleoyl-GPI (18:0/18:1)*	Lipid	Phosphatidylinositol (PI)			
19265	1-stearoyl-2-oleoyl-GPS (18:0/18:1)	Lipid	Phosphatidylserine (PS)	9547087		HMDB10163
33961	1-stearoyl-GPC (18:0)	Lipid	Lysophospholipid	497299	19420-57-6	HMDB10384
42398	1-stearoyl-GPE (18:0)	Lipid	Lysophospholipid	9547068	69747-55-3	HMDB11130
34437	1-stearoyl-GPG (18:0)	Lipid	Lysophospholipid			
19324	1-stearoyl-GPI (18:0)	Lipid	Lysophospholipid		796963-93-4	HMDB61696
62885	2,2'-Methylenebis(6-tert-butyl-p-cresol)	Xenobiotics	Chemical	8398	119-47-1	
57547	2,3-dihydroxy-2-methylbutyrate	Amino Acid	Leucine, Isoleucine and Valine Metabolism	301941	14868-24-7	HMDB29576
62805	2,3-dihydroxy-5-methylthio-4-pentenoate (DMTPA)*	Amino Acid	Methionine, Cysteine, SAM and Taurine Metabolism			
38276	2,3-dihydroxyisovalerate	Xenobiotics	Food Component/Plant	677	1756-18-9	HMDB12141
46115	21-hydroxypregnenolone disulfate	Lipid	Pregnenolone Steroids	134595	1164-98-3	
6146	2-aminoadipate	Amino Acid	Lysine Metabolism	469	542-32-5;1118-90-7	HMDB00510
42374	2-aminobutyrate	Amino Acid	Glutathione Metabolism	439691	1492-24-6	HMDB00650
43761	2-aminoheptanoate	Lipid	Fatty Acid, Amino	227939	1115-90-8	

43343	2-aminooctanoate	Lipid	Fatty Acid, Amino	69522	644-90-6	HMDB00991
43266	2-aminophenol sulfate	Xenobiotics	Chemical	181670		HMDB61116
61785	2-butenoylglycine	Lipid	Fatty Acid Metabolism(Acyl Glycine)	6303498	71428-89-2	
52602	2'-deoxyuridine	Nucleotide	Pyrimidine Metabolism, Uracil containing	13712	951-78-0	HMDB00012
36847	2-ethylphenylsulfate	Xenobiotics	Benzoate Metabolism			
36746	2-hydroxy-3-methylvalerate	Amino Acid	Leucine, Isoleucine and Valine Metabolism	164623	488-15-3	HMDB00317
33173	2-hydroxyacetaminophen sulfate*	Xenobiotics	Drug - Analgesics, Anesthetics	86290013		
61700	2-hydroxyarachidate*	Lipid	Fatty Acid, Monohydroxy	5225199		
57663	2-hydroxybehenate	Lipid	Fatty Acid, Monohydroxy	193484		
52281	2-hydroxybutyrate/2-hydroxyisobutyrate	Amino Acid	Glutathione Metabolism			
42489	2-hydroxydecanoate	Lipid	Fatty Acid, Monohydroxy	21488	5393-81-7	
62528	2-hydroxyfluorene sulfate	Xenobiotics	Tobacco Metabolite			
37253	2-hydroxyglutarate	Lipid	Fatty Acid, Dicarboxylate	43	40951-21-1	HMDB00606
61827	2-hydroxyheptanoate*	Lipid	Fatty Acid, Monohydroxy	2750949		
18281	2-hydroxyhippurate (salicylurate)	Xenobiotics	Benzoate Metabolism	10253	487-54-7	HMDB00840
43330	2-hydroxyibuprofen	Xenobiotics	Drug - Analgesics, Anesthetics	10443535	51146-55-5	HMDB60920
52916	2-hydroxylaurate	Lipid	Fatty Acid, Monohydroxy	97783	2984-55-6	
61698	2-hydroxynervonate*	Lipid	Fatty Acid, Monohydroxy	5312783		
22036	2-hydroxyoctanoate	Lipid	Fatty Acid, Monohydroxy	94180	617-73-2	HMDB02264
35675	2-hydroxypalmitate	Lipid	Fatty Acid, Monohydroxy	92836	764-67-0	HMDB31057
1432	2-hydroxyphenylacetate	Amino Acid	Phenylalanine Metabolism	11970	614-75-5	HMDB00669
52922	2-hydroxyphenylacetate sulfate	Amino Acid	Phenylalanine Metabolism			
17945	2-hydroxystearate	Lipid	Fatty Acid, Monohydroxy	69417	629-22-1	
15667	2-isopropylmalate	Xenobiotics	Food Component/Plant	77	3237-44-3	HMDB00402
48141	2-keto-3-deoxy-gluconate	Xenobiotics	Food Component/Plant	161227	17510-99-5	HMDB01353
32506	2-linoleoylglycerol (18:2)	Lipid	Monoacylglycerol	5365676	3443-82-1	HMDB11538
33161	2-methoxyacetaminophen glucuronide*	Xenobiotics	Drug - Analgesics, Anesthetics	14367271		
47031	2-methoxyacetaminophen sulfate*	Xenobiotics	Drug - Analgesics, Anesthetics	86290014		
45095	2-methylbutyrylcarnitine (C5)	Amino Acid	Leucine, Isoleucine and Valine Metabolism	6426901	31023-25-3	HMDB00378
52282	2-methylcitrate/homocitrate	Energy	TCA Cycle			
35482	2-methylmalonylcarnitine (C4-DC)	Lipid	Fatty Acid Synthesis	53481628	149181-64-6	HMDB13133

62520	2-naphthol sulfate	Xenobiotics	Chemical	74428		
21232	2-oleoylglycerol (18:1)	Lipid	Monoacylglycerol	5319879	3443-84-3	HMDB11537
57554	2'-O-methylcytidine	Nucleotide	Pyrimidine Metabolism, Cytidine containing	150971	2140-72-9	
57655	2'-O-methyluridine	Nucleotide	Pyrimidine Metabolism, Uracil containing	102212	2140-76-3	
55072	2-oxoarginine*	Amino Acid	Urea cycle; Arginine and Proline Metabolism	558	04/10/3715	HMDB04225
52432	2-palmitoleoylglycerol (16:1)*	Lipid	Monoacylglycerol			HMDB11565
47118	2-palmitoleoyl-GPC* (16:1)*	Lipid	Lysophospholipid			HMDB10383
35253	2-palmitoyl-GPC* (16:0)*	Lipid	Lysophospholipid	15061532		HMDB61702
43400	2-piperidinone	Xenobiotics	Food Component/Plant	12665	675-20-7	HMDB11749
41220	2-stearoyl-GPE (18:0)*	Lipid	Lysophospholipid			HMDB11129
62924	3-(3-amino-3-carboxypropyl)uridine*	Nucleotide	Pyrimidine Metabolism, Uracil containing	171198	52745-94-5	
35635	3-(3-hydroxyphenyl)propionate	Xenobiotics	Benzoate Metabolism	91	621-54-5	HMDB00375
32197	3-(4-hydroxyphenyl)lactate (HPLA)	Amino Acid	Tyrosine Metabolism	9378	6482-98-0	HMDB00755
34365	3-(cystein-S-yl)acetaminophen*	Xenobiotics	Drug - Analgesics, Anesthetics	5233914		
62567	3-(methylthio)acetaminophen sulfate*	Xenobiotics	Drug - Analgesics, Anesthetics			
45721	3-(N-acetyl-L-cystein-S-yl) acetaminophen	Xenobiotics	Drug - Analgesics, Anesthetics	83967	52372-86-8	
53026	3,4-methyleneheptanoate	Xenobiotics	Food Component/Plant			
52929	3,4-methyleneheptanoylcarnitine	Lipid	Fatty Acid Metabolism(Acyl Carnitine)			
48693	3-acetylphenol sulfate	Xenobiotics	Chemical			
62853	3-amino-2-piperidone	Amino Acid	Urea cycle; Arginine and Proline Metabolism	5200225	1892-22-4	HMDB00323
1566	3-aminoisobutyrate	Nucleotide	Pyrimidine Metabolism, Thymine containing	64956	10569-72-9;214139-20-5	HMDB03911
36803	3beta,7alpha-dihydroxy-5-cholestenoate	Lipid	Sterol	3081084	115538-84-6	
54805	3beta-hydroxy-5-cholestenoate	Lipid	Sterol	165511	6561-58-6	
43507	3b-hydroxy-5-cholenoic acid	Lipid	Secondary Bile Acid Metabolism	92997	5255-17-4	HMDB00308
61871	3-carboxy-4-methyl-5-pentyl-2-furanpropionate (3-CMPFP)	Lipid	Fatty Acid, Dicarboxylate	194501		
31787	3-carboxy-4-methyl-5-propyl-2-furanpropanoate (CMPF)	Lipid	Fatty Acid, Dicarboxylate	123979	86879-39-2	HMDB61112
62796	3-ethylcatechol sulfate (1)	Xenobiotics	Food Component/Plant			
36848	3-ethylphenylsulfate	Xenobiotics	Benzoate Metabolism			HMDB62721
62863	3-formylindole	Xenobiotics	Food Component/Plant	10256		HMDB29737

32397	3-hydroxy-2-ethylpropionate	Amino Acid	Leucine, Isoleucine and Valine Metabolism	188979	4374-62-3	HMDB00396
531	3-hydroxy-3-methylglutarate	Lipid	Mevalonate Metabolism	1662	503-49-1	HMDB00355
542	3-hydroxybutyrate (BHBA)	Lipid	Ketone Bodies	441	625-72-9	HMDB00357
62064	3-hydroxybutyroylglycine	Lipid	Fatty Acid Metabolism(Acyl Glycine)			
43264	3-hydroxybutyrylcarnitine (1)	Lipid	Fatty Acid Metabolism(Acyl Carnitine)	53481617		HMDB13127
52984	3-hydroxybutyrylcarnitine (2)	Lipid	Fatty Acid Metabolism(Acyl Carnitine)			HMDB13127
22053	3-hydroxydecanoate	Lipid	Fatty Acid, Monohydroxy	26612	5561-87-5	HMDB02203
62915	3-hydroxydodecanedioate*	Lipid	Fatty Acid, Dicarboxylate	16663321	34574-69-1	HMDB00413
53230	3-hydroxyhexanoate	Lipid	Fatty Acid, Monohydroxy	151492	10191-24-9	
39600	3-hydroxyhippurate	Xenobiotics	Benzoate Metabolism	450268	1637-75-8	HMDB06116
1549	3-hydroxyisobutyrate	Amino Acid	Leucine, Isoleucine and Valine Metabolism	87	2068-83-9	HMDB00336
32457	3-hydroxylaurate	Lipid	Fatty Acid, Monohydroxy	94216	53941-38-1	HMDB00387
22001	3-hydroxyoctanoate	Lipid	Fatty Acid, Monohydroxy	26613	88930-08-9	HMDB01954
61840	3-hydroxyoleoylcarnitine	Lipid	Fatty Acid Metabolism(Acyl Carnitine)			
61839	3-hydroxypalmitoylcarnitine	Lipid	Fatty Acid Metabolism(Acyl Carnitine)			
48448	3-hydroxypyridine sulfate	Xenobiotics	Chemical		1955-23-3	
31943	3-hydroxysebacate	Lipid	Fatty Acid, Monohydroxy	3017884	73141-46-5	HMDB00350
62062	3-hydroxystachydrine*	Xenobiotics	Food Component/Plant	442640		
27672	3-indoxyl sulfate	Amino Acid	Tryptophan Metabolism	10258	2642-37-7	HMDB00682
48763	3-methoxycatechol sulfate (1)	Xenobiotics	Benzoate Metabolism			
48752	3-methoxycatechol sulfate (2)	Xenobiotics	Benzoate Metabolism			
44618	3-methoxytyramine sulfate	Amino Acid	Tyrosine Metabolism			
12017	3-methoxytyrosine	Amino Acid	Tyrosine Metabolism	1670	300-48-1	HMDB01434
46165	3-methyl catechol sulfate (1)	Xenobiotics	Benzoate Metabolism			
44526	3-methyl-2-oxobutyrate	Amino Acid	Leucine, Isoleucine and Valine Metabolism	49	3715-29-5	HMDB00019
15676	3-methyl-2-oxovalerate	Amino Acid	Leucine, Isoleucine and Valine Metabolism	47	1460-34-0;51829-07-3	HMDB03736
36749	3-methyladipate	Lipid	Fatty Acid, Dicarboxylate	12292	03/01/3058	HMDB00555
57747	3-methylglutaconate	Amino Acid	Leucine, Isoleucine and Valine Metabolism	1551553	5746-90-7	HMDB00522

46548	3-methylglutarylcarnitine (2)	Amino Acid	Leucine, Isoleucine and Valine Metabolism	128145		HMDB00552
15677	3-methylhistidine	Amino Acid	Histidine Metabolism	64969	368-16-1	HMDB00479
32445	3-methylxanthine	Xenobiotics	Xanthine Metabolism	70639	1076-22-8	HMDB01886
15749	3-phenylpropionate (hydrocinnamate)	Xenobiotics	Benzoate Metabolism	107	501-52-0	HMDB00764
1414	3-phosphoglycerate	Carbohydrate	Glycolysis, Gluconeogenesis, and Pyruvate Metabolism	724	80731-10-8	HMDB00807
3155	3-ureidopropionate	Nucleotide	Pyrimidine Metabolism, Uracil containing	111	462-88-4	HMDB00026
1558	4-acetamidobutanoate	Amino Acid	Polyamine Metabolism	18189	3025-96-5	HMDB03681
12032	4-acetamidophenol	Xenobiotics	Drug - Analgesics, Anesthetics	1983	103-90-2	HMDB01859
15736	4-acetamidophenylglucuronide	Xenobiotics	Drug - Analgesics, Anesthetics	83944	120595-80-4	HMDB10316
37475	4-acetaminophen sulfate	Xenobiotics	Drug - Analgesics, Anesthetics	83939	10066-90-7;32113-41-0	HMDB59911
44620	4-acetylphenyl sulfate	Xenobiotics	Benzoate Metabolism	4684006		
37181	4-allylphenol sulfate	Xenobiotics	Food Component/Plant			
38125	4-cholesten-3-one	Lipid	Sterol	91477	601-57-0	HMDB00921
36099	4-ethylphenyl sulfate	Xenobiotics	Benzoate Metabolism		123-07-9	HMDB62551
15681	4-guanidinobutanoate	Amino Acid	Guanidino and Acetamido Metabolism	500	463-003;463-00-3	HMDB03464
40062	4-hydroxy-2-oxoglutaric acid	Lipid	Fatty Acid, Dicarboxylate	599	1187-99-1	HMDB02070
48441	4-hydroxychlorothalonil	Xenobiotics	Chemical	34217	28343-61-5	
40499	4-hydroxyglutamate	Amino Acid	Glutamate Metabolism	439902	2485-33-8	HMDB01344
35527	4-hydroxyhippurate	Xenobiotics	Benzoate Metabolism	151012	2482-25-9	HMDB13678
541	4-hydroxyphenylacetate	Amino Acid	Phenylalanine Metabolism	127	156-38-7	HMDB00020
55017	4-hydroxyphenylacetylglutamine	Peptide	Acetylated Peptides			
1669	4-hydroxyphenylpyruvate	Amino Acid	Tyrosine Metabolism	979	156-39-8	HMDB00707
48457	4-methoxyphenol sulfate	Amino Acid	Tyrosine Metabolism		3233-60-1	
22116	4-methyl-2-oxopentanoate	Amino Acid	Leucine, Isoleucine and Valine Metabolism	70	816-66-0	HMDB00695
46146	4-methylcatechol sulfate	Xenobiotics	Benzoate Metabolism			
48418	4-methylguaiacol sulfate	Xenobiotics	Benzoate Metabolism		756760-43-7	
48442	4-vinylguaiacol sulfate	Xenobiotics	Food Component/Plant			
36098	4-vinylphenol sulfate	Xenobiotics	Benzoate Metabolism	6426766	2628-17-3	HMDB62775
43582	5-(galactosylhydroxy)-L-lysine	Amino Acid	Lysine Metabolism		32448-36-5	
1418	5,6-dihydrothymine	Nucleotide	Pyrimidine Metabolism, Thymine containing	93556	696-04-8	HMDB00079

1559	5,6-dihydrouracil	Nucleotide	Pyrimidine Metabolism, Uracil containing	649	504-07-4	HMDB00076
61833	5,6-dihydrouridine	Nucleotide	Pyrimidine Metabolism, Uracil containing	94312	04/05/5627	
34424	5-acetylamino-6-amino-3-methyluracil	Xenobiotics	Xanthine Metabolism	88299	1196153-01-1	HMDB04400
34401	5-acetylamino-6-formylamino-3-methyluracil	Xenobiotics	Xanthine Metabolism	108214	85438-96-6	HMDB11105
37184	5alpha-androstan-3alpha,17beta-diol disulfate	Lipid	Androgenic Steroids			
37186	5alpha-androstan-3alpha,17beta-diol monosulfate (1)	Lipid	Androgenic Steroids			
37185	5alpha-androstan-3alpha,17beta-diol monosulfate (2)	Lipid	Androgenic Steroids			
37187	5alpha-androstan-3beta,17alpha-diol disulfate	Lipid	Androgenic Steroids			
37190	5alpha-androstan-3beta,17beta-diol disulfate	Lipid	Androgenic Steroids	242332	571-20-0	HMDB00493
37192	5alpha-androstan-3beta,17beta-diol monosulfate (2)	Lipid	Androgenic Steroids			
37198	5alpha-pregnan-3beta,20alpha-diol disulfate	Lipid	Progestin Steroids			
37200	5alpha-pregnan-3beta,20alpha-diol monosulfate (2)	Lipid	Progestin Steroids			
37196	5alpha-pregnan-3beta,20beta-diol monosulfate (1)	Lipid	Progestin Steroids			
46172	5alpha-pregnan-diol disulfate	Lipid	Progestin Steroids	5127902		
33968	5-dodecenoate (12:1n7)	Lipid	Medium Chain Fatty Acid	5312378	2430-94-6	HMDB00529
61769	5-dodecenoylcarnitine (C12:1)	Lipid	Fatty Acid Metabolism(Acyl Carnitine)			HMDB13326
46297	5-HEPE	Lipid	Eicosanoid	6439678	83952-40-3	HMDB05081
37372	5-HETE	Lipid	Eicosanoid	5280733	73307-52-5	HMDB11134
31938	5-hydroxyhexanoate	Lipid	Fatty Acid, Monohydroxy	170748	44843-89-2	HMDB00525
15685	5-hydroxylysine	Amino Acid	Lysine Metabolism	1029	13204-98-3	HMDB00450
1419	5-methylthioadenosine (MTA)	Amino Acid	Polyamine Metabolism	439176	2457-80-9	HMDB01173
35136	5-methyluridine (ribothymidine)	Nucleotide	Pyrimidine Metabolism, Uracil containing	445408	1463-10-1	HMDB00884
1494	5-oxoproline	Amino Acid	Glutathione Metabolism	7405	98-79-3	HMDB00267
62503	6-bromotryptophan	Amino Acid	Tryptophan Metabolism			
48698	6-hydroxyindole sulfate	Xenobiotics	Chemical			
43231	6-oxopiperidine-2-carboxylate	Amino Acid	Lysine Metabolism	3014237	34622-39-4	HMDB61705
36776	7-HOCA	Lipid	Sterol	3081085	115538-85-7	HMDB12458
35114	7-methylguanine	Nucleotide	Purine Metabolism, Guanine containing	11361	578-76-7	HMDB00897
21239	8-hydroxyoctanoate	Lipid	Fatty Acid, Monohydroxy	69820	764-89-6	HMDB61914
38399	9,10-DiHOME	Lipid	Fatty Acid, Dihydroxy	9966640	263399-34-4	HMDB04704
47120	9-hydroxystearate	Lipid	Fatty Acid, Monohydroxy	9570127	25498-28-6	HMDB61661

48569	acesulfame	Xenobiotics	Food Component/Plant	36573	55589-62-3	HMDB33585
32198	acetylcarnitine (C2)	Lipid	Fatty Acid Metabolism(Acyl Carnitine)	1	5080-50-2	HMDB00201
43258	acisoga	Amino Acid	Polyamine Metabolism	129397	106692-36-8	
46173	aconitate [cis or trans]	Energy	TCA Cycle			
554	adenine	Nucleotide	Purine Metabolism, Adenine containing	190	73-24-5	HMDB00034
32342	AMP	Nucleotide	Purine Metabolism, Adenine containing	6083	149022-20-8	HMDB00045
52988	adipoylcarnitine (C6-DC)	Lipid	Fatty Acid Metabolism(Acyl Carnitine)	71296139		HMDB61677
32980	adrenate (22:4n6)	Lipid	Polyunsaturated Fatty Acid (n3 and n6)	5497181	2091-25-0	HMDB02226
57528	adrenoylcarnitine (C22:4)*	Lipid	Fatty Acid Metabolism(Acyl Carnitine)			
1126	alanine	Amino Acid	Alanine and Aspartate Metabolism	5950	56-41-7	HMDB00161
1107	allantoin	Nucleotide	Purine Metabolism, (Hypo)Xanthine/Inosine containing	204	97-59-6	HMDB00462
41494	alliin	Xenobiotics	Food Component/Plant	87310	556-27-4	HMDB33592
43534	allopurinol	Xenobiotics	Drug - Metabolic	2094	315-30-0	
38321	allopurinol riboside	Xenobiotics	Drug - Metabolic		16220-07-8	
37073	alpha-hydroxycaproate	Lipid	Fatty Acid, Monohydroxy	99824	6064-63-7	HMDB01624
22132	alpha-hydroxyisocaproate	Amino Acid	Leucine, Isoleucine and Valine Metabolism	83697	10303-64-7	HMDB00746
46537	alpha-hydroxyisovalerate	Amino Acid	Leucine, Isoleucine and Valine Metabolism	99823	600-37-3	HMDB00407
54738	alpha-hydroxymetoprolol	Xenobiotics	Drug - Cardiovascular	114962	56392-16-6	HMDB60994
4968	alpha-ketobutyrate	Amino Acid	Methionine, Cysteine, SAM and Taurine Metabolism	58	600-18-0	HMDB00005
62101	alpha-ketoglutaramate*	Amino Acid	Glutamate Metabolism		18465-19-5	
528	alpha-ketoglutarate	Energy	TCA Cycle	51	305-72-6;328-50-7;22202-68-2	HMDB00208
1561	alpha-tocopherol	Cofactors and Vitamins	Tocopherol Metabolism	14985	59-02-9;10191-41-0	HMDB01893
52948	amoxicillin	Xenobiotics	Drug - Antibiotic	2171	26787-78-0	HMDB15193
32827	andro steroid monosulfate C19H28O6S (1)*	Lipid	Androgenic Steroids			HMDB02759
37207	androstenediol (3alpha, 17alpha) monosulfate (2)	Lipid	Androgenic Steroids			
37209	androstenediol (3alpha, 17alpha) monosulfate (3)	Lipid	Androgenic Steroids			
37202	androstenediol (3beta,17beta) disulfate (1)	Lipid	Androgenic Steroids	87120982		HMDB03818

37203	androstenediol (3beta,17beta) disulfate (2)	Lipid	Androgenic Steroids	87120982		HMDB03818
37211	androstenediol (3beta,17beta) monosulfate (1)	Lipid	Androgenic Steroids	13847309	521-17-5	HMDB03818
37210	androstenediol (3beta,17beta) monosulfate (2)	Lipid	Androgenic Steroids			
61846	androsterone glucuronide	Lipid	Androgenic Steroids	114833	1852-43-3	HMDB02829
31591	androsterone sulfate	Lipid	Androgenic Steroids	159663	2479-86-9	HMDB02759
575	arabinose	Carbohydrate	Pentose Metabolism	66308	28697-53-2	HMDB00646
48885	arabitol/xylitol	Carbohydrate	Pentose Metabolism	6912		
48255	arabonate/xylonate	Carbohydrate	Pentose Metabolism			
1118	arachidate (20:0)	Lipid	Long Chain Fatty Acid	10467	506-30-9	HMDB02212
1110	arachidonate (20:4n6)	Lipid	Polyunsaturated Fatty Acid (n3 and n6)	444899	506-32-1	HMDB01043
57518	arachidonoylcarnitine (C20:4)	Lipid	Fatty Acid Metabolism(Acyl Carnitine)			
53261	arachidonoylcholine	Lipid	Fatty Acid Metabolism (Acyl Choline)			
57513	arachidoylcarnitine (C20)*	Lipid	Fatty Acid Metabolism(Acyl Carnitine)			HMDB06460
57461	argininate*	Amino Acid	Urea cycle; Arginine and Proline Metabolism	160437	157-07-3	HMDB03148
1638	arginine	Amino Acid	Urea cycle; Arginine and Proline Metabolism	232	1119-34-2	HMDB00517
512	asparagine	Amino Acid	Alanine and Aspartate Metabolism	6267	70-47-3	HMDB00168
443	aspartate	Amino Acid	Alanine and Aspartate Metabolism	5960	56-84-8	HMDB00191
18362	azelate (nonanedioate; C9)	Lipid	Fatty Acid, Dicarboxylate	2266	123-99-9	HMDB00784
57331	behenoyl dihydrosphingomyelin (d18:0/22:0)*	Lipid	Dihydrosphingomyelins			HMDB12091
48492	behenoyl sphingomyelin (d18:1/22:0)*	Lipid	Sphingomyelins			HMDB12103
57514	behenoylcarnitine (C22)*	Lipid	Fatty Acid Metabolism(Acyl Carnitine)			
15778	benzoate	Xenobiotics	Benzoate Metabolism	243	65-85-0	HMDB01870
55	beta-alanine	Nucleotide	Pyrimidine Metabolism, Uracil containing	239	56-41-7;107-95-9	HMDB00056
54923	beta-citrylglutamate	Amino Acid	Glutamate Metabolism	72715786	73590-26-8	
57591	beta-cryptoxanthin	Cofactors and Vitamins	Vitamin A Metabolism	6384256	472-70-8	HMDB33844
12129	beta-hydroxyisovalerate	Amino Acid	Leucine, Isoleucine and Valine Metabolism	69362	625-08-1	HMDB00754
3141	betaine	Amino Acid	Glycine, Serine and Threonine Metabolism	247	107-43-7	HMDB00043
27414	beta-sitosterol	Lipid	Sterol	222284	83-46-5	HMDB00852

32586	bilirubin (E,E)*	Cofactors and Vitamins	Hemoglobin and Porphyrin Metabolism	5315454	114-25-0	
47886	bilirubin (E,Z or Z,E)*	Cofactors and Vitamins	Hemoglobin and Porphyrin Metabolism	5799469		HMDB00488
43807	bilirubin	Cofactors and Vitamins	Hemoglobin and Porphyrin Metabolism	5280352	635-65-4	HMDB00054
2137	biliverdin	Cofactors and Vitamins	Hemoglobin and Porphyrin Metabolism	5353439	55482-27-4;55482-27-4	HMDB01008
22154	bradykinin	Peptide	Polypeptide	439201	58-82-2	HMDB04246
34420	bradykinin, des-arg(9)	Peptide	Polypeptide	105044	15958-92-6	HMDB04246
32412	butyrylcarnitine (C4)	Lipid	Fatty Acid Metabolism (also BCAA Metabolism)	439829	25576-40-3	HMDB02013
53254	caffeic acid sulfate	Xenobiotics	Food Component/Plant			HMDB41708
569	caffeine	Xenobiotics	Xanthine Metabolism	2519	1958/8/2	HMDB01847
33997	campesterol	Lipid	Sterol	173183	474-62-4	HMDB02869
1642	caprate (10:0)	Lipid	Medium Chain Fatty Acid	2969	334-48-5	HMDB00511
32489	caproate (6:0)	Lipid	Medium Chain Fatty Acid	8892	142-62-1	HMDB00535
32492	caprylate (8:0)	Lipid	Medium Chain Fatty Acid	379	124-07-2	HMDB00482
40007	carboxyethyl-GABA	Amino Acid	Glutamate Metabolism	2572	02/03/4386	HMDB02201
43333	carboxyibuprofen	Xenobiotics	Drug - Analgesics, Anesthetics	10444113	15935-54-3	HMDB60564
62067	carboxyibuprofen glucuronide*	Xenobiotics	Drug - Analgesics, Anesthetics			HMDB60564
15500	carnitine	Lipid	Carnitine Metabolism	10917	461-05-2	HMDB00062
57635	carotene diol (1)	Cofactors and Vitamins	Vitamin A Metabolism			
57636	carotene diol (2)	Cofactors and Vitamins	Vitamin A Metabolism			
57637	carotene diol (3)	Cofactors and Vitamins	Vitamin A Metabolism			
35320	catechol sulfate	Xenobiotics	Benzoate Metabolism	3083879	4918-96-1	HMDB59724
57684	cefoperazone	Xenobiotics	Drug - Antibiotic	7048632	62893-20-3	
57437	ceramide (d16:1/24:1, d18:1/22:1)*	Lipid	Ceramides			
57434	ceramide (d18:1/17:0, d17:1/18:0)*	Lipid	Ceramides			
57440	ceramide (d18:1/20:0, d16:1/22:0, d20:1/18:0)*	Lipid	Ceramides			
57443	ceramide (d18:2/24:1, d18:1/24:2)*	Lipid	Ceramides			
57516	cerotoylcarnitine (C26)*	Lipid	Fatty Acid Metabolism(Acyl Carnitine)			HMDB06347
54745	cetirizine	Xenobiotics	Drug - Respiratory	2678	83881-51-0	HMDB05032
48782	C-glycosyltryptophan	Amino Acid	Tryptophan Metabolism	10981970	180509-18-6	
1563	chenodeoxycholate	Lipid	Primary Bile Acid Metabolism	10133	474-24-9;474-25-9	HMDB00518

22842	cholate	Lipid	Primary Bile Acid Metabolism	221493	81-25-4	HMDB00619
63	cholesterol	Lipid	Sterol	11025495	57-88-5	HMDB00067
15506	choline	Lipid	Phospholipid Metabolism	305	67-48-1	HMDB00097
34396	phosphocholine	Lipid	Phospholipid Metabolism	1014	72556-74-2	HMDB01565
42592	cimetidine	Xenobiotics	Drug - Gastrointestinal		51481-61-9	
38637	cinnamoylglycine	Xenobiotics	Food Component/Plant	709625	16534-24-0	HMDB11621
62873	cis-4-decenoate (10:1n6)*	Lipid	Medium Chain Fatty Acid	5312351	505-90-8	
38178	cis-4-decenoylcarnitine (C10:1)	Lipid	Fatty Acid Metabolism(Acyl Carnitine)		98930-66-6	
22158	citramalate	Amino Acid	Glutamate Metabolism	1081	08/10/6236	HMDB00426
1564	citrate	Energy	TCA Cycle	311	77-92-9	HMDB00094
2132	citrulline	Amino Acid	Urea cycle; Arginine and Proline Metabolism	9750	372-75-8	HMDB00904
57673	clindamycin	Xenobiotics	Drug - Antibiotic	446598	18323-44-9	
5983	corticosterone	Lipid	Corticosteroids	5753	50-22-6	HMDB01547
1712	cortisol	Lipid	Corticosteroids	5754	50-23-7	HMDB00063
1769	cortisone	Lipid	Corticosteroids	222786	1953/6/5	HMDB02802
553	cotinine	Xenobiotics	Tobacco Metabolite	854019	486-56-6	HMDB01046
27718	creatine	Amino Acid	Creatine Metabolism	586	57-00-1	HMDB00064
513	creatinine	Amino Acid	Creatine Metabolism	588	60-27-5	HMDB00562
18368	cys-gly, oxidized	Amino Acid	Glutathione Metabolism	333293	7729-20-6	
15705	cystathionine	Amino Acid	Methionine, Cysteine, SAM and Taurine Metabolism	439258	535-34-2	HMDB00099
1868	cysteine	Amino Acid	Methionine, Cysteine, SAM and Taurine Metabolism	5862	52-90-4;56-89-3	HMDB00574
22176	cysteine s-sulfate	Amino Acid	Methionine, Cysteine, SAM and Taurine Metabolism	115015	1637-71-4	HMDB00731
37443	cysteine sulfinic acid	Amino Acid	Methionine, Cysteine, SAM and Taurine Metabolism	109	207121-48-0	HMDB00996
35159	cysteine-glutathione disulfide	Amino Acid	Glutathione Metabolism	4247235	13081-14-6	HMDB00656
35637	cysteinylglycine	Amino Acid	Glutathione Metabolism	439498	19246-18-5	HMDB00078
62103	cysteinylglycine disulfide*	Amino Acid	Glutathione Metabolism		70555-24-7	HMDB00709
56	cystine	Amino Acid	Methionine, Cysteine, SAM and Taurine Metabolism	67678	56-89-3	HMDB00192

514	cytidine	Nucleotide	Pyrimidine Metabolism, Cytidine containing	6175	65-46-3	HMDB00089
48728	daidzein sulfate (2)	Xenobiotics	Food Component/Plant			
33941	decanoylcarnitine (C10)	Lipid	Fatty Acid Metabolism(Acyl Carnitine)	10245190	1492-27-9	HMDB00651
32425	dehydroisoandrosterone sulfate (DHEA-S)	Lipid	Androgenic Steroids	12594	651-48-9	HMDB01032
62286	delta-CEHC glucuronide*	Cofactors and Vitamins	Tocopherol Metabolism			
62287	delta-CEHC*	Cofactors and Vitamins	Tocopherol Metabolism	11536125	84599-16-6	
33418	delta-tocopherol	Cofactors and Vitamins	Tocopherol Metabolism	92094	119-13-1	HMDB02902
36747	deoxycarnitine	Lipid	Carnitine Metabolism	134	6249-56-5	HMDB01161
1114	deoxycholate	Lipid	Secondary Bile Acid Metabolism	222528	83-44-3	HMDB00626
46331	desmethylnaproxen	Xenobiotics	Drug - Analgesics, Anesthetics	13393711	52079-10-4	HMDB13989
46106	desmethylnaproxen sulfate	Xenobiotics	Drug - Analgesics, Anesthetics	184679		
57668	dexlansoprazole	Xenobiotics	Drug - Gastrointestinal	9578005	138530-94-6	
54953	diacylglycerol (14:0/18:1, 16:0/16:1) [1]*	Lipid	Diacylglycerol			
54954	diacylglycerol (14:0/18:1, 16:0/16:1) [2]*	Lipid	Diacylglycerol			
54966	diacylglycerol (16:1/18:2 [2], 16:0/18:3 [1])*	Lipid	Diacylglycerol			
52931	diclofenac	Xenobiotics	Drug - Analgesics, Anesthetics		15307-86-5	HMDB14724
17805	dihomolinoleate (20:2n6)	Lipid	Polyunsaturated Fatty Acid (n3 and n6)	6439848	2091-39-6	HMDB05060
35718	dihomolinolenate (20:3n3 or 3n6)	Lipid	Polyunsaturated Fatty Acid (n3 and n6)	5280581	17046-59-2	HMDB02925
57521	dihomo-linolenoylcarnitine (C20:3n3 or 6)*	Lipid	Fatty Acid Metabolism(Acyl Carnitine)			
53262	dihomo-linolenoyl-choline	Lipid	Fatty Acid Metabolism (Acyl Choline)			
57520	dihomo-linoleoylcarnitine (C20:2)*	Lipid	Fatty Acid Metabolism(Acyl Carnitine)			
62524	dihydrocaffeate sulfate (2)	Xenobiotics	Food Component/Plant			
40481	dihydroferulate	Xenobiotics	Food Component/Plant	14340	1135-23-5	
62857	dihydroferulic acid sulfate	Xenobiotics	Food Component/Plant	187489		HMDB41724
601	dihydroorotate	Nucleotide	Pyrimidine Metabolism, Orotate containing	648	155-54-4	HMDB03349
36808	dimethylarginine (ADMA + SDMA)	Amino Acid	Urea cycle; Arginine and Proline Metabolism	123831	102783-24-4	HMDB01539
5086	dimethylglycine	Amino Acid	Glycine, Serine and Threonine Metabolism	673	1118-68-9	HMDB00092

32415	docosadienoate (22:2n6)	Lipid	Polyunsaturated Fatty Acid (n3 and n6)	5282807	7370-49-2	HMDB61714
39837	docosadioate (C22-DC)	Lipid	Fatty Acid, Dicarboxylate	244872	505-56-6	
44675	docosahexaenoate (DHA; 22:6n3)	Lipid	Polyunsaturated Fatty Acid (n3 and n6)	445580	6217-54-5	HMDB02183
57523	docosahexaenoylcarnitine (C22:6)*	Lipid	Fatty Acid Metabolism(Acyl Carnitine)			
53263	docosahexaenoylcholine	Lipid	Fatty Acid Metabolism (Acyl Choline)			
32504	docosapentaenoate (DPA; 22:5n3)	Lipid	Polyunsaturated Fatty Acid (n3 and n6)	6441454	2234-74-4	HMDB06528
37478	docosapentaenoate (n6 DPA; 22:5n6)	Lipid	Polyunsaturated Fatty Acid (n3 and n6)	6441454	25182-74-5	HMDB01976
57529	docosapentaenoylcarnitine (C22:5n3)*	Lipid	Fatty Acid Metabolism(Acyl Carnitine)			
32417	docosatrienoate (22:3n3)	Lipid	Polyunsaturated Fatty Acid (n3 and n6)	5312556	59708-86-0	HMDB02823
57467	docosatrienoate (22:3n6)*	Lipid	Polyunsaturated Fatty Acid (n3 and n6)			
62276	dodecadienoate (12:2)*	Lipid	Fatty Acid, Dicarboxylate			
32388	dodecanedioate (C12)	Lipid	Fatty Acid, Dicarboxylate	12736	693-23-2	HMDB00623
61864	dodecenedioate (C12:1-DC)*	Lipid	Fatty Acid, Dicarboxylate			
48407	dopamine 3-O-sulfate	Amino Acid	Tyrosine Metabolism	122136		HMDB06275
39273	doxycycline	Xenobiotics	Drug - Antibiotic		24390-14-5	
35651	ectoine	Xenobiotics	Chemical	126041	96702-03-3	
22163	EDTA	Xenobiotics	Chemical	6049	60-00-4	HMDB15109
39831	eicosanedioate (C20-DC)	Lipid	Fatty Acid, Dicarboxylate	75502	2424-92-2	
18467	eicosapentaenoate (EPA; 20:5n3)	Lipid	Polyunsaturated Fatty Acid (n3 and n6)	446284	10-2005-9;10417-94-4	HMDB01999
62445	eicosenamide (20:1)*	Lipid	Fatty Acid, Amide	5365374		
62923	eicosenedioate (C20:1-DC)*	Lipid	Fatty Acid, Dicarboxylate			
33587	eicosenoate (20:1n9 or 1n11)	Lipid	Long Chain Fatty Acid	5282768		HMDB02231
57519	eicosenoylcarnitine (C20:1)*	Lipid	Fatty Acid Metabolism(Acyl Carnitine)			
33973	epiandrosterone sulfate	Lipid	Androgenic Steroids	9929317	22229-22-7	
40478	equol sulfate	Xenobiotics	Food Component/Plant			
37459	ergothioneine	Xenobiotics	Food Component/Plant	3032311	58511-63-0	HMDB03045
1552	erucate (22:1n9)	Lipid	Long Chain Fatty Acid	5281116	112-86-7	HMDB02068
57525	erucoylcarnitine (C22:1)*	Lipid	Fatty Acid Metabolism(Acyl			

			Carnitine)			
20699	erythritol	Xenobiotics	Food Component/Plant	222285	149-32-6	HMDB02994
42420	erythronate*	Carbohydrate	Aminosugar Metabolism	2781043	88759-55-1	HMDB00613
39603	ethyl glucuronide	Xenobiotics	Chemical	152226	17685-04-0	HMDB10325
15765	ethylmalonate	Amino Acid	Leucine, Isoleucine and Valine Metabolism	11756	601-75-2	HMDB00622
47112	etiocholanolone glucuronide	Lipid	Androgenic Steroids	270605	03/09/3602	HMDB04484
48715	eugenol sulfate	Xenobiotics	Food Component/Plant	180632	95480-60-7	
47114	ferulic acid 4-sulfate	Xenobiotics	Food Component/Plant	6305574	86321-29-1	HMDB29200
31548	DSGEGDFXAEGGGVR*	Peptide	Fibrinogen Cleavage Peptide			
57712	ADpSGEGDFXAEGGGVR*	Peptide	Fibrinogen Cleavage Peptide		886023-52-5	
61837	Fibrinopeptide B	Peptide	Fibrinogen Cleavage Peptide		36204-23-6	
62813	Fibrinopeptide B (1-13)	Peptide	Fibrinogen Cleavage Peptide			
2134	FAD	Cofactors and Vitamins	Riboflavin Metabolism	643975	146-14-5;84366-81-4	HMDB01248
43493	formiminoglutamate	Amino Acid	Histidine Metabolism	439233	816-90-0	HMDB00854
48195	fructose	Carbohydrate	Fructose, Mannose and Galactose Metabolism	5984	57-48-7	HMDB00660
1643	fumarate	Energy	TCA Cycle	444972	100-17-8	HMDB00134
27719	galactonate	Carbohydrate	Fructose, Mannose and Galactose Metabolism	128869	299-28-5	HMDB00565
38754	gamma-carboxyglutamate	Amino Acid	Glutamate Metabolism	40772	56271-99-9	HMDB41900
44876	gamma-CEHC	Cofactors and Vitamins	Tocopherol Metabolism	133098	178167-75-4	HMDB01931
42381	gamma-CEHC glucuronide*	Cofactors and Vitamins	Tocopherol Metabolism			
37092	gamma-glutamyl-2-aminobutyrate	Peptide	Gamma-glutamyl Amino Acid		16869-42-4	
37063	gamma-glutamylalanine	Peptide	Gamma-glutamyl Amino Acid	440103	5875-41-2	HMDB29142
55015	gamma-glutamyl-alpha-lysine	Peptide	Gamma-glutamyl Amino Acid	65254		
62104	gamma-glutamylcitrulline*	Peptide	Gamma-glutamyl Amino Acid			
33934	gamma-glutamyl-epsilon-lysine	Peptide	Gamma-glutamyl Amino Acid	7015685	17105-15-6	HMDB03869
36738	gamma-glutamylglutamate	Peptide	Gamma-glutamyl Amino Acid	92865	1116-22-9	HMDB11737
2730	gamma-glutamylglutamine	Peptide	Gamma-glutamyl Amino Acid	150914	10148-81-9	HMDB11738
33949	gamma-glutamylglycine	Peptide	Gamma-glutamyl Amino Acid	165527	1948-29-4	HMDB11667
18245	gamma-glutamylhistidine	Peptide	Gamma-glutamyl Amino Acid	7017195	37460-15-4	

34456	gamma-glutamylisoleucine*	Peptide	Gamma-glutamyl Amino Acid	14253342		HMDB11170
18369	gamma-glutamylleucine	Peptide	Gamma-glutamyl Amino Acid	151023	2566-39-4	HMDB11171
44872	gamma-glutamylmethionine	Peptide	Gamma-glutamyl Amino Acid	7009567	17663-87-5	HMDB29155
33422	gamma-glutamylphenylalanine	Peptide	Gamma-glutamyl Amino Acid	111299	7432-24-8	HMDB00594
33364	gamma-glutamylthreonine	Peptide	Gamma-glutamyl Amino Acid	76078708	5652-48-2	HMDB29159
33947	gamma-glutamyltryptophan	Peptide	Gamma-glutamyl Amino Acid	3989307	66471-20-3	HMDB29160
2734	gamma-glutamyltyrosine	Peptide	Gamma-glutamyl Amino Acid	94340	7432-23-7	HMDB11741
43829	gamma-glutamylvaline	Peptide	Gamma-glutamyl Amino Acid	7015683	2746-34-1	HMDB11172
52473	gamma-tocopherol/beta-tocopherol	Cofactors and Vitamins	Tocopherol Metabolism			
62061	genistein sulfate*	Xenobiotics	Food Component/Plant			
18280	gentisate	Amino Acid	Tyrosine Metabolism	3469	490-79-9	HMDB00152
587	gluconate	Xenobiotics	Food Component/Plant	10690	527-07-1	HMDB00625
48152	glucose	Carbohydrate	Glycolysis, Gluconeogenesis, and Pyruvate Metabolism	79025	50-99-7	HMDB00122
15443	glucuronate	Carbohydrate	Aminosugar Metabolism	444791	207300-70-7	HMDB00127
61887	glucuronide of C10H18O2 (7)*	Partially Characterized Molecules	Partially Characterized Molecules			
57	glutamate	Amino Acid	Glutamate Metabolism	611	56-86-0	HMDB00148
53	glutamine	Amino Acid	Glutamate Metabolism	5961	56-85-9	HMDB00641
396	glutarate (C5-DC)	Lipid	Fatty Acid, Dicarboxylate	743	110-94-1	HMDB00661
44664	glutaroylcarnitine (C5)	Amino Acid	Lysine Metabolism	71464488	102636-82-8	HMDB13130
1572	glycerate	Carbohydrate	Glycolysis, Gluconeogenesis, and Pyruvate Metabolism	752	600-19-1	HMDB00139
15122	glycerol	Lipid	Glycerolipid Metabolism	753	56-81-5	HMDB00131
43847	glycerol 3-phosphate	Lipid	Glycerolipid Metabolism	754	29849-82-9	HMDB00126
37455	glycerophosphoethanolamine	Lipid	Phospholipid Metabolism	123874	33049-08-0	HMDB00114
48857	glycerophosphoglycerol	Lipid	Glycerolipid Metabolism	439964		
15990	glycerophosphorylcholine (GPC)	Lipid	Phospholipid Metabolism	71920	28319-77-9	HMDB00086
58	glycine	Amino Acid	Glycine, Serine and Threonine Metabolism	750	56-40-6	HMDB00123
62146	glycine conjugate of C10H14O2 (1)*	Partially Characterized Molecules	Partially Characterized Molecules			
62065	glyco-alpha-muricholate	Lipid	Primary Bile Acid Metabolism			

62066	glyco-beta-muricholate	Lipid	Primary Bile Acid Metabolism			
32346	glycochenodeoxycholate	Lipid	Primary Bile Acid Metabolism	12544	16564-43-5	HMDB00637
52974	glycochenodeoxycholate 3-sulfate	Lipid	Primary Bile Acid Metabolism			
52983	glycochenodeoxycholate glucuronide (1)	Lipid	Primary Bile Acid Metabolism			
18476	glycocholate	Lipid	Primary Bile Acid Metabolism	10140	475-31-0;863-57-0	HMDB00138
32599	glycocholenate sulfate*	Lipid	Secondary Bile Acid Metabolism			
18477	glycodeoxycholate	Lipid	Secondary Bile Acid Metabolism	3035026	360-65-6	HMDB00631
52975	glycodeoxycholate 3-sulfate	Lipid	Secondary Bile Acid Metabolism			
42574	glycohyocholate	Lipid	Secondary Bile Acid Metabolism			
31912	glycolithocholate	Lipid	Secondary Bile Acid Metabolism	115245	474-74-8	HMDB00698
32620	glycolithocholate sulfate*	Lipid	Secondary Bile Acid Metabolism	72222	15324-64-8	HMDB02639
57595	glycosyl ceramide (d18:1/20:0, d16:1/22:0)*	Lipid	Hexosylceramides (HCER)			
57448	glycosyl ceramide (d18:1/23:1, d17:1/24:1)*	Lipid	Hexosylceramides (HCER)			
57453	glycosyl ceramide (d18:2/24:1, d18:1/24:2)*	Lipid	Hexosylceramides (HCER)			
57444	glycosyl-N-(2-hydroxynervonoyl)-sphingosine (d18:1/24:1(2OH))*	Lipid	Hexosylceramides (HCER)			
57421	glycosyl-N-behenoyl-sphingadienine (d18:2/22:0)*	Lipid	Hexosylceramides (HCER)			
57371	glycosyl-N-behenoyl-sphingosine (d18:1/22:0)*	Lipid	Hexosylceramides (HCER)			
57369	glycosyl-N-nervonoyl-sphingosine (d18:1/24:1)*	Lipid	Hexosylceramides (HCER)			
53013	glycosyl-N-palmitoyl-sphingosine (d18:1/16:0)	Lipid	Hexosylceramides (HCER)			
52234	glycosyl-N-stearoyl-sphingosine (d18:1/18:0)	Lipid	Hexosylceramides (HCER)			
57445	glycosyl-N-tricosanoyl-sphingadienine (d18:2/23:0)*	Lipid	Hexosylceramides (HCER)			
39379	glycoursodeoxycholate	Lipid	Secondary Bile Acid Metabolism	12310288	64480-66-6	HMDB00708
62518	glycoursodeoxycholate glucuronide (2)	Lipid	Secondary Bile Acid Metabolism			
39789	glycyrrhetinate	Xenobiotics	Food Component/Plant	18526330	471-53-4	HMDB11628
46111	guaiacol sulfate	Xenobiotics	Benzoate Metabolism	22473		HMDB60013
43802	guanidinoacetate	Amino Acid	Creatine Metabolism	763	352-97-6	HMDB00128
32446	guanidinosuccinate	Amino Acid	Guanidino and Acetamido	97856	6133-30-8	HMDB03157

			Metabolism			
1573	guanosine	Nucleotide	Purine Metabolism, Guanine containing	6802	118-00-3	HMDB00133
46957	gulonate*	Cofactors and Vitamins	Ascorbate and Aldarate Metabolism	9794176	20246-53-1	HMDB03290
41754	heme	Cofactors and Vitamins	Hemoglobin and Porphyrin Metabolism	26945	14875-96-8	HMDB03178
57658	heneicosapentaenoate (21:5n3)	Lipid	Polyunsaturated Fatty Acid (n3 and n6)	11998573	24257-10-1	
62443	heptadecenamide (17:1)*	Lipid	Fatty Acid, Amide			
61866	heptenedioate (C7:1-DC)*	Lipid	Fatty Acid, Dicarboxylate			
57652	hexadecadienoate (16:2n6)	Lipid	Polyunsaturated Fatty Acid (n3 and n6)		28290-73-5	HMDB00477
35678	hexadecanedioate (C16)	Lipid	Fatty Acid, Dicarboxylate	10459	505-54-4	HMDB00672
57428	hexadecasphingosine (d16:1)*	Lipid	Sphingosines			
61862	hexadecenedioate (C16:1-DC)*	Lipid	Fatty Acid, Dicarboxylate			
32328	hexanoylcarnitine (C6)	Lipid	Fatty Acid Metabolism(Acyl Carnitine)	6426853	6920-35-0	HMDB00705
54907	hexanoylglutamine	Lipid	Fatty Acid Metabolism (Acyl Glutamine)			
35436	hexanoylglycine (C6)	Lipid	Fatty Acid Metabolism(Acyl Glycine)	99463	24003-67-6	HMDB00701
15753	hippurate	Xenobiotics	Benzoate Metabolism	464	495-69-2	HMDB00714
59	histidine	Amino Acid	Histidine Metabolism	6274	5934-29-2	HMDB00177
62278	histidine betaine (hercynine)*	Xenobiotics	Food Component/Plant	3083620	534-30-5	
22137	homoarginine	Amino Acid	Urea cycle; Arginine and Proline Metabolism	9085	156-86-5	HMDB00670
22138	homocitrulline	Amino Acid	Urea cycle; Arginine and Proline Metabolism	65072	1190-49-4	HMDB00679
33009	homostachydrine*	Xenobiotics	Food Component/Plant	441447	1195-94-4	HMDB33433
1101	homovanillate (HVA)	Amino Acid	Tyrosine Metabolism	1738	306-08-1	HMDB00118
57711	HWESASXX*	Peptide	Polypeptide			
40473	hydantoin-5-propionate	Amino Acid	Histidine Metabolism	782	5624-26-0	HMDB01212
39625	hydrochlorothiazide	Xenobiotics	Drug - Cardiovascular	3639	58-93-5	HMDB01928
35322	hydroquinone sulfate	Xenobiotics	Drug - Topical Agents	161220	123-31-9	HMDB02434
62060	hydroxyasparagine	Amino Acid	Alanine and Aspartate Metabolism	97663		HMDB32332
62277	hydroxy-CMPF*	Lipid	Fatty Acid, Dicarboxylate			
38661	hydroxycotinine	Xenobiotics	Tobacco Metabolite	10219774	34834-67-8	HMDB01390
62959	hydroxy-N6,N6,N6-trimethyllysine*	Amino Acid	Lysine Metabolism			

62851	hydroxypalmitoyl sphingomyelin (d18:1/16:0(OH))	Lipid	Sphingomyelins			
34093	hyocholate	Lipid	Secondary Bile Acid Metabolism	92805	547-75-1	HMDB00760
590	hypotaurine	Amino Acid	Methionine, Cysteine, SAM and Taurine Metabolism	107812	300-84-5	HMDB00965
3127	hypoxanthine	Nucleotide	Purine Metabolism, (Hypo)Xanthine/Inosine containing	790	68-94-0	HMDB00157
17799	ibuprofen	Xenobiotics	Drug - Analgesics, Anesthetics	3672	15687-27-1	HMDB01925
38366	ibuprofen acyl glucuronide	Xenobiotics	Drug - Analgesics, Anesthetics	163959	115075-59-7	
15716	imidazole lactate	Amino Acid	Histidine Metabolism	440129	14403-45-3	HMDB02320
40730	imidazole propionate	Amino Acid	Histidine Metabolism	70630	1074-59-5	HMDB02271
21025	iminodiacetate (IDA)	Xenobiotics	Chemical	8897	142-73-4	HMDB11753
38116	indole-3-carboxylate	Amino Acid	Tryptophan Metabolism	69867	771-50-6	HMDB03320
27513	indoleacetate	Amino Acid	Tryptophan Metabolism	802	6505-45-9	HMDB00197
62279	indoleacetylcarnitine*	Xenobiotics	Chemical			
42087	indoleacetylglutamine	Amino Acid	Tryptophan Metabolism	25200879		HMDB13240
18349	indolelactate	Amino Acid	Tryptophan Metabolism	92904	832-97-3	HMDB00671
32405	indolepropionate	Amino Acid	Tryptophan Metabolism	3744	830-96-6	HMDB02302
43374	indolin-2-one	Xenobiotics	Food Component/Plant	321710	59-48-3	
1123	inosine	Nucleotide	Purine Metabolism, (Hypo)Xanthine/Inosine containing	6021	58-63-9	HMDB00195
33441	isobutyrylcarnitine (C4)	Amino Acid	Leucine, Isoleucine and Valine Metabolism	168379	25518-49-4	HMDB00736
35437	isobutyrylglycine (C4)	Amino Acid	Leucine, Isoleucine and Valine Metabolism	10855600	15926-18-8	HMDB00730
48997	isoeugenol sulfate	Xenobiotics	Food Component/Plant			
1125	isoleucine	Amino Acid	Leucine, Isoleucine and Valine Metabolism	6306	73-32-5	HMDB00172
57577	isoursodeoxycholate	Lipid	Secondary Bile Acid Metabolism	127601	78919-26-3	HMDB00686
44656	isovalerate (C5)	Amino Acid	Leucine, Isoleucine and Valine Metabolism	10430	503-74-2	HMDB00718
34407	isovalerylcarnitine (C5)	Amino Acid	Leucine, Isoleucine and Valine Metabolism	6426851	31023-24-2	HMDB00688
35107	isovalerylglycine	Amino Acid	Leucine, Isoleucine and Valine Metabolism	546304	16284-60-9	HMDB00678
32426	I-urobilinogen	Cofactors and Vitamins	Hemoglobin and Porphyrin Metabolism	26818	14684-37-8	HMDB04157
1417	kynurenate	Amino Acid	Tryptophan Metabolism	3845	492-27-3	HMDB00715

15140	kynurenine	Amino Acid	Tryptophan Metabolism	161166	2922-83-0	HMDB00684
527	lactate	Carbohydrate	Glycolysis, Gluconeogenesis, and Pyruvate Metabolism	612	79-33-4	HMDB00190
57422	lactosyl-N-behenoyl-sphingosine (d18:1/22:0)*	Lipid	Lactosylceramides (LCER)			
57370	lactosyl-N-nervonoyl-sphingosine (d18:1/24:1)*	Lipid	Lactosylceramides (LCER)			
53010	lactosyl-N-palmitoyl-sphingosine (d18:1/16:0)	Lipid	Lactosylceramides (LCER)		4201-62-1	
42002	lanthionine	Amino Acid	Methionine, Cysteine, SAM and Taurine Metabolism	98504	02/08/3183	
1645	laurate (12:0)	Lipid	Medium Chain Fatty Acid	3893	143-07-7	HMDB00638
34534	laurylcarnitine (C12)	Lipid	Fatty Acid Metabolism(Acyl Carnitine)	10427569	25518-54-1	HMDB02250
60	leucine	Amino Acid	Leucine, Isoleucine and Valine Metabolism	6106	61-90-5	HMDB00687
37530	leukotriene B4	Lipid	Eicosanoid	5280492	71160-24-2	HMDB01085
57330	lignoceroyl sphingomyelin (d18:1/24:0)	Lipid	Sphingomyelins		60037-60-7	
57515	lignoceroylcarnitine (C24)*	Lipid	Fatty Acid Metabolism(Acyl Carnitine)			
1105	linoleate (18:2n6)	Lipid	Polyunsaturated Fatty Acid (n3 and n6)	5280450	60-33-3	HMDB00673
34035	linolenate (18:3n3 or 3n6)	Lipid	Polyunsaturated Fatty Acid (n3 and n6)	5280934		HMDB03073
57511	linolenoylcarnitine (C18:3)*	Lipid	Fatty Acid Metabolism(Acyl Carnitine)			
52608	linoleoyl ethanolamide	Lipid	Endocannabinoid	5283446	68171-52-8	HMDB12252
54955	linoleoyl-arachidonoyl-glycerol (18:2/20:4) [1]*	Lipid	Diacylglycerol			HMDB07257
54956	linoleoyl-arachidonoyl-glycerol (18:2/20:4) [2]*	Lipid	Diacylglycerol			HMDB07257
46223	linoleoylcarnitine (C18:2)*	Lipid	Fatty Acid Metabolism(Acyl Carnitine)	6450015	36816-10-1	HMDB06469
57463	linoleoylcholine*	Lipid	Fatty Acid Metabolism (Acyl Choline)			
54950	linoleoyl-docosahexaenoyl-glycerol (18:2/22:6) [2]*	Lipid	Diacylglycerol			HMDB07266
54964	linoleoyl-linolenoyl-glycerol (18:2/18:3) [2]*	Lipid	Diacylglycerol			HMDB07250
54968	linoleoyl-linoleoyl-glycerol (18:2/18:2) [1]*	Lipid	Diacylglycerol			HMDB07248
62526	lithocholate sulfate (1)	Lipid	Secondary Bile Acid Metabolism			
40173	L-urobilin	Cofactors and Vitamins	Hemoglobin and Porphyrin Metabolism	5280818	34217-90-8	HMDB04159

1301	lysine	Amino Acid	Lysine Metabolism	5962	56-87-1	HMDB00182
1303	malate	Energy	TCA Cycle	525	6915-15-7	HMDB00156
20676	maleate	Lipid	Fatty Acid, Dicarboxylate	444266	110-16-7	HMDB00176
46142	mannitol/sorbitol	Carbohydrate	Fructose, Mannose and Galactose Metabolism	5780		HMDB00247
62864	mannonate*	Xenobiotics	Food Component/Plant	3246006	642-99-9	
48153	mannose	Carbohydrate	Fructose, Mannose and Galactose Metabolism	18950	3458-28-4	HMDB00169
1121	margarate (17:0)	Lipid	Long Chain Fatty Acid	10465	506-12-7	HMDB02259
57512	margaroylcarnitine (C17)*	Lipid	Fatty Acid Metabolism(Acyl Carnitine)		106182-29-0	HMDB06210
35174	mead acid (20:3n9)	Lipid	Polyunsaturated Fatty Acid (n3 and n6)	5312531	20590-32-3	HMDB10378
31461	melamine	Xenobiotics	Chemical	7955	108-78-1	HMDB41922
38306	metformin	Xenobiotics	Drug - Metabolic	4091	1115-70-4	HMDB01921
1302	methionine	Amino Acid	Methionine, Cysteine, SAM and Taurine Metabolism	6137	63-68-3	HMDB00696
44878	methionine sulfone	Amino Acid	Methionine, Cysteine, SAM and Taurine Metabolism	69961	820-10-0	
18374	methionine sulfoxide	Amino Acid	Methionine, Cysteine, SAM and Taurine Metabolism	158980	3226-65-1	HMDB02005
46144	methyl glucopyranoside (alpha + beta)	Xenobiotics	Food Component/Plant			
1584	methyl indole-3-acetate	Xenobiotics	Food Component/Plant	74706	1912-33-0	HMDB29738
48429	methyl-4-hydroxybenzoate sulfate	Xenobiotics	Benzoate Metabolism			
61873	methylnaphthyl sulfate (2)*	Xenobiotics	Chemical			
15745	methylsuccinate	Amino Acid	Leucine, Isoleucine and Valine Metabolism	10349	498-21-5	HMDB01844
18037	metoprolol	Xenobiotics	Drug - Cardiovascular	4171	56392-17-7	HMDB01932
34109	metoprolol acid metabolite*	Xenobiotics	Drug - Cardiovascular	62936	56392-14-4	
57642	metronidazole	Xenobiotics	Drug - Antibiotic	4173	73334-05-1	HMDB15052
1124	myo-inositol	Lipid	Inositol Metabolism	892	87-89-8	HMDB00211
1365	myristate (14:0)	Lipid	Long Chain Fatty Acid	11005	544-63-8	HMDB00806
32418	myristoleate (14:1n5)	Lipid	Long Chain Fatty Acid	5281119	544-64-9	HMDB02000
48182	myristoleoylcarnitine (C14:1)*	Lipid	Fatty Acid Metabolism(Acyl Carnitine)	90659872	889848-55-9	
57365	myristoyl dihydrosphingomyelin (d18:0/14:0)*	Lipid	Dihydrosphingomyelins			HMDB12085

33952	myristoylcarnitine (C14)	Lipid	Fatty Acid Metabolism(Acyl Carnitine)	6426854	18822-89-4	HMDB05066
57407	myristoyl-linoleoyl-glycerol (14:0/18:2) [1]*	Lipid	Diacylglycerol			
31536	N-(2-furoyl)glycine	Xenobiotics	Food Component/Plant	21863	5657-19-2;5657-19-2	HMDB00439
57687	N,N,N-trimethyl-5-aminovalerate	Amino Acid	Lysine Metabolism			
62947	N,N,N-trimethyl-alanylproline betaine (TMAP)	Amino Acid	Urea cycle; Arginine and Proline Metabolism			
40469	N1-Methyl-2-pyridone-5-carboxamide	Cofactors and Vitamins	Nicotinate and Nicotinamide Metabolism	69698	701-44-0	HMDB04193
15650	1-methyladenosine	Nucleotide	Purine Metabolism, Adenine containing	27476	15763-06-1	HMDB03331
48351	N1-methylinosine	Nucleotide	Purine Metabolism, (Hypo)Xanthine/Inosine containing	65095	20245-33-4	HMDB02721
35137	N2,N2-dimethylguanosine	Nucleotide	Purine Metabolism, Guanine containing	92919	2140-67-2	HMDB04824
43591	N2,N5-diacetylornithine	Amino Acid	Urea cycle; Arginine and Proline Metabolism	10398396	39825-23-5	
62954	N2-acetyl,N6-methyllysine	Amino Acid	Lysine Metabolism			
62107	N4-acetyl-5-hydroxysulfamethoxazole*	Xenobiotics	Drug - Antibiotic			
35130	N4-acetylcytidine	Nucleotide	Pyrimidine Metabolism, Cytidine containing	107461	3768-18-1	HMDB05923
62106	N4-acetylsulfamethoxazole*	Xenobiotics	Drug - Antibiotic	65280		HMDB13854
1498	N6,N6,N6-trimethyllysine	Amino Acid	Lysine Metabolism	440120	23284-33-5	HMDB01325
62862	N6,N6-dimethyllysine	Amino Acid	Lysine Metabolism	193344	2259-86-1	HMDB13287
36752	N6-acetyllysine	Amino Acid	Lysine Metabolism	92832	692-04-6	HMDB00206
35157	N6-carbamoylthreonyladenosine	Nucleotide	Purine Metabolism, Adenine containing	161466	24719-82-2	HMDB41623
62860	N6-methyllysine	Amino Acid	Lysine Metabolism	164795	1188-07-4	HMDB02038
43255	N-acetyl-1-methylhistidine*	Amino Acid	Histidine Metabolism	193270		
62059	N-acetyl-2-aminooctanoate*	Lipid	Fatty Acid, Amino	95555		HMDB59745
1585	N-acetylalanine	Amino Acid	Alanine and Aspartate Metabolism	88064	97-69-8	HMDB00766
45404	N-acetylalliin	Xenobiotics	Food Component/Plant			
33953	N-acetylarginine	Amino Acid	Urea cycle; Arginine and Proline Metabolism	67427	155-84-0	HMDB04620
22185	N-acetylaspartate (NAA)	Amino Acid	Alanine and Aspartate Metabolism	65065	997-55-7;997-55-7	HMDB00812
35665	N-acetyl-aspartyl-glutamate (NAAG)	Amino Acid	Glutamate Metabolism	5255	3106-85-2	HMDB01067
37432	N-acetyl-beta-alanine	Nucleotide	Pyrimidine Metabolism, Uracil containing	76406	3025-95-4	
43488	N-acetylcarnosine	Amino Acid	Histidine Metabolism	9903482	56353-15-2	HMDB12881

48434	N-acetylcitrulline	Amino Acid	Urea cycle; Arginine and Proline Metabolism	656979	33965-42-3	HMDB00856
46539	N-acetylglucosamine/N-acetylgalactosamine	Carbohydrate	Aminosugar Metabolism	24139		HMDB00215
48149	N-acetylglucosaminylasparagine	Carbohydrate	Aminosugar Metabolism	123826	2776-93-4	HMDB00489
15720	N-acetylglutamate	Amino Acid	Glutamate Metabolism	70914	03/08/5817	HMDB01138
33943	N-acetylglutamine	Amino Acid	Glutamate Metabolism	182230	2490-97-3	HMDB06029
27710	N-acetylglycine	Amino Acid	Glycine, Serine and Threonine Metabolism	10972	543-24-8	HMDB00532
33946	N-acetylhistidine	Amino Acid	Histidine Metabolism	75619	39145-52-3	HMDB32055
33967	N-acetylisoleucine	Amino Acid	Leucine, Isoleucine and Valine Metabolism	2802421	3077-46-1	HMDB61684
62309	N-acetyl-isoputreanine*	Amino Acid	Polyamine Metabolism			
1587	N-acetylleucine	Amino Acid	Leucine, Isoleucine and Valine Metabolism	70912	1188-21-2	HMDB11756
1589	N-acetylmethionine	Amino Acid	Methionine, Cysteine, SAM and Taurine Metabolism	448580	65-82-7	HMDB11745
32377	N-acetylneuraminate	Carbohydrate	Aminosugar Metabolism	439197	131-48-6	HMDB00230
33950	N-acetylphenylalanine	Amino Acid	Phenylalanine Metabolism	74839	2018-61-3	HMDB00512
34387	N-acetylproline	Amino Acid	Urea cycle; Arginine and Proline Metabolism	322640	1074-79-9	
37496	N-acetylputrescine	Amino Acid	Polyamine Metabolism	122356	18233-70-0	HMDB02064
37076	N-acetylserine	Amino Acid	Glycine, Serine and Threonine Metabolism	65249	97-14-3	HMDB02931
48187	N-acetyltaurine	Amino Acid	Methionine, Cysteine, SAM and Taurine Metabolism	159864		
33939	N-acetylthreonine	Amino Acid	Glycine, Serine and Threonine Metabolism	152204	17093-74-2	HMDB62557
33959	N-acetyltryptophan	Amino Acid	Tryptophan Metabolism	700653	1218-34-4	HMDB13713
32390	N-acetyltyrosine	Amino Acid	Tyrosine Metabolism	68310	537-55-3	HMDB00866
1591	N-acetylvaline	Amino Acid	Leucine, Isoleucine and Valine Metabolism	66789	96-81-1	HMDB11757
12122	naproxen	Xenobiotics	Drug - Analgesics, Anesthetics	156391	22204-53-1	HMDB01923
57372	N-behenoyl-sphingadienine (d18:2/22:0)*	Lipid	Ceramides			
62852	N-carbamoylvaline	Amino Acid	Leucine, Isoleucine and Valine Metabolism	5151292	26081-00-5	
43249	N-delta-acetylornithine	Amino Acid	Urea cycle; Arginine and Proline Metabolism	9920500		
57531	nervonoylcarnitine (C24:1)*	Lipid	Fatty Acid Metabolism(Acyl Carnitine)			
43549	N-formylanthranilic acid	Amino Acid	Tryptophan Metabolism	101399	3342-77-6	HMDB04089

2829	N-formylmethionine	Amino Acid	Methionine, Cysteine, SAM and Taurine Metabolism	439750	4289-98-9	HMDB01015
48433	N-formylphenylalanine	Amino Acid	Tyrosine Metabolism	759256	13200-85-6	
594	nicotinamide	Cofactors and Vitamins	Nicotinate and Nicotinamide Metabolism	936	98-92-0	HMDB01406
34598	nifedipine	Xenobiotics	Drug - Cardiovascular	4485	21829-25-4	
57810	nisinate (24:6n3)	Lipid	Polyunsaturated Fatty Acid (n3 and n6)	11792612	68378-49-4	HMDB02007
32462	N-linoleoylglycine	Lipid	Fatty Acid Metabolism(Acyl Glycine)	6433346	06/03/2764	
61823	N-linoleoylserine*	Lipid	Fatty Acid, Amide			
61826	N-linoleoyltaurine*	Lipid	Endocannabinoid			
62068	N-methylhydroxyproline	Amino Acid	Urea cycle; Arginine and Proline Metabolism			
47101	N-methylpipecolate	Xenobiotics	Bacterial/Fungal	11286529	41447-17-0	
37431	N-methylproline	Amino Acid	Urea cycle; Arginine and Proline Metabolism	557	475-11-6	
57664	N-oleoylserine	Lipid	Endocannabinoid			
39732	N-oleoyltaurine	Lipid	Endocannabinoid	6437033	52514-04-2	
1356	nonadecanoate (19:0)	Lipid	Long Chain Fatty Acid	12591	646-30-0	HMDB00772
61775	nonanoylcarnitine (C9)	Lipid	Fatty Acid Metabolism(Acyl Carnitine)			HMDB13288
42092	N-palmitoylglycine	Lipid	Fatty Acid Metabolism(Acyl Glycine)	151008	2441-41-0	HMDB13034
57430	N-palmitoyl-heptadecasphingosine (d17:1/16:0)*	Lipid	Ceramides			
57659	N-palmitoylserine	Lipid	Endocannabinoid	6453686	58725-46-5;474943-14-1	
57416	N-palmitoyl-sphingadienine (d18:2/16:0)*	Lipid	Ceramides			
52604	N-palmitoyl-sphinganine (d18:0/16:0)	Lipid	Dihydroceramides	5283572	5966-29-0	HMDB11760
44877	N-palmitoyl-sphingosine (d18:1/16:0)	Lipid	Ceramides	5283564	24696-26-2	HMDB04949
61822	N-stearoylserine*	Lipid	Endocannabinoid			
1759	N-stearoyl-sphinganine (d18:0/18:0)*	Lipid	Dihydroceramides	5283573	2304-80-5	
54979	N-stearoyl-sphingosine (d18:1/18:0)*	Lipid	Ceramides	5283565	104404-17-3	HMDB04950
39730	N-stearoyltaurine	Lipid	Endocannabinoid	168274	63155-80-6	
31539	O-acetylhomoserine	Amino Acid	Glycine, Serine and Threonine Metabolism	439389	7540-67-2	
36845	o-cresol sulfate	Xenobiotics	Benzoate Metabolism	11615528		
61860	octadecadienedioate (C18:2-DC)*	Lipid	Fatty Acid, Dicarboxylate			
36754	octadecanedioate (C18)	Lipid	Fatty Acid, Dicarboxylate	70095	871-70-5	HMDB00782

61867	octadecanedioylcarnitine (C18-DC)*	Lipid	Fatty Acid Metabolism(Acyl Carnitine)		123233-50-1	
61861	octadecenedioate (C18:1-DC)*	Lipid	Fatty Acid, Dicarboxylate			
61865	octadecenedioylcarnitine (C18:1-DC)*	Lipid	Fatty Acid Metabolism(Acyl Carnitine)		251981-06-3	
33936	octanoylcarnitine (C8)	Lipid	Fatty Acid Metabolism(Acyl Carnitine)	123701	3671-77-0	HMDB00791
52285	oleate/vaccenate (18:1)	Lipid	Long Chain Fatty Acid			
38102	oleoyl ethanolamide	Lipid	Endocannabinoid	5283454	11-58-0;111-58-0	HMDB02088
54960	oleoyl-arachidonoyl-glycerol (18:1/20:4) [1]*	Lipid	Diacylglycerol			HMDB07228
54961	oleoyl-arachidonoyl-glycerol (18:1/20:4) [2]*	Lipid	Diacylglycerol			HMDB07228
35160	oleoylcarnitine (C18)	Lipid	Fatty Acid Metabolism(Acyl Carnitine)	6441392	38677-66-6	HMDB05065
53260	oleoylcholine	Lipid	Fatty Acid Metabolism (Acyl Choline)			
54970	oleoyl-linolenoyl-glycerol (18:1/18:3) [2]*	Lipid	Diacylglycerol			HMDB07220
46798	oleoyl-linoleoyl-glycerol (18:1/18:2) [1]	Lipid	Diacylglycerol		106292-55-1	HMDB07219
46799	oleoyl-linoleoyl-glycerol (18:1/18:2) [2]	Lipid	Diacylglycerol		104346-53-4	HMDB07219
54945	oleoyl-oleoyl-glycerol (18:1/18:1) [1]*	Lipid	Diacylglycerol			HMDB07218
54946	oleoyl-oleoyl-glycerol (18:1/18:1) [2]*	Lipid	Diacylglycerol			HMDB07218
1493	ornithine	Amino Acid	Urea cycle; Arginine and Proline Metabolism	6262	3184-13-2	HMDB03374
1505	orotate	Nucleotide	Pyrimidine Metabolism, Orotate containing	967	50887-69-9	HMDB00226
35172	orotidine	Nucleotide	Pyrimidine Metabolism, Orotate containing	92751	314-50-1	HMDB00788
45413	O-sulfo-L-tyrosine	Xenobiotics	Chemical	514186		
20694	oxalate (ethanedioate)	Cofactors and Vitamins	Ascorbate and Aldarate Metabolism	971	144-62-7	HMDB02329
41726	oxypurinol	Xenobiotics	Drug - Metabolic	4644	2465-59-0	HMDB00786
1336	palmitate (16:0)	Lipid	Long Chain Fatty Acid	985	1957/10/3	HMDB00220
62438	palmitoleamide (16:1)*	Lipid	Fatty Acid, Amide			
33447	palmitoleate (16:1n7)	Lipid	Long Chain Fatty Acid	445638	373-49-9	HMDB03229
57406	palmitoleoyl-arachidonoyl-glycerol (16:1/20:4) [2]*	Lipid	Diacylglycerol			
53223	palmitoleoylcarnitine (C16:1)*	Lipid	Fatty Acid Metabolism(Acyl Carnitine)	71464547		
54967	palmitoleoyl-linoleoyl-glycerol (16:1/18:2) [1]*	Lipid	Diacylglycerol			HMDB07132

52434	palmitoyl dihydrosphingomyelin (d18:0/16:0)*	Lipid	Dihydrosphingomyelins	9939965		
37506	palmitoyl sphingomyelin (d18:1/16:0)	Lipid	Sphingomyelins	9939941	6254-89-3	
54957	palmitoyl-arachidonoyl-glycerol (16:0/20:4) [1]*	Lipid	Diacylglycerol			HMDB07112
54958	palmitoyl-arachidonoyl-glycerol (16:0/20:4) [2]*	Lipid	Diacylglycerol			HMDB07112
44681	palmitoylcarnitine (C16)	Lipid	Fatty Acid Metabolism(Acyl Carnitine)	461	6865-14-1	HMDB00222
52944	palmitoylcholine	Lipid	Fatty Acid Metabolism (Acyl Choline)	151731		
52633	palmitoyl-linoleoyl-glycerol (16:0/18:2) [1]*	Lipid	Diacylglycerol	9543695		HMDB07103
52634	palmitoyl-linoleoyl-glycerol (16:0/18:2) [2]*	Lipid	Diacylglycerol			HMDB07103
54943	palmitoyl-oleoyl-glycerol (16:0/18:1) [1]*	Lipid	Diacylglycerol			HMDB07102
54942	palmitoyl-oleoyl-glycerol (16:0/18:1) [2]*	Lipid	Diacylglycerol			HMDB07102
1508	pantothenate (Vitamin B5)	Cofactors and Vitamins	Pantothenate and CoA Metabolism	6613	137-08-6	HMDB00210
18254	paraxanthine	Xenobiotics	Xanthine Metabolism	4687	611-59-6	HMDB01860
48841	p-cresol glucuronide*	Amino Acid	Tyrosine Metabolism	154035	17680-99-8	HMDB11686
36103	p-cresol sulfate	Xenobiotics	Benzoate Metabolism	4615423	3233-57-7	HMDB11635
1361	pentadecanoate (15:0)	Lipid	Long Chain Fatty Acid	13849	1002-84-2;10002-84-2	HMDB00826
57564	perfluorooctanesulfonate (PFOS)	Xenobiotics	Chemical	74483	1763-23-1	HMDB59586
62071	perfluorooctanoate (PFOA)*	Xenobiotics	Chemical	9554		HMDB59587
52747	phenol glucuronide	Amino Acid	Tyrosine Metabolism	87235	17685-05-1	HMDB60014
32553	phenol sulfate	Amino Acid	Tyrosine Metabolism	74426	937-34-8	HMDB60015
32396	phenolphthalein beta-D-glucuronide	Xenobiotics	Chemical	3032634	6820-54-8	
57745	phenylacetate	Amino Acid	Phenylalanine Metabolism	999	103-82-2	HMDB00209
48425	phenylacetylcarnitine	Peptide	Acetylated Peptides	101724840	287108-21-8	
52925	phenylacetylglutamate	Peptide	Acetylated Peptides	11579826		HMDB59772
35126	phenylacetylglutamine	Peptide	Acetylated Peptides	92258	28047-15-6	HMDB06344
64	phenylalanine	Amino Acid	Phenylalanine Metabolism	6140	63-91-2	HMDB00159
22130	phenyllactate (PLA)	Amino Acid	Phenylalanine Metabolism	3848	828-01-3	HMDB00779
566	phenylpyruvate	Amino Acid	Phenylalanine Metabolism	997	156-06-9	HMDB00205
42109	phosphate	Energy	Oxidative Phosphorylation	1061	7664-38-2	HMDB01429
1600	phosphoethanolamine (PE)	Lipid	Phospholipid Metabolism	1015	1071-23-4	HMDB00224
1512	picolinate	Amino Acid	Tryptophan Metabolism	1018	98-98-6	HMDB02243
15704	pimelate (C7-DC)	Lipid	Fatty Acid, Dicarboxylate	385	111-16-0	HMDB00857
53224	pimeloylcarnitine/3-methyladipoylcarnitine (C7-DC)	Lipid	Fatty Acid			

			Metabolism(Acyl Carnitine)			
1444	pipecolate	Amino Acid	Lysine Metabolism	849	4043-87-2	HMDB00070
33935	piperine	Xenobiotics	Food Component/Plant	638024	94-62-2	HMDB29377
54742	prednisolone	Xenobiotics	Drug - Antiinflammatory, Immunosuppressant	5755		HMDB14998
40708	pregnanediol-3-glucuronide	Lipid	Progestin Steroids	123796	1852-49-9	HMDB10318
48394	pregnanolone/allopregnanolone sulfate	Lipid	Progestin Steroids			
32562	pregnen-diol disulfate*	Lipid	Pregnenolone Steroids			
32619	pregnenediol sulfate (C21H34O5S)*	Lipid	Pregnenolone Steroids		1247-64-9	
62921	pregnenetriol disulfate*	Lipid	Pregnenolone Steroids			
62922	pregnenetriol sulfate*	Lipid	Pregnenolone Steroids			
38170	pregnenolone sulfate	Lipid	Pregnenolone Steroids	105074	1247-64-9	HMDB00774
35127	prolylhydroxyproline	Amino Acid	Urea cycle; Arginine and Proline Metabolism	11673055	18684-24-7	HMDB06695
1898	proline	Amino Acid	Urea cycle; Arginine and Proline Metabolism	145742	147-85-3	HMDB00162
40703	prolylglycine	Peptide	Dipeptide	6426709	2578-57-6	HMDB11178
32452	propionylcarnitine (C3)	Lipid	Fatty Acid Metabolism (also BCAA Metabolism)	107738	17298-37-2	HMDB00824
31932	propionylglycine (C3)	Lipid	Fatty Acid Metabolism (also BCAA Metabolism)	98681	21709-90-0	HMDB00783
48460	propyl 4-hydroxybenzoate sulfate	Xenobiotics	Benzoate Metabolism		162338-10-5	
33442	pseudouridine	Nucleotide	Pyrimidine Metabolism, Uracil containing	15047	1445-07-4	HMDB00767
1651	pyridoxal	Cofactors and Vitamins	Vitamin B6 Metabolism	1050	65-22-5	HMDB01545
31555	pyridoxate	Cofactors and Vitamins	Vitamin B6 Metabolism	6723	82-82-6	HMDB00017
608	pyridoxine (Vitamin B6)	Cofactors and Vitamins	Vitamin B6 Metabolism	1054	58-56-0	HMDB02075
46225	pyroglutamine*	Amino Acid	Glutamate Metabolism	134508	2353-44-8	
48990	pyruvate	Carbohydrate	Glycolysis, Gluconeogenesis, and Pyruvate Metabolism	1060	127-17-3	HMDB00243
18335	quinate	Xenobiotics	Food Component/Plant	6508	77-95-2	HMDB03072
1899	quinolinate	Cofactors and Vitamins	Nicotinate and Nicotinamide Metabolism	1066	89-00-9	HMDB00232
38595	ranitidine	Xenobiotics	Drug - Gastrointestinal	3001055	66357-59-3	HMDB01930
62485	ranitidine N-oxide*	Xenobiotics	Drug - Gastrointestinal	3033888		
1806	retinol (Vitamin A)	Cofactors and Vitamins	Vitamin A Metabolism	445354	68-26-8	HMDB00305
15772	ribitol	Carbohydrate	Pentose Metabolism	6912	488-81-3	HMDB00508
27731	ribonate (ribonolactone)	Carbohydrate	Pentose Metabolism	5460677	03/08/5336	HMDB00867

61858	ribulonate/xylulonate*	Carbohydrate	Pentose Metabolism			
44552	S-(3-hydroxypropyl)mercapturic acid (HPMA)	Xenobiotics	Chemical	3371179	23127-40-4	
42370	S-1-pyrroline-5-carboxylate	Amino Acid	Glutamate Metabolism	1196	2906-39-0	HMDB01301
21151	saccharin	Xenobiotics	Food Component/Plant	5143	1981/7/2	HMDB29723
42382	S-adenosylhomocysteine (SAH)	Amino Acid	Methionine, Cysteine, SAM and Taurine Metabolism	439155	979-92-0	HMDB00939
1515	salicylate	Xenobiotics	Drug - Topical Agents	338	69-72-7	HMDB01895
33384	salicyluric glucuronide*	Xenobiotics	Drug - Analgesics, Anesthetics			
43239	S-allylcysteine	Xenobiotics	Food Component/Plant	98280	21593-77-1	HMDB34323
1516	sarcosine	Amino Acid	Glycine, Serine and Threonine Metabolism	1088	107-97-1	HMDB00271
32398	sebacate (C10-DC)	Lipid	Fatty Acid, Dicarboxylate	5192	111-20-6	HMDB00792
1648	serine	Amino Acid	Glycine, Serine and Threonine Metabolism	5951	56-45-1	HMDB00187
39592	S-methylcysteine	Amino Acid	Methionine, Cysteine, SAM and Taurine Metabolism	24417	1187-84-4	HMDB02108
43378	S-methylcysteine sulfoxide	Amino Acid	Methionine, Cysteine, SAM and Taurine Metabolism	82142	6853-87-8	HMDB29432
38127	S-methylmethionine	Amino Acid	Methionine, Cysteine, SAM and Taurine Metabolism	458	07/12/3493	HMDB38670
485	spermidine	Amino Acid	Polyamine Metabolism	1102	124-20-9	HMDB01257
57426	sphingadienine	Lipid	Sphingolipid Synthesis	6449795	25696-03-1	
17769	sphinganine	Lipid	Sphingolipid Synthesis	3126	3102-56-5	HMDB00269
52605	sphinganine-1-phosphate	Lipid	Sphingolipid Synthesis	520	19794-97-9	HMDB01383
62152	sphingomyelin (d17:1/14:0, d16:1/15:0)*	Lipid	Sphingomyelins			
52433	sphingomyelin (d17:1/16:0, d18:1/15:0, d16:1/17:0)*	Lipid	Sphingomyelins		121999-58-4	
57483	sphingomyelin (d17:2/16:0, d18:2/15:0)*	Lipid	Sphingomyelins			
57473	sphingomyelin (d18:0/18:0, d19:0/17:0)*	Lipid	Dihydrosphingomyelins			HMDB12087
57476	sphingomyelin (d18:0/20:0, d16:0/22:0)*	Lipid	Dihydrosphingomyelins			
42463	sphingomyelin (d18:1/14:0, d16:1/16:0)*	Lipid	Sphingomyelins	11433862		HMDB12097
52615	sphingomyelin (d18:1/17:0, d17:1/18:0, d19:1/16:0)	Lipid	Sphingomyelins		121999-64-2	
37529	sphingomyelin (d18:1/18:1, d18:2/18:0)	Lipid	Sphingomyelins	6443882	108392-10-5	HMDB12101
57475	sphingomyelin (d18:1/19:0, d19:1/18:0)*	Lipid	Sphingomyelins			
48490	sphingomyelin (d18:1/20:0, d16:1/22:0)*	Lipid	Sphingomyelins			HMDB12102
48491	sphingomyelin (d18:1/20:1, d18:2/20:0)*	Lipid	Sphingomyelins		222403-67-0	

57481	sphingomyelin (d18:1/20:2, d18:2/20:1, d16:1/22:2)*	Lipid	Sphingomyelins			
52495	sphingomyelin (d18:1/21:0, d17:1/22:0, d16:1/23:0)*	Lipid	Sphingomyelins			
48493	sphingomyelin (d18:1/22:1, d18:2/22:0, d16:1/24:1)*	Lipid	Sphingomyelins			HMDB12104
57477	sphingomyelin (d18:1/22:2, d18:2/22:1, d16:1/24:2)*	Lipid	Sphingomyelins			
47153	sphingomyelin (d18:1/24:1, d18:2/24:0)*	Lipid	Sphingomyelins		94359-13-4	HMDB12107
57478	sphingomyelin (d18:1/25:0, d19:0/24:1, d20:1/23:0, d19:1/24:0)*	Lipid	Sphingomyelins			
47154	sphingomyelin (d18:2/14:0, d18:1/14:1)*	Lipid	Sphingomyelins			
42459	sphingomyelin (d18:2/16:0, d18:1/16:1)*	Lipid	Sphingomyelins			
57474	sphingomyelin (d18:2/18:1)*	Lipid	Sphingomyelins			
57480	sphingomyelin (d18:2/21:0, d16:2/23:0)*	Lipid	Sphingomyelins			
52435	sphingomyelin (d18:2/23:0, d18:1/23:1, d17:1/24:1)*	Lipid	Sphingomyelins			
57482	sphingomyelin (d18:2/23:1)*	Lipid	Sphingomyelins			
52437	sphingomyelin (d18:2/24:1, d18:1/24:2)*	Lipid	Sphingomyelins			
57479	sphingomyelin (d18:2/24:2)*	Lipid	Sphingomyelins			
17747	sphingosine	Lipid	Sphingosines	5353955	123-78-4	HMDB00252
34445	sphingosine 1-phosphate	Lipid	Sphingosines	5283560	26993-30-6	HMDB00277
34384	stachydrine	Xenobiotics	Food Component/Plant	115244	4136-37-2	HMDB04827
37487	stearamide (18:0)	Lipid	Fatty Acid, Amide	31292	124-26-5	HMDB34146
1358	stearate (18:0)	Lipid	Long Chain Fatty Acid	5281	1957/11/4	HMDB00827
33969	stearidonate (18:4n3)	Lipid	Polyunsaturated Fatty Acid (n3 and n6)	5312508	111174-40-4	HMDB06547
19503	stearoyl sphingomyelin (d18:1/18:0)	Lipid	Sphingomyelins	6453725	85187-10-6;85187-10-6	HMDB01348
57450	stearoyl-arachidonoyl-glycerol (18:0/20:4) [1]*	Lipid	Diacylglycerol			
57449	stearoyl-arachidonoyl-glycerol (18:0/20:4) [2]*	Lipid	Diacylglycerol			
34409	stearoylcarnitine (C18)	Lipid	Fatty Acid Metabolism(Acyl Carnitine)	6426855	18822-91-8	HMDB00848
57464	stearoylcholine*	Lipid	Fatty Acid Metabolism (Acyl Choline)			
15730	suberate (C8-DC)	Lipid	Fatty Acid, Dicarboxylate	10457	505-48-6	HMDB00893
52990	suberoylcarnitine (C8-DC)	Lipid	Fatty Acid Metabolism(Acyl Carnitine)			
1437	succinate	Energy	TCA Cycle	1110	110-15-6	HMDB00254
41888	succinimide	Xenobiotics	Chemical	11439	123-56-8	
37058	succinylcarnitine (C4)	Energy	TCA Cycle	71464481	256928-74-2	HMDB61717
1519	sucrose	Carbohydrate	Disaccharides and	5988	57-50-1	HMDB00258

			Oligosaccharides			
57653	sulbactam	Xenobiotics	Drug - Antibiotic	130313	68373-14-8	
53240	sulfamethoxazole	Xenobiotics	Drug - Antibiotic	5329		HMDB15150
46960	sulfate*	Xenobiotics	Chemical	1118	14808-79-8	HMDB01448
48454	syringol sulfate	Xenobiotics	Food Component/Plant		52904-67-3	
15336	tartarate	Xenobiotics	Food Component/Plant	444305	87-69-4;6106-24-7	HMDB00956
20693	tartronate (hydroxymalonate)	Xenobiotics	Bacterial/Fungal	45	80-69-3	HMDB35227
2125	taurine	Amino Acid	Methionine, Cysteine, SAM and Taurine Metabolism	1123	107-35-7	HMDB00251
18494	taurochenodeoxycholate	Lipid	Primary Bile Acid Metabolism	387316	6009-98-9	HMDB00951
62890	taurochenodeoxycholic acid 3-sulfate	Lipid	Secondary Bile Acid Metabolism			HMDB02486
18497	taurocholate	Lipid	Primary Bile Acid Metabolism	6675	145-42-6	HMDB00036
32807	taurocholenate sulfate*	Lipid	Secondary Bile Acid Metabolism			
12261	taurodeoxycholate	Lipid	Secondary Bile Acid Metabolism	2733768	207737-97-1	HMDB00896
62889	taurodeoxycholic acid 3-sulfate	Lipid	Secondary Bile Acid Metabolism			
36850	taurolithocholate 3-sulfate	Lipid	Secondary Bile Acid Metabolism	440071	64936-83-0	HMDB02580
39378	tauroursodeoxycholate	Lipid	Secondary Bile Acid Metabolism	9848818	14605-22-2	HMDB00874
62918	tetradecadienedioate (C14:2-DC)*	Lipid	Fatty Acid, Dicarboxylate			
62850	tetradecadienoate (14:2)*	Lipid	Polyunsaturated Fatty Acid (n3 and n6)	5312409	39039-37-7	HMDB00560
35669	tetradecanedioate (C14)	Lipid	Fatty Acid, Dicarboxylate	13185	821-38-5	HMDB00872
22206	theanine	Xenobiotics	Food Component/Plant	439378	3081-61-6	HMDB34365
18392	theobromine	Xenobiotics	Xanthine Metabolism	5429	83-67-0	HMDB02825
18394	theophylline	Xenobiotics	Xanthine Metabolism	2153	58-55-9	HMDB01889
53231	thioproline	Xenobiotics	Chemical	93176	34592-47-4	
27738	threonate	Cofactors and Vitamins	Ascorbate and Aldarate Metabolism	151152	70753-61-6	HMDB00943
1284	threonine	Amino Acid	Glycine, Serine and Threonine Metabolism	6288	72-19-5	HMDB00167
36095	thymol sulfate	Xenobiotics	Food Component/Plant		89-83-8	HMDB01878
2761	thyroxine	Amino Acid	Tyrosine Metabolism	5819	51-48-9	HMDB01918
35428	tiglyl carnitine (C5)	Amino Acid	Leucine, Isoleucine and Valine Metabolism	22833596	64191-86-2	HMDB02366
32306	hydroxyproline	Amino Acid	Urea cycle; Arginine and Proline Metabolism	5810	51-35-4	HMDB00725

607	trans-urocanate	Amino Acid	Histidine Metabolism	736715	104-98-3	HMDB00301
52436	tricosanoyl sphingomyelin (d18:1/23:0)*	Lipid	Sphingomyelins			HMDB12105
62919	tridecenedioate (C13:1-DC)*	Lipid	Fatty Acid, Dicarboxylate			
32401	trigonelline (N'-methylnicotinate)	Cofactors and Vitamins	Nicotinate and Nicotinamide Metabolism	5570	535-83-1	HMDB00875
40406	trimethylamine N-oxide	Lipid	Phospholipid Metabolism	1145	1184-78-7	HMDB00925
54	tryptophan	Amino Acid	Tryptophan Metabolism	6305	73-22-3	HMDB00929
37097	tryptophan betaine	Amino Acid	Tryptophan Metabolism	442106	20671-76-5	HMDB61115
48408	tyramine O-sulfate	Amino Acid	Tyrosine Metabolism	153005	30223-92-8	HMDB06409
1299	tyrosine	Amino Acid	Tyrosine Metabolism	6057	60-18-4	HMDB00158
48674	umbelliferone sulfate	Xenobiotics	Food Component/Plant	129659	69526-88-1	
42395	undecanedioate (C11-DC)	Lipid	Fatty Acid, Dicarboxylate	15816	1852-04-6	HMDB00888
605	uracil	Nucleotide	Pyrimidine Metabolism, Uracil containing	1174	66-22-8	HMDB00300
1604	urate	Nucleotide	Purine Metabolism, (Hypo)Xanthine/Inosine containing	1175	69-93-2;120K5305	HMDB00289
1670	urea	Amino Acid	Urea cycle; Arginine and Proline Metabolism	1176	57-13-6	HMDB00294
606	uridine	Nucleotide	Pyrimidine Metabolism, Uracil containing	6029	58-96-8	HMDB00296
1605	ursodeoxycholate	Lipid	Secondary Bile Acid Metabolism	31401	128-13-2	HMDB00946
33443	valerate (5:0)	Lipid	Short Chain Fatty Acid	7991	109-52-4	HMDB00892
1649	valine	Amino Acid	Leucine, Isoleucine and Valine Metabolism	6287	72-18-4	HMDB00883
39994	valylleucine	Peptide	Dipeptide	352039	22906-55-4	HMDB29131
54910	vanillactate	Amino Acid	Tyrosine Metabolism	160637	2475-56-1	HMDB00913
1567	vanillylmandelate (VMA)	Amino Acid	Tyrosine Metabolism	1245	1955/10/7	HMDB00291
3147	xanthine	Nucleotide	Purine Metabolism, (Hypo)Xanthine/Inosine containing	1188	69-89-6	HMDB00292
15679	xanthurenate	Amino Acid	Tryptophan Metabolism	5699	59-007	HMDB00881
31538	XHWESASXXR*	Peptide	Polypeptide			
57517	ximenoylcarnitine (C26:1)*	Lipid	Fatty Acid Metabolism(Acyl Carnitine)			
15581	xylose	Carbohydrate	Pentose Metabolism	135191	609-06-3	HMDB00098
46590	X - 07765					
46355	X - 09789					
47478	X - 10458					

32578	X - 11261
46390	X - 11308
49592	X - 11315
46594	X - 11372
46595	X - 11378
46347	X - 11381
46596	X - 11407
46360	X - 11441
46443	X - 11442
46460	X - 11444
46601	X - 11470
46602	X - 11478
47651	X - 11491
46914	X - 11522
46603	X - 11530
47493	X - 11632
33132	X - 11787
33140	X - 11795
46466	X - 11843
46488	X - 11847
46607	X - 11849
46507	X - 11850
46521	X - 11852
47654	X - 11858
47655	X - 11861
46608	X - 11880
46610	X - 12007
46611	X - 12013
47390	X - 12015
47905	X - 12026
46588	X - 12063
46684	X - 12093 - retired for N2-acetyl,N6-methyllysine
47687	X - 12100

47642	X - 12101
46932	X - 12104
47690	X - 12112
47695	X - 12117
47702	X - 12127
47909	X - 12170
46985	X - 12193
46612	X - 12206
46358	X - 12212
46613	X - 12216
47911	X - 12221
46614	X - 12230
47657	X - 12261
47914	X - 12262
47708	X - 12283
47917	X - 12306
47659	X - 12329
46615	X - 12407
47921	X - 12410
46616	X - 12411
47639	X - 12456
46620	X - 12462
46628	X - 12472
46621	X - 12524
47922	X - 12543
46510	X - 12544
47437	X - 12680
47719	X - 12689
47929	X - 12707
46973	X - 12718
46623	X - 12729
47937	X - 12730
46626	X - 12739

46627	X - 12740
47723	X - 12753
46986	X - 12812
46997	X - 12822
46633	X - 12844
46634	X - 12846
46364	X - 12847
46636	X - 12849
46639	X - 12851
47954	X - 12879
47955	X - 12906
47788	X - 13431
47439	X - 13507
47959	X - 13553
47664	X - 13658
47790	X - 13684
47962	X - 13695
47967	X - 13726
47971	X - 13729
47441	X - 13737
46646	X - 13835
47977	X - 13844
46417	X - 13866
46632	X - 14056
46656	X - 14662
46657	X - 14939
46658	X - 15220
46661	X - 15245
46283	X - 15461
46640	X - 15469
46266	X - 15486
46662	X - 15492
46977	X - 15503

46664	X - 15664
47800	X - 15666
46666	X - 15728
47988	X - 16087
46461	X - 16124
47802	X - 16397
47013	X - 16570
46673	X - 16576
47804	X - 16580
47992	X - 16649
46677	X - 16654
46680	X - 16932
46681	X - 16935
46511	X - 16938
46683	X - 16944
46517	X - 16946
46685	X - 16964
47993	X - 17010
46686	X - 17137
46689	X - 17145
47806	X - 17162
46490	X - 17185
46704	X - 17269
46672	X - 17325
47820	X - 17335
47821	X - 17337
47872	X - 17340
48001	X - 17351
48009	X - 17357
48011	X - 17359
46976	X - 17365
46994	X - 17367
46705	X - 17438

48052	X - 17612
46707	X - 17653
46709	X - 17654
48019	X - 17655
48021	X - 17673
46970	X - 17676
48025	X - 17685
46366	X - 18249
47342	X - 18345
48047	X - 18886
48048	X - 18888
47670	X - 18899
46690	X - 18901
46695	X - 18913
46368	X - 18914
47671	X - 18921
46700	X - 18922
46701	X - 19141
47672	X - 19183
46261	X - 19438
46259	X - 21258
46295	X - 21286
46316	X - 21295
46354	X - 21310
46359	X - 21315
46363	X - 21319
46384	X - 21339
46387	X - 21342
46398	X - 21353
46409	X - 21364
46455	X - 21410
46456	X - 21411
46486	X - 21441

46493	X - 21448
46512	X - 21467
46515	X - 21470
46516	X - 21471
46674	X - 21607
46730	X - 21661
46898	X - 21729
46902	X - 21733
46905	X - 21736
46961	X - 21785
46968	X - 21792
46972	X - 21796
46980	X - 21803
46984	X - 21807
46998	X - 21821
47006	X - 21829
47011	X - 21834
47205	X - 21959
47389	X - 22143
47417	X - 22162
47783	X - 22519
47784	X - 22520
48067	X - 22764
48076	X - 22771
48080	X - 22775
48081	X - 22776
48185	X - 22834
52659	X - 22918
48774	X - 23160
48892	X - 23196
48972	X - 23276
48993	X - 23294
48994	X - 23295

48995	X - 23296
48996	X - 23297
49704	X - 23314
49228	X - 23369
49374	X - 23498
49383	X - 23507
49459	X - 23583
49461	X - 23585
49463	X - 23587
49466	X - 23590
49469	X - 23593
49512	X - 23636
49515	X - 23639
49521	X - 23644
49525	X - 23648
49539	X - 23662
49542	X - 23665
49543	X - 23666
49557	X - 23680
49637	X - 23739
49665	X - 23767
49679	X - 23780
49681	X - 23782
49688	X - 23787
49883	X - 23974
52025	X - 23997
52106	X - 24077
52135	X - 24106
52280	X - 24241
52286	X - 24243
52297	X - 24293
52483	X - 24295
52501	X - 24306

52502	X - 24307
52524	X - 24328
52533	X - 24337
52540	X - 24344
52640	X - 24411
52647	X - 24418
52636	X - 24422
52661	X - 24431
52662	X - 24432
52665	X - 24435
52772	X - 24455
52773	X - 24456
52779	X - 24462
52790	X - 24473
52792	X - 24475
52839	X - 24518
52848	X - 24527
52861	X - 24540
52863	X - 24542
52865	X - 24544
52867	X - 24546
52870	X - 24549
52877	X - 24556
52886	X - 24565
52892	X - 24571
52909	X - 24588
53023	X - 24608
53065	X - 24637
53114	X - 24686
53127	X - 24699
53156	X - 24727
53157	X - 24728
53172	X - 24736

53267	X - 24748
54703	X - 24765
54840	X - 24812
54841	X - 24813
54877	X - 24849
57714	X - 24947
57716	X - 24949
57718	X - 24951
57719	X - 24952
57756	X - 24972
62151	X - 25172
62406	X - 25220
62494	X - 25265
62495	X - 25266
62496	X - 25267
62500	X - 25271
62502	X - 25273
62521	X - 25279
62611	X - 25318
62636	X - 25343
62664	X - 25371
62681	X - 25388
62716	X - 25419
62717	X - 25420
62719	X - 25422
62876	X - 25463

Compound ID in different databases: KEGG, Kyoto Encyclopedia of Genes and Genomes; CAS, Chemical Abstracts Service; PubChem; HMDB: Human Metabolome Database.
Abbreviation: RI, retention index.

Supplementary Table 2. Genetic variants associated with BMI in the GIANT consortium

SNP	UKB+GIANT		CKB (orientated to BMI-increasing allele in CEU)				BBJ (orientated to BMI-increasing allele in CEU)			
	BMI-increasing allele	EAF	EAF	SD difference	SE	*p*-value	EAF	SD difference	SE	*p*-value
rs1000096	C	0.38	0.19	0.001	0.006	0.913	0.17	-0.004	0.005	0.443
rs10002111	A	0.22	0.04	0.011	0.011	0.326	0.06	0.012	0.007	0.108
rs1000940	G	0.28	0.38	0.004	0.004	0.38	0.58	0.009	0.004	0.016
rs10044136	G	0.57	0.03	-0.009	0.012	0.447	0.03	0.011	0.011	0.355
rs1006893	C	0.76	0.71	-0.019	0.005	4.54E-05	0.76	0.016	0.004	<5E-08
rs1009188	T	0.71	0.86	0.011	0.006	0.0682	0.83	-0.024	0.005	<5E-08
rs10092723	C	0.25	0.23	-0.004	0.005	0.387	0.26	0.005	0.004	0.275
rs10099330	G	0.54	0.5	-0.011	0.004	0.0113	0.54	0.001	0.004	0.812
rs10101364	T	0.68	0.99	0.021	0.02	0.28	0.98	-0.022	0.013	0.086
rs10116186	G	0.46	0.79	-0.007	0.005	0.161	0.8	0.01	0.004	0.029
rs10132280	C	0.31	0.09	-0.024	0.008	0.00157	0.07	0.018	0.007	0.01
rs10197031	C	0.27	0.41	-0.014	0.005	0.00235	0.54	0.009	0.004	0.015
rs10198345	C	0.66	0.75	0.006	0.005	0.213	0.72	-0.001	0.004	0.736
rs10203386	A	0.45	0.43	0.034	0.004	3.78E-15	0.47	-0.023	0.004	<5E-08
rs10269783	A	0.42	0.6	0.013	0.004	0.00208	0.58	-0.015	0.004	<5E-08
rs1030015	T	0.54	0.34	0.012	0.004	0.0062	0.38	-0.005	0.004	0.205
rs1031670	C	0.52	0.47	-0.008	0.004	0.0593	0.58	0	0.004	0.89
rs1035010	T	0.25	0.29	0.007	0.005	0.125	0.28	-0.023	0.004	<5E-08
rs1038088	G	0.49	0.68	-0.014	0.005	0.00265	0.68	0.016	0.004	<5E-08
rs10402145	A	0.3	0.13	0.005	0.006	0.413	0.17	0.004	0.005	0.366
rs1045411	C	0.28	0.2	-0.009	0.005	0.0984	0.15	0.002	0.005	0.736
rs1048303	C	0.58	0.43	-0.009	0.004	0.0441	0.33	0.01	0.004	0.011
rs10497870	A	0.61	0.33	0.008	0.004	0.071	0.3	-0.003	0.004	0.385
rs10498767	G	0.45	0.38	-0.01	0.005	0.0287	0.4	0.008	0.004	0.033
rs10498891	T	0.93	0.99	0.068	0.061	0.27		Low MAF*		
rs10499276	T	0.11	0.06	0.015	0.009	0.0963	0.05	-0.008	0.009	0.377

rs10499694	A	0.49	0.61	0.006	0.004	0.149	0.6	-0.005	0.004	0.214
rs10510419	G	0.15	0.003	0.05	0.044	0.263		Low MAF*		
rs10518269	C	0.17	0.001	-0.031	0.022	0.155		Low MAF*		
rs10744146	G	0.54	0.68	-0.013	0.005	0.00542	0.69	0.015	0.004	<5E-08
rs1075901	C	0.45	0.79	-0.011	0.005	0.0276	0.74	0.011	0.004	0.53
rs10761247	G	0.59	0.44	0.004	0.004	0.344	0.54	0.011	0.004	0.001
rs10761785	G	0.51	0.66	-0.011	0.004	0.0153	0.69	0	0.00393	0.001465
rs10783779	T	0.6	0.7	-0.001	0.005	0.812	0.66	0.002	0.004	0.593
rs10792006	T	0.45	0.71	0.006	0.005	0.183	0.66	-0.004	0.004	0.257
rs10797987	C	0.51	0.32	-0.003	0.005	0.557	0.38	0.01	0.004	0.008
rs10823893	A	0.41	0.08	0.001	0.008	0.9	0.04	-0.002	0.009	0.825
rs10840606	G	0.82	0.95	-0.023	0.01	0.0156	0.96	0.032	0.009	0.001
rs10850185	A	0.3	0.4	0.014	0.004	0.00112	0.36	-0.006	0.004	0.13
rs10850777	A	0.44	0.43	-0.004	0.004	0.368	0.43	-0.01	0.004	0.005
rs10858334	G	0.85	0.9	-0.011	0.007	0.111	0.88	-0.001	0.006	0.815
rs10871589	G	0.68	0.64	-0.009	0.004	0.0394	0.62	0.016	0.004	<5E-08
rs10886017	A	0.24	0.4	0.005	0.004	0.28	0.36	-0.006	0.004	0.125
rs10887584	A	0.45	0.27	0.017	0.005	0.000262	0.36	-0.002	0.004	0.627
rs10892873	C	0.36	0.18	0.009	0.006	0.0954	0.05	-0.006	0.008	0.358
rs10896012	C	0.78	0.85	0.009	0.006	0.133	0.82	0.002	0.005	0.716
rs10920678	A	0.43	0.34	0.01	0.004	0.0219	0.35	-0.021	0.004	<5E-08
rs10923724	C	0.56	0.6	-0.006	0.004	0.169	0.61	0.015	0.004	<5E-08
rs10929925	C	0.41	0.39	-0.016	0.004	0.00021	0.42	0.006	0.004	0.103
rs10930502	A	0.69	0.31	-0.003	0.005	0.585	0.29	-0.002	0.004	0.606
rs10937094	A	0.71	0.87	0.004	0.006	0.512	0.77	-0.002	0.004	0.693
rs10938397	G	0.57	0.7	-0.037	0.005	1.45E-15	0.7	0.028	0.004	<5E-08
rs10942267	A	0.69	0.82	0.008	0.005	0.134	0.85	-0.017	0.005	0.001
rs10950289	A	0.84	0.76	-0.005	0.005	0.382	0.76	-0.001	0.005	0.84
rs10954772	T	0.32	0.04	-0.015	0.011	0.192		Low MAF*		
rs10965780	G	0.41	0.15	-0.008	0.006	0.191	0.27	0.019	0.004	<5E-08
rs10971721	C	0.1	0.01	0.04	0.025	0.117		Low MAF*		
rs10984756	G	0.91	0.99	0.086	0.032	0.00761		Low MAF*		

rs10985968	C	0.5	0.52	0.003	0.004	0.531	0.57	-0.002	0.004	0.673
rs11001963	T	0.55	0.34	0.01	0.005	0.0389	0.35	-0.013	0.004	<5E-08
rs11047132	G	0.92	0.78	-0.002	0.005	0.656	0.71	0.007	0.004	0.078
rs1106761	A	0.37	0.16	0	0.006	0.98	0.14	-0.017	0.005	0.001
rs1106908	G	0.45	0.33	-0.016	0.004	0.000402	0.41	0.014	0.004	<5E-08
rs11074446	T	0.85	0.73	0.035	0.005	1.10E-13	0.72	-0.03	0.005	<5E-08
rs11075489	C	0.47	0.55	-0.009	0.004	0.0437	0.58	0.003	0.004	0.42
rs11105839	T	0.37	0.5	-0.019	0.004	2.24E-05	0.48	0.013	0.004	<5E-08
rs11115176	T	0.78	0.79	0.001	0.005	0.824	0.84	-0.001	0.005	0.888
rs11121210	C	0.34	0.17	-0.003	0.006	0.57	0.16	0.01	0.005	0.039
rs11126822	A	0.31	0.16	0.012	0.006	0.0359	0.2	-0.002	0.004	0.685
rs11138313	A	0.9	0.99	-0.009	0.053	0.865		Low MAF*		
rs11158434	A	0.2	0.42	0.003	0.004	0.485	0.55	-0.003	0.004	0.331
rs11170468	A	0.78	0.99	0.026	0.021	0.204		Low MAF*		
rs11181001	A	0.48	0.41	0.002	0.004	0.629	0.4	-0.008	0.004	0.029
rs11185111	G	0.29	0.47	-0.004	0.004	0.406	0.5	0.008	0.004	0.018
rs1119950	G	0.57	0.3	-0.015	0.005	0.00121	0.45	0.006	0.004	0.12
rs11218510	G	0.39	0.28	-0.006	0.005	0.233	0.26	0.008	0.004	0.049
rs112566467	T	0.21	0.19	0.012	0.005	0.0207	0.17	-0.008	0.005	0.12
rs113397893	G	0.92	0.99	-0.144	0.096	0.131		Low MAF*		
rs11525873	T	0.9	0.75	0.02	0.005	6.56E-05	0.64	-0.013	0.004	0.001
rs11577094	T	0.08	0.01	0.009	0.024	0.697		Low MAF*		
rs11608710	G	0.94	0.99	-0.329	0.141	0.0191		Low MAF*		
rs11611246	T	0.2	0.33	0.014	0.005	0.0016	0.3	-0.002	0.004	0.565
rs11629783	C	0.77	0.86	0.009	0.006	0.146	0.93	-0.026	0.007	<5E-08
rs11633626	C	0.63	0.68	-0.007	0.004	0.107	0.72	0.006	0.004	0.101
rs11636611	T	0.5	0.8	0.007	0.005	0.193	0.82	-0.003	0.005	0.482
rs11638950	A	0.59	0.71	0.008	0.005	0.0818	0.71	-0.006	0.004	0.137
rs11649864	A	0.09	0.01	0.037	0.031	0.229		Low MAF*		
rs11655587	C	0.36	0.17	-0.011	0.006	0.0537	0.17	0.014	0.005	0.005
rs11672660	C	0.19	0.19	-0.024	0.005	6.43E-06	0.23	0.025	0.004	<5E-08
rs1167821	T	0.55	0.95	-0.02	0.01	0.0408		Low MAF*		

rs11692326	T	0.23	0.11	0.022	0.007	0.00125	0.16	-0.016	0.005	0.001
rs11702843	A	0.25	0.03	0.008	0.012	0.52	0.03	-0.004	0.011	0.753
rs11739877	T	0.62	0.55	-0.003	0.004	0.415	0.6	-0.001	0.004	0.758
rs11757278	T	0.7	0.38	0.011	0.004	0.0105	0.49	-0.009	0.004	0.008
rs11761528	C	0.09	0.0001	-0.033	0.059	0.571		Low MAF*		
rs11781222	T	0.85	0.72	0.001	0.005	0.805	0.7	-0.008	0.004	0.038
rs11836108	A	0.3	0.19	0.01	0.005	0.0655	0.17	-0.011	0.005	0.027
rs11856579	G	0.26	0.03	-0.006	0.013	0.644	0.01	0.017	0.018	0.337
rs11866815	C	0.26	0.21	-0.011	0.005	0.0314	0.2	0	0.004	0.963
rs1187352	C	0.34	0.18	-0.013	0.005	0.0188	0.17	0.01	0.005	0.039
rs11874040	A	0.71	0.49	0.006	0.004	0.136	0.57	-0.008	0.004	0.025
rs11915371	C	0.8	0.93	0.009	0.008	0.245	0.97	0.006	0.01	0.567
rs11931941	A	0.22	0.39	0.011	0.004	0.015	0.36	0.0001	0.004	0.934
rs11971098	G	0.91	0.99	-0.022	0.032	0.496		Low MAF*		
rs1199334	A	0.19	0.03	0.027	0.012	0.0308	0.04	-0.024	0.009	0.01
rs11997238	G	0.91	0.83	-0.011	0.006	0.0532	0.84	0.012	0.005	0.012
rs12022461	G	0.17	0.21	-0.015	0.005	0.00347	0.15	0.013	0.005	0.008
rs12049202	T	0.19	0.36	-0.005	0.004	0.293	0.39	-0.005	0.004	0.212
rs12072739	G	0.78	0.92	0.009	0.007	0.241	0.97	0.013	0.01	0.205
rs12098284	T	0.12	0.13	-0.004	0.006	0.499	0.12	-0.004	0.006	0.468
rs12121950	T	0.34	0.76	0.009	0.005	0.0652	0.65	-0.013	0.004	0.001
rs12147845	T	0.11	0.0001	0.006	0.039	0.875		Low MAF*		
rs12151152	G	0.41	0.31	-0.027	0.005	9.49E-09		Low MAF*		
rs12193797	A	0.87	0.99	-0.017	0.022	0.431		Low MAF*		
rs12206094	C	0.29	0.2	-0.02	0.005	0.000148	0.17	0.015	0.005	0.002
rs12209887	A	0.46	0.29	0.019	0.005	4.50E-05	0.27	-0.004	0.004	0.359
rs12222235	C	0.33	0.16	-0.014	0.006	0.0182	0.09	0.002	0.006	0.725
rs12238336	C	0.38	0.43	-0.004	0.004	0.299	0.34	0.001	0.004	0.703
rs12282785	C	0.22	0.17	0.003	0.006	0.615	0.15	0.004	0.005	0.427
rs12286929	G	0.5	0.73	-0.01	0.005	0.027	0.8	0.015	0.005	0.001
rs12316047	G	0.24	0.01	-0.057	0.05	0.252		Low MAF*		
rs12321904	T	0.5	0.24	0	0.005	0.978	0.23	-0.005	0.004	0.221

rs12336441	A	0.62	0.99	-0.008	0.027	0.775		Low MAF*		
rs12364470	G	0.85	0.99	-0.049	0.027	0.0646		Low MAF*		
rs12422552	G	0.27	0.3	0.001	0.005	0.812	0.24	-0.006	0.004	0.152
rs12429545	A	0.12	0.25	0.034	0.005	1.97E-12	0.22	-0.031	0.004	<5E-08
rs12431682	C	0.34	0.46	-0.003	0.004	0.501	0.49	0.009	0.004	0.014
rs12438629	C	0.97	0.87	0.024	0.006	0.000127	0.85	-0.018	0.005	<5E-08
rs12439798	T	0.44	0.3	0.007	0.005	0.157	0.3	-0.009	0.004	0.023
rs12446632	G	0.14	0.0001	-0.123	0.071	0.0834		Low MAF*		
rs12448257	A	0.22	0.28	0.029	0.005	1.29E-09	0.3	-0.023	0.004	<5E-08
rs12449219	G	0.86	0.92	-0.022	0.008	0.00469	0.88	0.007	0.006	0.21
rs12449442	A	0.22	0.61	0.019	0.004	9.08E-06	0.7	-0.021	0.004	<5E-08
rs12454712	C	0.62	0.55	-0.016	0.004	0.000168	0.52	0.019	0.004	<5E-08
rs12462975	A	0.32	0.19	0.004	0.006	0.437	0.23	-0.009	0.004	0.037
rs12470698	A	0.57	0.8	0.01	0.005	0.0636	0.77	-0.011	0.004	0.016
rs12488237	C	0.94	0.94	0.003	0.008	0.737	0.93	0.012	0.007	0.1
rs12509234	C	0.72	0.63	-0.009	0.004	0.0377	0.94	0.0001	0.00371	0.009134
rs12522567	T	0.49	0.17	0.007	0.006	0.236	0.15	-0.007	0.005	0.165
rs12546331	T	0.51	0.6	0.005	0.004	0.267	0.63	-0.014	0.004	<5E-08
rs12551906	G	0.29	0.09	-0.001	0.007	0.838	0.1	0.01	0.006	0.097
rs12628051	T	0.64	0.6	0.018	0.004	3.23E-05	0.53	-0.024	0.004	<5E-08
rs12628891	C	0.32	0.46	-0.003	0.004	0.514	0.41	0.002	0.004	0.613
rs12635553	A	0.49	0.92	0.007	0.008	0.399	0.85	-0.003	0.005	0.563
rs1263627	T	0.76	0.59	-0.008	0.004	0.0599	0.68	0.006	0.004	0.114
rs12651833	C	0.81	0.57	0.002	0.004	0.658	0.42	-0.007	0.004	0.046
rs12680842	A	0.68	0.51	0.013	0.004	0.00312	0.5	-0.019	0.004	<5E-08
rs12681792	A	0.2	0.28	0.007	0.005	0.151	0.31	-0.001	0.004	0.759
rs1269175	A	0.51	0.86	0.011	0.006	0.0873	0.81	-0.009	0.005	0.047
rs12692596	T	0.36	0.26	-0.003	0.005	0.519	0.34	0.003	0.004	0.374
rs12705916	C	0.62	0.71	-0.005	0.005	0.277	0.69	0.007	0.004	0.086
rs12714199	C	0.61	0.79	-0.01	0.005	0.0423	0.67	0.004	0.004	0.334
rs12765914	T	0.08	0.25	0.016	0.005	0.00149	0.18	-0.016	0.005	0.001
rs1277733	T	0.77	0.76	0.014	0.005	0.00425	0.66	-0.017	0.004	<5E-08

rs1285245	G	0.39	0.34	-0.014	0.004	0.0015	0.33	0.018	0.004	<5E-08
rs12868881	A	0.41	0.41	0.013	0.004	0.00259	0.39	-0.003	0.004	0.352
rs12885454	C	0.36	0.56	-0.013	0.004	0.0019	0.52	0.001	0.004	0.848
rs12889085	G	0.58	0.81	-0.009	0.005	0.0993	0.83	0.007	0.005	0.149
rs12890931	G	0.63	0.36	-0.002	0.004	0.572	0.38	0.002	0.004	0.646
rs12899905	C	0.26	0.04	-0.017	0.011	0.115	0.03	0.027	0.011	0.01
rs12912380	C	0.11	0.05	-0.014	0.01	0.165	0.03	0.014	0.011	0.196
rs12914623	G	0.27	0.14	-0.002	0.006	0.767	0.14	0.012	0.006	0.035
rs12922346	C	0.26	0.16	0.014	0.006	0.0153	0.2	0.014	0.005	0.003
rs12939549	A	0.56	0.7	0.02	0.005	1.39E-05	0.65	-0.008	0.004	0.041
rs13002946	T	0.27	0.55	0	0.004	0.915	0.46	0.002	0.004	0.59
rs13021737	G	0.16	0.09	-0.082	0.007	4.38E-28	0.1	0.049	0.006	<5E-08
rs13033310	A	0.25	0.31	0.007	0.005	0.139	0.31	0.004	0.004	0.241
rs13062093	G	0.63	0.85	0.005	0.006	0.442	0.85	-0.003	0.005	0.573
rs13110266	G	0.4	0.51	0.001	0.004	0.771	0.45	0.0001	0.004	0.975
rs13155259	G	0.67	0.9	0.007	0.007	0.332	0.92	0.0001	0.007	0.962
rs13174863	G	0.85	0.95	-0.003	0.01	0.794	0.94	0.025	0.008	0.001
rs13191362	A	0.86	0.99	0.017	0.056	0.768		Low MAF*		
rs1320251	C	0.46	0.58	-0.012	0.004	0.00743	0.66	0.013	0.004	0.001
rs13209872	G	0.35	0.45	0	0.004	0.995	0.36	0.009	0.004	0.018
rs13264909	A	0.57	0.34	0	0.004	0.955	0.28	-0.001	0.004	0.89
rs13298062	A	0.81	0.99	0.031	0.02	0.12	0.94	-0.008	0.007	0.288
rs13299788	C	0.85	0.83	0.002	0.006	0.774	0.8	-0.015	0.004	<5E-08
rs13303252	T	0.18	0.4	0.004	0.004	0.316	0.36	0.003	0.004	0.403
rs13417156	C	0.42	0.34	-0.007	0.004	0.139	0.29	0.005	0.004	0.172
rs13432055	C	0.71	0.95	-0.009	0.01	0.352	0.95	0.017	0.009	0.053
rs1346841	G	0.41	0.55	-0.012	0.004	0.008	0.6	0.007	0.004	0.07
rs1355459	A	0.32	0.09	-0.002	0.008	0.783	0.04	-0.01	0.009	0.23
rs1356506	T	0.63	0.5	0.019	0.004	9.61E-06	0.61	-0.017	0.004	<5E-08
rs1363695	C	0.24	0.21	-0.006	0.005	0.241	0.16	0.0001	0.005	0.993
rs1383592	A	0.21	0.22	0.005	0.005	0.377	0.14	-0.004	0.005	0.426
rs1394879	C	0.4	0.12	0.006	0.006	0.385	0.05	-0.016	0.008	0.042

rs1409818	T	0.11	0.15	0.011	0.006	0.0735	0.13	-0.011	0.005	0.036
rs1411431	A	0.16	0.27	0.011	0.005	0.0211	0.35	-0.016	0.004	<5E-08
rs1417665	T	0.8	0.9	0.005	0.007	0.506	0.95	0.002	0.008	0.839
rs1426652	A	0.17	0.31	0.021	0.005	6.92E-06	0.32	-0.004	0.004	0.277
rs1431659	A	0.27	0.31	0.001	0.005	0.893	0.29	-0.009	0.004	0.026
rs1436344	C	0.57	0.36	0.004	0.004	0.337	0.35	-0.011	0.004	0.004
rs1437842	G	0.49	0.31	-0.002	0.005	0.6	0.33	0.011	0.004	0.003
rs1451077	G	0.42	0.41	-0.007	0.004	0.118	0.32	0.006	0.004	0.122
rs1451109	G	0.68	0.54	-0.002	0.004	0.641	0.47	0.002	0.004	0.592
rs1452075	T	0.73	0.71	0.007	0.005	0.119	0.8	0.001	0.004	0.289
rs1460676	C	0.82	0.62	-0.005	0.004	0.23	0.6	0.009	0.004	0.015
rs1470545	T	0.04	0.01	0.037	0.038	0.327		Low MAF*		
rs1471212	A	0.47	0.91	-0.003	0.007	0.652	0.96	-0.007	0.009	0.44
rs1477890	G	0.49	0.5	-0.004	0.004	0.318	0.47	0.007	0.004	0.044
rs1481012	A	0.89	0.71	0.012	0.005	0.0109	0.7	-0.012	0.004	0.002
rs1491905	T	0.53	0.74	0.011	0.005	0.0212	0.7	-0.011	0.004	0.005
rs1501673	A	0.14	0.49	0.023	0.004	4.18E-08	0.44	-0.02	0.004	<5E-08
rs1506662	A	0.53	0.62	0.005	0.004	0.26	0.66	-0.002	0.004	0.658
rs1514177	C	0.43	0.77	0.032	0.005	1.01E-10	0.83	-0.017	0.005	<5E-08
rs1522569	T	0.82	0.99	0.037	0.032	0.256		Low MAF*		
rs1523768	G	0.67	0.83	-0.011	0.006	0.0556	0.82	-0.002	0.005	0.675
rs1536053	C	0.31	0.05	0.016	0.01	0.106	0.02	0.018	0.011	0.108
rs1541777	G	0.54	0.85	-0.004	0.006	0.485	0.88	0.008	0.005	0.145
rs1546924	T	0.5	0.34	0.002	0.005	0.695	0.38	-0.007	0.004	0.079
rs1554194	C	0.5	0.53	0.023	0.004	6.27E-08	0.5	0.009	0.004	0.019
rs1561554	G	0.61	0.92	-0.006	0.007	0.416	0.94	0.004	0.008	0.593
rs1580099	C	0.58	0.68	-0.013	0.005	0.00305	0.74	0.014	0.004	0.001
rs1582931	G	0.47	0.6	-0.022	0.004	4.66E-07	0.53	0.017	0.004	0.511
rs159032	T	0.23	0.13	0.004	0.006	0.542	0.18	0.005	0.005	0.303
rs1601817	C	0.31	0.16	-0.003	0.006	0.563	0.13	0.011	0.005	0.034
rs1634350	A	0.44	0.7	0.016	0.005	0.000756	0.64	-0.008	0.004	0.039
rs1635853	G	0.41	0.33	-0.01	0.004	0.0227	0.3	-0.003	0.004	0.502

rs1658820	T	0.24	0.38	0.014	0.004	0.00226	0.29	-0.013	0.004	0.001
rs16834431	T	0.21	0.01	-0.019	0.03	0.518		Low MAF*		
rs16851483	T	0.07	0.24	0.025	0.005	4.90E-07	0.29	-0.016	0.004	<5E-08
rs16882001	G	0.95	0.96	-0.021	0.01	0.0317	0.98	0.006	0.011	0.606
rs16906845	G	0.06	0.12	-0.018	0.006	0.0043	0.15	0.016	0.005	0.002
rs16907751	C	0.09	0.19	-0.016	0.005	0.00238	0.14	0.028	0.005	<5E-08
rs16940823	C	0.17	0.09	0.001	0.007	0.845	0.09	-0.001	0.006	0.836
rs16978350	G	0.28	0.11	0.008	0.007	0.221	0.12	0.01	0.005	0.066
rs16989232	A	0.39	0.52	-0.001	0.004	0.823	0.42	-0.002	0.004	0.509
rs17001561	A	0.15	0.02	0.003	0.014	0.82	0.01	0.008	0.018	0.644
rs17014375	G	0.87	0.87	-0.006	0.006	0.295	0.88	0.008	0.006	0.158
rs17019336	T	0.23	0.35	-0.01	0.004	0.0215	0.27	0.011	0.004	0.006
rs1707322	G	0.32	0.3	-0.014	0.005	0.0019	0.32	0.004	0.004	0.354
rs17105272	T	0.32	0.47	0.011	0.004	0.0109	0.43	-0.011	0.004	0.003
rs17193211	C	0.07	0.07	-0.018	0.008	0.0326	0.07	0.013	0.008	0.092
rs17201143	C	0.31	0.48	-0.004	0.004	0.411	0.44	0.013	0.004	<5E-08
rs1721447	G	0.51	0.22	-0.001	0.005	0.848	0.32	0.002	0.004	0.553
rs17276464	T	0.41	0.02	-0.005	0.016	0.751		Low MAF*		
rs1730859	G	0.65	0.74	-0.004	0.005	0.451	0.72	0.006	0.004	0.158
rs17405819	T	0.68	0.55	0.023	0.004	6.40E-08	0.39	-0.021	0.004	<5E-08
rs17425707	C	0.9	0.99	-0.03	0.043	0.489		Low MAF*		
rs17448885	C	0.65	0.57	0.012	0.004	0.00672	0.62	-0.006	0.004	0.096
rs17522122	T	0.47	0.36	0.012	0.004	0.00555	0.35	-0.01	0.004	0.014
rs17535749	A	0.11	0.001	0.075	0.068	0.27		Low MAF*		
rs17551974	C	0.19	0.19	-0.013	0.005	0.0141	0.19	0.008	0.005	0.069
rs17574378	T	0.73	0.85	0.009	0.006	0.138	0.84	-0.007	0.005	0.122
rs17609108	C	0.85	0.85	-0.011	0.006	0.0552	0.81	-0.011	0.005	0.019
rs17636031	C	0.72	0.98	-0.018	0.019	0.35		Low MAF*		
rs17681451	G	0.08	0.01	0.032	0.029	0.262		Low MAF*		
rs17720922	T	0.19	0.08	0.001	0.008	0.857	0.11	0.002	0.006	0.773
rs17757975	T	0.85	0.99	0.006	0.028	0.836		Low MAF*		
rs17806224	G	0.18	0.15	-0.012	0.006	0.0479	0.14	0.011	0.005	0.033

rs1819844	A	0.17	0.28	0.019	0.005	8.24E-05	0.19	-0.013	0.005	0.006
rs1852006	G	0.38	0.44	-0.028	0.004	6.50E-11	0.43	0.014	0.004	<5E-08
rs1871329	G	0.76	0.71	-0.008	0.005	0.106	0.68	-0.006	0.004	0.136
rs1876359	T	0.37	0.35	0	0.004	0.951	0.42	-0.007	0.004	0.044
rs1877875	C	0.43	0.3	-0.009	0.005	0.0534	0.34	0.002	0.004	0.64
rs1884389	C	0.44	0.8	0.015	0.005	0.00413	0.67	0.01	0.004	0.008
rs1884429	T	0.23	0.07	0.019	0.008	0.0258	0.06	-0.011	0.008	0.163
rs1884897	G	0.37	0.14	-0.021	0.006	0.000737	0.09	0.014	0.006	0.024
rs1891215	C	0.54	0.66	0.004	0.004	0.426	0.53	0.003	0.004	0.491
rs1899689	T	0.39	0.5	0.01	0.004	0.0141	0.41	-0.01	0.004	0.005
rs1911746	T	0.21	0.26	-0.006	0.005	0.246	0.34	-0.008	0.004	0.045
rs1916801	A	0.62	0.43	0.003	0.004	0.491	0.31	-0.011	0.004	0.009
rs1927790	C	0.61	0.66	-0.016	0.004	0.000294	0.58	0.01	0.004	0.005
rs1928295	T	0.57	0.63	0.012	0.004	0.00771	0.56	-0.015	0.004	<5E-08
rs1941697	A	0.45	0.8	0.002	0.005	0.765	0.76	-0.013	0.004	0.001
rs194809	A	0.19	0.27	0.004	0.005	0.406	0.19	-0.012	0.005	0.009
rs1951455	C	0.28	0.11	-0.01	0.007	0.167	0.12	0.004	0.007	0.56
rs1954494	T	0.56	0.48	0.007	0.004	0.129	0.49	-0.003	0.004	0.343
rs1967772	G	0.27	0.06	-0.002	0.009	0.837	0.03	0.031	0.01	0.002
rs1982350	G	0.64	0.62	0.004	0.005	0.354	0.67	0.007	0.004	0.065
rs1982441	T	0.13	0.0001	0.069	0.042	0.0993		Low MAF*		
rs1987960	C	0.05	0.0001	-0.304	0.155	0.0499		Low MAF*		
rs1990573	G	0.31	0.34	0.005	0.005	0.255	0.46	0.005	0.004	0.203
rs1996120	G	0.59	0.65	-0.03	0.004	2.38E-11	0.65	0.007	0.004	0.066
rs2012502	A	0.38	0.18	0.013	0.006	0.0171	0.19	-0.013	0.005	0.006
rs2033529	G	0.73	0.81	-0.006	0.005	0.266	0.7	0.011	0.004	0.006
rs2044469	G	0.66	0.77	-0.006	0.005	0.218	0.73	0.004	0.004	0.281
rs2051559	C	0.86	0.85	-0.003	0.006	0.554	0.8	-0.001	0.004	0.831
rs2053682	A	0.68	0.83	0.008	0.006	0.132	0.91	-0.011	0.006	0.07
rs2058527	G	0.27	0.28	-0.014	0.005	0.00316	0.33	0.003	0.004	0.49
rs2063177	A	0.35	0.11	-0.003	0.007	0.64	0.12	-0.001	0.005	0.873
rs2065418	T	0.65	0.63	0.016	0.004	0.000425	0.64	-0.012	0.004	0.001

rs2070929	C	0.29	0.51	0.007	0.004	0.0876	0.55	-0.002	0.004	0.607
rs2072518	A	0.57	0.46	0.004	0.004	0.398	0.51	0.0001	0.004	0.911
rs2072597	G	0.31	0.39	-0.01	0.004	0.016	0.29	0.004	0.004	0.344
rs2074881	C	0.19	0.28	-0.014	0.005	0.00408	0.29	0.007	0.005	0.104
rs2081493	C	0.3	0.19	-0.003	0.005	0.531	0.26	0.003	0.004	0.438
rs2105808	C	0.67	0.97	-0.029	0.012	0.0147	0.95	0.006	0.008	0.434
rs2107118	A	0.5	0.48	0.012	0.004	0.00443	0.57	-0.011	0.004	0.004
rs2112347	T	0.63	0.43	0.02	0.004	2.73E-06	0.44	-0.024	0.004	<5E-08
rs2134858	C	0.51	0.42	-0.005	0.004	0.26	0.47	0.01	0.004	0.004
rs2155645	C	0.26	0.38	-0.01	0.004	0.028	0.35	0.007	0.004	0.054
rs215614	G	0.38	0.39	0.002	0.004	0.573	0.34	0.011	0.004	0.002
rs2170382	T	0.11	0.0001	0.053	0.029	0.0674		Low MAF*		
rs217669	C	0.72	0.57	-0.005	0.004	0.236	0.61	0.001	0.004	0.775
rs2178899	A	0.88	0.98	-0.012	0.014	0.402	0.99	0.009	0.017	0.587
rs2183824	T	0.31	0.3	0.002	0.005	0.71	0.27	0.001	0.004	0.843
rs2187449	A	0.77	0.93	0.001	0.008	0.862	0.93	-0.008	0.007	0.266
rs2192158	A	0.55	0.6	-0.003	0.004	0.549	0.63	-0.004	0.004	0.325
rs2196618	G	0.26	0.38	-0.007	0.004	0.0972	0.29	0.011	0.004	0.017
rs2206277	T	0.16	0.23	0.037	0.005	1.60E-13	0.02	-0.029	0.015	0.873
rs2228213	G	0.34	0.23	-0.017	0.005	0.000684	0.27	0.017	0.004	<5E-08
rs223058	G	0.33	0.04	-0.009	0.011	0.424	0.02	0.002	0.014	0.916
rs2237403	C	0.64	0.55	-0.001	0.004	0.755	0.52	0.009	0.004	0.014
rs2241743	G	0.43	0.36	-0.003	0.004	0.532	0.35	0.005	0.004	0.188
rs2246012	C	0.85	0.65	-0.003	0.004	0.549	0.65	0.006	0.004	0.099
rs2299383	T	0.4	0.45	0.016	0.004	0.000154	0.38	-0.01	0.004	0.009
rs2307022	A	0.33	0.14	0.013	0.006	0.0385	0.12	-0.015	0.006	0.008
rs2322622	C	0.35	0.14	0.007	0.006	0.228	0.08	0.008	0.005	0.09
rs2342892	T	0.49	0.58	0.021	0.004	1.04E-06	0.63	-0.015	0.004	<5E-08
rs2357760	A	0.67	0.76	0.014	0.005	0.00632	0.69	-0.012	0.004	0.002
rs2391540	T	0.67	0.79	0.008	0.005	0.148	0.83	0.006	0.005	0.22
rs2396625	T	0.58	0.63	-0.01	0.004	0.028	0.63	-0.002	0.004	0.646
rs2400414	C	0.35	0.25	-0.018	0.005	0.000217	0.22	0.013	0.004	0.002

rs2425857	A	0.43	0.38	0.014	0.004	0.00138	0.36	-0.005	0.004	0.174
rs2434467	T	0.34	0.27	0.014	0.005	0.0047	0.35	-0.013	0.004	<5E-08
rs2439823	G	0.45	0.74	-0.004	0.005	0.46	0.71	0.003	0.004	0.438
rs2447832	T	0.44	0.33	0.007	0.005	0.12	0.37	-0.004	0.004	0.276
rs2450448	A	0.69	0.93	0.003	0.008	0.668	0.92	0.0001	0.007	0.954
rs2455793	A	0.42	0.63	-0.005	0.004	0.291	0.61	-0.005	0.004	0.199
rs2470397	C	0.81	0.99	-0.002	0.022	0.937		Low MAF*		
rs249293	C	0.7	0.43	0.003	0.004	0.525	0.5	-0.003	0.004	0.42
rs249612	T	0.71	0.46	0.009	0.004	0.0426	0.42	-0.005	0.004	0.151
rs2577947	C	0.82	0.88	-0.006	0.006	0.357	0.89	0.013	0.006	0.027
rs2596902	G	0.35	0.39	-0.014	0.004	0.00146	0.41	0.005	0.004	0.15
rs2601777	G	0.39	0.44	-0.019	0.004	9.37E-06	0.47	0.009	0.004	0.011
rs2605603	G	0.48	0.87	-0.009	0.006	0.158	0.85	0.007	0.005	0.178
rs2619976	T	0.4	0.27	0.004	0.005	0.412	0.23	-0.004	0.004	0.341
rs2622274	G	0.46	0.43	-0.016	0.004	0.000269	0.41	0.012	0.004	0.001
rs264962	C	0.54	0.35	-0.01	0.004	0.0288	0.28	-0.001	0.004	0.795
rs2670854	A	0.72	0.83	0.012	0.006	0.0388	0.86	-0.003	0.005	0.5
rs2682406	T	0.59	0.64	-0.013	0.004	0.00265	0.61	0.01	0.004	0.006
rs2693686	G	0.61	0.74	0	0.005	0.999	0.8	-0.005	0.004	0.275
rs2715423	G	0.28	0.06	0	0.009	0.991	0.04	-0.007	0.009	0.45
rs273505	C	0.58	0.66	-0.004	0.004	0.431	0.7	0.012	0.004	0.004
rs2777768	A	0.72	0.96	0.004	0.01	0.677	0.98	-0.019	0.012	0.12
rs2803316	G	0.46	0.39	-0.003	0.004	0.455	0.41	0.007	0.004	0.057
rs2820295	A	0.33	0.12	0.006	0.007	0.394	0.1	-0.021	0.006	<5E-08
rs2832283	A	0.23	0.05	0.003	0.01	0.772	0.04	-0.012	0.01	0.07
rs28350	A	0.17	0.08	0.017	0.008	0.0343	0.08	-0.015	0.007	0.022
rs2837398	C	0.61	0.47	-0.011	0.004	0.00753	0.47	-0.001	0.004	0.849
rs284227	C	0.73	0.95	-0.021	0.009	0.0274	0.95	0.005	0.008	0.548
rs2861089	A	0.38	0.1	0.007	0.007	0.324	0.1	-0.006	0.006	0.287
rs2861685	T	0.59	0.41	0.006	0.004	0.166	0.37	-0.01	0.004	0.006
rs2862996	G	0.7	0.84	-0.003	0.006	0.6	0.86	0.003	0.005	0.597
rs2870710	C	0.81	0.74	0.02	0.005	2.99E-05	0.68	-0.013	0.004	0.001

rs288230	T	0.84	0.84	0.006	0.006	0.304		0.85	-0.003	0.005	0.597
rs2899663	G	0.49	0.57	0.002	0.004	0.712		0.43	0.004	0.004	0.264
rs2907948	G	0.24	0.05	-0.006	0.01	0.571		0.04	0.009	0.009	0.336
rs2933451	C	0.67	0.75	-0.005	0.005	0.32		0.74	0.008	0.004	0.062
rs2954021	G	0.51	0.43	0.005	0.004	0.238		0.45	0.002	0.004	0.564
rs2968487	T	0.28	0.41	0.01	0.004	0.024		0.43	-0.006	0.004	0.085
rs29938	C	0.66	0.76	-0.027	0.005	7.52E-08		0.79	0.014	0.004	0.001
rs3101336	C	0.38	0.08	-0.022	0.008	0.00379		0.07	0.025	0.007	<5E-08
rs3134358	T	0.62	0.64	0.006	0.004	0.202		0.56	-0.003	0.004	0.366
rs325220	G	0.3	0.06	-0.007	0.009	0.476		0.11	0.014	0.006	0.016
rs326845	G	0.38	0.48	-0.001	0.004	0.872		0.5	-0.003	0.004	0.38
rs329124	A	0.59	0.6	0.018	0.004	3.21E-05		0.63	-0.019	0.004	<5E-08
rs340025	C	0.41	0.27	-0.01	0.005	0.0466		0.17	0.004	0.005	0.38
rs34277166	A	0.44	0.11	0.011	0.007	0.117		0.08	-0.015	0.006	0.017
rs34720381	T	0.09	0.08	0.006	0.008	0.433		0.07	-0.008	0.007	0.265
rs34801745	C	0.36	0.84	0.002	0.006	0.776		0.84	0.001	0.005	0.836
rs349088	C	0.48	0.34	-0.001	0.005	0.767		0.44	0.004	0.004	0.257
rs354508	C	0.16	0.21	-0.008	0.005	0.113		0.2	0.003	0.004	0.488
rs355777	C	0.4	0.43	0.007	0.004	0.11		0.53	-0.009	0.004	0.008
rs355914	C	0.63	0.52	0.001	0.004	0.868		0.47	-0.007	0.004	0.05
rs35809007	G	0.36	0.48	-0.001	0.004	0.877		0.55	-0.002	0.004	0.566
rs35851183	G	0.64	0.57	-0.005	0.004	0.203		0.52	0.013	0.004	<5E-08
rs36061954	T	0.4	0.33	0.019	0.005	2.45E-05		0.3	0.0001	0.004	0.989
rs3738476	C	0.88	0.63	-0.004	0.005	0.38		0.91	0.004	0.006	0.519
rs3739514	A	0.34	0.55	0.009	0.004	0.0353		0.55	-0.004	0.004	0.315
rs3744017	A	0.19	0.14	0.003	0.006	0.646		0.15	-0.001	0.005	0.864
rs3746038	C	0.22	0.41	-0.007	0.004	0.0997		0.5	0.008	0.004	0.043
rs3750944	G	0.56	0.5	-0.009	0.004	0.0287		0.63	0.004	0.004	0.291
rs3752904	C	0.51	0.46	-0.003	0.004	0.464		0.48	0.0001	0.004	0.995
rs3766160	G	0.77	0.53	-0.011	0.004	0.00805		0.52	0.003	0.004	0.438
rs3770799	G	0.64	0.78	-0.008	0.005	0.111		0.81	0.008	0.004	0.068
rs3774573	T	0.74	0.84	0.012	0.006	0.0413		0.81	-0.011	0.005	0.014

rs3806114	G	0.69	0.8	-0.004	0.006	0.493	0.76	0.003	0.004	0.45
rs3808477	C	0.27	0.3	-0.019	0.005	4.32E-05	0.27	0.015	0.004	<5E-08
rs3811125	C	0.28	0.24	-0.008	0.005	0.127	0.27	0.01	0.004	0.024
rs3822683	A	0.79	0.58	0	0.004	0.984	0.76	0.002	0.004	0.254
rs3825061	T	0.39	0.17	0.009	0.006	0.127	0.12	-0.01	0.006	0.07
rs3914628	T	0.86	0.9	0.003	0.007	0.696	0.1	-0.007	0.005	0.218
rs3922980	G	0.5	0.84	0.001	0.006	0.875		Low MAF*		
rs3923783	C	0.18	0.13	-0.005	0.006	0.405	0.11	0.008	0.006	0.172
rs3930349	C	0.21	0.29	-0.011	0.005	0.0235	0.36	0.006	0.004	0.113
rs3935190	G	0.54	0.69	-0.016	0.005	0.000684	0.6	0.015	0.004	<5E-08
rs39654	G	0.45	0.4	-0.009	0.004	0.0363	0.41	0.009	0.004	0.015
rs4097319	T	0.56	0.42	0.001	0.004	0.798	0.41	-0.002	0.004	0.652
rs4123853	T	0.37	0.25	0.003	0.005	0.55	0.18	-0.011	0.005	0.018
rs41290587	T	0.01	0.0001	0.085	0.192	0.659		Low MAF*		
rs4239020	T	0.68	0.93	-0.005	0.008	0.588	0.95	0.01	0.008	0.251
rs4242244	T	0.58	0.37	0.014	0.004	0.00218	0.28	-0.007	0.004	0.092
rs4256980	G	0.67	0.62	-0.022	0.004	2.91E-07	0.62	0.019	0.004	<5E-08
rs4270551	A	0.13	0.003	0.086	0.05	0.0864		Low MAF*		
rs4273371	C	0.51	0.82	-0.01	0.005	0.0573	0.79	0.001	0.004	0.787
rs427943	C	0.43	0.49	-0.014	0.004	0.00113	0.56	0.01	0.004	0.008
rs4290163	T	0.4	0.58	0.015	0.004	0.000442	0.46	-0.017	0.004	<5E-08
rs4307239	G	0.46	0.3	-0.003	0.005	0.544	0.19	0.005	0.005	0.231
rs4372836	T	0.33	0.27	0.013	0.005	0.0085	0.32	-0.007	0.004	0.065
rs4382152	T	0.62	0.2	0.01	0.005	0.068	0.19	-0.01	0.004	0.011
rs4414033	A	0.6	0.69	0.006	0.005	0.19	0.69	-0.014	0.004	<5E-08
rs4421883	C	0.49	0.67	0.002	0.005	0.733	0.74	0.002	0.004	0.683
rs4581940	T	0.59	0.75	0.02	0.005	3.22E-05	0.75	-0.002	0.004	0.632
rs459552	T	0.77	0.91	-0.001	0.007	0.895	0.9	0.0001	0.006	0.969
rs4609871	T	0.54	0.4	0.014	0.004	0.00112	0.31	-0.012	0.004	0.001
rs4671328	T	0.45	0.38	0.02	0.004	4.96E-06	0.41	-0.02	0.004	<5E-08
rs4700608	C	0.48	0.15	-0.015	0.006	0.0147	0.15	0.004	0.005	0.41
rs4703019	A	0.39	0.4	-0.001	0.004	0.738	0.35	-0.007	0.004	0.044

rs4721089	T	0.78	0.89	0.008	0.007	0.231	0.88	0.005	0.006	0.399
rs4721823	G	0.14	0.44	-0.012	0.004	0.00404	0.51	0.004	0.004	0.241
rs4722398	T	0.15	0.02	0.012	0.014	0.418	0.06	-0.006	0.007	0.408
rs4739570	A	0.62	0.59	0.01	0.004	0.021	0.54	-0.013	0.004	<5E-08
rs4740619	T	0.54	0.24	0.015	0.005	0.00199	0.23	-0.012	0.004	0.005
rs4746339	T	0.26	0.03	0.01	0.012	0.417	0.04	-0.026	0.009	0.004
rs4794977	C	0.57	0.76	-0.002	0.005	0.709	0.74	-0.002	0.004	0.682
rs4795195	A	0.29	0.41	0	0.004	0.983	0.37	-0.004	0.004	0.259
rs4802570	C	0.4	0.75	-0.001	0.005	0.88	0.75	-0.009	0.004	0.029
rs4814512	A	0.81	0.8	0.012	0.005	0.0278	0.71	-0.006	0.004	0.117
rs4818226	G	0.31	0.09	0.011	0.008	0.144	0.08	-0.004	0.007	0.522
rs4834272	C	0.69	0.46	-0.01	0.004	0.0236	0.53	0.014	0.004	<5E-08
rs4841504	C	0.5	0.04	-0.014	0.011	0.221	0.03	-0.014	0.011	0.223
rs4851057	T	0.13	0.03	0.011	0.013	0.389	0.02	0.002	0.014	0.877
rs4857968	G	0.29	0.04	-0.018	0.012	0.118	0.03	0.008	0.011	0.473
rs487152	A	0.5	0.33	0.012	0.005	0.0072	0.29	-0.02	0.004	<5E-08
rs4880341	C	0.57	0.94	-0.01	0.008	0.234	0.92	0.0001	0.01	0.39
rs4895231	C	0.47	0.2	-0.005	0.006	0.398	0.13	0.0001	0.00555	0.4097
rs4906263	G	0.66	0.58	-0.028	0.004	4.90E-11	0.54	0.011	0.004	0.003
rs4973618	G	0.66	0.5	-0.004	0.004	0.404	0.49	0.009	0.004	0.014
rs4985155	A	0.67	0.55	0.01	0.004	0.0151	0.55	-0.016	0.004	<5E-08
rs5017416	T	0.05	0.01	0.028	0.029	0.324		Low MAF*		
rs507856	T	0.56	0.44	0.01	0.004	0.0235	0.36	-0.008	0.004	0.041
rs5215	T	0.63	0.62	0.013	0.004	0.00301	0.63	-0.018	0.004	<5E-08
rs539100	A	0.22	0.02	0.013	0.015	0.379	0.04	-0.001	0.01	0.893
rs543874	G	0.78	0.82	-0.056	0.005	3.01E-25	0.78	0.047	0.004	<5E-08
rs544722	T	0.45	0.76	0.004	0.005	0.395	0.79	-0.002	0.004	0.604
rs55676934	G	0.67	0.81	-0.005	0.005	0.349	0.81	0.004	0.005	0.416
rs559267	G	0.67	0.58	-0.015	0.004	0.000416	0.56	0.018	0.004	<5E-08
rs55966114	T	0.19	0.16	0.01	0.006	0.0938	0.14	-0.013	0.005	0.014
rs561136	C	0.87	0.92	-0.021	0.008	0.00826	0.81	0.009	0.004	0.041
rs56356382	T	0.81	0.99	0.033	0.037	0.372		Low MAF*		

rs57800857	A	0.63	0.73	-0.014	0.005	0.0036	0.76	-0.011	0.004	0.009
rs58139454	G	0.22	0.19	-0.007	0.005	0.18	0.22	0.01	0.004	0.026
rs592483	C	0.41	0.21	-0.02	0.005	0.000221	0.19	0.004	0.005	0.386
rs59302296	A	0.1	0.002	0.06	0.054	0.271		Low MAF*		
rs594821	C	0.09	0.06	-0.031	0.009	0.000535	0.12	0.01	0.006	0.075
rs6019483	T	0.17	0.08	-0.015	0.008	0.0632	0.12	0.004	0.006	0.44
rs6023633	G	0.78	0.99	0.002	0.04	0.956		Low MAF*		
rs6092179	C	0.2	0.15	-0.027	0.006	5.23E-06	0.12	0.02	0.005	<5E-08
rs61051952	A	0.75	0.62	0	0.004	0.939	0.67	-0.003	0.004	0.383
rs6130360	A	0.85	0.75	0.014	0.005	0.00417	0.8	-0.017	0.005	<5E-08
rs6142067	T	0.6	0.75	0.014	0.005	0.00411	0.65	-0.009	0.004	0.019
rs615568	G	0.52	0.9	0.012	0.007	0.0765	0.9	0.005	0.006	0.385
rs61740466	G	0.77	0.51	-0.02	0.004	3.26E-06	0.65	0.007	0.004	0.079
rs61813324	T	0.13	0.003	0.021	0.051	0.68		Low MAF*		
rs61828641	A	0.11	0.26	0.023	0.005	1.44E-06	0.21	-0.021	0.004	<5E-08
rs61903695	G	0.75	0.9	-0.008	0.007	0.247	0.89	0.013	0.006	0.017
rs62118504	G	0.61	0.98	-0.005	0.015	0.723	0.99	-0.01	0.028	0.731
rs6265	C	0.18	0.49	-0.036	0.004	1.75E-17	0.41	0.038	0.004	<5E-08
rs630602	C	0.61	0.33	0.002	0.005	0.628	0.38	0.001	0.004	0.804
rs6419734	T	0.14	0.01	-0.008	0.023	0.745		Low MAF*		
rs6435622	C	0.32	0.33	0.015	0.005	0.00138	0.48	-0.013	0.004	<5E-08
rs6443750	C	0.2	0.01	-0.039	0.048	0.416		Low MAF*		
rs6451675	G	0.32	0.18	-0.019	0.006	0.000672	0.19	0.004	0.004	0.312
rs6463489	T	0.1	0.08	0.004	0.008	0.619	0.04	-0.003	0.009	0.74
rs6476617	G	0.38	0.2	-0.01	0.005	0.0642	0.26	0.011	0.004	0.006
rs6477694	C	0.36	0.43	-0.014	0.004	0.00148	0.39	0.016	0.004	<5E-08
rs6493498	T	0.45	0.48	0.007	0.004	0.0947	0.59	0.002	0.004	0.586
rs6500208	A	0.2	0.42	0.002	0.004	0.689	0.39	-0.01	0.004	0.005
rs650198	C	0.73	0.76	0.008	0.005	0.107	0.72	0.003	0.004	0.464
rs6511027	C	0.17	0.02	-0.027	0.015	0.0708	0.01	-0.004	0.016	0.813
rs6512302	C	0.74	0.72	0.005	0.005	0.304	0.73	-0.003	0.004	0.438
rs651548	A	0.36	0.38	0.008	0.004	0.0651	0.41	0.006	0.004	0.121

rs6539064	C	0.75	0.66	0.005	0.005	0.3	0.64	-0.009	0.004	0.02
rs6540498	T	0.62	0.55	-0.002	0.004	0.61	0.42	0.003	0.004	0.473
rs6545714	G	0.61	0.64	0.008	0.004	0.0603	0.7	0.009	0.004	0.018
rs6556301	G	0.63	0.55	-0.011	0.004	0.00766	0.57	0.007	0.004	0.066
rs6561766	A	0.13	0.11	0	0.007	0.949	0.12	-0.012	0.005	0.028
rs6567160	C	0.75	0.8	-0.06	0.005	3.45E-30	0.78	0.051	0.004	<5E-08
rs6574100	A	0.59	0.9	0.011	0.007	0.123	0.9	-0.006	0.006	0.314
rs6577584	G	0.66	0.82	-0.009	0.005	0.11	0.82	0.014	0.005	0.002
rs6591407	C	0.2	0.1	-0.006	0.007	0.421	0.15	0.008	0.005	0.09
rs6602411	T	0.18	0.02	0.014	0.014	0.334	0.01	0.008	0.016	0.634
rs6604015	C	0.91	0.97	-0.016	0.011	0.155	0.98	0.019	0.014	0.163
rs6606686	G	0.7	0.99	-0.031	0.025	0.204	0.98	0.009	0.013	0.691
rs6607337	C	0.3	0.25	-0.01	0.005	0.0521	0.75	0.004	0.004	0.359
rs6661316	T	0.59	0.44	0.013	0.004	0.00322	0.42	-0.013	0.004	<5E-08
rs6690398	A	0.55	0.51	0.012	0.004	0.00364	0.44	-0.009	0.004	0.017
rs6696828	C	0.3	0.28	0.008	0.005	0.079	0.25	-0.004	0.004	0.341
rs6710871	A	0.14	0.1	0.025	0.007	0.000328	0.14	-0.011	0.005	0.034
rs6720868	T	0.31	0.23	0	0.005	0.925	0.23	-0.007	0.004	0.1
rs6733834	C	0.81	0.71	0.019	0.005	5.98E-05	0.78	-0.018	0.004	<5E-08
rs6781254	T	0.3	0.34	0.006	0.004	0.201	0.32	-0.006	0.004	0.15
rs6804181	A	0.82	0.84	0.02	0.006	0.000617	0.76	-0.016	0.004	<5E-08
rs6804842	G	0.43	0.37	-0.008	0.005	0.0782	0.38	0.013	0.004	0.001
rs6804915	A	0.29	0.25	0.02	0.005	4.50E-05	0.23	-0.011	0.004	0.012
rs6809307	T	0.26	0.36	0.011	0.004	0.0124	0.39	-0.013	0.004	<5E-08
rs6818414	C	0.52	0.69	-0.003	0.005	0.469	0.72	0.011	0.004	0.007
rs6832762	G	0.51	0.08	0.003	0.008	0.689	0.05	0.002	0.008	0.82
rs6850421	A	0.46	0.54	0.003	0.004	0.442	0.44	0.0001	0.004	0.99
rs6857	C	0.17	0.1	-0.004	0.007	0.616	0.17	0.014	0.005	0.004
rs6864049	G	0.49	0.3	-0.005	0.005	0.25	0.31	0.015	0.004	<5E-08
rs6890310	G	0.29	0.09	-0.01	0.008	0.211	0.08	0.0001	0.006	0.993
rs6905288	G	0.57	0.73	-0.012	0.005	0.0144	0.79	-0.005	0.005	0.244
rs6916553	G	0.24	0.02	-0.015	0.016	0.341	0.03	0.028	0.011	0.013

rs6922607	G	0.81	0.92	0.001	0.007	0.886	0.89	0.0001	0.006	0.979
rs6931385	A	0.48	0.4	-0.001	0.004	0.904	0.48	-0.008	0.004	0.028
rs6932930	G	0.79	0.65	-0.006	0.004	0.171	0.25	0.004	0.004	0.33
rs6934973	G	0.7	0.58	-0.006	0.004	0.138	0.68	0.003	0.004	0.356
rs6954694	G	0.84	0.63	-0.007	0.005	0.108		Low MAF*		
rs6962280	G	0.44	0.19	-0.009	0.005	0.0996	0.19	0.013	0.005	0.011
rs6968554	G	0.61	0.58	-0.005	0.004	0.248	0.66	0.004	0.004	0.236
rs6970595	C	0.58	0.8	0.002	0.005	0.638	0.77	0.005	0.004	0.279
rs698147	A	0.45	0.45	0.005	0.004	0.244	0.51	-0.002	0.004	0.551
rs7024334	T	0.23	0.57	0.009	0.004	0.0362	0.58	-0.014	0.004	<5E-08
rs7031064	A	0.53	0.59	0.004	0.004	0.3	0.42	0.0001	0.004	0.927
rs7037266	C	0.36	0.69	-0.003	0.005	0.483	0.63	0.008	0.004	0.035
rs704061	C	0.55	0.62	-0.006	0.004	0.159	0.95	0.005	0.009	0.059
rs7070670	C	0.32	0.18	-0.001	0.006	0.878	0.13	0.0001	0.006	0.97
rs7084454	A	0.31	0.01	0.018	0.018	0.322		Low MAF*		
rs709400	A	0.62	0.93	-0.006	0.008	0.486	0.87	-0.01	0.005	0.054
rs7123876	C	0.75	0.84	-0.007	0.006	0.24	0.76	0.015	0.004	<5E-08
rs7124681	A	0.42	0.31	0.021	0.005	4.48E-06	0.31	-0.022	0.004	<5E-08
rs7133378	A	0.33	0.13	0.011	0.006	0.0826	0.16	-0.008	0.005	0.117
rs7134628	A	0.1	0.01	-0.008	0.032	0.814		Low MAF*		
rs713763	A	0.42	0.19	-0.001	0.005	0.91	0.19	-0.004	0.005	0.459
rs7138803	A	0.39	0.28	0.023	0.005	1.63E-06	0.35	-0.01	0.004	0.007
rs7141307	C	0.74	0.86	-0.016	0.006	0.00996	0.79	0.008	0.005	0.083
rs7144011	T	0.23	0.002	-0.028	0.044	0.525		Low MAF*		
rs715	C	0.7	0.82	-0.011	0.005	0.0504	0.84	0.014	0.005	0.005
rs7181610	A	0.86	0.99	-0.028	0.062	0.656	0.84	-0.002	0.005	0.607
rs7186893	G	0.26	0.1	0	0.007	0.997	0.08	0.014	0.006	0.03
rs7189122	C	0.83	0.66	-0.005	0.004	0.258	0.66	0.009	0.004	0.012
rs719802	T	0.4	0.48	0.001	0.004	0.82	0.56	-0.007	0.004	0.052
rs7206608	G	0.68	0.94	-0.015	0.009	0.0776	0.94	0.001	0.008	0.88
rs7217226	G	0.66	0.74	-0.016	0.005	0.000727	0.77	0.02	0.004	<5E-08
rs7219230	T	0.34	0.74	0.015	0.005	0.00134	0.85	-0.005	0.005	0.56

rs7238896	G	0.86	0.99	-0.036	0.044	0.418		Low MAF*		
rs7239114	A	0.54	0.12	0.017	0.007	0.0104	0.11	-0.014	0.006	0.01
rs7243357	T	0.83	0.8	0.029	0.005	4.75E-08	0.77	-0.015	0.005	0.005
rs7259325	G	0.48	0.7	-0.004	0.005	0.402	0.7	-0.003	0.004	0.515
rs72673947	G	0.89	0.92	-0.006	0.008	0.437	0.9	0.004	0.006	0.486
rs730180	A	0.79	0.46	0.006	0.004	0.163	0.49	-0.005	0.004	0.145
rs7318817	C	0.63	0.83	-0.018	0.006	0.00129	0.86	0.012	0.005	0.014
rs73213501	A	0.83	0.83	-0.002	0.006	0.659	0.9	-0.004	0.006	0.541
rs73249175	G	0.88	0.99	0.006	0.066	0.93		Low MAF*		
rs7357754	G	0.5	0.72	0.001	0.005	0.864	0.65	0.005	0.004	0.175
rs740157	A	0.45	0.37	0.01	0.004	0.0185	0.37	-0.01	0.004	0.005
rs7498044	G	0.21	0.01	-0.016	0.025	0.513		Low MAF*		
rs7498665	G	0.62	0.88	-0.017	0.006	0.00793	0.86	0.03	0.005	<5E-08
rs7523668	G	0.44	0.72	0	0.005	0.981	0.65	0.005	0.004	0.17
rs7534091	G	0.72	0.66	-0.017	0.004	0.000104	0.51	0.015	0.004	<5E-08
rs7537581	A	0.53	0.53	0.013	0.004	0.00324	0.48	0.0001	0.00411	0.03179
rs7561278	T	0.77	0.67	0.016	0.005	0.000322	0.56	-0.016	0.004	<5E-08
rs7570446	A	0.54	0.59	0	0.004	0.962	0.43	0	0.004	0.988
rs7575118	T	0.16	0.02	0.002	0.021	0.916	0.02	-0.024	0.012	0.051
rs7588437	G	0.37	0.25	-0.016	0.005	0.000979	0.22	0.018	0.004	<5E-08
rs7589023	T	0.84	0.96	0.017	0.011	0.106	0.96	0.003	0.009	0.753
rs7599312	G	0.27	0.03	-0.018	0.012	0.134	0.04	0.015	0.01	0.111
rs7601895	C	0.69	0.94	0.014	0.009	0.11	0.91	0.003	0.006	0.675
rs761423	T	0.54	0.73	0.006	0.005	0.221	0.71	-0.011	0.004	0.005
rs7616009	G	0.16	0.42	0.004	0.004	0.336	0.5	0.012	0.004	0.001
rs7621025	C	0.26	0.13	-0.005	0.006	0.427	0.18	0.015	0.005	0.002
rs7631156	A	0.31	0.13	0.025	0.006	7.41E-05	0.08	-0.021	0.006	0.001
rs765875	C	0.47	0.55	-0.009	0.004	0.04	0.55	0.0001	0.004	0.918
rs7678054	G	0.48	0.7	-0.007	0.005	0.124	0.79	0.013	0.004	0.003
rs76942203	A	0.06	0.1	0.019	0.007	0.00628	0.1	-0.019	0.006	0.002
rs7713317	G	0.72	0.67	-0.019	0.004	3.21E-05	0.78	0.021	0.004	<5E-08
rs7714712	C	0.37	0.19	-0.021	0.005	0.000116	0.14	0.012	0.005	0.023

rs7734385	G	0.44	0.23	0.005	0.005	0.303	0.26	0.005	0.004	0.199
rs7740107	T	0.74	0.97	-0.035	0.011	0.00171	0.98	0.03	0.012	0.013
rs77432547	G	0.72	0.95	0.011	0.009	0.232	0.95	0.02	0.008	0.013
rs7769594	T	0.16	0.14	-0.005	0.006	0.41	0.07	-0.004	0.007	0.541
rs7777084	A	0.46	0.41	0.003	0.004	0.417	0.37	-0.012	0.004	0.002
rs7784465	C	0.86	0.99	-0.006	0.016	0.724	0.99	0.009	0.018	0.624
rs779206	G	0.72	0.67	0.006	0.004	0.16	0.64	0.004	0.004	0.249
rs7802342	G	0.7	0.78	-0.013	0.005	0.0106	0.74	0.01	0.004	0.016
rs7826312	C	0.43	0.83	0	0.006	0.969	0.84	-0.003	0.005	0.514
rs786420	T	0.77	0.36	0.006	0.004	0.179	0.42	0.001	0.004	0.842
rs7871866	C	0.16	0.19	0.014	0.006	0.0102	0.22	-0.016	0.005	<5E-08
rs7893571	T	0.67	0.89	-0.005	0.007	0.485	0.9	-0.017	0.006	0.003
rs7899106	G	0.95	0.99	-0.061	0.035	0.0791		Low MAF*		
rs7903146	C	0.28	0.04	-0.012	0.011	0.291	0.04	-0.057	0.009	<5E-08
rs79113395	G	0.27	0.15	-0.008	0.006	0.221		Low MAF*		
rs7944782	G	0.5	0.45	-0.013	0.004	0.00231	0.55	0.005	0.004	0.15
rs7947143	G	0.15	0.13	0.003	0.006	0.583	0.09	0.001	0.006	0.85
rs7961979	A	0.13	0.002	-0.042	0.051	0.411		Low MAF*		
rs7968390	A	0.68	0.57	0.008	0.004	0.076	0.41	-0.01	0.004	0.004
rs7975187	G	0.77	0.69	0.001	0.005	0.877	0.62	0.013	0.004	0.001
rs7976757	C	0.18	0.28	0.001	0.005	0.816	0.23	0.007	0.004	0.091
rs8016859	C	0.04	0.001	0.063	0.084	0.453		Low MAF*		
rs8024806	T	0.94	0.98	-0.006	0.015	0.667	0.99	0.007	0.017	0.696
rs8033510	T	0.36	0.39	0.008	0.004	0.0703	0.4	-0.009	0.004	0.017
rs8038464	T	0.4	0.5	-0.013	0.004	0.00318	0.47	0.008	0.004	0.033
rs8075273	C	0.29	0.07	-0.007	0.008	0.392	0.05	-0.004	0.008	0.633
rs8081039	T	0.06	0.005	0.034	0.033	0.298	0.03	0.016	0.011	0.149
rs8092503	G	0.77	0.57	-0.007	0.004	0.0858	0.7	0.018	0.004	<5E-08
rs8122855	A	0.34	0.62	0.01	0.004	0.0185	0.62	0.001	0.004	0.756
rs8134638	C	0.63	0.75	0.003	0.005	0.59	0.77	0.005	0.005	0.286
rs816367	G	0.65	0.67	-0.008	0.005	0.0766	0.64	0.008	0.004	0.023
rs8181823	C	0.23	0.08	-0.011	0.008	0.136	0.09	0.005	0.006	0.423

rs845084	A	0.27	0.43	0.022	0.004	2.20E-07	0.35	-0.013	0.004	0.001
rs857601	T	0.71	0.96	0.001	0.01	0.906	0.97	0.03	0.01	0.003
rs867560	G	0.57	0.15	-0.018	0.006	0.0027	0.1	0.014	0.006	0.019
rs872281	C	0.18	0.32	-0.004	0.005	0.324	0.25	0.006	0.004	0.143
rs889398	C	0.42	0.12	-0.004	0.007	0.5	0.14	0.006	0.005	0.258
rs891387	T	0.5	0.08	0.014	0.008	0.0662	0.06	-0.018	0.007	0.015
rs9077	G	0.27	0.06	-0.014	0.009	0.117	0.05	-0.002	0.008	0.771
rs930295	A	0.15	0.19	0.008	0.005	0.142	0.17	-0.011	0.005	0.029
rs9317002	A	0.51	0.32	0.008	0.005	0.0718	0.3	-0.01	0.004	0.014
rs9320823	C	0.41	0.42	0.005	0.004	0.216	0.37	0.007	0.004	0.048
rs934515	A	0.12	0.2	0.014	0.005	0.0109	0.18	0.005	0.005	0.265
rs9376609	A	0.38	0.28	0.006	0.005	0.224	0.21	0.007	0.004	0.091
rs942066	G	0.63	0.8	-0.007	0.005	0.202	0.82	0.018	0.005	<5E-08
rs944340	T	0.42	0.64	0.003	0.004	0.47	0.6	0.0001	0.004	0.992
rs945211	C	0.63	0.32	0.007	0.005	0.12	0.3	-0.011	0.004	0.004
rs946824	T	0.14	0.11	0.008	0.007	0.236	0.09	-0.007	0.006	0.257
rs947088	T	0.74	0.99	0.029	0.029	0.319		Low MAF*		
rs9479509	A	0.29	0.62	0.008	0.004	0.0583	0.5	-0.015	0.004	<5E-08
rs9514131	G	0.13	0.1	-0.009	0.007	0.219	0.1	0.006	0.006	0.31
rs9527455	C	0.23	0.08	-0.019	0.008	0.0156		Low MAF*		
rs9540493	A	0.46	0.75	0.01	0.005	0.0615	0.67	-0.016	0.005	0.001
rs9571687	C	0.34	0.38	-0.016	0.004	0.000326	0.37	0.003	0.004	0.424
rs9595908	T	0.64	0.83	0.009	0.006	0.0989	0.84	-0.014	0.005	0.006
rs9603697	T	0.32	0.48	0.01	0.004	0.0176	0.49	-0.007	0.004	0.061
rs972540	G	0.72	0.8	-0.005	0.005	0.327	0.83	0.013	0.005	0.008
rs9808302	G	0.53	0.52	0.004	0.004	0.315	0.47	-0.007	0.004	0.038
rs9816029	C	0.34	0.08	0.015	0.008	0.047	0.09	-0.005	0.006	0.403
rs9816226	T	0.18	0.05	-0.061	0.01	1.27E-09	0.02	0.04	0.015	0.008
rs9818122	C	0.79	0.99	-0.013	0.031	0.673		Low MAF*		
rs9826775	A	0.85	0.86	-0.007	0.006	0.24	0.87	-0.005	0.005	0.338
rs9839267	T	0.89	0.85	0.028	0.006	2.60E-06	0.83	-0.011	0.005	0.018
rs9839653	G	0.83	0.99	-0.071	0.046	0.122		Low MAF*		

rs985060	G	0.27	0.43	-0.002	0.004	0.601	0.39	0.001	0.004	0.846
rs9862795	T	0.52	0.85	0.005	0.006	0.388	0.84	0.015	0.005	0.002
rs987071	C	0.77	0.99	0.012	0.026	0.648		Low MAF*		
rs9929792	A	0.5	0.86	0.013	0.006	0.0269	0.91	0.013	0.006	0.042
rs9937053	A	0.44	0.17	0.072	0.006	2.09E-38	0.23	-0.069	0.004	<5E-08
rs9965170	G	0.43	0.14	-0.009	0.006	0.152	0.15	0.012	0.005	0.016

Abbreviation: MAF, minor allele frequency.

Supplementary Table 3. Associations of potential confounders with BMI GRS

Confounder	OR (95% CI)
**Current smoking**	1.03 (0.94, 1.13)
**Weekly drinking**	1.09 (0.98, 1.21)
**Physical activity**	0.93 (0.82, 1.04)
**Education**	0.99 (0.89, 1.10)
**Income**	1.02 (0.95, 1.09)

The model was adjusted for age at baseline, age squared, sex, 10 regions, and 10 PCs, education, smoking, and alcohol.
Potential confounders were dichotomised: current smoking (yes vs no), weekly drinking (yes vs no), total PA (≥30 vs <30 MET-h/day), education (≥9 vs <9 years), and household income (≥10,000 vs <10,000 RMB/year).
The analysis of smoking and alcohol was conducted in men due to the small number of women who smoked or drank.

Supplementary Table 4. Observational associations of adiposity with metabolic biomarkers

Biochemical	BMI				WC				WCadjBMI			
	β	SE	p-valule	p-BH	β	SE	p-valule	p-BH	β	SE	p-valule	p-BH
**FDR-adjusted p-value<0.05 for BMI**												
1-(1-enyl-palmitoyl)-2-linoleoyl-GPC (P-16:0/18:2)*	-0.216	0.074	0.004	0.029	-0.269	0.076	0.001	0.006	-0.323	0.164	0.050	0.819
1-(1-enyl-palmitoyl)-2-oleoyl-GPC (P-16:0/18:1)*	-0.350	0.070	0.000	0.000	-0.409	0.072	0.000	0.000	-0.393	0.153	0.011	0.738
1-(1-enyl-palmitoyl)-2-palmitoleoyl-GPC (P-16:0/16:1)*	-0.232	0.082	0.005	0.034	-0.287	0.084	0.001	0.008	-0.338	0.180	0.062	0.819
1,5-anhydroglucitol (1,5-AG)	-0.309	0.079	0.000	0.002	-0.357	0.081	0.000	0.000	-0.333	0.174	0.057	0.819
1-arachidonylglycerol (20:4)	0.263	0.079	0.001	0.011	0.257	0.084	0.003	0.018	0.055	0.181	0.760	0.982
1-carboxyethylisoleucine	0.386	0.090	0.000	0.001	0.366	0.094	0.000	0.002	0.068	0.192	0.724	0.980
1-carboxyethylleucine	0.413	0.083	0.000	0.000	0.369	0.088	0.000	0.001	-0.045	0.184	0.806	0.988
1-carboxyethylphenylalanine	0.306	0.078	0.000	0.002	0.299	0.082	0.000	0.004	0.077	0.174	0.657	0.980
1-carboxyethylvaline	0.343	0.076	0.000	0.000	0.333	0.080	0.000	0.001	0.083	0.168	0.621	0.972
1-linolenoylglycerol (18:3)	0.218	0.075	0.004	0.029	0.248	0.078	0.002	0.014	0.218	0.173	0.210	0.841
1-linoleoylglycerol (18:2)	0.315	0.075	0.000	0.001	0.336	0.077	0.000	0.001	0.212	0.166	0.202	0.841
1-linoleoyl-GPG (18:2)*	0.259	0.066	0.000	0.002	0.263	0.068	0.000	0.002	0.113	0.146	0.442	0.902
1-methylhistidine	0.215	0.080	0.008	0.047	0.203	0.084	0.017	0.082	0.021	0.179	0.909	0.992
1-methylurate	0.221	0.081	0.007	0.043	0.201	0.085	0.020	0.092	-0.022	0.185	0.904	0.992
1-myristoyl-2-arachidonoyl-GPC (14:0/20:4)*	0.378	0.078	0.000	0.000	0.355	0.083	0.000	0.001	0.036	0.175	0.837	0.988
1-myristoyl-2-palmitoyl-GPC (14:0/16:0)	0.282	0.077	0.000	0.004	0.304	0.080	0.000	0.003	0.205	0.170	0.232	0.844
1-oleoyl-2-docosahexaenoyl-GPC (18:1/22:6)*	-0.368	0.080	0.000	0.000	-0.411	0.083	0.000	0.000	-0.329	0.177	0.064	0.819
1-oleoylglycerol (18:1)	0.304	0.071	0.000	0.001	0.354	0.073	0.000	0.000	0.338	0.156	0.032	0.738
1-palmitoleoylglycerol (16:1)*	0.329	0.077	0.000	0.001	0.392	0.079	0.000	0.000	0.398	0.169	0.019	0.738
1-palmitoyl-2-arachidonoyl-GPC (16:0/20:4n6)	0.348	0.078	0.000	0.000	0.317	0.083	0.000	0.003	-0.013	0.175	0.940	0.993
1-palmitoyl-2-dihomo-linolenoyl-GPC (16:0/20:3n3 or 6)*	0.273	0.074	0.000	0.004	0.335	0.076	0.000	0.000	0.381	0.162	0.020	0.738
1-palmitoyl-2-linoleoyl-GPC (16:0/18:2)	0.281	0.076	0.000	0.004	0.291	0.079	0.000	0.004	0.149	0.169	0.381	0.888
1-palmitoyl-2-oleoyl-GPE (16:0/18:1)	0.231	0.076	0.003	0.021	0.268	0.079	0.001	0.008	0.252	0.168	0.136	0.819
1-palmitoyl-2-palmitoleoyl-GPC (16:0/16:1)*	0.220	0.072	0.003	0.020	0.253	0.074	0.001	0.008	0.229	0.159	0.152	0.819
1-palmitoyl-2-stearoyl-GPE (16:0/18:0)*	0.320	0.083	0.000	0.002	0.326	0.087	0.000	0.003	0.133	0.183	0.468	0.902
1-stearoyl-2-arachidonoyl-GPC (18:0/20:4)	0.237	0.082	0.004	0.029	0.212	0.086	0.015	0.075	-0.031	0.183	0.865	0.992
1-stearoyl-2-arachidonoyl-GPE (18:0/20:4)	0.326	0.079	0.000	0.001	0.342	0.082	0.000	0.001	0.191	0.175	0.277	0.869
1-stearoyl-2-arachidonoyl-GPI (18:0/20:4)	0.263	0.078	0.001	0.009	0.330	0.080	0.000	0.001	0.404	0.171	0.019	0.738

1-stearoyl-2-docosahexaenoyl-GPE (18:0/22:6)*	0.309	0.073	0.000	0.001	0.321	0.076	0.000	0.001	0.167	0.162	0.306	0.873
1-stearoyl-2-linoleoyl-GPE (18:0/18:2)*	0.295	0.078	0.000	0.003	0.305	0.082	0.000	0.003	0.155	0.175	0.376	0.888
1-stearoyl-2-oleoyl-GPE (18:0/18:1)	0.332	0.075	0.000	0.000	0.326	0.078	0.000	0.001	0.095	0.167	0.571	0.947
1-stearoyl-2-oleoyl-GPS (18:0/18:1)	0.330	0.077	0.000	0.001	0.302	0.081	0.000	0.003	-0.009	0.172	0.959	0.994
2,3-dihydroxy-5-methylthio-4-pentenoate (DMTPA)*	0.308	0.082	0.000	0.003	0.340	0.085	0.000	0.001	0.257	0.181	0.157	0.819
2-aminoadipate	0.414	0.104	0.000	0.002	0.458	0.111	0.000	0.002	0.296	0.236	0.214	0.841
2-aminoheptanoate	0.228	0.078	0.004	0.027	0.248	0.081	0.002	0.018	0.174	0.173	0.314	0.875
2-hydroxyarachidate*	0.222	0.080	0.006	0.037	0.212	0.083	0.012	0.063	0.033	0.178	0.854	0.988
2-hydroxybehenate	0.216	0.069	0.002	0.018	0.226	0.072	0.002	0.016	0.125	0.154	0.420	0.893
2-hydroxyphenylacetate	0.244	0.087	0.006	0.037	0.207	0.091	0.024	0.105	-0.079	0.197	0.690	0.980
2-linoleoylglycerol (18:2)	0.213	0.079	0.008	0.047	0.201	0.083	0.017	0.081	0.022	0.176	0.903	0.992
2-methylbutyrylcarnitine (C5)	0.320	0.076	0.000	0.001	0.355	0.080	0.000	0.000	0.251	0.168	0.138	0.819
2-oxoarginine*	0.368	0.071	0.000	0.000	0.346	0.075	0.000	0.000	0.031	0.158	0.845	0.988
3beta-hydroxy-5-cholestenoate	-0.227	0.074	0.003	0.020	-0.227	0.077	0.004	0.026	-0.080	0.165	0.629	0.974
3b-hydroxy-5-cholenoic acid	-0.287	0.106	0.008	0.047	-0.153	0.115	0.184	0.404	0.460	0.226	0.044	0.797
3-hydroxy-2-ethylpropionate	0.396	0.082	0.000	0.000	0.411	0.085	0.000	0.000	0.212	0.182	0.244	0.847
3-hydroxyisobutyrate	0.371	0.081	0.000	0.000	0.364	0.085	0.000	0.001	0.107	0.180	0.554	0.947
3-methyl-2-oxovalerate	0.251	0.082	0.003	0.020	0.251	0.086	0.004	0.027	0.089	0.183	0.629	0.974
4-cholesten-3-one	0.329	0.074	0.000	0.000	0.362	0.077	0.000	0.000	0.254	0.164	0.124	0.819
4-guanidinobutanoate	0.279	0.074	0.000	0.003	0.259	0.078	0.001	0.009	0.014	0.165	0.931	0.993
4-hydroxyglutamate	0.394	0.074	0.000	0.000	0.326	0.082	0.000	0.002	-0.197	0.167	0.240	0.847
5-hydroxylysine	0.267	0.080	0.001	0.010	0.199	0.084	0.020	0.091	-0.213	0.177	0.232	0.844
5-methylthioadenosine (MTA)	0.222	0.070	0.002	0.016	0.274	0.073	0.000	0.003	0.313	0.155	0.045	0.811
6-oxopiperidine-2-carboxylate	0.294	0.085	0.001	0.007	0.361	0.088	0.000	0.001	0.405	0.185	0.030	0.738
alanine	0.230	0.084	0.007	0.042	0.237	0.087	0.007	0.044	0.116	0.187	0.535	0.943
arabinose	0.284	0.090	0.002	0.017	0.248	0.090	0.007	0.042	-0.001	0.190	0.998	0.999
argininate*	0.262	0.079	0.001	0.011	0.291	0.082	0.000	0.005	0.229	0.176	0.194	0.839
aspartate	0.252	0.071	0.001	0.006	0.277	0.074	0.000	0.003	0.206	0.158	0.195	0.840
behenoyl dihydrosphingomyelin (d18:0/22:0)*	0.416	0.069	0.000	0.000	0.457	0.071	0.000	0.000	0.342	0.152	0.026	0.738
beta-alanine	0.293	0.076	0.000	0.002	0.300	0.080	0.000	0.003	0.138	0.170	0.419	0.893
beta-citrylglutamate	0.250	0.073	0.001	0.008	0.240	0.076	0.002	0.014	0.047	0.162	0.771	0.983
beta-hydroxyisovalerate	0.357	0.075	0.000	0.000	0.341	0.079	0.000	0.001	0.057	0.168	0.735	0.981
betaine	-0.232	0.074	0.002	0.017	-0.199	0.078	0.011	0.061	0.065	0.165	0.693	0.980

bilirubin	-0.246	0.080	0.002	0.019	-0.282	0.083	0.001	0.008	-0.256	0.177	0.149	0.819
bilirubin (E,E)*	-0.224	0.077	0.004	0.029	-0.268	0.080	0.001	0.009	-0.286	0.170	0.095	0.819
bilirubin (E,Z or Z,E)*	-0.252	0.080	0.002	0.016	-0.273	0.083	0.001	0.010	-0.190	0.177	0.285	0.870
biliverdin	-0.231	0.073	0.002	0.016	-0.244	0.076	0.002	0.013	-0.143	0.163	0.380	0.888
butyrylcarnitine (C4)	0.302	0.077	0.000	0.002	0.336	0.079	0.000	0.001	0.275	0.168	0.104	0.819
carotene diol (2)	-0.238	0.078	0.002	0.019	-0.211	0.081	0.010	0.056	0.036	0.173	0.835	0.988
ceramide (d16:1/24:1, d18:1/22:1)*	0.159	0.057	0.006	0.037	0.172	0.058	0.004	0.025	0.127	0.127	0.319	0.875
ceramide (d18:1/17:0, d17:1/18:0)*	0.242	0.086	0.005	0.035	0.276	0.088	0.002	0.016	0.248	0.188	0.190	0.839
ceramide (d18:1/20:0, d16:1/22:0, d20:1/18:0)*	0.271	0.075	0.000	0.005	0.302	0.078	0.000	0.002	0.242	0.167	0.149	0.819
cortisol	-0.243	0.081	0.003	0.022	-0.270	0.084	0.001	0.012	-0.216	0.179	0.230	0.844
cortisone	-0.314	0.078	0.000	0.002	-0.352	0.081	0.000	0.001	-0.284	0.175	0.106	0.819
cystathionine	0.231	0.071	0.001	0.013	0.201	0.075	0.008	0.048	-0.054	0.159	0.737	0.981
cysteine-glutathione disulfide	-0.234	0.078	0.003	0.023	-0.199	0.082	0.016	0.079	0.076	0.176	0.665	0.980
diacylglycerol (14:0/18:1, 16:0/16:1) [2]*	0.267	0.094	0.005	0.034	0.323	0.099	0.001	0.013	0.334	0.222	0.135	0.819
diacylglycerol (16:1/18:2 [2], 16:0/18:3 [1])*	0.388	0.082	0.000	0.000	0.401	0.083	0.000	0.000	0.239	0.169	0.161	0.819
dimethylglycine	0.258	0.081	0.002	0.015	0.277	0.085	0.001	0.011	0.179	0.181	0.323	0.877
ergothioneine	0.206	0.076	0.007	0.043	0.150	0.080	0.062	0.203	-0.185	0.168	0.272	0.857
erythritol	0.258	0.087	0.004	0.026	0.268	0.091	0.004	0.025	0.142	0.195	0.468	0.902
formiminoglutamate	0.441	0.073	0.000	0.000	0.414	0.077	0.000	0.000	0.036	0.162	0.827	0.988
gamma-glutamyl-alpha-lysine	0.239	0.080	0.003	0.024	0.268	0.083	0.002	0.013	0.216	0.179	0.229	0.844
gamma-glutamylglutamate	0.322	0.076	0.000	0.001	0.380	0.078	0.000	0.000	0.378	0.168	0.025	0.738
gamma-glutamylglycine	-0.260	0.080	0.001	0.012	-0.295	0.083	0.000	0.005	-0.251	0.177	0.158	0.819
gamma-glutamylisoleucine*	0.419	0.075	0.000	0.000	0.458	0.078	0.000	0.000	0.330	0.166	0.049	0.819
gamma-glutamylleucine	0.423	0.076	0.000	0.000	0.422	0.080	0.000	0.000	0.150	0.169	0.377	0.888
gamma-glutamylphenylalanine	0.339	0.083	0.000	0.001	0.366	0.086	0.000	0.001	0.248	0.183	0.177	0.839
gamma-glutamyltyrosine	0.268	0.076	0.001	0.006	0.306	0.079	0.000	0.002	0.270	0.168	0.111	0.819
gamma-glutamylvaline	0.387	0.072	0.000	0.000	0.408	0.075	0.000	0.000	0.239	0.160	0.137	0.819
gamma-tocopherol/beta-tocopherol	0.205	0.067	0.003	0.020	0.261	0.069	0.000	0.003	0.331	0.148	0.026	0.738
glucose	0.284	0.082	0.001	0.007	0.282	0.086	0.001	0.011	0.090	0.183	0.623	0.972
glucuronide of C10H18O2 (7)*	-0.820	0.224	0.003	0.020	-0.766	0.313	0.028	0.118	0.528	0.597	0.393	0.891
glutamate	0.428	0.072	0.000	0.000	0.456	0.074	0.000	0.000	0.283	0.159	0.076	0.819
glycine	-0.325	0.077	0.000	0.001	-0.358	0.079	0.000	0.000	-0.269	0.170	0.115	0.819
glycosyl-N-(2-hydroxynervonoyl)-sphingosine (d18:1/24:1(2OH))*	0.211	0.055	0.000	0.002	0.207	0.057	0.000	0.004	0.060	0.122	0.623	0.972

glycosyl-N-nervonoyl-sphingosine (d18:1/24:1)*	-0.180	0.049	0.000	0.004	-0.202	0.050	0.000	0.001	-0.170	0.108	0.118	0.819
gulonate*	0.251	0.086	0.004	0.029	0.228	0.090	0.012	0.065	-0.010	0.193	0.958	0.994
hexadecasphingosine (d16:1)*	0.323	0.078	0.000	0.001	0.309	0.084	0.000	0.004	0.029	0.178	0.873	0.992
homoarginine	0.299	0.081	0.000	0.004	0.315	0.084	0.000	0.003	0.186	0.180	0.305	0.873
homocitrulline	0.298	0.089	0.001	0.010	0.290	0.093	0.002	0.017	0.071	0.199	0.722	0.980
hydantoin-5-propionate	0.338	0.087	0.000	0.002	0.290	0.090	0.002	0.013	-0.073	0.189	0.701	0.980
hydroxyasparagine	0.230	0.083	0.006	0.037	0.269	0.086	0.002	0.015	0.261	0.183	0.156	0.819
isoleucine	0.416	0.082	0.000	0.000	0.411	0.086	0.000	0.000	0.130	0.183	0.480	0.902
isovalerate (C5)	0.358	0.076	0.000	0.000	0.381	0.079	0.000	0.000	0.237	0.168	0.159	0.819
isovalerylcarnitine (C5)	0.406	0.078	0.000	0.000	0.408	0.082	0.000	0.000	0.157	0.173	0.366	0.888
lactosyl-N-nervonoyl-sphingosine (d18:1/24:1)*	-0.213	0.062	0.001	0.007	-0.204	0.065	0.002	0.014	-0.035	0.137	0.800	0.988
lactosyl-N-palmitoyl-sphingosine (d18:1/16:0)	-0.218	0.073	0.003	0.024	-0.173	0.077	0.027	0.114	0.127	0.163	0.436	0.900
leucine	0.430	0.081	0.000	0.000	0.421	0.085	0.000	0.000	0.115	0.180	0.525	0.942
linoleoyl-arachidonoyl-glycerol (18:2/20:4) [1]*	0.374	0.073	0.000	0.000	0.412	0.076	0.000	0.000	0.310	0.162	0.057	0.819
linoleoyl-arachidonoyl-glycerol (18:2/20:4) [2]*	0.404	0.073	0.000	0.000	0.391	0.077	0.000	0.000	0.088	0.163	0.592	0.961
linoleoyl-docosahexaenoyl-glycerol (18:2/22:6) [2]*	0.249	0.065	0.000	0.003	0.254	0.067	0.000	0.003	0.124	0.144	0.392	0.891
linoleoyl-linolenoyl-glycerol (18:2/18:3) [2]*	0.272	0.073	0.000	0.004	0.274	0.076	0.000	0.004	0.115	0.163	0.480	0.902
linoleoyl-linoleoyl-glycerol (18:2/18:2) [1]*	0.202	0.063	0.002	0.015	0.219	0.066	0.001	0.010	0.153	0.141	0.280	0.869
lysine	0.250	0.085	0.004	0.026	0.281	0.088	0.002	0.013	0.235	0.188	0.213	0.841
mannitol/sorbitol	0.223	0.084	0.008	0.049	0.194	0.088	0.028	0.118	-0.052	0.187	0.780	0.986
mannonate*	0.355	0.083	0.000	0.001	0.374	0.086	0.000	0.001	0.215	0.185	0.246	0.847
mannose	0.279	0.083	0.001	0.009	0.314	0.086	0.000	0.004	0.262	0.183	0.156	0.819
myristoyl dihydrosphingomyelin (d18:0/14:0)*	0.263	0.080	0.001	0.011	0.294	0.083	0.001	0.005	0.236	0.177	0.185	0.839
myristoyl-linoleoyl-glycerol (14:0/18:2) [1]*	0.292	0.086	0.001	0.009	0.371	0.086	0.000	0.001	0.488	0.186	0.009	0.738
N6,N6,N6-trimethyllysine	0.275	0.077	0.000	0.005	0.319	0.080	0.000	0.002	0.298	0.171	0.082	0.819
N-acetylalanine	0.233	0.085	0.007	0.043	0.220	0.089	0.015	0.074	0.027	0.190	0.889	0.992
N-acetylcarnosine	0.346	0.061	0.000	0.000	0.345	0.064	0.000	0.000	0.124	0.136	0.363	0.888
N-acetylglutamate	0.239	0.076	0.002	0.017	0.192	0.080	0.018	0.085	-0.125	0.169	0.461	0.902
N-acetylglycine	-0.243	0.071	0.001	0.008	-0.265	0.073	0.000	0.004	-0.188	0.157	0.232	0.844
N-acetylisoleucine	0.341	0.079	0.000	0.001	0.341	0.083	0.000	0.001	0.126	0.177	0.477	0.902
N-acetylleucine	0.415	0.086	0.000	0.000	0.411	0.090	0.000	0.000	0.127	0.193	0.512	0.934
N-acetylphenylalanine	0.324	0.084	0.000	0.002	0.367	0.087	0.000	0.001	0.314	0.185	0.092	0.819
N-acetylputrescine	0.274	0.087	0.002	0.016	0.293	0.091	0.001	0.012	0.188	0.194	0.332	0.880

N-acetyltryptophan	0.232	0.082	0.005	0.036	0.298	0.085	0.001	0.006	0.384	0.181	0.036	0.761
N-acetyltyrosine	0.306	0.091	0.001	0.010	0.299	0.092	0.001	0.012	0.119	0.197	0.548	0.947
N-acetylvaline	0.266	0.079	0.001	0.009	0.290	0.082	0.001	0.005	0.206	0.175	0.241	0.847
N-palmitoyl-sphinganine (d18:0/16:0)	0.378	0.078	0.000	0.000	0.381	0.081	0.000	0.000	0.149	0.173	0.388	0.889
N-palmitoyl-sphingosine (d18:1/16:0)	0.239	0.081	0.004	0.026	0.275	0.084	0.001	0.011	0.252	0.179	0.161	0.819
N-stearoyl-sphinganine (d18:0/18:0)*	0.502	0.076	0.000	0.000	0.562	0.077	0.000	0.000	0.471	0.165	0.005	0.738
N-stearoyl-sphingosine (d18:1/18:0)*	0.367	0.075	0.000	0.000	0.404	0.078	0.000	0.000	0.304	0.167	0.069	0.819
oleoyl-arachidonoyl-glycerol (18:1/20:4) [1]*	0.376	0.077	0.000	0.000	0.401	0.080	0.000	0.000	0.250	0.170	0.143	0.819
oleoyl-arachidonoyl-glycerol (18:1/20:4) [2]*	0.421	0.077	0.000	0.000	0.436	0.080	0.000	0.000	0.222	0.170	0.194	0.839
oleoyl-linolenoyl-glycerol (18:1/18:3) [2]*	0.293	0.093	0.002	0.017	0.297	0.096	0.002	0.018	0.134	0.216	0.536	0.943
oleoyl-linoleoyl-glycerol (18:1/18:2) [1]	0.339	0.081	0.000	0.001	0.395	0.083	0.000	0.000	0.375	0.179	0.037	0.761
oleoyl-linoleoyl-glycerol (18:1/18:2) [2]	0.359	0.081	0.000	0.000	0.406	0.084	0.000	0.000	0.345	0.179	0.055	0.819
oleoyl-oleoyl-glycerol (18:1/18:1) [1]*	0.300	0.069	0.000	0.001	0.349	0.071	0.000	0.000	0.330	0.153	0.032	0.738
oleoyl-oleoyl-glycerol (18:1/18:1) [2]*	0.237	0.064	0.000	0.004	0.295	0.066	0.000	0.000	0.349	0.140	0.014	0.738
palmitoleoyl-arachidonoyl-glycerol (16:1/20:4) [2]*	0.294	0.078	0.000	0.003	0.297	0.083	0.000	0.005	0.098	0.180	0.585	0.957
palmitoleoyl-linoleoyl-glycerol (16:1/18:2) [1]*	0.306	0.079	0.000	0.002	0.375	0.080	0.000	0.000	0.427	0.172	0.014	0.738
palmitoyl-arachidonoyl-glycerol (16:0/20:4) [1]*	0.345	0.096	0.000	0.005	0.333	0.098	0.001	0.009	0.102	0.209	0.626	0.973
palmitoyl-arachidonoyl-glycerol (16:0/20:4) [2]*	0.412	0.078	0.000	0.000	0.417	0.081	0.000	0.000	0.170	0.173	0.325	0.877
palmitoyl-linoleoyl-glycerol (16:0/18:2) [1]*	0.378	0.078	0.000	0.000	0.443	0.079	0.000	0.000	0.434	0.170	0.012	0.738
palmitoyl-linoleoyl-glycerol (16:0/18:2) [2]*	0.385	0.080	0.000	0.000	0.444	0.081	0.000	0.000	0.412	0.174	0.019	0.738
palmitoyl-oleoyl-glycerol (16:0/18:1) [1]*	0.379	0.074	0.000	0.000	0.437	0.075	0.000	0.000	0.400	0.162	0.014	0.738
palmitoyl-oleoyl-glycerol (16:0/18:1) [2]*	0.359	0.076	0.000	0.000	0.420	0.077	0.000	0.000	0.411	0.166	0.014	0.738
pantothenate (Vitamin B5)	0.206	0.077	0.008	0.048	0.215	0.080	0.008	0.047	0.116	0.172	0.498	0.917
phenylalanine	0.316	0.080	0.000	0.002	0.317	0.084	0.000	0.003	0.122	0.178	0.493	0.912
proline	0.309	0.074	0.000	0.001	0.296	0.077	0.000	0.003	0.052	0.164	0.752	0.982
prolylglycine	0.264	0.083	0.002	0.015	0.243	0.087	0.006	0.038	-0.010	0.186	0.957	0.994
prolylhydroxyproline	0.212	0.076	0.006	0.037	0.167	0.080	0.038	0.144	-0.129	0.169	0.447	0.902
propionylcarnitine (C3)	0.251	0.077	0.001	0.012	0.304	0.079	0.000	0.002	0.336	0.169	0.049	0.819
pyridoxate	0.251	0.090	0.006	0.039	0.282	0.094	0.003	0.022	0.234	0.201	0.245	0.847
retinol (Vitamin A)	0.220	0.071	0.002	0.019	0.188	0.075	0.013	0.067	-0.066	0.158	0.677	0.980
S-adenosylhomocysteine (SAH)	0.295	0.103	0.005	0.033	0.253	0.107	0.020	0.092	-0.089	0.236	0.707	0.980
serine	-0.218	0.082	0.008	0.049	-0.263	0.085	0.002	0.017	-0.282	0.181	0.122	0.819
sphingadienine	0.247	0.073	0.001	0.009	0.252	0.076	0.001	0.010	0.111	0.162	0.496	0.916

sphingomyelin (d17:2/16:0, d18:2/15:0)*	0.237	0.065	0.000	0.004	0.220	0.068	0.001	0.012	0.009	0.145	0.952	0.993
sphingomyelin (d18:0/18:0, d19:0/17:0)*	0.447	0.072	0.000	0.000	0.488	0.075	0.000	0.000	0.351	0.159	0.029	0.738
sphingomyelin (d18:0/20:0, d16:0/22:0)*	0.491	0.074	0.000	0.000	0.518	0.077	0.000	0.000	0.301	0.163	0.066	0.819
sphingomyelin (d18:1/14:0, d16:1/16:0)*	0.253	0.075	0.001	0.009	0.283	0.078	0.000	0.004	0.230	0.166	0.168	0.826
sphingomyelin (d18:1/18:1, d18:2/18:0)	0.293	0.072	0.000	0.001	0.282	0.075	0.000	0.003	0.057	0.160	0.724	0.980
sphingomyelin (d18:1/21:0, d17:1/22:0, d16:1/23:0)*	0.173	0.057	0.003	0.022	0.162	0.060	0.008	0.046	0.012	0.128	0.928	0.993
sphingomyelin (d18:2/14:0, d18:1/14:1)*	0.291	0.069	0.000	0.001	0.284	0.072	0.000	0.002	0.074	0.154	0.632	0.974
sphingomyelin (d18:2/21:0, d16:2/23:0)*	0.176	0.064	0.007	0.043	0.111	0.068	0.105	0.285	-0.235	0.143	0.101	0.819
stearoyl-arachidonoyl-glycerol (18:0/20:4) [1]*	0.229	0.075	0.002	0.019	0.197	0.078	0.013	0.066	-0.062	0.166	0.709	0.980
stearoyl-arachidonoyl-glycerol (18:0/20:4) [2]*	0.221	0.083	0.008	0.049	0.201	0.087	0.021	0.096	-0.007	0.184	0.972	0.995
tyrosine	0.304	0.087	0.001	0.007	0.304	0.091	0.001	0.009	0.113	0.194	0.562	0.947
urate	0.244	0.067	0.000	0.004	0.249	0.070	0.000	0.005	0.115	0.149	0.440	0.900
valine	0.438	0.082	0.000	0.000	0.425	0.087	0.000	0.000	0.101	0.183	0.584	0.957
X - 11442	-0.229	0.085	0.008	0.046	-0.236	0.088	0.008	0.048	-0.117	0.189	0.536	0.943
X - 12063	0.291	0.076	0.000	0.002	0.342	0.078	0.000	0.000	0.337	0.166	0.044	0.797
X - 12170	0.261	0.087	0.003	0.022	0.249	0.091	0.007	0.042	0.041	0.193	0.834	0.988
X - 12411	0.306	0.080	0.000	0.002	0.292	0.083	0.001	0.006	0.047	0.178	0.791	0.988
X - 12456	0.343	0.100	0.001	0.009	0.413	0.108	0.000	0.003	0.344	0.211	0.106	0.819
X - 12524	0.282	0.076	0.000	0.003	0.252	0.080	0.002	0.014	-0.033	0.169	0.845	0.988
X - 12680	0.216	0.080	0.008	0.046	0.183	0.084	0.031	0.127	-0.069	0.176	0.697	0.980
X - 12689	0.306	0.073	0.000	0.001	0.290	0.076	0.000	0.003	0.037	0.162	0.819	0.988
X - 13737	0.238	0.082	0.004	0.029	0.285	0.085	0.001	0.009	0.301	0.183	0.102	0.819
X - 13844	0.386	0.127	0.004	0.027	0.488	0.127	0.000	0.004	0.792	0.338	0.023	0.738
X - 16946	-0.254	0.079	0.002	0.015	-0.267	0.083	0.002	0.013	-0.149	0.177	0.400	0.893
X - 17340	0.265	0.094	0.006	0.037	0.291	0.099	0.004	0.026	0.206	0.209	0.325	0.877
X - 18345	0.411	0.112	0.000	0.004	0.376	0.118	0.002	0.014	-0.109	0.298	0.716	0.980
X - 18886	0.358	0.079	0.000	0.000	0.358	0.082	0.000	0.001	0.130	0.175	0.460	0.902
X - 18922	0.228	0.068	0.001	0.009	0.269	0.070	0.000	0.002	0.272	0.149	0.069	0.819
X - 21829	0.330	0.079	0.000	0.001	0.356	0.082	0.000	0.001	0.247	0.173	0.157	0.819
X - 22775	0.264	0.091	0.004	0.029	0.255	0.095	0.008	0.049	0.050	0.203	0.806	0.988
X - 23294	0.326	0.116	0.006	0.039	0.365	0.124	0.004	0.029	0.252	0.273	0.358	0.887
X - 23590	0.409	0.084	0.000	0.000	0.436	0.088	0.000	0.000	0.270	0.188	0.153	0.819
X - 23593	0.348	0.081	0.000	0.001	0.306	0.086	0.000	0.005	-0.064	0.181	0.725	0.980

X - 23680	0.188	0.070	0.008	0.049	0.135	0.074	0.071	0.217	-0.176	0.156	0.262	0.848
X - 24106	-0.235	0.075	0.002	0.017	-0.207	0.079	0.010	0.054	0.045	0.168	0.790	0.988
X - 24328	0.221	0.070	0.002	0.015	0.232	0.072	0.002	0.013	0.130	0.154	0.399	0.893
X - 24588	0.286	0.080	0.000	0.006	0.293	0.084	0.001	0.006	0.137	0.179	0.444	0.902
X - 24728	0.283	0.078	0.000	0.004	0.287	0.081	0.001	0.005	0.122	0.174	0.485	0.905
X - 25371	0.307	0.087	0.001	0.006	0.355	0.090	0.000	0.002	0.331	0.192	0.086	0.819
**FDR-adjusted p-value>=0.05 for BMI**												
(14 or 15)-methylpalmitate (a17:0 or i17:0)	-0.045	0.082	0.582	0.754	-0.033	0.086	0.696	0.838	0.037	0.183	0.838	0.988
(16 or 17)-methylstearate (a19:0 or i19:0)	-0.023	0.077	0.768	0.877	-0.027	0.080	0.741	0.870	-0.026	0.172	0.881	0.992
(2,4 or 2,5)-dimethylphenol sulfate	0.076	0.075	0.309	0.530	0.062	0.077	0.422	0.644	-0.045	0.176	0.799	0.988
(N(1) + N(8))-acetylspermidine	0.098	0.086	0.254	0.472	0.147	0.089	0.099	0.278	0.260	0.190	0.173	0.831
1-(1-enyl-oleoyl)-GPE (P-18:1)*	-0.033	0.079	0.676	0.810	-0.025	0.082	0.760	0.881	0.024	0.176	0.892	0.992
1-(1-enyl-palmitoyl)-2-arachidonoyl-GPC (P-16:0/20:4)*	0.026	0.077	0.739	0.857	-0.031	0.080	0.699	0.838	-0.248	0.170	0.145	0.819
1-(1-enyl-palmitoyl)-2-arachidonoyl-GPE (P-16:0/20:4)*	0.143	0.075	0.058	0.192	0.137	0.078	0.081	0.240	0.025	0.167	0.882	0.992
1-(1-enyl-palmitoyl)-2-linoleoyl-GPE (P-16:0/18:2)*	0.067	0.079	0.399	0.608	0.042	0.082	0.606	0.771	-0.086	0.175	0.624	0.972
1-(1-enyl-palmitoyl)-2-oleoyl-GPE (P-16:0/18:1)*	0.135	0.072	0.063	0.200	0.096	0.076	0.204	0.428	-0.128	0.161	0.427	0.895
1-(1-enyl-palmitoyl)-2-palmitoyl-GPC (P-16:0/16:0)*	-0.176	0.079	0.027	0.116	-0.213	0.082	0.010	0.055	-0.235	0.175	0.180	0.839
1-(1-enyl-palmitoyl)-GPC (P-16:0)*	-0.161	0.084	0.058	0.192	-0.145	0.088	0.102	0.281	0.017	0.188	0.928	0.993
1-(1-enyl-palmitoyl)-GPE (P-16:0)*	0.016	0.082	0.841	0.920	0.039	0.086	0.652	0.804	0.107	0.183	0.560	0.947
1-(1-enyl-stearoyl)-2-arachidonoyl-GPE (P-18:0/20:4)*	0.041	0.075	0.583	0.754	0.045	0.079	0.565	0.755	0.033	0.168	0.847	0.988
1-(1-enyl-stearoyl)-2-linoleoyl-GPE (P-18:0/18:2)*	0.037	0.072	0.613	0.775	0.049	0.075	0.518	0.730	0.068	0.161	0.672	0.980
1-(1-enyl-stearoyl)-2-oleoyl-GPE (P-18:0/18:1)	0.037	0.062	0.549	0.726	0.052	0.064	0.421	0.644	0.081	0.137	0.557	0.947
1-(1-enyl-stearoyl)-GPE (P-18:0)*	-0.124	0.077	0.110	0.289	-0.092	0.081	0.254	0.483	0.099	0.172	0.564	0.947
1,2,3-benzenetriol sulfate (2)	0.003	0.089	0.972	0.988	-0.050	0.092	0.586	0.765	-0.243	0.196	0.218	0.844
1,2-dilinoleoyl-GPC (18:2/18:2)	-0.050	0.079	0.529	0.713	-0.049	0.082	0.553	0.749	-0.014	0.176	0.936	0.993
1,2-dilinoleoyl-GPE (18:2/18:2)*	0.173	0.083	0.038	0.142	0.173	0.086	0.046	0.168	0.066	0.184	0.721	0.980
1,2-dipalmitoyl-GPC (16:0/16:0)	0.049	0.078	0.531	0.713	0.053	0.081	0.518	0.730	0.035	0.174	0.842	0.988
1,2-dipalmitoyl-GPE (16:0/16:0)*	0.228	0.090	0.012	0.064	0.261	0.094	0.006	0.038	0.222	0.197	0.262	0.848
1,3-dimethylurate	0.285	0.164	0.090	0.254	0.335	0.171	0.057	0.193	0.408	0.459	0.379	0.888
1,7-dimethylurate	0.214	0.128	0.100	0.276	0.194	0.136	0.158	0.371	-0.074	0.337	0.826	0.988
10-heptadecenoate (17:1n7)	-0.021	0.079	0.789	0.886	0.002	0.082	0.983	0.989	0.097	0.176	0.583	0.957
10-nonadecenoate (19:1n9)	-0.079	0.079	0.317	0.541	-0.067	0.082	0.417	0.643	0.027	0.176	0.878	0.992

10-undecenoate (11:1n1)	-0.012	0.070	0.866	0.930	0.004	0.073	0.954	0.972	0.069	0.155	0.660	0.980
12,13-DiHOME	-0.086	0.077	0.268	0.486	-0.080	0.081	0.325	0.566	-0.001	0.177	0.998	0.999
13-HODE + 9-HODE	0.106	0.088	0.229	0.446	0.117	0.091	0.202	0.427	0.088	0.195	0.653	0.980
16a-hydroxy DHEA 3-sulfate	0.138	0.072	0.057	0.191	0.074	0.075	0.324	0.566	-0.239	0.159	0.135	0.819
16-hydroxypalmitate	-0.002	0.081	0.981	0.990	0.010	0.084	0.904	0.955	0.055	0.180	0.763	0.982
17alpha-hydroxypregnenolone 3-sulfate	0.062	0.108	0.568	0.742	0.103	0.113	0.363	0.601	0.187	0.226	0.411	0.893
1-arachidonoyl-GPA (20:4)	0.032	0.099	0.742	0.860	0.082	0.102	0.423	0.644	0.252	0.225	0.266	0.848
1-arachidonoyl-GPC* (20:4)*	0.003	0.083	0.971	0.988	-0.013	0.087	0.885	0.946	-0.070	0.186	0.706	0.980
1-arachidonoyl-GPE (20:4n6)*	0.066	0.077	0.388	0.598	0.082	0.080	0.307	0.547	0.094	0.171	0.583	0.957
1-arachidonoyl-GPI* (20:4)*	0.187	0.080	0.021	0.094	0.232	0.083	0.006	0.037	0.271	0.177	0.128	0.819
1-carboxyethyltyrosine	0.253	0.100	0.012	0.065	0.234	0.106	0.029	0.121	0.002	0.216	0.991	0.998
1-dihomo-linolenylglycerol (20:3)	0.118	0.097	0.222	0.437	0.125	0.101	0.219	0.443	0.070	0.219	0.749	0.982
1-dihomo-linoleoylglycerol (20:2)	0.060	0.109	0.582	0.753	0.066	0.113	0.562	0.755	0.047	0.234	0.841	0.988
1-docosahexaenoylglycerol (22:6)	0.225	0.105	0.033	0.131	0.185	0.111	0.096	0.271	-0.111	0.237	0.642	0.980
1H-indole-7-acetic acid	0.026	0.106	0.805	0.897	-0.020	0.115	0.863	0.941	-0.226	0.257	0.381	0.888
1-lignoceroyl-GPC (24:0)	-0.100	0.068	0.142	0.337	-0.099	0.071	0.163	0.379	-0.029	0.157	0.854	0.988
1-linolenoyl-GPC (18:3)*	0.057	0.080	0.477	0.674	0.044	0.083	0.595	0.765	-0.037	0.178	0.837	0.988
1-linoleoyl-2-arachidonoyl-GPC (18:2/20:4n6)*	0.090	0.083	0.280	0.503	0.059	0.087	0.498	0.711	-0.110	0.186	0.555	0.947
1-linoleoyl-2-linolenoyl-GPC (18:2/18:3)*	0.046	0.076	0.542	0.721	0.010	0.079	0.903	0.955	-0.149	0.168	0.375	0.888
1-linoleoyl-GPA (18:2)*	-0.162	0.085	0.057	0.192	-0.128	0.089	0.150	0.359	0.102	0.192	0.596	0.961
1-linoleoyl-GPC (18:2)	-0.084	0.093	0.368	0.584	-0.076	0.097	0.435	0.658	0.007	0.207	0.973	0.995
1-linoleoyl-GPE (18:2)*	0.046	0.084	0.588	0.756	0.072	0.087	0.408	0.635	0.139	0.187	0.457	0.902
1-linoleoyl-GPI* (18:2)*	0.131	0.076	0.085	0.245	0.141	0.079	0.074	0.224	0.097	0.168	0.567	0.947
1-methyl-4-imidazoleacetate	0.198	0.089	0.028	0.118	0.206	0.093	0.028	0.118	0.109	0.199	0.585	0.957
1-methyl-5-imidazoleacetate	0.017	0.104	0.867	0.930	-0.041	0.108	0.709	0.845	-0.264	0.234	0.260	0.848
1-methyladenosine	0.137	0.096	0.154	0.350	0.184	0.099	0.066	0.208	0.262	0.213	0.219	0.844
1-methylnicotinamide	-0.149	0.078	0.058	0.192	-0.155	0.081	0.058	0.196	-0.083	0.174	0.635	0.976
1-oleoyl-2-linoleoyl-GPE (18:1/18:2)*	0.154	0.084	0.068	0.212	0.140	0.087	0.112	0.294	-0.008	0.187	0.967	0.995
1-oleoylglycerophosphate (18:1)	-0.083	0.106	0.435	0.636	-0.061	0.111	0.584	0.765	0.095	0.259	0.716	0.980
1-oleoyl-GPC (18:1)	-0.167	0.064	0.010	0.055	-0.142	0.067	0.036	0.141	0.054	0.143	0.704	0.980
1-oleoyl-GPE (18:1)	0.071	0.075	0.341	0.557	0.060	0.078	0.443	0.663	-0.026	0.167	0.875	0.992
1-oleoyl-GPG (18:1)*	0.166	0.072	0.023	0.102	0.183	0.076	0.017	0.082	0.132	0.162	0.416	0.893
1-oleoyl-GPI (18:1)*	0.117	0.074	0.117	0.299	0.114	0.077	0.142	0.346	0.029	0.166	0.863	0.991

1-palmitoleoyl-2-linolenoyl-GPC (16:1/18:3)*	0.108	0.077	0.160	0.357	0.110	0.080	0.170	0.384	0.049	0.166	0.770	0.983
1-palmitoleoyl-GPC* (16:1)*	-0.003	0.078	0.973	0.988	0.065	0.081	0.423	0.644	0.309	0.173	0.076	0.819
1-palmitoyl-2-arachidonoyl-GPE (16:0/20:4)*	0.219	0.083	0.009	0.051	0.245	0.086	0.005	0.032	0.198	0.184	0.282	0.869
1-palmitoyl-2-arachidonoyl-GPI (16:0/20:4)*	0.183	0.079	0.022	0.097	0.253	0.081	0.002	0.016	0.387	0.173	0.026	0.738
1-palmitoyl-2-docosahexaenoyl-GPC (16:0/22:6)	0.128	0.069	0.066	0.207	0.117	0.072	0.108	0.289	-0.005	0.155	0.974	0.995
1-palmitoyl-2-docosahexaenoyl-GPE (16:0/22:6)*	0.207	0.080	0.011	0.059	0.240	0.083	0.005	0.030	0.225	0.179	0.209	0.841
1-palmitoyl-2-linoleoyl-GPE (16:0/18:2)	0.193	0.082	0.020	0.093	0.235	0.085	0.006	0.040	0.259	0.182	0.156	0.819
1-palmitoyl-2-linoleoyl-GPI (16:0/18:2)	0.074	0.077	0.340	0.557	0.115	0.080	0.153	0.362	0.215	0.171	0.211	0.841
1-palmitoyl-2-oleoyl-GPC (16:0/18:1)	0.116	0.062	0.062	0.199	0.152	0.064	0.019	0.090	0.204	0.138	0.140	0.819
1-palmitoyl-2-oleoyl-GPI (16:0/18:1)*	0.167	0.073	0.024	0.105	0.218	0.076	0.005	0.030	0.297	0.164	0.071	0.819
1-palmitoyl-2-stearoyl-GPC (16:0/18:0)	0.076	0.083	0.364	0.582	0.142	0.086	0.102	0.281	0.329	0.184	0.076	0.819
1-palmitoyl-GPC (16:0)	0.078	0.094	0.409	0.617	0.155	0.098	0.114	0.298	0.379	0.208	0.070	0.819
1-palmitoyl-GPE (16:0)	0.059	0.086	0.496	0.688	0.101	0.090	0.259	0.490	0.216	0.192	0.262	0.848
1-palmitoyl-GPG (16:0)*	0.171	0.086	0.047	0.164	0.172	0.089	0.054	0.186	0.073	0.186	0.696	0.980
1-palmitoyl-GPI* (16:0)	0.057	0.083	0.490	0.683	0.105	0.087	0.229	0.458	0.229	0.183	0.211	0.841
1-ribosyl-imidazoleacetate*	0.148	0.086	0.089	0.250	0.189	0.089	0.036	0.142	0.241	0.192	0.210	0.841
1-stearoyl-2-docosahexaenoyl-GPC (18:0/22:6)	0.089	0.072	0.217	0.432	0.082	0.075	0.273	0.505	0.002	0.160	0.990	0.998
1-stearoyl-2-linoleoyl-GPC (18:0/18:2)*	0.146	0.078	0.065	0.204	0.152	0.082	0.065	0.207	0.080	0.175	0.648	0.980
1-stearoyl-2-linoleoyl-GPI (18:0/18:2)	0.128	0.080	0.112	0.293	0.153	0.083	0.068	0.210	0.162	0.178	0.365	0.888
1-stearoyl-2-oleoyl-GPC (18:0/18:1)	0.110	0.059	0.064	0.202	0.151	0.061	0.015	0.074	0.224	0.131	0.088	0.819
1-stearoyl-2-oleoyl-GPI (18:0/18:1)*	0.119	0.074	0.109	0.288	0.158	0.077	0.042	0.158	0.217	0.165	0.190	0.839
1-stearoyl-GPC (18:0)	-0.030	0.092	0.743	0.860	0.052	0.096	0.588	0.765	0.366	0.204	0.075	0.819
1-stearoyl-GPE (18:0)	0.091	0.087	0.294	0.517	0.153	0.090	0.091	0.261	0.314	0.192	0.104	0.819
1-stearoyl-GPG (18:0)	-0.087	0.087	0.323	0.542	-0.047	0.092	0.610	0.773	0.161	0.201	0.425	0.893
1-stearoyl-GPI (18:0)	-0.073	0.082	0.376	0.591	-0.052	0.085	0.546	0.744	0.069	0.182	0.705	0.980
2,2'-Methylenebis(6-tert-butyl-p-cresol)	-0.098	0.080	0.222	0.437	-0.074	0.083	0.375	0.615	0.072	0.178	0.684	0.980
2,3-dihydroxy-2-methylbutyrate	0.151	0.121	0.215	0.427	0.178	0.120	0.142	0.346	0.226	0.280	0.422	0.893
2,3-dihydroxyisovalerate	-0.012	0.088	0.895	0.945	-0.032	0.091	0.727	0.858	-0.097	0.196	0.619	0.972
21-hydroxypregnenolone disulfate	-0.138	0.076	0.070	0.212	-0.194	0.079	0.014	0.074	-0.303	0.167	0.072	0.819
2-aminobutyrate	0.152	0.072	0.036	0.136	0.139	0.075	0.066	0.208	-0.004	0.160	0.982	0.997
2-aminooctanoate	0.019	0.080	0.812	0.903	-0.033	0.083	0.695	0.838	-0.229	0.178	0.199	0.841
2-aminophenol sulfate	-0.016	0.075	0.833	0.918	0.025	0.081	0.757	0.880	0.175	0.169	0.302	0.873
2-butenoylglycine	0.115	0.150	0.445	0.647	0.146	0.169	0.390	0.622	0.167	0.420	0.692	0.980

2'-deoxyuridine	-0.114	0.084	0.180	0.384	-0.143	0.088	0.108	0.289	-0.166	0.185	0.371	0.888
2-ethylphenylsulfate	0.011	0.103	0.912	0.953	-0.065	0.105	0.537	0.742	-0.456	0.255	0.077	0.819
2-hydroxy-3-methylvalerate	0.084	0.076	0.267	0.486	0.099	0.079	0.209	0.434	0.099	0.169	0.558	0.947
2-hydroxybutyrate/2-hydroxyisobutyrate	0.191	0.081	0.020	0.094	0.170	0.085	0.047	0.169	-0.024	0.181	0.894	0.992
2-hydroxydecanoate	0.056	0.075	0.458	0.658	0.008	0.078	0.920	0.959	-0.198	0.167	0.236	0.845
2-hydroxyfluorene sulfate	-0.108	0.064	0.092	0.258	-0.106	0.066	0.112	0.295	-0.030	0.142	0.831	0.988
2-hydroxyglutarate	0.056	0.103	0.591	0.760	-0.012	0.107	0.912	0.959	-0.237	0.210	0.260	0.848
2-hydroxyheptanoate*	-0.123	0.089	0.169	0.371	-0.124	0.093	0.185	0.405	-0.047	0.199	0.815	0.988
2-hydroxyhippurate (salicylurate)	0.091	0.087	0.293	0.517	0.111	0.091	0.221	0.445	0.121	0.192	0.529	0.942
2-hydroxylaurate	0.041	0.073	0.572	0.745	-0.007	0.076	0.923	0.961	-0.208	0.163	0.203	0.841
2-hydroxynervonate*	0.069	0.043	0.105	0.281	0.061	0.044	0.172	0.387	-0.014	0.095	0.887	0.992
2-hydroxyoctanoate	0.119	0.080	0.142	0.337	0.073	0.084	0.390	0.622	-0.168	0.179	0.351	0.885
2-hydroxypalmitate	0.026	0.088	0.765	0.877	0.009	0.091	0.919	0.959	-0.068	0.195	0.730	0.980
2-hydroxystearate	-0.132	0.092	0.152	0.348	-0.133	0.096	0.168	0.382	-0.050	0.205	0.809	0.988
2-isopropylmalate	0.171	0.106	0.109	0.288	0.175	0.109	0.109	0.289	0.095	0.221	0.670	0.980
2-keto-3-deoxy-gluconate	0.176	0.082	0.033	0.131	0.170	0.085	0.048	0.170	0.038	0.182	0.833	0.988
2-methylcitrate/homocitrate	0.154	0.081	0.059	0.195	0.085	0.084	0.311	0.551	-0.248	0.177	0.164	0.819
2-methylmalonylcarnitine (C4-DC)	0.186	0.098	0.060	0.196	0.151	0.102	0.141	0.344	-0.088	0.218	0.686	0.980
2-naphthol sulfate	0.021	0.070	0.768	0.877	0.020	0.073	0.783	0.898	0.005	0.157	0.975	0.995
2-oleoylglycerol (18:1)	0.166	0.076	0.030	0.122	0.184	0.079	0.021	0.096	0.139	0.167	0.408	0.893
2'-O-methylcytidine	-0.137	0.087	0.117	0.301	-0.111	0.091	0.222	0.446	0.066	0.193	0.734	0.981
2'-O-methyluridine	-0.089	0.077	0.251	0.471	-0.049	0.080	0.536	0.742	0.132	0.165	0.425	0.893
2-palmitoleoylglycerol (16:1)*	0.095	0.122	0.440	0.641	0.112	0.126	0.379	0.618	0.120	0.285	0.675	0.980
2-palmitoleoyl-GPC* (16:1)*	0.166	0.100	0.101	0.276	0.192	0.104	0.068	0.210	0.174	0.215	0.419	0.893
2-palmitoyl-GPC* (16:0)*	-0.002	0.084	0.978	0.990	0.061	0.088	0.486	0.701	0.288	0.186	0.124	0.819
2-piperidinone	-0.096	0.084	0.251	0.471	-0.059	0.088	0.504	0.716	0.138	0.187	0.461	0.902
2-stearoyl-GPE (18:0)*	0.097	0.086	0.264	0.485	0.164	0.090	0.069	0.212	0.341	0.191	0.076	0.819
3-(3-amino-3-carboxypropyl)uridine*	0.219	0.096	0.024	0.105	0.248	0.099	0.014	0.070	0.221	0.217	0.310	0.873
3-(3-hydroxyphenyl)propionate	-0.155	0.105	0.142	0.337	-0.154	0.102	0.134	0.331	-0.094	0.211	0.657	0.980
3-(4-hydroxyphenyl)lactate (HPLA)	0.151	0.070	0.033	0.131	0.147	0.073	0.046	0.168	0.037	0.156	0.815	0.988
3-(methylthio)acetaminophen sulfate*	-0.217	0.502	0.687	0.818	-0.057	0.507	0.916	0.959	1.260	1.522	0.469	0.902
3,4-methyleneheptanoate	0.056	0.080	0.490	0.683	-0.011	0.084	0.897	0.952	-0.279	0.177	0.118	0.819
3,4-methyleneheptanoylcarnitine	0.144	0.108	0.186	0.392	0.016	0.115	0.892	0.949	-0.487	0.228	0.035	0.761

3-acetylphenol sulfate	0.130	0.116	0.265	0.485	0.083	0.121	0.498	0.711	-0.209	0.278	0.455	0.902
3-amino-2-piperidone	0.100	0.092	0.278	0.501	0.111	0.095	0.248	0.477	0.087	0.206	0.674	0.980
3-aminoisobutyrate	0.119	0.088	0.178	0.384	0.182	0.091	0.047	0.170	0.331	0.195	0.091	0.819
3beta,7alpha-dihydroxy-5-cholestenoate	-0.012	0.071	0.869	0.930	-0.088	0.074	0.236	0.466	-0.352	0.157	0.026	0.738
3-carboxy-4-methyl-5-pentyl-2-furanpropionate (3-CMPFP)	0.150	0.071	0.037	0.140	0.136	0.075	0.070	0.214	-0.008	0.159	0.960	0.994
3-carboxy-4-methyl-5-propyl-2-furanpropanoate (CMPF)	0.095	0.053	0.077	0.227	0.091	0.056	0.102	0.281	0.019	0.119	0.877	0.992
3-ethylcatechol sulfate (1)	0.059	0.111	0.599	0.766	0.039	0.121	0.747	0.871	-0.116	0.300	0.700	0.980
3-ethylphenylsulfate	0.293	0.175	0.107	0.286	0.159	0.179	0.383	0.620	-0.605	0.407	0.150	0.819
3-formylindole	0.059	0.085	0.491	0.684	0.047	0.089	0.594	0.765	-0.031	0.190	0.872	0.992
3-hydroxy-3-methylglutarate	0.124	0.094	0.187	0.393	0.086	0.097	0.374	0.615	-0.126	0.209	0.547	0.947
3-hydroxybutyrate (BHBA)	0.007	0.080	0.927	0.963	0.033	0.083	0.696	0.838	0.118	0.178	0.509	0.930
3-hydroxybutyroylglycine	-0.071	0.090	0.432	0.635	-0.086	0.095	0.364	0.602	-0.089	0.195	0.650	0.980
3-hydroxybutyrylcarnitine (1)	0.167	0.109	0.129	0.321	0.163	0.113	0.152	0.361	0.045	0.249	0.856	0.988
3-hydroxybutyrylcarnitine (2)	0.116	0.078	0.139	0.335	0.121	0.081	0.139	0.342	0.065	0.174	0.711	0.980
3-hydroxydecanoate	0.017	0.084	0.843	0.920	0.032	0.088	0.720	0.852	0.074	0.188	0.695	0.980
3-hydroxydodecanedioate*	-0.060	0.126	0.635	0.788	0.006	0.133	0.962	0.976	0.257	0.271	0.346	0.885
3-hydroxyhexanoate	0.163	0.086	0.062	0.199	0.188	0.090	0.037	0.143	0.177	0.192	0.360	0.887
3-hydroxyhippurate	-0.116	0.084	0.169	0.371	-0.053	0.087	0.544	0.744	0.227	0.181	0.211	0.841
3-hydroxylaurate	-0.070	0.083	0.401	0.611	-0.056	0.087	0.518	0.730	0.037	0.185	0.840	0.988
3-hydroxyoctanoate	0.109	0.082	0.187	0.393	0.112	0.086	0.193	0.416	0.053	0.183	0.772	0.984
3-hydroxyoleoylcarnitine	0.067	0.079	0.396	0.605	0.109	0.082	0.182	0.402	0.218	0.174	0.214	0.841
3-hydroxypalmitoylcarnitine	0.273	0.108	0.013	0.066	0.341	0.107	0.002	0.014	0.430	0.222	0.056	0.819
3-hydroxypyridine sulfate	0.072	0.072	0.321	0.542	0.031	0.075	0.681	0.830	-0.169	0.164	0.304	0.873
3-hydroxysebacate	0.091	0.103	0.376	0.591	0.063	0.104	0.546	0.744	-0.088	0.225	0.697	0.980
3-hydroxystachydrine*	0.151	0.113	0.187	0.393	0.115	0.116	0.324	0.566	-0.122	0.266	0.649	0.980
3-indoxyl sulfate	-0.041	0.086	0.635	0.788	0.012	0.090	0.891	0.949	0.228	0.191	0.234	0.844
3-methoxycatechol sulfate (1)	-0.088	0.091	0.334	0.554	-0.117	0.093	0.213	0.437	-0.171	0.203	0.402	0.893
3-methoxycatechol sulfate (2)	0.296	0.126	0.021	0.094	0.304	0.134	0.025	0.110	0.125	0.274	0.649	0.980
3-methoxytyramine sulfate	0.098	0.120	0.414	0.618	0.113	0.121	0.355	0.591	0.105	0.241	0.665	0.980
3-methoxytyrosine	-0.050	0.082	0.543	0.721	0.042	0.086	0.626	0.782	0.395	0.180	0.029	0.738
3-methyl catechol sulfate (1)	0.023	0.069	0.733	0.855	-0.004	0.071	0.951	0.971	-0.122	0.154	0.432	0.898
3-methyl-2-oxobutyrate	0.130	0.074	0.082	0.239	0.155	0.077	0.046	0.168	0.160	0.165	0.333	0.880
3-methyladipate	0.049	0.114	0.666	0.806	-0.038	0.122	0.758	0.880	-0.475	0.284	0.098	0.819

3-methylglutaconate	0.081	0.091	0.375	0.591	0.061	0.095	0.520	0.731	-0.062	0.203	0.763	0.982
3-methylglutarylcarnitine (2)	0.081	0.093	0.387	0.598	0.058	0.096	0.547	0.745	-0.081	0.214	0.706	0.980
3-methylhistidine	0.049	0.102	0.630	0.785	-0.058	0.107	0.587	0.765	-0.417	0.213	0.052	0.819
3-methylxanthine	0.191	0.190	0.321	0.542	0.309	0.204	0.141	0.344	0.632	0.472	0.191	0.839
3-phenylpropionate (hydrocinnamate)	-0.141	0.085	0.101	0.276	-0.021	0.090	0.814	0.914	0.494	0.187	0.009	0.738
3-phosphoglycerate	0.072	0.075	0.341	0.557	0.066	0.078	0.404	0.633	-0.003	0.168	0.986	0.998
3-ureidopropionate	0.246	0.108	0.025	0.109	0.217	0.114	0.060	0.200	-0.064	0.252	0.801	0.988
4-acetamidobutanoate	0.197	0.092	0.034	0.133	0.187	0.096	0.053	0.186	0.028	0.205	0.892	0.992
4-acetamidophenol	-0.145	0.200	0.543	0.721	-0.110	0.235	0.686	0.832	2.010	0.004	0.001	0.614
4-acetamidophenylglucuronide	-0.408	0.252	0.180	0.384	-0.335	0.361	0.406	0.635	0.803	0.773	0.376	0.888
4-acetaminophen sulfate	-0.313	0.305	0.326	0.544	-0.196	0.395	0.629	0.784	0.693	0.831	0.422	0.893
4-acetylphenyl sulfate	0.043	0.093	0.644	0.793	0.005	0.099	0.963	0.976	-0.182	0.221	0.412	0.893
4-allylphenol sulfate	0.083	0.071	0.245	0.464	0.098	0.074	0.191	0.412	0.096	0.160	0.550	0.947
4-ethylphenyl sulfate	0.086	0.087	0.320	0.542	0.130	0.090	0.149	0.359	0.231	0.192	0.231	0.844
4-hydroxy-2-oxoglutaric acid	-0.021	0.074	0.778	0.881	-0.067	0.078	0.390	0.622	-0.221	0.167	0.189	0.839
4-hydroxychlorothalonil	0.001	0.080	0.985	0.990	-0.031	0.083	0.708	0.845	-0.145	0.175	0.408	0.893
4-hydroxyhippurate	0.041	0.082	0.619	0.778	0.016	0.086	0.852	0.936	-0.099	0.183	0.590	0.961
4-hydroxyphenylacetate	0.103	0.090	0.256	0.475	0.053	0.093	0.572	0.758	-0.192	0.201	0.341	0.885
4-hydroxyphenylacetylglutamine	0.104	0.113	0.359	0.574	0.023	0.117	0.842	0.933	-0.470	0.289	0.107	0.819
4-hydroxyphenylpyruvate	0.120	0.082	0.146	0.342	0.070	0.086	0.415	0.643	-0.183	0.183	0.318	0.875
4-methoxyphenol sulfate	0.033	0.075	0.664	0.806	0.062	0.078	0.430	0.651	0.143	0.166	0.389	0.889
4-methyl-2-oxopentanoate	0.195	0.083	0.020	0.094	0.189	0.087	0.031	0.125	0.046	0.186	0.804	0.988
4-methylcatechol sulfate	-0.067	0.078	0.393	0.603	-0.013	0.081	0.877	0.944	0.223	0.173	0.200	0.841
4-methylguaiacol sulfate	0.007	0.098	0.943	0.972	0.015	0.103	0.882	0.944	0.042	0.226	0.855	0.988
4-vinylguaiacol sulfate	0.183	0.084	0.031	0.126	0.182	0.087	0.037	0.142	0.069	0.195	0.723	0.980
4-vinylphenol sulfate	0.090	0.072	0.212	0.425	0.050	0.076	0.508	0.721	-0.152	0.161	0.347	0.885
5-(galactosylhydroxy)-L-lysine	0.283	0.120	0.020	0.093	0.238	0.121	0.052	0.181	-0.078	0.270	0.774	0.984
5,6-dihydrothymine	0.003	0.076	0.966	0.987	0.037	0.080	0.641	0.794	0.156	0.170	0.361	0.887
5,6-dihydrouracil	-0.035	0.080	0.665	0.806	-0.035	0.083	0.679	0.830	-0.012	0.178	0.947	0.993
5,6-dihydrouridine	0.042	0.085	0.622	0.779	0.042	0.089	0.641	0.794	0.013	0.190	0.948	0.993
5-acetylamino-6-amino-3-methyluracil	0.164	0.119	0.175	0.379	0.162	0.124	0.196	0.420	0.045	0.297	0.880	0.992
5-acetylamino-6-formylamino-3-methyluracil	0.157	0.121	0.201	0.411	0.188	0.132	0.161	0.377	0.194	0.342	0.574	0.947
5alpha-androstan-3alpha,17beta-diol disulfate	0.066	0.138	0.636	0.788	0.005	0.122	0.968	0.980	-0.193	0.251	0.444	0.902

5alpha-androstan-3alpha,17beta-diol monosulfate (1)	0.106	0.071	0.138	0.334	0.070	0.074	0.348	0.587	-0.127	0.159	0.425	0.893
5alpha-androstan-3alpha,17beta-diol monosulfate (2)	0.036	0.213	0.867	0.930	-0.046	0.194	0.812	0.914	-0.330	0.409	0.423	0.893
5alpha-androstan-3beta,17alpha-diol disulfate	-0.049	0.137	0.720	0.846	-0.018	0.133	0.893	0.949	0.097	0.270	0.722	0.980
5alpha-androstan-3beta,17beta-diol disulfate	0.084	0.051	0.101	0.276	0.051	0.053	0.339	0.579	-0.119	0.113	0.296	0.873
5alpha-androstan-3beta,17beta-diol monosulfate (2)	0.034	0.091	0.711	0.840	0.015	0.089	0.867	0.943	-0.064	0.189	0.737	0.981
5alpha-pregnan-3beta,20alpha-diol disulfate	-0.124	0.079	0.118	0.302	-0.163	0.082	0.049	0.174	-0.221	0.176	0.212	0.841
5alpha-pregnan-3beta,20alpha-diol monosulfate (2)	-0.177	0.077	0.022	0.097	-0.231	0.080	0.004	0.028	-0.306	0.171	0.076	0.819
5alpha-pregnan-3beta,20beta-diol monosulfate (1)	-0.114	0.080	0.153	0.350	-0.177	0.082	0.032	0.130	-0.325	0.174	0.063	0.819
5alpha-pregnan-diol disulfate	0.092	0.115	0.425	0.630	-0.024	0.119	0.838	0.930	-0.387	0.225	0.088	0.819
5-dodecenoate (12:1n7)	-0.146	0.086	0.090	0.254	-0.114	0.089	0.205	0.428	0.094	0.191	0.624	0.972
5-dodecenoylcarnitine (C12:1)	0.053	0.083	0.520	0.708	0.062	0.086	0.470	0.686	0.060	0.184	0.745	0.982
5-HEPE	-0.206	0.206	0.325	0.543	0.006	0.256	0.980	0.989	0.833	0.473	0.088	0.819
5-hydroxyhexanoate	0.103	0.089	0.251	0.471	0.130	0.092	0.161	0.376	0.164	0.197	0.407	0.893
5-methyluridine (ribothymidine)	0.019	0.080	0.810	0.901	0.027	0.083	0.744	0.870	0.044	0.179	0.808	0.988
5-oxoproline	0.003	0.082	0.967	0.988	0.045	0.085	0.595	0.765	0.193	0.182	0.290	0.873
6-bromotryptophan	-0.051	0.077	0.508	0.700	-0.024	0.081	0.769	0.888	0.107	0.172	0.534	0.943
6-hydroxyindole sulfate	-0.027	0.087	0.758	0.873	0.031	0.089	0.727	0.858	0.251	0.190	0.187	0.839
7-HOCA	0.037	0.073	0.612	0.775	0.061	0.075	0.416	0.643	0.126	0.161	0.437	0.900
7-methylguanine	0.008	0.077	0.920	0.957	0.060	0.080	0.456	0.676	0.240	0.170	0.161	0.819
8-hydroxyoctanoate	-0.099	0.081	0.227	0.444	-0.170	0.084	0.046	0.168	-0.381	0.186	0.043	0.797
9,10-DiHOME	-0.084	0.076	0.271	0.491	-0.061	0.080	0.444	0.663	0.074	0.170	0.665	0.980
9-hydroxystearate	-0.121	0.075	0.107	0.286	-0.128	0.078	0.102	0.281	-0.076	0.167	0.648	0.980
acesulfame	-0.505	0.339	0.160	0.357	-0.528	0.366	0.173	0.388	-0.234	0.733	0.755	0.982
acetylcarnitine (C2)	0.049	0.083	0.557	0.734	0.067	0.086	0.437	0.660	0.102	0.185	0.583	0.957
acisoga	-0.002	0.083	0.982	0.990	0.048	0.086	0.581	0.762	0.225	0.184	0.222	0.844
aconitate [cis or trans]	0.161	0.079	0.044	0.156	0.237	0.082	0.004	0.027	0.409	0.174	0.020	0.738
adenine	0.014	0.088	0.875	0.932	0.028	0.092	0.761	0.882	0.067	0.194	0.729	0.980
adipoylcarnitine (C6-DC)	0.190	0.080	0.019	0.090	0.174	0.084	0.039	0.147	-0.003	0.179	0.987	0.998
adrenate (22:4n6)	-0.020	0.090	0.820	0.909	-0.027	0.093	0.774	0.891	-0.037	0.200	0.854	0.988
adrenoylcarnitine (C22:4)*	-0.053	0.076	0.490	0.683	0.043	0.080	0.592	0.765	0.424	0.169	0.013	0.738
allantoin	0.054	0.081	0.512	0.701	0.058	0.085	0.498	0.711	0.038	0.182	0.834	0.988
alliin	0.147	0.089	0.101	0.276	0.151	0.091	0.100	0.279	0.082	0.195	0.675	0.980
alpha-hydroxycaproate	-0.009	0.087	0.914	0.953	0.011	0.090	0.900	0.954	0.090	0.192	0.641	0.980

alpha-hydroxyisocaproate	0.038	0.077	0.619	0.778	0.013	0.080	0.872	0.944	-0.102	0.172	0.553	0.947
alpha-hydroxyisovalerate	-0.035	0.071	0.624	0.780	-0.045	0.074	0.543	0.744	-0.060	0.159	0.709	0.980
alpha-ketobutyrate	0.196	0.092	0.036	0.136	0.152	0.093	0.106	0.286	-0.125	0.210	0.553	0.947
alpha-ketoglutaramate*	0.067	0.085	0.429	0.632	0.013	0.088	0.882	0.944	-0.222	0.188	0.239	0.847
alpha-ketoglutarate	-0.025	0.082	0.761	0.874	-0.005	0.085	0.949	0.971	0.080	0.182	0.662	0.980
alpha-tocopherol	0.033	0.090	0.713	0.842	0.034	0.094	0.720	0.852	0.015	0.200	0.942	0.993
AMP	0.046	0.073	0.532	0.713	0.042	0.076	0.578	0.761	0.001	0.162	0.995	0.999
andro steroid monosulfate C19H28O6S (1)*	0.092	0.084	0.275	0.497	0.035	0.089	0.695	0.838	-0.221	0.185	0.235	0.844
androstenediol (3alpha, 17alpha) monosulfate (2)	0.031	0.074	0.679	0.812	0.047	0.078	0.544	0.744	0.084	0.163	0.610	0.967
androstenediol (3alpha, 17alpha) monosulfate (3)	-0.083	0.053	0.119	0.303	-0.105	0.055	0.057	0.193	-0.133	0.118	0.261	0.848
androstenediol (3beta,17beta) disulfate (1)	0.131	0.057	0.021	0.096	0.097	0.059	0.105	0.285	-0.110	0.126	0.384	0.889
androstenediol (3beta,17beta) disulfate (2)	-0.057	0.061	0.347	0.563	-0.088	0.063	0.165	0.381	-0.161	0.135	0.234	0.844
androstenediol (3beta,17beta) monosulfate (1)	0.082	0.062	0.186	0.392	0.029	0.065	0.652	0.804	-0.212	0.137	0.124	0.819
androstenediol (3beta,17beta) monosulfate (2)	-0.010	0.071	0.885	0.938	-0.056	0.074	0.452	0.672	-0.212	0.158	0.182	0.839
androsterone glucuronide	0.096	0.074	0.197	0.406	0.030	0.077	0.694	0.838	-0.242	0.158	0.127	0.819
androsterone sulfate	-0.150	0.065	0.022	0.097	-0.194	0.067	0.004	0.029	-0.253	0.144	0.080	0.819
arabitol/xylitol	0.193	0.090	0.032	0.130	0.125	0.094	0.186	0.405	-0.241	0.199	0.227	0.844
arabonate/xylonate	0.199	0.086	0.021	0.096	0.149	0.090	0.099	0.278	-0.155	0.191	0.416	0.893
arachidate (20:0)	0.073	0.075	0.332	0.552	0.063	0.078	0.418	0.643	-0.017	0.167	0.920	0.992
arachidonate (20:4n6)	-0.003	0.088	0.971	0.988	-0.009	0.091	0.924	0.961	-0.027	0.196	0.891	0.992
arachidonoylcarnitine (C20:4)	-0.085	0.077	0.267	0.486	-0.017	0.080	0.830	0.924	0.280	0.170	0.101	0.819
arachidonoylcholine	-0.088	0.081	0.277	0.500	-0.121	0.084	0.150	0.359	-0.184	0.180	0.308	0.873
arachidoylcarnitine (C20)*	-0.066	0.080	0.413	0.618	-0.019	0.084	0.820	0.919	0.196	0.181	0.282	0.869
arginine	0.072	0.085	0.396	0.605	0.072	0.088	0.417	0.643	0.025	0.189	0.897	0.992
asparagine	-0.086	0.095	0.370	0.587	-0.110	0.099	0.268	0.502	-0.144	0.212	0.499	0.918
azelate (nonanedioate; C9)	0.066	0.077	0.388	0.598	0.025	0.080	0.755	0.879	-0.164	0.170	0.336	0.883
behenoyl sphingomyelin (d18:1/22:0)*	0.122	0.055	0.029	0.121	0.125	0.058	0.032	0.128	0.058	0.123	0.636	0.976
behenoylcarnitine (C22)*	0.125	0.085	0.142	0.337	0.135	0.087	0.124	0.315	0.096	0.185	0.604	0.963
benzoate	-0.147	0.082	0.074	0.220	-0.163	0.085	0.057	0.193	-0.128	0.183	0.485	0.905
beta-cryptoxanthin	-0.023	0.079	0.774	0.881	-0.087	0.082	0.289	0.525	-0.303	0.175	0.085	0.819
beta-sitosterol	-0.190	0.077	0.015	0.074	-0.204	0.080	0.012	0.063	-0.132	0.170	0.438	0.900
bradykinin	3.141	1.852	0.339	0.557	1.020	0.578	0.328	0.569	34.000	NA	NA	NA
bradykinin, des-arg(9)	0.302	0.354	0.413	0.618	0.491	0.329	0.166	0.381	0.647	0.544	0.264	0.848

caffeic acid sulfate	-0.128	0.087	0.142	0.337	-0.142	0.092	0.126	0.321	-0.098	0.198	0.623	0.972
caffeine	0.220	0.120	0.070	0.212	0.161	0.125	0.203	0.427	-0.265	0.298	0.377	0.888
campesterol	-0.171	0.085	0.045	0.158	-0.159	0.090	0.079	0.235	0.003	0.191	0.987	0.998
caprate (10:0)	0.028	0.081	0.729	0.854	0.006	0.084	0.944	0.969	-0.090	0.180	0.616	0.972
caproate (6:0)	-0.104	0.074	0.160	0.357	-0.151	0.076	0.049	0.174	-0.253	0.163	0.122	0.819
caprylate (8:0)	0.025	0.083	0.759	0.873	-0.019	0.086	0.828	0.923	-0.192	0.184	0.298	0.873
carboxyethyl-GABA	0.106	0.089	0.237	0.454	0.079	0.094	0.398	0.629	-0.071	0.191	0.709	0.980
carnitine	0.150	0.083	0.074	0.219	0.162	0.087	0.063	0.204	0.112	0.186	0.546	0.947
carotene diol (1)	-0.102	0.077	0.189	0.395	-0.095	0.080	0.240	0.471	-0.005	0.172	0.978	0.996
carotene diol (3)	-0.123	0.103	0.236	0.452	-0.094	0.108	0.388	0.622	0.084	0.226	0.712	0.980
catechol sulfate	0.027	0.074	0.715	0.842	0.143	0.076	0.061	0.201	0.540	0.159	0.001	0.614
ceramide (d18:2/24:1, d18:1/24:2)*	0.075	0.062	0.230	0.447	0.067	0.065	0.304	0.544	-0.010	0.139	0.943	0.993
cerotoylcarnitine (C26)*	0.107	0.062	0.086	0.245	0.166	0.064	0.010	0.054	0.312	0.136	0.023	0.738
C-glycosyltryptophan	0.132	0.091	0.147	0.343	0.169	0.094	0.074	0.224	0.218	0.201	0.281	0.869
chenodeoxycholate	-0.017	0.082	0.836	0.920	-0.044	0.085	0.603	0.769	-0.131	0.182	0.473	0.902
cholate	-0.010	0.083	0.906	0.952	-0.023	0.087	0.792	0.903	-0.063	0.186	0.734	0.981
cholesterol	0.182	0.086	0.036	0.136	0.191	0.090	0.035	0.138	0.104	0.192	0.588	0.959
choline	0.143	0.078	0.070	0.212	0.183	0.081	0.025	0.108	0.238	0.173	0.171	0.831
cinnamoylglycine	-0.168	0.092	0.071	0.212	-0.070	0.096	0.470	0.686	0.363	0.198	0.069	0.819
cis-4-decenoate (10:1n6)*	0.052	0.079	0.506	0.699	0.054	0.082	0.510	0.723	0.027	0.175	0.879	0.992
cis-4-decenoylcarnitine (C10:1)	0.043	0.079	0.586	0.755	0.047	0.082	0.570	0.758	0.033	0.176	0.854	0.988
citramalate	0.214	0.121	0.082	0.239	0.318	0.134	0.021	0.094	0.499	0.295	0.095	0.819
citrate	-0.143	0.082	0.083	0.239	-0.081	0.086	0.350	0.587	0.233	0.182	0.202	0.841
citrulline	-0.087	0.087	0.321	0.542	-0.085	0.091	0.351	0.588	-0.023	0.195	0.906	0.992
corticosterone	-0.081	0.079	0.306	0.528	-0.140	0.081	0.086	0.250	-0.307	0.175	0.082	0.819
cotinine	-0.050	0.070	0.475	0.673	-0.095	0.073	0.194	0.417	-0.240	0.162	0.142	0.819
creatine	0.114	0.078	0.144	0.340	0.102	0.081	0.210	0.434	-0.013	0.173	0.938	0.993
creatinine	0.180	0.082	0.029	0.121	0.194	0.085	0.024	0.105	0.130	0.182	0.476	0.902
cys-gly, oxidized	0.005	0.081	0.954	0.978	0.049	0.084	0.558	0.754	0.206	0.180	0.254	0.848
cysteine	-0.008	0.090	0.932	0.967	-0.050	0.094	0.591	0.765	-0.197	0.200	0.326	0.877
cysteine s-sulfate	0.057	0.082	0.488	0.683	0.120	0.086	0.162	0.377	0.307	0.182	0.094	0.819
cysteine sulfinic acid	0.059	0.082	0.475	0.673	0.096	0.087	0.268	0.502	0.191	0.187	0.308	0.873
cysteinylglycine	-0.052	0.082	0.529	0.713	-0.007	0.085	0.930	0.962	0.183	0.182	0.316	0.875

cysteinylglycine disulfide*	0.099	0.078	0.205	0.417	0.144	0.081	0.077	0.233	0.239	0.173	0.168	0.826
cystine	0.056	0.087	0.522	0.709	0.124	0.089	0.165	0.381	0.304	0.180	0.093	0.819
cytidine	-0.094	0.089	0.292	0.516	-0.110	0.093	0.239	0.470	-0.105	0.201	0.602	0.963
daidzein sulfate (2)	0.115	0.112	0.308	0.530	-0.007	0.116	0.952	0.971	-0.537	0.249	0.033	0.738
decanoylcarnitine (C10)	-0.040	0.086	0.643	0.792	-0.022	0.090	0.805	0.910	0.067	0.192	0.728	0.980
dehydroisoandrosterone sulfate (DHEA-S)	-0.069	0.058	0.234	0.450	-0.137	0.060	0.023	0.101	-0.336	0.127	0.009	0.738
delta-CEHC glucuronide*	0.133	0.163	0.418	0.623	0.025	0.162	0.879	0.944	-0.357	0.322	0.272	0.857
delta-CEHC*	0.051	0.068	0.453	0.655	0.042	0.072	0.560	0.755	-0.030	0.159	0.849	0.988
delta-tocopherol	-0.070	0.112	0.532	0.713	-0.025	0.115	0.826	0.922	0.195	0.257	0.451	0.902
deoxycarnitine	-0.035	0.066	0.594	0.762	0.005	0.068	0.945	0.969	0.169	0.146	0.249	0.848
deoxycholate	0.041	0.081	0.616	0.776	0.083	0.085	0.334	0.574	0.206	0.183	0.263	0.848
diacylglycerol (14:0/18:1, 16:0/16:1) [1]*	0.238	0.107	0.028	0.118	0.289	0.113	0.012	0.061	0.286	0.229	0.214	0.841
dihomolinoleate (20:2n6)	-0.089	0.085	0.298	0.521	-0.086	0.089	0.332	0.573	-0.021	0.190	0.911	0.992
dihomolinolenate (20:3n3 or 3n6)	0.031	0.087	0.726	0.852	0.055	0.091	0.546	0.744	0.122	0.194	0.530	0.942
dihomo-linolenoylcarnitine (C20:3n3 or 6)*	-0.036	0.077	0.645	0.793	0.065	0.080	0.416	0.643	0.448	0.169	0.009	0.738
dihomo-linolenoyl-choline	0.020	0.086	0.817	0.907	0.021	0.089	0.812	0.914	0.014	0.184	0.941	0.993
dihomo-linoleoylcarnitine (C20:2)*	-0.058	0.073	0.432	0.635	0.025	0.076	0.742	0.870	0.357	0.161	0.028	0.738
dihydrocaffeate sulfate (2)	-0.067	0.089	0.453	0.655	-0.018	0.092	0.848	0.935	0.207	0.200	0.302	0.873
dihydroferulate	0.076	0.111	0.496	0.689	0.089	0.107	0.408	0.635	0.127	0.255	0.620	0.972
dihydroferulic acid sulfate	0.053	0.114	0.646	0.793	0.074	0.118	0.535	0.742	0.140	0.287	0.627	0.973
dihydroorotate	0.106	0.088	0.229	0.447	0.094	0.091	0.304	0.544	-0.014	0.195	0.943	0.993
dimethylarginine (ADMA + SDMA)	0.169	0.092	0.067	0.209	0.169	0.095	0.079	0.235	0.060	0.204	0.770	0.983
docosadienoate (22:2n6)	0.080	0.065	0.221	0.437	0.059	0.068	0.383	0.620	-0.065	0.145	0.655	0.980
docosadioate (C22-DC)	0.055	0.067	0.414	0.618	0.004	0.070	0.956	0.973	-0.214	0.149	0.154	0.819
docosahexaenoate (DHA; 22:6n3)	-0.062	0.072	0.385	0.598	-0.066	0.075	0.379	0.618	-0.038	0.160	0.810	0.988
docosahexaenoylcarnitine (C22:6)*	-0.078	0.082	0.343	0.559	-0.073	0.086	0.397	0.628	0.000	0.191	0.999	0.999
docosahexaenoylcholine	-0.067	0.083	0.426	0.630	-0.096	0.087	0.273	0.505	-0.155	0.185	0.403	0.893
docosapentaenoate (DPA; 22:5n3)	-0.022	0.075	0.767	0.877	-0.029	0.078	0.714	0.848	-0.037	0.167	0.824	0.988
docosapentaenoate (n6 DPA; 22:5n6)	-0.076	0.079	0.335	0.554	-0.077	0.082	0.350	0.587	-0.031	0.176	0.862	0.991
docosapentaenoylcarnitine (C22:5n3)*	-0.044	0.078	0.572	0.745	0.021	0.082	0.797	0.903	0.313	0.181	0.087	0.819
docosatrienoate (22:3n3)	0.002	0.079	0.979	0.990	-0.002	0.082	0.981	0.989	-0.018	0.178	0.919	0.992
docosatrienoate (22:3n6)*	0.110	0.116	0.344	0.559	0.123	0.121	0.312	0.552	0.100	0.274	0.717	0.980
dodecadienoate (12:2)*	-0.054	0.082	0.512	0.701	-0.045	0.086	0.602	0.769	0.023	0.183	0.902	0.992

dodecanedioate (C12)	0.024	0.084	0.777	0.881	-0.009	0.087	0.921	0.960	-0.139	0.186	0.456	0.902
dodecenedioate (C12:1-DC)*	-0.071	0.085	0.403	0.612	-0.101	0.089	0.254	0.483	-0.163	0.190	0.391	0.891
dopamine 3-O-sulfate	0.107	0.075	0.157	0.353	0.089	0.079	0.260	0.490	-0.045	0.169	0.789	0.988
DSGEGDFXAEGGGVR*	0.523	0.288	0.119	0.303	0.603	0.448	0.227	0.455	-1.550	1.499	0.348	0.885
ectoine	0.163	0.081	0.047	0.163	0.188	0.085	0.028	0.118	0.172	0.185	0.354	0.885
EDTA	0.030	0.066	0.651	0.797	0.013	0.069	0.852	0.936	-0.068	0.148	0.649	0.980
eicosanedioate (C20-DC)	0.000	0.076	0.999	0.999	-0.031	0.079	0.695	0.838	-0.141	0.168	0.402	0.893
eicosapentaenoate (EPA; 20:5n3)	0.079	0.074	0.287	0.510	0.057	0.077	0.462	0.681	-0.072	0.165	0.663	0.980
eicosenamide (20:1)*	0.039	0.080	0.624	0.780	0.009	0.084	0.919	0.959	-0.126	0.178	0.480	0.902
eicosenedioate (C20:1-DC)*	-0.004	0.063	0.953	0.977	-0.042	0.066	0.528	0.735	-0.165	0.137	0.229	0.844
eicosenoate (20:1n9 or 1n11)	-0.027	0.061	0.658	0.803	-0.031	0.064	0.633	0.788	-0.025	0.137	0.855	0.988
eicosenoylcarnitine (C20:1)*	-0.051	0.050	0.302	0.525	-0.030	0.052	0.564	0.755	0.079	0.110	0.475	0.902
epiandrosterone sulfate	-0.106	0.067	0.114	0.296	-0.154	0.069	0.028	0.117	-0.257	0.148	0.086	0.819
equol sulfate	0.412	0.187	0.033	0.131	0.313	0.213	0.149	0.358	-0.359	0.427	0.406	0.893
erucate (22:1n9)	-0.006	0.042	0.888	0.940	-0.023	0.044	0.605	0.770	-0.078	0.093	0.402	0.893
erucoylcarnitine (C22:1)*	0.043	0.074	0.566	0.742	0.008	0.079	0.917	0.959	-0.192	0.190	0.315	0.875
erythronate*	0.196	0.088	0.028	0.118	0.194	0.092	0.037	0.143	0.059	0.197	0.765	0.982
ethylmalonate	0.056	0.088	0.530	0.713	0.057	0.092	0.537	0.742	0.026	0.198	0.895	0.992
etiocholanolone glucuronide	0.071	0.087	0.412	0.618	-0.002	0.089	0.982	0.989	-0.286	0.183	0.121	0.819
eugenol sulfate	-0.059	0.092	0.523	0.710	-0.134	0.100	0.180	0.400	-0.418	0.232	0.074	0.819
FAD	0.121	0.105	0.253	0.472	0.138	0.111	0.218	0.443	0.112	0.235	0.637	0.977
ferulic acid 4-sulfate	0.140	0.092	0.130	0.322	0.115	0.094	0.220	0.445	-0.048	0.201	0.811	0.988
fructose	0.202	0.077	0.010	0.054	0.170	0.081	0.037	0.142	-0.071	0.172	0.680	0.980
fumarate	-0.141	0.088	0.110	0.289	-0.128	0.092	0.167	0.381	0.015	0.200	0.941	0.993
galactonate	0.017	0.092	0.851	0.924	0.021	0.095	0.828	0.923	0.023	0.203	0.911	0.992
gamma-carboxyglutamate	0.160	0.088	0.071	0.212	0.204	0.091	0.026	0.113	0.261	0.195	0.182	0.839
gamma-CEHC	0.042	0.068	0.539	0.718	-0.021	0.070	0.768	0.887	-0.269	0.149	0.073	0.819
gamma-CEHC glucuronide*	0.159	0.098	0.108	0.287	0.135	0.101	0.183	0.402	-0.058	0.230	0.801	0.988
gamma-glutamyl-2-aminobutyrate	0.075	0.094	0.426	0.630	0.015	0.097	0.875	0.944	-0.252	0.210	0.233	0.844
gamma-glutamylalanine	0.078	0.086	0.365	0.582	0.024	0.089	0.788	0.900	-0.210	0.188	0.264	0.848
gamma-glutamylcitrulline*	-0.112	0.078	0.155	0.352	-0.077	0.082	0.345	0.585	0.116	0.174	0.505	0.926
gamma-glutamyl-epsilon-lysine	0.181	0.074	0.015	0.076	0.205	0.077	0.009	0.049	0.172	0.165	0.299	0.873
gamma-glutamylglutamine	-0.197	0.084	0.020	0.092	-0.155	0.088	0.079	0.235	0.121	0.186	0.518	0.941

gamma-glutamylhistidine	-0.087	0.084	0.301	0.524	-0.084	0.088	0.341	0.581	-0.015	0.188	0.935	0.993
gamma-glutamylmethionine	0.059	0.082	0.466	0.666	0.072	0.085	0.398	0.629	0.078	0.182	0.669	0.980
gamma-glutamylthreonine	0.059	0.080	0.461	0.660	0.096	0.083	0.250	0.479	0.191	0.179	0.288	0.873
gamma-glutamyltryptophan	0.202	0.084	0.018	0.087	0.194	0.087	0.027	0.114	0.051	0.186	0.784	0.988
genistein sulfate*	0.190	0.120	0.118	0.302	0.109	0.128	0.396	0.628	-0.271	0.258	0.297	0.873
gentisate	0.022	0.087	0.804	0.897	0.078	0.089	0.383	0.620	0.266	0.190	0.163	0.819
gluconate	0.066	0.089	0.456	0.656	0.080	0.093	0.388	0.622	0.087	0.198	0.662	0.980
glucuronate	0.036	0.076	0.632	0.787	-0.050	0.079	0.526	0.735	-0.386	0.167	0.022	0.738
glutamine	-0.138	0.093	0.142	0.337	-0.117	0.097	0.232	0.462	0.045	0.208	0.830	0.988
glutarate (C5-DC)	-0.188	0.093	0.044	0.156	-0.220	0.098	0.025	0.110	-0.203	0.210	0.335	0.883
glutaroylcarnitine (C5)	0.118	0.080	0.138	0.334	0.140	0.083	0.093	0.266	0.141	0.178	0.430	0.898
glycerate	-0.076	0.079	0.340	0.557	-0.121	0.082	0.144	0.349	-0.232	0.176	0.190	0.839
glycerol	0.103	0.082	0.208	0.420	0.100	0.085	0.243	0.474	0.022	0.183	0.906	0.992
glycerol 3-phosphate	0.119	0.078	0.127	0.318	0.121	0.081	0.136	0.336	0.053	0.173	0.759	0.982
glycerophosphoethanolamine	-0.015	0.085	0.858	0.927	0.024	0.089	0.786	0.900	0.175	0.190	0.359	0.887
glycerophosphoglycerol	0.181	0.095	0.058	0.192	0.150	0.099	0.132	0.329	-0.066	0.206	0.748	0.982
glycerophosphorylcholine (GPC)	-0.163	0.092	0.079	0.232	-0.113	0.096	0.244	0.475	0.170	0.205	0.408	0.893
glycine conjugate of C10H14O2 (1)*	0.122	0.082	0.141	0.337	0.082	0.086	0.340	0.580	-0.139	0.184	0.450	0.902
glyco-alpha-muricholate	-0.071	0.099	0.474	0.673	-0.083	0.103	0.422	0.644	-0.082	0.226	0.717	0.980
glyco-beta-muricholate	0.007	0.094	0.939	0.971	0.002	0.100	0.984	0.989	-0.022	0.216	0.919	0.992
glycochenodeoxycholate	0.087	0.076	0.250	0.471	0.083	0.079	0.292	0.529	0.014	0.169	0.934	0.993
glycochenodeoxycholate 3-sulfate	0.113	0.074	0.128	0.320	0.112	0.077	0.147	0.355	0.037	0.165	0.821	0.988
glycochenodeoxycholate glucuronide (1)	0.107	0.076	0.161	0.357	0.123	0.079	0.121	0.309	0.114	0.169	0.503	0.922
glycocholate	0.094	0.079	0.234	0.450	0.085	0.082	0.302	0.542	-0.007	0.176	0.969	0.995
glycocholenate sulfate*	-0.017	0.084	0.841	0.920	0.052	0.087	0.549	0.747	0.309	0.185	0.097	0.819
glycodeoxycholate	0.092	0.095	0.336	0.554	0.091	0.102	0.373	0.614	0.018	0.221	0.937	0.993
glycodeoxycholate 3-sulfate	0.070	0.093	0.456	0.656	0.085	0.099	0.394	0.627	0.086	0.210	0.682	0.980
glycohyocholate	-0.040	0.086	0.640	0.792	-0.069	0.091	0.445	0.664	-0.143	0.192	0.458	0.902
glycolithocholate	-0.086	0.099	0.385	0.598	-0.066	0.106	0.534	0.742	0.069	0.226	0.761	0.982
glycolithocholate sulfate*	0.077	0.084	0.359	0.574	0.110	0.088	0.212	0.435	0.174	0.186	0.352	0.885
glycosyl ceramide (d18:1/20:0, d16:1/22:0)*	-0.062	0.073	0.391	0.602	-0.091	0.076	0.231	0.462	-0.152	0.162	0.349	0.885
glycosyl ceramide (d18:1/23:1, d17:1/24:1)*	-0.150	0.062	0.017	0.084	-0.171	0.066	0.010	0.056	-0.144	0.144	0.321	0.875
glycosyl ceramide (d18:2/24:1, d18:1/24:2)*	-0.157	0.063	0.014	0.070	-0.208	0.065	0.002	0.013	-0.292	0.139	0.037	0.761

glycosyl-N-behenoyl-sphingadienine (d18:2/22:0)*	-0.129	0.082	0.115	0.298	-0.165	0.086	0.055	0.190	-0.203	0.182	0.264	0.848
glycosyl-N-behenoyl-sphingosine (d18:1/22:0)*	-0.116	0.078	0.138	0.335	-0.137	0.081	0.093	0.266	-0.138	0.174	0.431	0.898
glycosyl-N-palmitoyl-sphingosine (d18:1/16:0)	-0.120	0.080	0.135	0.330	-0.071	0.084	0.395	0.627	0.179	0.178	0.316	0.875
glycosyl-N-stearoyl-sphingosine (d18:1/18:0)	-0.103	0.072	0.155	0.352	-0.086	0.076	0.254	0.483	0.039	0.161	0.810	0.988
glycosyl-N-tricosanoyl-sphingadienine (d18:2/23:0)*	-0.139	0.065	0.035	0.134	-0.199	0.067	0.004	0.025	-0.323	0.143	0.025	0.738
glycoursodeoxycholate	-0.045	0.080	0.571	0.745	-0.032	0.083	0.699	0.838	0.044	0.178	0.807	0.988
glycoursodeoxycholate glucuronide (2)	-0.059	0.175	0.737	0.856	-0.132	0.184	0.479	0.695	-0.365	0.407	0.378	0.888
glycyrrhetinate	-0.124	0.114	0.281	0.503	-0.201	0.119	0.095	0.269	-0.359	0.241	0.141	0.819
guaiacol sulfate	0.054	0.072	0.449	0.651	0.150	0.074	0.044	0.164	0.455	0.156	0.004	0.738
guanidinoacetate	-0.188	0.076	0.014	0.071	-0.208	0.079	0.009	0.052	-0.157	0.167	0.349	0.885
guanidinosuccinate	0.145	0.138	0.295	0.518	0.113	0.142	0.426	0.647	-0.087	0.307	0.778	0.985
guanosine	-0.041	0.100	0.684	0.815	-0.004	0.107	0.971	0.982	0.128	0.209	0.540	0.947
heme	0.100	0.082	0.224	0.440	0.084	0.084	0.323	0.566	-0.043	0.190	0.822	0.988
heneicosapentaenoate (21:5n3)	-0.076	0.096	0.431	0.635	-0.101	0.103	0.331	0.573	-0.142	0.236	0.549	0.947
heptadecenamide (17:1)*	0.128	0.107	0.233	0.450	0.152	0.108	0.164	0.380	0.197	0.267	0.462	0.902
heptenedioate (C7:1-DC)*	0.049	0.096	0.613	0.775	-0.001	0.098	0.995	0.995	-0.186	0.201	0.357	0.887
hexadecadienoate (16:2n6)	-0.050	0.075	0.501	0.693	-0.044	0.078	0.574	0.759	0.012	0.166	0.944	0.993
hexadecanedioate (C16)	0.033	0.078	0.674	0.809	0.008	0.082	0.924	0.961	-0.103	0.175	0.555	0.947
hexadecenedioate (C16:1-DC)*	0.001	0.069	0.985	0.990	-0.045	0.072	0.528	0.735	-0.212	0.153	0.167	0.825
hexanoylcarnitine (C6)	0.058	0.080	0.472	0.671	0.062	0.084	0.461	0.681	0.039	0.179	0.830	0.988
hexanoylglutamine	0.089	0.080	0.269	0.487	0.089	0.083	0.285	0.521	0.036	0.174	0.838	0.988
hexanoylglycine (C6)	-0.077	0.088	0.381	0.595	-0.134	0.089	0.131	0.328	-0.291	0.187	0.121	0.819
hippurate	-0.068	0.081	0.404	0.612	0.061	0.085	0.471	0.686	0.564	0.176	0.002	0.614
histidine	-0.080	0.089	0.370	0.587	-0.033	0.093	0.721	0.852	0.184	0.198	0.354	0.885
histidine betaine (hercynine)*	0.200	0.078	0.011	0.060	0.130	0.082	0.116	0.303	-0.248	0.173	0.153	0.819
homostachydrine*	-0.106	0.082	0.197	0.406	-0.146	0.086	0.092	0.266	-0.210	0.182	0.251	0.848
homovanillate (HVA)	0.063	0.099	0.529	0.713	0.070	0.101	0.490	0.705	0.063	0.224	0.778	0.985
HWESASXX*	0.201	0.107	0.063	0.200	0.229	0.105	0.032	0.130	0.253	0.237	0.288	0.873
hydroquinone sulfate	0.112	0.080	0.161	0.357	0.149	0.083	0.075	0.226	0.210	0.180	0.245	0.847
hydroxy-CMPF*	-0.024	0.046	0.607	0.773	-0.013	0.048	0.784	0.899	0.039	0.102	0.701	0.980
hydroxycotinine	-0.275	0.241	0.262	0.483	-0.282	0.232	0.233	0.462	-0.243	0.592	0.685	0.980
hydroxy-N6,N6,N6-trimethyllysine*	0.178	0.081	0.030	0.123	0.224	0.084	0.008	0.049	0.275	0.180	0.129	0.819
hydroxypalmitoyl sphingomyelin (d18:1/16:0(OH))	-0.147	0.072	0.044	0.156	-0.143	0.075	0.060	0.200	-0.035	0.161	0.830	0.988

hydroxyproline	0.192	0.074	0.011	0.059	0.143	0.078	0.069	0.212	-0.152	0.166	0.361	0.887
hyocholate	-0.214	0.095	0.026	0.112	-0.238	0.101	0.020	0.092	-0.170	0.223	0.448	0.902
hypotaurine	-0.167	0.081	0.041	0.149	-0.143	0.085	0.095	0.269	0.047	0.179	0.791	0.988
hypoxanthine	-0.044	0.068	0.520	0.708	-0.068	0.071	0.338	0.578	-0.126	0.152	0.407	0.893
imidazole lactate	-0.110	0.076	0.145	0.341	-0.109	0.079	0.167	0.381	-0.034	0.168	0.839	0.988
imidazole propionate	0.122	0.092	0.185	0.392	0.114	0.096	0.234	0.463	0.002	0.214	0.991	0.998
iminodiacetate (IDA)	0.037	0.071	0.603	0.770	0.013	0.074	0.862	0.941	-0.097	0.159	0.543	0.947
indole-3-carboxylate	0.137	0.103	0.186	0.392	0.224	0.110	0.044	0.164	0.409	0.229	0.077	0.819
indoleacetate	0.074	0.076	0.335	0.554	0.064	0.079	0.421	0.644	-0.017	0.170	0.921	0.992
indoleacetylcarnitine*	0.049	0.090	0.586	0.755	0.091	0.094	0.334	0.574	0.202	0.198	0.309	0.873
indoleacetylglutamine	0.094	0.107	0.380	0.594	0.095	0.110	0.388	0.622	0.044	0.233	0.852	0.988
indolelactate	0.008	0.071	0.916	0.953	0.038	0.074	0.613	0.776	0.140	0.158	0.379	0.888
indolepropionate	-0.069	0.081	0.399	0.608	-0.008	0.085	0.926	0.962	0.256	0.181	0.160	0.819
indolin-2-one	-0.041	0.120	0.733	0.855	-0.111	0.127	0.385	0.620	-0.276	0.247	0.265	0.848
inosine	-0.086	0.070	0.224	0.440	-0.103	0.073	0.158	0.371	-0.112	0.156	0.473	0.902
isobutyrylcarnitine (C4)	-0.024	0.085	0.774	0.881	-0.017	0.088	0.851	0.936	0.027	0.189	0.888	0.992
isobutyrylglycine (C4)	0.003	0.112	0.982	0.990	-0.006	0.116	0.960	0.975	-0.040	0.257	0.877	0.992
isoeugenol sulfate	0.103	0.135	0.447	0.649	0.045	0.131	0.733	0.864	-0.286	0.324	0.382	0.888
isoursodeoxycholate	-0.080	0.080	0.315	0.538	-0.071	0.083	0.393	0.627	0.013	0.178	0.944	0.993
isovalerylglycine	0.104	0.103	0.314	0.537	0.036	0.107	0.738	0.868	-0.313	0.241	0.197	0.841
I-urobilinogen	0.057	0.122	0.642	0.792	0.022	0.128	0.864	0.941	-0.144	0.277	0.606	0.963
kynurenate	0.180	0.086	0.038	0.140	0.173	0.089	0.055	0.190	0.035	0.191	0.856	0.988
kynurenine	0.005	0.079	0.947	0.974	0.084	0.082	0.305	0.544	0.364	0.174	0.038	0.761
lactate	0.036	0.077	0.638	0.790	0.038	0.080	0.638	0.792	0.020	0.172	0.906	0.992
lactosyl-N-behenoyl-sphingosine (d18:1/22:0)*	-0.148	0.069	0.033	0.131	-0.132	0.072	0.068	0.210	0.017	0.153	0.910	0.992
lanthionine	0.160	0.084	0.058	0.192	0.163	0.087	0.064	0.205	0.071	0.186	0.704	0.980
laurate (12:0)	-0.021	0.083	0.801	0.896	-0.002	0.087	0.982	0.989	0.079	0.185	0.669	0.980
laurylcarnitine (C12)	-0.044	0.087	0.611	0.775	-0.008	0.091	0.928	0.962	0.149	0.194	0.445	0.902
leukotriene B4	0.022	0.116	0.849	0.922	0.075	0.117	0.523	0.732	0.237	0.243	0.330	0.880
lignoceroyl sphingomyelin (d18:1/24:0)	0.060	0.054	0.265	0.485	0.071	0.056	0.206	0.428	0.072	0.120	0.552	0.947
lignoceroylcarnitine (C24)*	0.132	0.073	0.071	0.212	0.158	0.076	0.039	0.147	0.162	0.162	0.317	0.875
linoleate (18:2n6)	-0.022	0.079	0.778	0.881	-0.001	0.083	0.994	0.995	0.091	0.177	0.606	0.963
linolenate (18:3n3 or 3n6)	-0.031	0.072	0.664	0.806	-0.025	0.075	0.742	0.870	0.019	0.161	0.909	0.992

linolenoylcarnitine (C18:3)*	-0.014	0.069	0.842	0.920	-0.010	0.071	0.889	0.949	0.013	0.156	0.934	0.993
linoleoyl ethanolamide	-0.032	0.070	0.647	0.794	-0.075	0.073	0.303	0.543	-0.203	0.153	0.188	0.839
linoleoylcarnitine (C18:2)*	-0.043	0.069	0.535	0.716	0.005	0.072	0.945	0.969	0.202	0.153	0.187	0.839
linoleoylcholine*	-0.114	0.080	0.155	0.352	-0.142	0.082	0.087	0.253	-0.172	0.177	0.331	0.880
lithocholate sulfate (1)	-0.160	0.106	0.134	0.329	-0.142	0.115	0.221	0.445	0.034	0.232	0.883	0.992
L-urobilin	-0.017	0.132	0.899	0.947	0.059	0.138	0.672	0.823	0.384	0.312	0.222	0.844
malate	0.012	0.077	0.874	0.931	0.075	0.080	0.349	0.587	0.291	0.170	0.089	0.819
maleate	0.051	0.081	0.526	0.712	0.022	0.084	0.793	0.903	-0.115	0.180	0.526	0.942
margarate (17:0)	-0.071	0.083	0.396	0.605	-0.050	0.087	0.564	0.755	0.069	0.186	0.711	0.980
margaroylcarnitine (C17)*	0.058	0.082	0.482	0.680	0.119	0.085	0.166	0.381	0.299	0.182	0.103	0.819
mead acid (20:3n9)	-0.038	0.103	0.710	0.840	-0.034	0.113	0.768	0.887	0.029	0.276	0.917	0.992
methionine	0.098	0.084	0.243	0.462	0.103	0.087	0.238	0.469	0.059	0.186	0.753	0.982
methionine sulfone	-0.036	0.082	0.661	0.805	-0.010	0.086	0.910	0.957	0.107	0.183	0.559	0.947
methionine sulfoxide	0.166	0.078	0.035	0.135	0.155	0.082	0.060	0.200	0.008	0.174	0.963	0.994
methyl glucopyranoside (alpha + beta)	-0.167	0.080	0.037	0.139	-0.217	0.082	0.009	0.051	-0.296	0.179	0.100	0.819
methyl indole-3-acetate	0.152	0.101	0.135	0.331	0.211	0.106	0.049	0.174	0.331	0.235	0.163	0.819
methyl-4-hydroxybenzoate sulfate	-0.077	0.076	0.311	0.533	-0.071	0.078	0.367	0.604	-0.002	0.168	0.989	0.998
methylnaphthyl sulfate (2)*	-0.075	0.070	0.284	0.507	-0.113	0.072	0.117	0.303	-0.215	0.159	0.179	0.839
methylsuccinate	-0.021	0.086	0.810	0.901	0.014	0.089	0.879	0.944	0.149	0.191	0.435	0.900
myo-inositol	0.068	0.082	0.406	0.614	0.046	0.085	0.593	0.765	-0.078	0.182	0.669	0.980
myristate (14:0)	0.022	0.079	0.780	0.881	0.069	0.083	0.403	0.633	0.222	0.176	0.210	0.841
myristoleate (14:1n5)	-0.129	0.075	0.087	0.249	-0.102	0.078	0.194	0.417	0.075	0.167	0.653	0.980
myristoleoylcarnitine (C14:1)*	-0.077	0.082	0.344	0.559	-0.042	0.085	0.620	0.779	0.132	0.182	0.467	0.902
myristoylcarnitine (C14)	0.046	0.087	0.599	0.766	0.079	0.091	0.382	0.620	0.170	0.194	0.383	0.888
N-(2-furoyl)glycine	0.009	0.112	0.938	0.970	-0.050	0.113	0.660	0.811	-0.250	0.236	0.292	0.873
N,N,N-trimethyl-5-aminovalerate	0.094	0.072	0.189	0.395	0.075	0.075	0.314	0.554	-0.052	0.159	0.743	0.982
N,N,N-trimethyl-alanylproline betaine (TMAP)	0.161	0.084	0.057	0.191	0.150	0.088	0.088	0.256	0.009	0.187	0.963	0.994
N1-Methyl-2-pyridone-5-carboxamide	0.149	0.076	0.051	0.175	0.187	0.079	0.018	0.087	0.230	0.168	0.173	0.833
N1-methylinosine	0.165	0.086	0.057	0.192	0.234	0.089	0.009	0.052	0.376	0.190	0.050	0.819
N2,N2-dimethylguanosine	0.222	0.093	0.018	0.086	0.274	0.096	0.005	0.031	0.317	0.205	0.124	0.819
N2,N5-diacetylornithine	0.010	0.069	0.890	0.941	0.013	0.072	0.861	0.941	0.017	0.153	0.910	0.992
N2-acetyl,N6-methyllysine	-0.118	0.102	0.252	0.471	-0.133	0.106	0.216	0.440	-0.117	0.248	0.639	0.978
N4-acetylcytidine	0.200	0.080	0.014	0.070	0.199	0.084	0.019	0.088	0.068	0.179	0.705	0.980

N6,N6-dimethyllysine	0.121	0.083	0.150	0.346	0.164	0.086	0.060	0.200	0.247	0.188	0.190	0.839
N6-acetyllysine	0.193	0.081	0.018	0.088	0.229	0.084	0.007	0.043	0.236	0.180	0.193	0.839
N6-carbamoylthreonyladenosine	0.205	0.093	0.028	0.118	0.220	0.096	0.024	0.105	0.143	0.208	0.493	0.912
N6-methyllysine	-0.088	0.082	0.287	0.510	-0.052	0.086	0.546	0.744	0.132	0.183	0.473	0.902
N-acetyl-1-methylhistidine*	0.059	0.136	0.667	0.806	0.100	0.143	0.489	0.704	0.234	0.334	0.486	0.905
N-acetyl-2-aminooctanoate*	0.097	0.081	0.233	0.450	0.064	0.084	0.452	0.672	-0.116	0.180	0.520	0.941
N-acetylalliin	0.201	0.094	0.034	0.133	0.226	0.096	0.020	0.094	0.198	0.206	0.338	0.883
N-acetylarginine	0.024	0.086	0.782	0.883	0.078	0.089	0.386	0.622	0.254	0.191	0.184	0.839
N-acetylaspartate (NAA)	-0.144	0.090	0.109	0.288	-0.132	0.093	0.157	0.371	-0.002	0.195	0.993	0.999
N-acetyl-aspartyl-glutamate (NAAG)	0.049	0.099	0.618	0.778	-0.008	0.103	0.938	0.966	-0.243	0.219	0.270	0.855
N-acetyl-beta-alanine	0.076	0.080	0.341	0.557	0.107	0.083	0.197	0.420	0.168	0.176	0.341	0.885
N-acetylcitrulline	0.101	0.128	0.435	0.636	0.087	0.134	0.519	0.730	-0.029	0.289	0.920	0.992
N-acetylglucosamine/N-acetylgalactosamine	0.228	0.094	0.016	0.081	0.250	0.098	0.011	0.061	0.183	0.209	0.382	0.888
N-acetylglucosaminylasparagine	0.333	0.125	0.009	0.053	0.270	0.132	0.044	0.164	-0.170	0.280	0.547	0.947
N-acetylglutamine	0.101	0.084	0.232	0.450	0.095	0.088	0.281	0.516	0.010	0.188	0.959	0.994
N-acetylhistidine	0.014	0.097	0.886	0.938	0.041	0.105	0.702	0.840	0.122	0.225	0.587	0.959
N-acetyl-isoputreanine*	0.019	0.089	0.828	0.914	0.052	0.093	0.573	0.758	0.158	0.198	0.428	0.897
N-acetylmethionine	-0.066	0.074	0.376	0.591	-0.075	0.077	0.332	0.573	-0.067	0.166	0.689	0.980
N-acetylneuraminate	0.041	0.097	0.673	0.809	0.052	0.101	0.605	0.770	0.066	0.216	0.760	0.982
N-acetylproline	0.131	0.097	0.178	0.383	0.194	0.100	0.055	0.190	0.340	0.217	0.120	0.819
N-acetylserine	0.111	0.090	0.218	0.433	0.102	0.093	0.276	0.510	0.001	0.200	0.996	0.999
N-acetyltaurine	-0.060	0.073	0.418	0.623	-0.072	0.076	0.347	0.586	-0.080	0.165	0.631	0.974
N-acetylthreonine	0.215	0.087	0.015	0.074	0.207	0.091	0.024	0.106	0.043	0.195	0.824	0.988
N-behenoyl-sphingadienine (d18:2/22:0)*	0.101	0.067	0.134	0.329	0.090	0.070	0.201	0.426	-0.015	0.150	0.921	0.992
N-carbamoylvaline	0.156	0.085	0.068	0.212	0.106	0.089	0.232	0.462	-0.165	0.187	0.381	0.888
N-delta-acetylornithine	0.070	0.080	0.383	0.597	0.094	0.083	0.261	0.491	0.135	0.179	0.451	0.902
nervonoylcarnitine (C24:1)*	0.065	0.050	0.194	0.403	0.061	0.053	0.250	0.480	-0.004	0.119	0.974	0.995
N-formylanthranilic acid	0.023	0.084	0.789	0.886	0.016	0.087	0.851	0.936	-0.020	0.187	0.917	0.992
N-formylmethionine	0.065	0.087	0.458	0.658	0.049	0.091	0.590	0.765	-0.049	0.195	0.804	0.988
N-formylphenylalanine	0.135	0.101	0.183	0.389	0.098	0.106	0.358	0.596	-0.136	0.232	0.560	0.947
nicotinamide	0.023	0.074	0.754	0.870	-0.012	0.077	0.874	0.944	-0.154	0.165	0.354	0.885
nisinate (24:6n3)	0.172	0.085	0.044	0.156	0.139	0.087	0.112	0.295	-0.086	0.191	0.655	0.980
N-linoleoylglycine	-0.055	0.108	0.612	0.775	-0.101	0.107	0.349	0.587	-0.229	0.223	0.308	0.873

N-linoleoylserine*	0.022	0.085	0.795	0.891	0.026	0.091	0.777	0.893	0.022	0.195	0.910	0.992
N-linoleoyltaurine*	0.074	0.104	0.479	0.675	0.079	0.102	0.441	0.662	0.066	0.211	0.757	0.982
N-methylhydroxyproline	-0.020	0.098	0.841	0.920	-0.087	0.103	0.400	0.630	-0.355	0.233	0.132	0.819
N-methylpipecolate	-0.042	0.084	0.616	0.776	0.011	0.088	0.902	0.955	0.227	0.187	0.227	0.844
N-methylproline	0.014	0.081	0.864	0.930	-0.071	0.084	0.400	0.630	-0.407	0.184	0.028	0.738
N-oleoylserine	-0.115	0.080	0.149	0.345	-0.129	0.083	0.122	0.311	-0.103	0.177	0.561	0.947
N-oleoyltaurine	0.089	0.075	0.237	0.454	0.103	0.078	0.189	0.411	0.097	0.169	0.569	0.947
nonadecanoate (19:0)	0.033	0.078	0.671	0.809	0.009	0.082	0.913	0.959	-0.099	0.175	0.571	0.947
nonanoylcarnitine (C9)	0.018	0.101	0.858	0.927	0.019	0.102	0.853	0.936	0.013	0.225	0.955	0.994
N-palmitoylglycine	0.013	0.081	0.874	0.931	0.013	0.085	0.880	0.944	0.004	0.181	0.983	0.997
N-palmitoyl-heptadecasphingosine (d17:1/16:0)*	0.130	0.094	0.169	0.371	0.183	0.096	0.060	0.200	0.299	0.209	0.155	0.819
N-palmitoylserine	-0.133	0.130	0.308	0.530	-0.076	0.145	0.602	0.769	0.212	0.291	0.468	0.902
N-palmitoyl-sphingadienine (d18:2/16:0)*	0.083	0.078	0.289	0.512	0.105	0.081	0.198	0.420	0.130	0.174	0.456	0.902
N-stearoylserine*	-0.041	0.110	0.710	0.840	-0.023	0.115	0.844	0.934	0.070	0.246	0.778	0.985
N-stearoyltaurine	-0.092	0.095	0.336	0.554	-0.080	0.103	0.443	0.663	0.053	0.237	0.823	0.988
O-acetylhomoserine	0.085	0.098	0.388	0.598	0.052	0.105	0.618	0.778	-0.126	0.224	0.573	0.947
o-cresol sulfate	0.114	0.071	0.112	0.293	0.100	0.075	0.179	0.398	-0.020	0.160	0.899	0.992
octadecadienedioate (C18:2-DC)*	0.082	0.083	0.322	0.542	0.010	0.086	0.909	0.957	-0.300	0.183	0.104	0.819
octadecanedioate (C18)	0.071	0.081	0.382	0.596	0.018	0.086	0.834	0.927	-0.227	0.185	0.221	0.844
octadecanedioylcarnitine (C18-DC)*	0.025	0.075	0.734	0.855	0.056	0.078	0.472	0.686	0.149	0.166	0.371	0.888
octadecenedioate (C18:1-DC)*	0.074	0.071	0.296	0.519	0.015	0.074	0.840	0.931	-0.243	0.157	0.123	0.819
octadecenedioylcarnitine (C18:1-DC)*	0.004	0.070	0.949	0.976	-0.018	0.073	0.808	0.911	-0.100	0.157	0.524	0.942
octanoylcarnitine (C8)	-0.024	0.085	0.778	0.881	-0.012	0.089	0.893	0.949	0.046	0.190	0.809	0.988
oleate/vaccenate (18:1)	-0.002	0.080	0.982	0.990	0.010	0.083	0.904	0.955	0.054	0.178	0.764	0.982
oleoyl ethanolamide	-0.041	0.074	0.577	0.749	-0.064	0.077	0.403	0.632	-0.121	0.164	0.463	0.902
oleoylcarnitine (C18)	-0.013	0.070	0.852	0.924	0.040	0.073	0.589	0.765	0.236	0.156	0.132	0.819
oleoylcholine	-0.155	0.079	0.051	0.176	-0.171	0.082	0.039	0.147	-0.131	0.176	0.460	0.902
ornithine	0.168	0.087	0.055	0.186	0.174	0.090	0.055	0.190	0.091	0.194	0.638	0.977
orotate	0.020	0.099	0.840	0.920	0.016	0.103	0.875	0.944	-0.010	0.220	0.965	0.994
orotidine	0.191	0.102	0.062	0.199	0.178	0.106	0.097	0.273	0.009	0.226	0.970	0.995
O-sulfo-L-tyrosine	0.115	0.088	0.192	0.400	0.142	0.091	0.121	0.309	0.166	0.195	0.394	0.893
oxalate (ethanedioate)	-0.050	0.084	0.548	0.726	-0.104	0.087	0.233	0.462	-0.262	0.185	0.158	0.819
palmitate (16:0)	0.043	0.083	0.609	0.775	0.070	0.087	0.424	0.644	0.138	0.186	0.458	0.902

palmitoleamide (16:1)*	0.150	0.082	0.068	0.212	0.187	0.085	0.029	0.120	0.223	0.183	0.226	0.844
palmitoleate (16:1n7)	-0.050	0.077	0.519	0.708	-0.015	0.080	0.848	0.935	0.139	0.171	0.420	0.893
palmitoleoylcarnitine (C16:1)*	-0.075	0.077	0.328	0.548	-0.040	0.080	0.616	0.777	0.133	0.171	0.440	0.900
palmitoyl dihydrosphingomyelin (d18:0/16:0)*	-0.034	0.077	0.662	0.805	-0.037	0.081	0.643	0.796	-0.028	0.173	0.869	0.992
palmitoyl sphingomyelin (d18:1/16:0)	-0.139	0.076	0.070	0.212	-0.120	0.080	0.134	0.331	0.036	0.171	0.832	0.988
palmitoylcarnitine (C16)	0.148	0.082	0.072	0.214	0.237	0.084	0.005	0.035	0.459	0.179	0.011	0.738
palmitoylcholine	-0.119	0.081	0.145	0.341	-0.132	0.085	0.120	0.308	-0.104	0.182	0.566	0.947
paraxanthine	-0.077	0.147	0.603	0.770	-0.111	0.161	0.493	0.706	-0.200	0.392	0.612	0.969
p-cresol glucuronide*	-0.082	0.098	0.403	0.612	-0.052	0.102	0.615	0.777	0.105	0.215	0.626	0.973
p-cresol sulfate	-0.101	0.083	0.228	0.445	-0.024	0.087	0.781	0.896	0.313	0.185	0.092	0.819
pentadecanoate (15:0)	0.001	0.079	0.994	0.997	0.046	0.082	0.579	0.762	0.205	0.175	0.243	0.847
perfluorooctanesulfonate (PFOS)	0.107	0.050	0.033	0.131	0.065	0.052	0.218	0.443	-0.155	0.110	0.162	0.819
perfluorooctanoate (PFOA)*	0.066	0.036	0.069	0.212	0.076	0.038	0.044	0.164	0.070	0.080	0.387	0.889
phenol glucuronide	-0.011	0.101	0.916	0.953	0.066	0.106	0.536	0.742	0.367	0.232	0.117	0.819
phenol sulfate	0.087	0.083	0.299	0.522	0.090	0.087	0.298	0.538	0.049	0.185	0.794	0.988
phenylacetate	-0.185	0.106	0.082	0.239	-0.154	0.106	0.151	0.361	0.052	0.229	0.822	0.988
phenylacetylcarnitine	-0.016	0.092	0.863	0.930	-0.028	0.094	0.770	0.888	-0.060	0.202	0.766	0.982
phenylacetylglutamate	0.094	0.145	0.520	0.708	0.065	0.151	0.669	0.820	-0.118	0.346	0.733	0.981
phenylacetylglutamine	-0.045	0.085	0.598	0.766	0.015	0.089	0.868	0.943	0.258	0.190	0.176	0.839
phenyllactate (PLA)	0.068	0.072	0.350	0.564	0.052	0.076	0.493	0.706	-0.048	0.162	0.766	0.982
phenylpyruvate	0.082	0.082	0.321	0.542	0.062	0.086	0.473	0.686	-0.062	0.184	0.737	0.981
phosphate	-0.072	0.087	0.408	0.616	-0.083	0.091	0.360	0.597	-0.077	0.194	0.695	0.980
phosphocholine	0.168	0.082	0.043	0.154	0.139	0.086	0.108	0.289	-0.070	0.184	0.703	0.980
phosphoethanolamine (PE)	0.122	0.083	0.145	0.341	0.060	0.087	0.493	0.706	-0.238	0.184	0.198	0.841
picolinate	-0.131	0.127	0.304	0.526	-0.093	0.132	0.484	0.700	0.101	0.264	0.705	0.980
pimelate (C7-DC)	-0.007	0.115	0.952	0.977	-0.039	0.116	0.736	0.866	-0.167	0.262	0.526	0.942
pimeloylcarnitine/3-methyladipoylcarnitine (C7-DC)	0.239	0.112	0.036	0.136	0.189	0.120	0.118	0.303	-0.190	0.269	0.482	0.902
pipecolate	0.105	0.087	0.231	0.448	0.190	0.090	0.037	0.142	0.425	0.192	0.028	0.738
piperine	0.268	0.114	0.021	0.094	0.196	0.119	0.102	0.281	-0.251	0.261	0.339	0.885
pregnanediol-3-glucuronide	-0.079	0.089	0.380	0.594	-0.136	0.092	0.140	0.344	-0.288	0.194	0.139	0.819
pregnanolone/allopregnanolone sulfate	0.102	0.114	0.371	0.587	0.112	0.126	0.377	0.617	0.055	0.256	0.832	0.988
pregnen-diol disulfate*	-0.028	0.066	0.671	0.809	-0.055	0.068	0.425	0.646	-0.132	0.146	0.369	0.888
pregnenediol sulfate (C21H34O5S)*	-0.163	0.066	0.015	0.075	-0.210	0.068	0.003	0.018	-0.273	0.146	0.064	0.819

pregnenetriol disulfate*	0.082	0.066	0.211	0.424	0.042	0.068	0.542	0.744	-0.154	0.146	0.291	0.873
pregnenetriol sulfate*	0.012	0.063	0.853	0.924	-0.040	0.066	0.542	0.744	-0.233	0.140	0.098	0.819
pregnenolone sulfate	-0.160	0.069	0.022	0.097	-0.187	0.073	0.011	0.061	-0.168	0.155	0.281	0.869
propionylglycine (C3)	-0.212	0.086	0.015	0.073	-0.180	0.090	0.046	0.168	0.063	0.190	0.741	0.981
propyl 4-hydroxybenzoate sulfate	-0.050	0.125	0.688	0.818	-0.100	0.129	0.443	0.663	-0.244	0.276	0.382	0.888
pseudouridine	0.148	0.089	0.099	0.273	0.165	0.093	0.078	0.234	0.130	0.199	0.515	0.939
pyridoxal	0.083	0.108	0.444	0.646	0.156	0.113	0.166	0.381	0.364	0.240	0.131	0.819
pyroglutamine*	-0.113	0.057	0.047	0.163	-0.068	0.060	0.256	0.486	0.168	0.126	0.185	0.839
pyruvate	0.010	0.078	0.896	0.945	0.050	0.081	0.536	0.742	0.187	0.174	0.283	0.869
quinate	-0.013	0.085	0.882	0.936	-0.022	0.089	0.806	0.910	-0.048	0.195	0.805	0.988
quinolinate	0.120	0.090	0.185	0.392	0.175	0.094	0.063	0.204	0.292	0.199	0.144	0.819
ribitol	0.075	0.080	0.350	0.564	0.068	0.084	0.417	0.643	-0.005	0.179	0.978	0.996
ribonate (ribonolactone)	0.051	0.088	0.567	0.742	0.077	0.092	0.400	0.630	0.140	0.196	0.476	0.902
ribulonate/xylulonate*	0.118	0.087	0.174	0.378	0.053	0.090	0.560	0.755	-0.248	0.189	0.192	0.839
S-(3-hydroxypropyl)mercapturic acid (HPMA)	0.078	0.112	0.489	0.683	0.089	0.119	0.457	0.676	0.084	0.303	0.784	0.988
S-1-pyrroline-5-carboxylate	0.042	0.090	0.638	0.790	0.007	0.093	0.939	0.966	-0.146	0.200	0.468	0.902
saccharin	0.022	0.136	0.873	0.931	0.036	0.154	0.816	0.915	0.077	0.372	0.837	0.988
salicylate	0.040	0.084	0.631	0.785	0.059	0.087	0.504	0.716	0.097	0.187	0.605	0.963
salicyluric glucuronide*	0.333	0.195	0.107	0.286	0.160	0.201	0.438	0.660	-0.735	0.419	0.100	0.819
S-allylcysteine	-0.031	0.088	0.727	0.852	0.026	0.091	0.777	0.893	0.258	0.198	0.195	0.839
sarcosine	0.117	0.080	0.148	0.344	0.042	0.084	0.622	0.780	-0.301	0.178	0.092	0.819
sebacate (C10-DC)	-0.024	0.076	0.751	0.868	-0.064	0.079	0.419	0.644	-0.191	0.169	0.260	0.848
S-methylcysteine	0.093	0.078	0.239	0.455	0.112	0.081	0.170	0.384	0.123	0.174	0.480	0.902
S-methylcysteine sulfoxide	0.056	0.078	0.472	0.671	0.012	0.081	0.883	0.944	-0.181	0.173	0.297	0.873
S-methylmethionine	0.111	0.077	0.152	0.348	0.101	0.081	0.214	0.438	-0.007	0.172	0.968	0.995
spermidine	0.133	0.083	0.113	0.293	0.198	0.089	0.028	0.117	0.343	0.197	0.084	0.819
sphinganine	0.164	0.080	0.042	0.151	0.158	0.084	0.061	0.200	0.030	0.179	0.867	0.992
sphinganine-1-phosphate	-0.068	0.088	0.437	0.638	-0.107	0.091	0.242	0.473	-0.201	0.195	0.303	0.873
sphingomyelin (d17:1/14:0, d16:1/15:0)*	0.111	0.071	0.120	0.303	0.113	0.074	0.127	0.322	0.051	0.158	0.749	0.982
sphingomyelin (d17:1/16:0, d18:1/15:0, d16:1/17:0)*	-0.008	0.072	0.908	0.953	0.004	0.075	0.957	0.973	0.053	0.160	0.739	0.981
sphingomyelin (d18:1/17:0, d17:1/18:0, d19:1/16:0)	-0.110	0.069	0.110	0.289	-0.105	0.071	0.145	0.350	-0.016	0.153	0.918	0.992
sphingomyelin (d18:1/19:0, d19:1/18:0)*	0.158	0.076	0.040	0.149	0.132	0.080	0.100	0.279	-0.059	0.170	0.728	0.980
sphingomyelin (d18:1/20:0, d16:1/22:0)*	0.160	0.075	0.035	0.134	0.145	0.079	0.067	0.210	-0.013	0.168	0.941	0.993

sphingomyelin (d18:1/20:1, d18:2/20:0)*	0.008	0.071	0.912	0.953	-0.072	0.073	0.325	0.566	-0.363	0.155	0.020	0.738
sphingomyelin (d18:1/20:2, d18:2/20:1, d16:1/22:2)*	0.058	0.071	0.412	0.618	0.065	0.074	0.381	0.620	0.052	0.162	0.749	0.982
sphingomyelin (d18:1/22:1, d18:2/22:0, d16:1/24:1)*	0.133	0.068	0.053	0.180	0.091	0.071	0.205	0.428	-0.144	0.152	0.344	0.885
sphingomyelin (d18:1/22:2, d18:2/22:1, d16:1/24:2)*	0.074	0.057	0.196	0.406	0.011	0.060	0.859	0.941	-0.261	0.125	0.038	0.761
sphingomyelin (d18:1/24:1, d18:2/24:0)*	0.041	0.050	0.413	0.618	0.029	0.052	0.587	0.765	-0.043	0.112	0.702	0.980
sphingomyelin (d18:1/25:0, d19:0/24:1, d20:1/23:0, d19:1/24:0)*	0.085	0.059	0.152	0.348	0.080	0.062	0.197	0.420	0.007	0.132	0.958	0.994
sphingomyelin (d18:2/16:0, d18:1/16:1)*	0.133	0.073	0.069	0.212	0.157	0.076	0.039	0.147	0.159	0.162	0.328	0.880
sphingomyelin (d18:2/18:1)*	0.002	0.073	0.977	0.990	-0.030	0.076	0.697	0.838	-0.145	0.163	0.375	0.888
sphingomyelin (d18:2/23:0, d18:1/23:1, d17:1/24:1)*	0.084	0.067	0.214	0.427	0.065	0.070	0.354	0.590	-0.055	0.150	0.716	0.980
sphingomyelin (d18:2/23:1)*	0.048	0.061	0.435	0.636	0.010	0.063	0.872	0.944	-0.153	0.135	0.259	0.848
sphingomyelin (d18:2/24:1, d18:1/24:2)*	-0.019	0.066	0.776	0.881	-0.070	0.068	0.305	0.544	-0.241	0.145	0.098	0.819
sphingomyelin (d18:2/24:2)*	-0.051	0.046	0.265	0.485	-0.065	0.048	0.174	0.388	-0.082	0.102	0.424	0.893
sphingosine	0.182	0.077	0.020	0.092	0.199	0.080	0.014	0.071	0.146	0.172	0.396	0.893
sphingosine 1-phosphate	-0.081	0.082	0.321	0.542	-0.058	0.085	0.500	0.712	0.078	0.182	0.667	0.980
stachydrine	0.035	0.081	0.668	0.807	-0.039	0.085	0.642	0.795	-0.326	0.179	0.071	0.819
stearamide (18:0)	0.000	0.085	0.998	0.999	0.006	0.088	0.946	0.969	0.027	0.189	0.889	0.992
stearate (18:0)	0.021	0.082	0.796	0.891	0.014	0.085	0.868	0.943	-0.024	0.182	0.894	0.992
stearidonate (18:4n3)	0.065	0.069	0.347	0.563	0.059	0.072	0.413	0.641	-0.004	0.153	0.982	0.997
stearoyl sphingomyelin (d18:1/18:0)	0.154	0.075	0.040	0.149	0.155	0.078	0.048	0.173	0.059	0.167	0.725	0.980
stearoylcarnitine (C18)	0.049	0.075	0.511	0.701	0.132	0.077	0.089	0.256	0.398	0.164	0.017	0.738
stearoylcholine*	-0.087	0.085	0.309	0.530	-0.118	0.088	0.181	0.400	-0.175	0.187	0.351	0.885
suberate (C8-DC)	0.006	0.087	0.944	0.973	-0.055	0.090	0.539	0.743	-0.275	0.190	0.151	0.819
suberoylcarnitine (C8-DC)	0.182	0.114	0.114	0.296	0.145	0.115	0.212	0.435	-0.082	0.249	0.744	0.982
succinate	-0.044	0.082	0.595	0.764	-0.019	0.086	0.826	0.922	0.098	0.184	0.595	0.961
succinimide	-0.062	0.085	0.471	0.671	-0.108	0.088	0.222	0.446	-0.229	0.186	0.218	0.844
succinylcarnitine (C4)	0.192	0.084	0.024	0.104	0.235	0.087	0.008	0.046	0.266	0.187	0.156	0.819
sucrose	-0.094	0.101	0.355	0.572	-0.117	0.109	0.286	0.521	-0.126	0.235	0.593	0.961
sulfate*	0.219	0.086	0.012	0.064	0.160	0.091	0.080	0.236	-0.190	0.192	0.325	0.877
syringol sulfate	0.312	0.202	0.138	0.334	0.300	0.225	0.197	0.420	-0.022	0.495	0.964	0.994
tartarate	-0.152	0.091	0.095	0.264	-0.167	0.094	0.079	0.235	-0.123	0.202	0.544	0.947
tartronate (hydroxymalonate)	-0.109	0.081	0.177	0.383	-0.154	0.084	0.067	0.209	-0.245	0.179	0.172	0.831
taurine	0.058	0.085	0.498	0.691	-0.014	0.089	0.874	0.944	-0.308	0.189	0.106	0.819
taurochenodeoxycholate	0.084	0.081	0.300	0.522	0.083	0.084	0.324	0.566	0.027	0.177	0.877	0.992

taurochenodeoxycholic acid 3-sulfate	0.140	0.098	0.158	0.355	0.097	0.102	0.342	0.581	-0.140	0.219	0.523	0.942
taurocholate	0.025	0.096	0.796	0.891	-0.019	0.100	0.851	0.936	-0.206	0.222	0.354	0.885
taurocholenate sulfate*	0.138	0.084	0.103	0.278	0.153	0.088	0.083	0.245	0.118	0.187	0.530	0.942
taurodeoxycholate	0.096	0.099	0.335	0.554	0.086	0.107	0.419	0.644	-0.015	0.220	0.945	0.993
taurodeoxycholic acid 3-sulfate	-0.049	0.160	0.759	0.873	-0.115	0.165	0.486	0.701	-0.292	0.335	0.387	0.889
taurolithocholate 3-sulfate	0.038	0.095	0.692	0.821	0.020	0.102	0.847	0.935	-0.073	0.218	0.738	0.981
tauroursodeoxycholate	-0.275	0.145	0.061	0.199	-0.374	0.149	0.015	0.074	-0.467	0.287	0.109	0.819
tetradecadienedioate (C14:2-DC)*	-0.119	0.076	0.120	0.303	-0.087	0.080	0.277	0.511	0.104	0.170	0.543	0.947
tetradecadienoate (14:2)*	-0.118	0.081	0.148	0.344	-0.108	0.085	0.204	0.428	0.003	0.181	0.986	0.998
tetradecanedioate (C14)	-0.038	0.074	0.606	0.772	-0.024	0.077	0.752	0.875	0.049	0.165	0.767	0.982
theanine	0.193	0.148	0.200	0.409	0.119	0.163	0.470	0.686	-0.408	0.383	0.294	0.873
theobromine	0.157	0.108	0.151	0.346	0.154	0.113	0.179	0.398	0.025	0.274	0.926	0.993
theophylline	0.213	0.121	0.084	0.241	0.255	0.126	0.046	0.168	0.304	0.303	0.319	0.875
thioproline	0.132	0.079	0.096	0.264	0.176	0.082	0.033	0.132	0.246	0.175	0.161	0.819
threonate	-0.040	0.082	0.628	0.783	-0.092	0.086	0.285	0.521	-0.251	0.183	0.172	0.831
threonine	-0.032	0.084	0.701	0.831	-0.032	0.088	0.713	0.847	-0.012	0.188	0.950	0.993
thymol sulfate	0.111	0.103	0.281	0.503	0.122	0.105	0.249	0.479	0.104	0.251	0.681	0.980
thyroxine	0.050	0.081	0.537	0.716	0.009	0.084	0.917	0.959	-0.170	0.180	0.346	0.885
tiglyl carnitine (C5)	0.149	0.082	0.071	0.214	0.181	0.085	0.034	0.135	0.205	0.182	0.261	0.848
trans-urocanate	-0.053	0.083	0.521	0.708	-0.044	0.089	0.625	0.782	0.035	0.195	0.858	0.988
tricosanoyl sphingomyelin (d18:1/23:0)*	0.130	0.055	0.019	0.090	0.126	0.057	0.029	0.119	0.031	0.122	0.802	0.988
tridecenedioate (C13:1-DC)*	-0.043	0.075	0.567	0.742	-0.042	0.078	0.591	0.765	-0.011	0.166	0.947	0.993
trigonelline (N'-methylnicotinate)	0.003	0.083	0.968	0.988	0.036	0.086	0.675	0.827	0.151	0.184	0.414	0.893
trimethylamine N-oxide	0.064	0.074	0.389	0.600	0.117	0.076	0.129	0.324	0.265	0.163	0.106	0.819
tryptophan	0.028	0.103	0.783	0.883	0.062	0.107	0.565	0.755	0.163	0.229	0.479	0.902
tryptophan betaine	0.015	0.075	0.843	0.920	-0.074	0.078	0.346	0.585	-0.398	0.164	0.016	0.738
tyramine O-sulfate	0.171	0.086	0.048	0.167	0.149	0.091	0.102	0.281	-0.047	0.194	0.811	0.988
umbelliferone sulfate	0.329	0.138	0.021	0.095	0.288	0.155	0.069	0.212	-0.238	0.368	0.521	0.942
undecanedioate (C11-DC)	0.024	0.072	0.736	0.855	-0.030	0.075	0.686	0.832	-0.240	0.160	0.134	0.819
uracil	0.160	0.087	0.069	0.212	0.171	0.092	0.065	0.207	0.101	0.193	0.603	0.963
urea	0.193	0.088	0.030	0.124	0.172	0.092	0.063	0.205	-0.023	0.197	0.906	0.992
uridine	0.023	0.082	0.779	0.881	0.043	0.085	0.619	0.778	0.098	0.183	0.594	0.961
ursodeoxycholate	-0.150	0.082	0.069	0.212	-0.130	0.085	0.130	0.325	0.038	0.183	0.836	0.988

valerate (5:0)	-0.096	0.076	0.206	0.417	-0.124	0.079	0.117	0.303	-0.162	0.169	0.338	0.883
valylleucine	-0.319	0.148	0.034	0.133	-0.223	0.162	0.173	0.388	0.248	0.303	0.415	0.893
vanillactate	-0.031	0.090	0.730	0.854	0.008	0.094	0.936	0.965	0.163	0.200	0.415	0.893
vanillylmandelate (VMA)	-0.021	0.092	0.824	0.912	-0.009	0.096	0.928	0.962	0.046	0.205	0.822	0.988
X - 07765	0.051	0.088	0.566	0.742	0.052	0.093	0.578	0.761	0.021	0.204	0.919	0.992
X - 09789	-0.071	0.081	0.386	0.598	-0.042	0.085	0.619	0.778	0.104	0.181	0.566	0.947
X - 10458	0.177	0.088	0.045	0.160	0.245	0.091	0.008	0.048	0.367	0.195	0.062	0.819
X - 11261	0.184	0.069	0.009	0.050	0.135	0.073	0.065	0.207	-0.153	0.154	0.321	0.875
X - 11308	-0.094	0.067	0.158	0.355	-0.128	0.069	0.066	0.208	-0.187	0.148	0.208	0.841
X - 11315	-0.144	0.088	0.103	0.277	-0.140	0.092	0.130	0.325	-0.030	0.198	0.880	0.992
X - 11372	-0.101	0.071	0.159	0.356	-0.125	0.074	0.093	0.266	-0.147	0.158	0.355	0.886
X - 11378	-0.082	0.070	0.243	0.462	-0.111	0.073	0.130	0.325	-0.161	0.156	0.305	0.873
X - 11381	-0.092	0.097	0.345	0.560	-0.039	0.104	0.709	0.845	0.233	0.226	0.306	0.873
X - 11407	-0.153	0.075	0.044	0.156	-0.128	0.079	0.105	0.285	0.056	0.168	0.740	0.981
X - 11441	-0.202	0.085	0.018	0.088	-0.206	0.089	0.021	0.096	-0.091	0.190	0.631	0.974
X - 11444	-0.085	0.085	0.323	0.542	-0.076	0.089	0.394	0.627	0.010	0.193	0.961	0.994
X - 11470	-0.169	0.089	0.060	0.196	-0.122	0.092	0.185	0.405	0.133	0.193	0.491	0.911
X - 11478	0.021	0.073	0.776	0.881	-0.020	0.076	0.796	0.903	-0.174	0.161	0.280	0.869
X - 11491	0.086	0.093	0.359	0.574	0.131	0.097	0.180	0.400	0.234	0.208	0.262	0.848
X - 11522	-0.201	0.081	0.014	0.070	-0.238	0.084	0.005	0.032	-0.240	0.179	0.183	0.839
X - 11530	-0.206	0.079	0.010	0.056	-0.243	0.082	0.003	0.024	-0.246	0.175	0.162	0.819
X - 11632	0.179	0.086	0.039	0.145	0.178	0.091	0.052	0.181	0.050	0.196	0.798	0.988
X - 11787	0.100	0.073	0.172	0.376	0.161	0.076	0.035	0.138	0.312	0.161	0.055	0.819
X - 11795	-0.040	0.072	0.579	0.751	-0.057	0.075	0.447	0.666	-0.093	0.161	0.566	0.947
X - 11843	-0.104	0.091	0.254	0.472	-0.039	0.095	0.681	0.830	0.265	0.203	0.194	0.839
X - 11847	0.157	0.073	0.033	0.131	0.181	0.076	0.019	0.088	0.163	0.163	0.319	0.875
X - 11849	0.133	0.071	0.061	0.199	0.165	0.074	0.027	0.116	0.190	0.160	0.237	0.845
X - 11850	-0.197	0.093	0.036	0.136	-0.138	0.101	0.173	0.388	0.222	0.213	0.298	0.873
X - 11852	-0.003	0.109	0.981	0.990	-0.010	0.116	0.930	0.962	-0.042	0.272	0.878	0.992
X - 11858	0.145	0.084	0.086	0.245	0.156	0.090	0.085	0.249	0.084	0.189	0.657	0.980
X - 11861	0.069	0.115	0.549	0.726	0.124	0.119	0.300	0.540	0.311	0.273	0.259	0.848
X - 11880	-0.040	0.065	0.532	0.713	-0.048	0.067	0.472	0.686	-0.051	0.144	0.722	0.980
X - 12007	0.117	0.089	0.190	0.395	0.091	0.094	0.334	0.574	-0.078	0.197	0.694	0.980

X - 12013	-0.088	0.111	0.428	0.631	-0.085	0.117	0.467	0.686	-0.016	0.245	0.949	0.993
X - 12015	0.166	0.105	0.115	0.298	0.081	0.108	0.456	0.676	-0.321	0.229	0.163	0.819
X - 12026	0.006	0.085	0.945	0.973	0.068	0.088	0.440	0.662	0.287	0.188	0.129	0.819
X - 12093 - retired for N2-acetyl,N6-methyllysine	-0.128	0.102	0.211	0.424	-0.147	0.106	0.169	0.383	-0.141	0.247	0.568	0.947
X - 12100	0.019	0.089	0.828	0.914	0.048	0.092	0.600	0.769	0.140	0.197	0.479	0.902
X - 12101	0.107	0.077	0.165	0.364	0.091	0.080	0.259	0.490	-0.035	0.171	0.837	0.988
X - 12104	0.019	0.102	0.853	0.924	0.095	0.108	0.382	0.620	0.388	0.242	0.111	0.819
X - 12112	-0.013	0.084	0.877	0.932	0.014	0.088	0.874	0.944	0.115	0.185	0.536	0.943
X - 12117	0.327	0.136	0.019	0.090	0.336	0.135	0.015	0.077	0.222	0.296	0.456	0.902
X - 12127	0.009	0.107	0.935	0.969	0.062	0.110	0.571	0.758	0.230	0.226	0.310	0.873
X - 12193	0.109	0.086	0.206	0.417	0.116	0.090	0.200	0.423	0.069	0.204	0.738	0.981
X - 12206	0.014	0.087	0.873	0.931	0.029	0.091	0.747	0.871	0.075	0.195	0.700	0.980
X - 12212	0.002	0.080	0.984	0.990	0.048	0.085	0.579	0.761	0.230	0.191	0.230	0.844
X - 12216	-0.105	0.092	0.257	0.476	-0.048	0.095	0.615	0.777	0.210	0.201	0.296	0.873
X - 12221	-0.151	0.115	0.192	0.399	-0.124	0.115	0.285	0.521	0.044	0.247	0.858	0.988
X - 12230	-0.030	0.084	0.719	0.846	-0.036	0.088	0.688	0.833	-0.035	0.194	0.859	0.988
X - 12261	0.338	0.470	0.489	0.683	0.060	0.679	0.932	0.962	-1.910	1.457	0.223	0.844
X - 12283	0.099	0.099	0.318	0.541	0.166	0.102	0.105	0.285	0.299	0.200	0.137	0.819
X - 12306	-0.023	0.108	0.831	0.916	-0.032	0.116	0.787	0.900	-0.048	0.268	0.857	0.988
X - 12329	0.191	0.111	0.088	0.250	0.126	0.114	0.272	0.505	-0.202	0.239	0.400	0.893
X - 12407	-0.106	0.173	0.544	0.722	-0.372	0.191	0.059	0.197	-0.906	0.337	0.011	0.738
X - 12410	0.171	0.095	0.074	0.218	0.117	0.101	0.247	0.477	-0.251	0.233	0.284	0.869
X - 12462	0.049	0.074	0.510	0.701	0.050	0.077	0.523	0.732	0.020	0.166	0.904	0.992
X - 12472	0.080	0.083	0.336	0.554	0.059	0.085	0.486	0.701	-0.050	0.173	0.774	0.984
X - 12543	-0.177	0.170	0.305	0.527	-0.190	0.160	0.241	0.473	-0.221	0.388	0.572	0.947
X - 12544	-0.112	0.143	0.435	0.636	-0.141	0.152	0.355	0.591	-0.141	0.286	0.623	0.972
X - 12707	-0.159	0.080	0.048	0.167	-0.120	0.084	0.155	0.365	0.121	0.178	0.497	0.916
X - 12718	0.214	0.117	0.070	0.212	0.129	0.119	0.280	0.515	-0.302	0.258	0.244	0.847
X - 12729	0.155	0.082	0.062	0.199	0.141	0.086	0.102	0.281	-0.005	0.183	0.976	0.995
X - 12730	-0.069	0.119	0.562	0.740	-0.007	0.120	0.952	0.971	0.262	0.263	0.321	0.875
X - 12739	-0.011	0.096	0.912	0.953	0.058	0.100	0.565	0.755	0.305	0.212	0.154	0.819
X - 12740	-0.063	0.108	0.563	0.740	-0.026	0.116	0.824	0.922	0.193	0.272	0.480	0.902
X - 12753	-0.037	0.113	0.743	0.860	-0.057	0.114	0.622	0.780	-0.095	0.230	0.679	0.980

X - 12812	0.048	0.120	0.689	0.819	-0.031	0.122	0.802	0.907	-0.308	0.249	0.219	0.844
X - 12822	0.187	0.091	0.042	0.151	0.190	0.095	0.047	0.169	0.082	0.204	0.687	0.980
X - 12844	0.018	0.082	0.827	0.914	0.046	0.086	0.598	0.767	0.133	0.185	0.474	0.902
X - 12846	0.196	0.081	0.017	0.081	0.191	0.084	0.025	0.109	0.050	0.179	0.782	0.988
X - 12847	-0.172	0.120	0.156	0.353	-0.104	0.125	0.408	0.635	0.280	0.280	0.320	0.875
X - 12849	0.196	0.137	0.157	0.353	0.178	0.141	0.211	0.435	-0.029	0.341	0.933	0.993
X - 12851	-0.193	0.137	0.162	0.358	-0.115	0.148	0.441	0.662	0.216	0.277	0.439	0.900
X - 12879	0.102	0.095	0.282	0.504	0.057	0.099	0.566	0.756	-0.171	0.212	0.422	0.893
X - 12906	0.104	0.085	0.221	0.437	0.099	0.090	0.271	0.505	0.007	0.194	0.972	0.995
X - 13431	0.174	0.080	0.032	0.129	0.160	0.084	0.058	0.196	0.000	0.179	0.998	0.999
X - 13507	0.074	0.101	0.466	0.666	0.015	0.108	0.892	0.949	-0.270	0.237	0.257	0.848
X - 13553	-0.040	0.073	0.585	0.755	0.009	0.076	0.905	0.955	0.209	0.162	0.199	0.841
X - 13658	0.039	0.093	0.672	0.809	-0.085	0.098	0.384	0.620	-0.560	0.204	0.007	0.738
X - 13684	0.187	0.083	0.026	0.111	0.175	0.087	0.046	0.168	0.012	0.186	0.947	0.993
X - 13695	0.133	0.087	0.128	0.319	0.171	0.089	0.057	0.193	0.225	0.189	0.235	0.844
X - 13729	-0.040	0.089	0.652	0.798	-0.051	0.091	0.576	0.761	-0.070	0.201	0.729	0.980
X - 13835	0.074	0.112	0.508	0.700	-0.038	0.118	0.747	0.871	-0.431	0.235	0.070	0.819
X - 13866	0.133	0.074	0.073	0.218	0.111	0.077	0.153	0.362	-0.053	0.165	0.748	0.982
X - 14056	0.145	0.083	0.081	0.236	0.179	0.086	0.038	0.144	0.209	0.184	0.256	0.848
X - 14662	0.004	0.081	0.959	0.982	-0.010	0.085	0.906	0.955	-0.067	0.187	0.721	0.980
X - 14939	0.107	0.073	0.143	0.338	0.084	0.076	0.269	0.503	-0.065	0.162	0.690	0.980
X - 15220	-0.149	0.124	0.234	0.450	-0.145	0.136	0.292	0.529	0.017	0.322	0.959	0.994
X - 15245	0.026	0.095	0.783	0.883	0.091	0.095	0.339	0.579	0.350	0.216	0.108	0.819
X - 15461	0.213	0.082	0.010	0.055	0.213	0.085	0.013	0.068	0.080	0.182	0.661	0.980
X - 15469	-0.034	0.083	0.685	0.816	-0.006	0.087	0.941	0.968	0.113	0.185	0.544	0.947
X - 15486	0.175	0.067	0.010	0.054	0.114	0.071	0.110	0.291	-0.214	0.148	0.150	0.819
X - 15492	0.149	0.072	0.041	0.149	0.166	0.075	0.028	0.118	0.133	0.161	0.410	0.893
X - 15503	0.229	0.089	0.011	0.059	0.256	0.092	0.006	0.038	0.207	0.197	0.295	0.873
X - 15664	0.020	0.104	0.845	0.921	-0.024	0.109	0.825	0.922	-0.180	0.224	0.423	0.893
X - 15666	0.020	0.081	0.808	0.900	-0.046	0.084	0.587	0.765	-0.292	0.179	0.105	0.819
X - 15728	-0.126	0.107	0.242	0.461	-0.130	0.113	0.253	0.483	-0.056	0.250	0.824	0.988
X - 16087	0.101	0.090	0.262	0.483	0.052	0.094	0.584	0.765	-0.225	0.213	0.294	0.873
X - 16124	0.113	0.128	0.379	0.594	0.229	0.133	0.087	0.253	0.662	0.306	0.033	0.738

X - 16397	0.130	0.089	0.148	0.344	0.144	0.091	0.117	0.303	0.122	0.197	0.535	0.943
X - 16570	0.039	0.084	0.646	0.793	-0.047	0.087	0.590	0.765	-0.385	0.187	0.041	0.783
X - 16576	-0.060	0.145	0.681	0.814	0.067	0.156	0.669	0.820	0.635	0.345	0.071	0.819
X - 16580	0.104	0.083	0.211	0.424	0.088	0.086	0.311	0.551	-0.036	0.184	0.847	0.988
X - 16649	0.031	0.102	0.764	0.877	-0.002	0.106	0.985	0.989	-0.146	0.233	0.533	0.943
X - 16654	0.119	0.113	0.294	0.517	0.193	0.120	0.112	0.294	0.379	0.263	0.153	0.819
X - 16932	0.029	0.114	0.804	0.897	-0.085	0.117	0.471	0.686	-0.425	0.228	0.065	0.819
X - 16935	-0.100	0.067	0.140	0.336	-0.132	0.070	0.060	0.200	-0.184	0.150	0.222	0.844
X - 16938	-0.006	0.082	0.942	0.972	-0.011	0.085	0.895	0.950	-0.027	0.183	0.885	0.992
X - 16944	0.123	0.073	0.094	0.261	0.102	0.077	0.183	0.402	-0.052	0.163	0.753	0.982
X - 16964	0.095	0.082	0.248	0.468	0.122	0.085	0.152	0.361	0.160	0.182	0.380	0.888
X - 17010	-0.074	0.074	0.320	0.542	-0.085	0.077	0.273	0.505	-0.077	0.165	0.644	0.980
X - 17137	0.008	0.103	0.942	0.972	-0.045	0.109	0.682	0.830	-0.217	0.224	0.333	0.880
X - 17145	-0.015	0.080	0.847	0.922	-0.032	0.084	0.706	0.845	-0.079	0.179	0.660	0.980
X - 17162	-0.088	0.123	0.477	0.674	-0.034	0.131	0.796	0.903	0.219	0.278	0.434	0.900
X - 17185	0.027	0.078	0.725	0.851	-0.013	0.081	0.876	0.944	-0.184	0.177	0.301	0.873
X - 17269	0.134	0.086	0.122	0.307	0.139	0.091	0.130	0.325	0.062	0.193	0.749	0.982
X - 17325	-0.238	0.106	0.028	0.118	-0.145	0.116	0.214	0.438	0.412	0.252	0.106	0.819
X - 17335	0.076	0.084	0.367	0.584	0.088	0.088	0.318	0.559	0.081	0.188	0.668	0.980
X - 17337	0.200	0.077	0.010	0.055	0.193	0.080	0.017	0.082	0.042	0.171	0.809	0.988
X - 17351	0.110	0.096	0.253	0.472	0.191	0.098	0.053	0.184	0.389	0.201	0.055	0.819
X - 17357	0.139	0.092	0.132	0.327	0.232	0.094	0.015	0.075	0.467	0.199	0.020	0.738
X - 17359	0.211	0.085	0.014	0.073	0.238	0.088	0.007	0.044	0.210	0.189	0.268	0.854
X - 17365	0.074	0.167	0.662	0.805	0.064	0.173	0.712	0.847	-0.026	0.414	0.950	0.993
X - 17367	-0.302	0.124	0.017	0.083	-0.192	0.135	0.159	0.373	0.448	0.285	0.120	0.819
X - 17438	-0.179	0.078	0.023	0.102	-0.216	0.081	0.009	0.049	-0.232	0.174	0.183	0.839
X - 17612	0.161	0.107	0.135	0.330	0.154	0.116	0.186	0.405	-0.021	0.274	0.939	0.993
X - 17653	-0.085	0.067	0.206	0.417	-0.116	0.069	0.096	0.271	-0.173	0.148	0.243	0.847
X - 17654	-0.139	0.068	0.041	0.150	-0.155	0.070	0.029	0.120	-0.121	0.151	0.423	0.893
X - 17655	-0.112	0.139	0.424	0.629	-0.037	0.145	0.801	0.907	0.308	0.312	0.327	0.879
X - 17673	0.218	0.190	0.267	0.486	0.261	0.216	0.243	0.474	0.214	0.584	0.719	0.980
X - 17676	0.190	0.087	0.030	0.124	0.148	0.091	0.105	0.285	-0.127	0.196	0.519	0.941
X - 17685	0.230	0.120	0.058	0.192	0.233	0.126	0.067	0.210	0.089	0.280	0.751	0.982

X - 18249	0.121	0.061	0.047	0.164	0.136	0.063	0.033	0.132	0.109	0.135	0.423	0.893
X - 18888	0.073	0.087	0.402	0.612	0.025	0.093	0.788	0.900	-0.187	0.193	0.333	0.880
X - 18899	-0.036	0.072	0.616	0.776	-0.031	0.076	0.682	0.830	0.011	0.161	0.944	0.993
X - 18901	-0.030	0.087	0.733	0.855	0.024	0.090	0.794	0.903	0.232	0.192	0.229	0.844
X - 18913	0.096	0.079	0.226	0.443	0.095	0.082	0.246	0.477	0.034	0.175	0.845	0.988
X - 18914	0.090	0.067	0.179	0.384	0.081	0.070	0.247	0.477	-0.009	0.149	0.951	0.993
X - 18921	0.027	0.074	0.715	0.842	0.016	0.077	0.834	0.927	-0.040	0.165	0.809	0.988
X - 19141	0.069	0.086	0.420	0.624	0.165	0.089	0.066	0.208	0.479	0.194	0.014	0.738
X - 19183	0.315	0.351	0.387	0.598	0.259	0.425	0.553	0.749	-0.556	1.099	0.623	0.972
X - 19438	0.135	0.078	0.087	0.249	0.173	0.082	0.036	0.142	0.229	0.182	0.210	0.841
X - 21258	0.156	0.064	0.016	0.078	0.227	0.065	0.001	0.006	0.384	0.139	0.007	0.738
X - 21286	-0.085	0.086	0.324	0.542	-0.041	0.089	0.647	0.799	0.164	0.188	0.385	0.889
X - 21295	-0.003	0.099	0.976	0.990	0.017	0.102	0.867	0.943	0.094	0.223	0.674	0.980
X - 21310	-0.156	0.083	0.062	0.199	-0.081	0.087	0.352	0.589	0.284	0.184	0.124	0.819
X - 21315	-0.582	0.220	0.029	0.121	-0.593	0.213	0.024	0.105	-0.430	0.668	0.540	0.947
X - 21319	0.032	0.075	0.673	0.809	0.004	0.078	0.963	0.976	-0.117	0.167	0.486	0.905
X - 21339	0.022	0.049	0.656	0.803	-0.029	0.052	0.572	0.758	-0.227	0.109	0.040	0.773
X - 21342	0.082	0.072	0.250	0.471	0.083	0.075	0.265	0.497	0.034	0.160	0.833	0.988
X - 21353	-0.014	0.082	0.867	0.930	-0.009	0.085	0.915	0.959	0.016	0.183	0.931	0.993
X - 21364	0.082	0.063	0.198	0.407	0.075	0.066	0.254	0.483	0.001	0.140	0.995	0.999
X - 21410	0.150	0.086	0.082	0.239	0.121	0.090	0.183	0.402	-0.089	0.196	0.651	0.980
X - 21411	0.075	0.083	0.368	0.584	0.001	0.087	0.994	0.995	-0.313	0.184	0.090	0.819
X - 21441	-0.055	0.079	0.487	0.683	-0.097	0.084	0.247	0.477	-0.204	0.178	0.251	0.848
X - 21448	-0.204	0.079	0.011	0.059	-0.200	0.083	0.017	0.082	-0.053	0.177	0.765	0.982
X - 21467	0.032	0.079	0.682	0.814	0.090	0.082	0.272	0.505	0.276	0.175	0.116	0.819
X - 21470	0.103	0.076	0.179	0.384	0.078	0.079	0.323	0.566	-0.067	0.166	0.685	0.980
X - 21471	0.055	0.079	0.486	0.683	0.108	0.081	0.187	0.407	0.260	0.173	0.135	0.819
X - 21607	0.052	0.085	0.536	0.716	0.078	0.088	0.376	0.616	0.133	0.186	0.474	0.902
X - 21661	0.099	0.091	0.278	0.501	0.105	0.100	0.295	0.533	0.042	0.208	0.841	0.988
X - 21729	0.078	0.083	0.349	0.563	0.159	0.086	0.066	0.208	0.399	0.183	0.031	0.738
X - 21733	-0.118	0.105	0.264	0.485	-0.136	0.108	0.210	0.434	-0.131	0.229	0.570	0.947
X - 21736	0.190	0.075	0.012	0.065	0.204	0.079	0.010	0.056	0.131	0.168	0.439	0.900
X - 21785	0.197	0.077	0.011	0.059	0.205	0.080	0.011	0.061	0.108	0.171	0.527	0.942

X - 21792	0.023	0.086	0.787	0.886	0.043	0.089	0.627	0.783	0.099	0.189	0.601	0.963
X - 21796	0.052	0.076	0.495	0.688	0.083	0.079	0.297	0.537	0.159	0.170	0.350	0.885
X - 21803	-0.244	0.254	0.348	0.563	-0.142	0.241	0.563	0.755	0.270	0.515	0.607	0.963
X - 21807	-0.029	0.162	0.861	0.929	0.016	0.181	0.931	0.962	0.209	0.397	0.601	0.963
X - 21821	0.125	0.098	0.205	0.417	0.188	0.100	0.061	0.200	0.303	0.197	0.125	0.819
X - 21834	-0.010	0.091	0.915	0.953	-0.001	0.096	0.991	0.993	0.040	0.215	0.851	0.988
X - 21959	-0.103	0.075	0.174	0.378	-0.131	0.078	0.095	0.269	-0.167	0.167	0.320	0.875
X - 22143	0.214	0.105	0.043	0.154	0.143	0.109	0.191	0.412	-0.236	0.230	0.307	0.873
X - 22162	0.011	0.087	0.901	0.948	0.047	0.090	0.601	0.769	0.170	0.193	0.379	0.888
X - 22519	-0.059	0.080	0.461	0.660	-0.020	0.083	0.811	0.914	0.156	0.177	0.379	0.888
X - 22520	0.237	0.152	0.124	0.312	0.190	0.149	0.205	0.428	-0.057	0.314	0.857	0.988
X - 22764	-0.105	0.083	0.209	0.420	-0.101	0.087	0.246	0.477	-0.020	0.186	0.916	0.992
X - 22771	0.067	0.083	0.420	0.624	0.107	0.086	0.215	0.438	0.208	0.184	0.261	0.848
X - 22776	0.166	0.086	0.054	0.184	0.212	0.089	0.018	0.088	0.269	0.192	0.164	0.819
X - 22834	-0.210	0.162	0.202	0.413	-0.126	0.170	0.460	0.679	0.207	0.319	0.520	0.941
X - 22918	0.257	0.233	0.289	0.512	0.292	0.279	0.313	0.553	0.116	0.534	0.831	0.988
X - 23160	0.081	0.116	0.486	0.683	0.060	0.123	0.631	0.786	-0.075	0.263	0.777	0.985
X - 23196	-0.016	0.080	0.838	0.920	-0.087	0.083	0.294	0.532	-0.330	0.176	0.063	0.819
X - 23276	-0.109	0.073	0.137	0.333	-0.150	0.076	0.049	0.174	-0.225	0.162	0.166	0.823
X - 23295	0.047	0.071	0.511	0.701	0.007	0.074	0.929	0.962	-0.169	0.159	0.291	0.873
X - 23296	-0.026	0.155	0.868	0.930	-0.045	0.172	0.795	0.903	-0.081	0.347	0.817	0.988
X - 23297	-0.137	0.083	0.101	0.276	-0.138	0.087	0.114	0.298	-0.054	0.183	0.767	0.982
X - 23314	-0.010	0.088	0.910	0.953	-0.043	0.092	0.645	0.797	-0.159	0.202	0.431	0.898
X - 23369	0.116	0.085	0.174	0.378	0.142	0.088	0.110	0.291	0.160	0.188	0.398	0.893
X - 23498	0.131	0.098	0.184	0.392	0.130	0.103	0.209	0.434	0.033	0.241	0.891	0.992
X - 23507	0.168	0.102	0.102	0.276	0.153	0.101	0.132	0.328	0.029	0.215	0.892	0.992
X - 23583	-0.197	0.220	0.376	0.591	-0.159	0.217	0.467	0.686	0.056	0.471	0.907	0.992
X - 23585	-0.102	0.094	0.282	0.503	-0.170	0.098	0.084	0.247	-0.369	0.216	0.090	0.819
X - 23587	0.027	0.088	0.756	0.871	-0.006	0.093	0.952	0.971	-0.145	0.200	0.472	0.902
X - 23636	0.088	0.084	0.293	0.517	0.112	0.087	0.199	0.423	0.141	0.187	0.450	0.902
X - 23639	-0.124	0.088	0.161	0.357	-0.047	0.092	0.610	0.773	0.305	0.195	0.119	0.819
X - 23644	0.121	0.075	0.106	0.285	0.107	0.078	0.169	0.384	-0.017	0.165	0.919	0.992
X - 23648	0.154	0.100	0.125	0.315	0.073	0.104	0.484	0.700	-0.315	0.221	0.158	0.819

X - 23662	0.119	0.159	0.455	0.656	0.066	0.175	0.710	0.845	-0.185	0.347	0.596	0.961
X - 23665	0.117	0.091	0.199	0.409	0.135	0.097	0.168	0.382	0.113	0.218	0.607	0.963
X - 23666	0.122	0.090	0.179	0.384	0.130	0.092	0.162	0.377	0.090	0.206	0.662	0.980
X - 23739	0.097	0.086	0.261	0.482	0.074	0.090	0.408	0.635	-0.068	0.191	0.724	0.980
X - 23767	-0.063	0.128	0.622	0.779	-0.070	0.132	0.596	0.765	-0.059	0.291	0.840	0.988
X - 23780	0.141	0.069	0.041	0.150	0.126	0.071	0.078	0.233	-0.010	0.150	0.946	0.993
X - 23782	0.000	0.073	0.996	0.998	-0.031	0.076	0.685	0.832	-0.139	0.163	0.393	0.891
X - 23787	0.096	0.150	0.524	0.710	0.101	0.160	0.528	0.735	0.050	0.349	0.887	0.992
X - 23974	0.008	0.078	0.915	0.953	-0.034	0.081	0.680	0.830	-0.186	0.172	0.282	0.869
X - 23997	-0.059	0.089	0.504	0.697	-0.014	0.091	0.880	0.944	0.180	0.193	0.351	0.885
X - 24077	-0.075	0.072	0.297	0.519	-0.045	0.075	0.554	0.749	0.113	0.160	0.481	0.902
X - 24241	-0.001	0.081	0.987	0.991	0.015	0.084	0.861	0.941	0.074	0.181	0.685	0.980
X - 24243	0.050	0.084	0.550	0.727	0.096	0.089	0.285	0.521	0.233	0.197	0.238	0.845
X - 24293	0.120	0.080	0.139	0.335	0.061	0.084	0.469	0.686	-0.243	0.183	0.188	0.839
X - 24295	0.114	0.082	0.164	0.362	0.130	0.085	0.127	0.322	0.115	0.182	0.527	0.942
X - 24306	-0.011	0.073	0.877	0.932	-0.030	0.076	0.699	0.838	-0.086	0.162	0.595	0.961
X - 24307	-0.021	0.071	0.769	0.878	-0.048	0.074	0.519	0.730	-0.130	0.158	0.411	0.893
X - 24337	0.213	0.080	0.009	0.051	0.200	0.084	0.018	0.087	0.020	0.179	0.913	0.992
X - 24344	-0.008	0.215	0.971	0.988	-0.070	0.210	0.743	0.870	-0.298	0.463	0.525	0.942
X - 24411	0.216	0.141	0.130	0.322	0.232	0.142	0.108	0.289	0.174	0.303	0.567	0.947
X - 24418	0.396	0.204	0.065	0.204	0.337	0.187	0.085	0.249	0.024	0.510	0.963	0.994
X - 24422	-0.032	0.076	0.673	0.809	-0.076	0.079	0.336	0.575	-0.211	0.167	0.210	0.841
X - 24431	-0.053	0.089	0.553	0.729	-0.088	0.092	0.343	0.581	-0.182	0.199	0.363	0.888
X - 24432	0.044	0.129	0.735	0.855	-0.098	0.135	0.472	0.686	-0.622	0.278	0.029	0.738
X - 24435	0.207	0.081	0.011	0.060	0.195	0.085	0.022	0.100	0.020	0.180	0.911	0.992
X - 24455	0.063	0.098	0.519	0.708	0.006	0.103	0.954	0.972	-0.264	0.228	0.249	0.848
X - 24456	0.242	0.143	0.095	0.264	0.220	0.147	0.140	0.344	-0.012	0.332	0.971	0.995
X - 24473	0.012	0.081	0.883	0.937	0.041	0.084	0.626	0.782	0.137	0.179	0.446	0.902
X - 24475	-0.018	0.082	0.827	0.914	-0.044	0.086	0.607	0.771	-0.128	0.185	0.491	0.911
X - 24518	0.234	0.142	0.103	0.277	0.182	0.142	0.204	0.428	-0.064	0.273	0.817	0.988
X - 24527	0.261	0.153	0.093	0.260	0.293	0.160	0.071	0.216	0.227	0.320	0.481	0.902
X - 24540	-0.020	0.099	0.838	0.920	0.053	0.099	0.594	0.765	0.402	0.233	0.087	0.819
X - 24542	0.014	0.099	0.892	0.942	0.069	0.105	0.512	0.725	0.255	0.224	0.256	0.848

X - 24544	0.140	0.075	0.062	0.199	0.072	0.080	0.366	0.604	-0.260	0.166	0.119	0.819
X - 24546	0.120	0.083	0.149	0.345	0.070	0.089	0.431	0.652	-0.175	0.181	0.337	0.883
X - 24549	0.051	0.090	0.574	0.746	0.054	0.095	0.573	0.758	0.029	0.207	0.889	0.992
X - 24556	0.089	0.077	0.245	0.465	0.081	0.080	0.310	0.550	-0.004	0.171	0.983	0.997
X - 24565	0.098	0.095	0.303	0.525	0.094	0.097	0.333	0.574	0.028	0.199	0.887	0.992
X - 24608	0.009	0.105	0.933	0.967	0.070	0.109	0.522	0.732	0.284	0.234	0.226	0.844
X - 24637	0.018	0.111	0.869	0.930	-0.070	0.117	0.550	0.747	-0.375	0.241	0.123	0.819
X - 24686	0.198	0.092	0.032	0.130	0.268	0.096	0.006	0.037	0.378	0.204	0.067	0.819
X - 24699	0.111	0.087	0.204	0.417	0.089	0.091	0.330	0.571	-0.061	0.195	0.756	0.982
X - 24727	0.022	0.134	0.867	0.930	0.082	0.143	0.568	0.757	0.272	0.303	0.372	0.888
X - 24736	0.081	0.112	0.472	0.671	0.042	0.117	0.719	0.852	-0.148	0.250	0.556	0.947
X - 24748	0.136	0.075	0.070	0.212	0.196	0.077	0.012	0.061	0.326	0.164	0.049	0.819
X - 24765	0.039	0.087	0.657	0.803	0.061	0.090	0.500	0.712	0.116	0.193	0.547	0.947
X - 24812	0.011	0.092	0.908	0.953	-0.031	0.092	0.734	0.864	-0.186	0.196	0.345	0.885
X - 24813	0.046	0.082	0.572	0.745	0.091	0.085	0.288	0.525	0.220	0.182	0.230	0.844
X - 24849	-0.204	0.082	0.013	0.069	-0.245	0.085	0.004	0.029	-0.259	0.181	0.155	0.819
X - 24947	-0.056	0.073	0.449	0.651	-0.091	0.076	0.234	0.463	-0.180	0.162	0.270	0.855
X - 24949	0.189	0.085	0.028	0.117	0.222	0.089	0.013	0.068	0.216	0.190	0.259	0.848
X - 24951	0.059	0.072	0.411	0.618	0.023	0.075	0.764	0.884	-0.149	0.161	0.358	0.887
X - 24952	0.096	0.077	0.214	0.427	0.073	0.080	0.362	0.600	-0.068	0.172	0.692	0.980
X - 24972	-0.142	0.083	0.088	0.249	-0.162	0.087	0.064	0.205	-0.137	0.185	0.460	0.902
X - 25172	-0.036	0.085	0.678	0.811	-0.015	0.089	0.862	0.941	0.079	0.190	0.679	0.980
X - 25220	0.111	0.097	0.257	0.476	0.044	0.099	0.658	0.809	-0.284	0.220	0.201	0.841
X - 25265	0.004	0.089	0.962	0.984	-0.002	0.093	0.983	0.989	-0.027	0.199	0.892	0.992
X - 25266	-0.071	0.073	0.330	0.550	-0.091	0.076	0.232	0.462	-0.116	0.163	0.477	0.902
X - 25267	-0.016	0.082	0.846	0.922	0.020	0.086	0.814	0.914	0.159	0.183	0.387	0.889
X - 25271	-0.002	0.077	0.984	0.990	0.081	0.081	0.318	0.559	0.373	0.170	0.030	0.738
X - 25279	0.022	0.079	0.784	0.883	0.002	0.084	0.978	0.988	-0.089	0.186	0.632	0.974
X - 25343	-0.128	0.076	0.091	0.256	-0.046	0.079	0.561	0.755	0.328	0.167	0.051	0.819
X - 25388	0.147	0.093	0.116	0.299	0.145	0.098	0.141	0.344	0.033	0.222	0.881	0.992
X - 25419	0.043	0.067	0.528	0.713	0.080	0.071	0.265	0.497	0.211	0.166	0.206	0.841
X - 25420	-0.136	0.069	0.051	0.175	-0.115	0.072	0.114	0.298	0.047	0.154	0.759	0.982
X - 25422	0.136	0.088	0.126	0.316	0.150	0.092	0.104	0.285	0.114	0.197	0.563	0.947

X - 25463	-0.077	0.083	0.358	0.574	-0.048	0.087	0.584	0.765	0.105	0.186	0.573	0.947
xanthine	0.178	0.083	0.034	0.133	0.186	0.087	0.034	0.135	0.099	0.186	0.594	0.961
xanthurenate	0.041	0.084	0.622	0.779	0.053	0.088	0.545	0.744	0.067	0.187	0.718	0.980
ximenoylcarnitine (C26:1)*	0.089	0.051	0.084	0.242	0.121	0.053	0.024	0.106	0.176	0.114	0.122	0.819
xylose	0.193	0.093	0.040	0.149	0.151	0.096	0.118	0.305	-0.105	0.203	0.607	0.963

Supplementary Table 5. Observational associations of metabolic biomarkers with NAFLD

Biochemical	HR	logHR	SE	p-valule	p-BH
FDR-adjusted p-value<0.05					
1-(1-enyl-palmitoyl)-2-oleoyl-GPC (P-16:0/18:1)*	0.551	-0.595	0.164	0.000	0.018
1-linoleoyl-GPG (18:2)*	1.859	0.620	0.184	0.001	0.031
1-myristoyl-2-palmitoyl-GPC (14:0/16:0)	1.920	0.652	0.168	0.000	0.010
1-palmitoyl-2-dihomo-linolenoyl-GPC (16:0/20:3n3 or 6)*	1.915	0.650	0.183	0.000	0.023
1-palmitoyl-2-oleoyl-GPE (16:0/18:1)	1.708	0.535	0.168	0.001	0.048
1-palmitoyl-2-palmitoleoyl-GPC (16:0/16:1)*	1.942	0.664	0.180	0.000	0.017
1-stearoyl-2-oleoyl-GPC (18:0/18:1)	1.899	0.641	0.200	0.001	0.047
2-hydroxybehenate	1.811	0.594	0.155	0.000	0.010
4-acetamidophenol	0.092	-2.391	0.605	0.000	0.009
4-cholesten-3-one	1.791	0.583	0.177	0.001	0.037
5-acetylamino-6-amino-3-methyluracil	0.486	-0.721	0.211	0.001	0.030
5alpha-androstan-3alpha,17beta-diol disulfate	2.934	1.076	0.319	0.001	0.031
androstenediol (3beta,17beta) monosulfate (2)	1.952	0.669	0.210	0.001	0.048
behenoyl dihydrosphingomyelin (d18:0/22:0)*	1.916	0.650	0.153	0.000	0.003
butyrylcarnitine (C4)	1.907	0.645	0.165	0.000	0.009
cysteine-glutathione disulfide	0.437	-0.827	0.181	0.000	0.001
diacylglycerol (14:0/18:1, 16:0/16:1) [2]*	1.707	0.535	0.163	0.001	0.037
glutamate	1.947	0.666	0.140	0.000	0.000
hexadecasphingosine (d16:1)*	1.885	0.634	0.186	0.001	0.030
nisinate (24:6n3)	1.753	0.562	0.160	0.000	0.024
phenylacetylglutamine	0.616	-0.485	0.143	0.001	0.030
sphingomyelin (d18:0/20:0, d16:0/22:0)*	1.741	0.554	0.151	0.000	0.018
X - 11381	1.938	0.662	0.187	0.000	0.023
X - 11850	0.575	-0.553	0.168	0.001	0.037
X - 12707	0.601	-0.509	0.153	0.001	0.034
X - 13729	0.329	-1.111	0.169	0.000	0.000
X - 21286	0.653	-0.426	0.128	0.001	0.034
X - 21785	1.820	0.599	0.156	0.000	0.010
X - 23997	0.512	-0.669	0.162	0.000	0.005

X - 24241	2.152	0.766	0.223	0.001	0.030
FDR-adjusted p-value>=0.05					
(14 or 15)-methylpalmitate (a17:0 or i17:0)	1.074	0.071	0.128	0.577	0.821
(16 or 17)-methylstearate (a19:0 or i19:0)	0.958	-0.043	0.145	0.769	0.919
(2,4 or 2,5)-dimethylphenol sulfate	1.151	0.141	0.189	0.458	0.748
(N(1) + N(8))-acetylspermidine	1.165	0.153	0.126	0.223	0.547
1-(1-enyl-oleoyl)-GPE (P-18:1)*	0.869	-0.141	0.168	0.404	0.710
1-(1-enyl-palmitoyl)-2-arachidonoyl-GPC (P-16:0/20:4)*	0.727	-0.319	0.144	0.027	0.211
1-(1-enyl-palmitoyl)-2-arachidonoyl-GPE (P-16:0/20:4)*	0.859	-0.152	0.158	0.336	0.664
1-(1-enyl-palmitoyl)-2-linoleoyl-GPC (P-16:0/18:2)*	0.730	-0.314	0.144	0.029	0.221
1-(1-enyl-palmitoyl)-2-linoleoyl-GPE (P-16:0/18:2)*	0.915	-0.089	0.141	0.530	0.788
1-(1-enyl-palmitoyl)-2-oleoyl-GPE (P-16:0/18:1)*	0.938	-0.064	0.153	0.677	0.878
1-(1-enyl-palmitoyl)-2-palmitoleoyl-GPC (P-16:0/16:1)*	0.648	-0.434	0.159	0.006	0.097
1-(1-enyl-palmitoyl)-2-palmitoyl-GPC (P-16:0/16:0)*	0.741	-0.299	0.150	0.047	0.281
1-(1-enyl-palmitoyl)-GPC (P-16:0)*	0.836	-0.179	0.151	0.236	0.554
1-(1-enyl-palmitoyl)-GPE (P-16:0)*	0.983	-0.018	0.156	0.910	0.973
1-(1-enyl-stearoyl)-2-arachidonoyl-GPE (P-18:0/20:4)*	0.737	-0.305	0.157	0.051	0.287
1-(1-enyl-stearoyl)-2-linoleoyl-GPE (P-18:0/18:2)*	1.066	0.064	0.159	0.686	0.882
1-(1-enyl-stearoyl)-2-oleoyl-GPE (P-18:0/18:1)	0.977	-0.024	0.194	0.903	0.970
1-(1-enyl-stearoyl)-GPE (P-18:0)*	0.837	-0.178	0.171	0.297	0.628
1,2,3-benzenetriol sulfate (2)	0.980	-0.020	0.121	0.871	0.966
1,2-dilinoleoyl-GPC (18:2/18:2)	1.176	0.162	0.149	0.276	0.603
1,2-dilinoleoyl-GPE (18:2/18:2)*	1.255	0.227	0.137	0.096	0.382
1,2-dipalmitoyl-GPC (16:0/16:0)	1.085	0.081	0.138	0.557	0.810
1,2-dipalmitoyl-GPE (16:0/16:0)*	1.303	0.265	0.158	0.094	0.381
1,3-dimethylurate	0.774	-0.256	0.181	0.157	0.474
1,5-anhydroglucitol (1,5-AG)	0.987	-0.013	0.138	0.922	0.973
1,7-dimethylurate	0.624	-0.471	0.182	0.010	0.120
10-heptadecenoate (17:1n7)	1.077	0.074	0.133	0.577	0.821
10-nonadecenoate (19:1n9)	1.036	0.035	0.128	0.783	0.924
10-undecenoate (11:1n1)	1.283	0.249	0.175	0.153	0.473
12,13-DiHOME	1.081	0.078	0.143	0.585	0.824

13-HODE + 9-HODE	1.058	0.057	0.139	0.683	0.882
16a-hydroxy DHEA 3-sulfate	1.480	0.392	0.169	0.020	0.174
16-hydroxypalmitate	1.022	0.022	0.135	0.870	0.966
17alpha-hydroxypregnenolone 3-sulfate	1.483	0.394	0.199	0.048	0.285
1-arachidonoyl-GPA (20:4)	0.873	-0.136	0.173	0.431	0.731
1-arachidonoyl-GPC* (20:4)*	0.883	-0.124	0.158	0.433	0.731
1-arachidonoyl-GPE (20:4n6)*	0.969	-0.031	0.176	0.860	0.960
1-arachidonoyl-GPI* (20:4)*	1.315	0.274	0.170	0.108	0.400
1-arachidonylglycerol (20:4)	0.978	-0.022	0.162	0.893	0.970
1-carboxyethylisoleucine	1.071	0.069	0.144	0.632	0.855
1-carboxyethylleucine	1.225	0.203	0.147	0.168	0.482
1-carboxyethylphenylalanine	1.317	0.275	0.142	0.052	0.287
1-carboxyethyltyrosine	1.128	0.121	0.171	0.482	0.760
1-carboxyethylvaline	1.432	0.359	0.151	0.017	0.162
1-dihomo-linolenylglycerol (20:3)	1.310	0.270	0.147	0.066	0.323
1-dihomo-linoleoylglycerol (20:2)	1.097	0.092	0.169	0.585	0.824
1-docosahexaenoylglycerol (22:6)	1.174	0.160	0.164	0.329	0.659
1H-indole-7-acetic acid	1.021	0.021	0.206	0.919	0.973
1-lignoceroyl-GPC (24:0)	1.167	0.155	0.190	0.415	0.718
1-linolenoylglycerol (18:3)	1.421	0.351	0.162	0.031	0.229
1-linolenoyl-GPC (18:3)*	1.512	0.413	0.164	0.012	0.139
1-linoleoyl-2-arachidonoyl-GPC (18:2/20:4n6)*	0.942	-0.059	0.145	0.681	0.880
1-linoleoyl-2-linolenoyl-GPC (18:2/18:3)*	1.241	0.216	0.154	0.160	0.477
1-linoleoylglycerol (18:2)	1.440	0.365	0.144	0.011	0.135
1-linoleoyl-GPA (18:2)*	0.969	-0.032	0.174	0.854	0.957
1-linoleoyl-GPC (18:2)	1.188	0.173	0.150	0.251	0.577
1-linoleoyl-GPE (18:2)*	1.354	0.303	0.144	0.035	0.243
1-linoleoyl-GPI* (18:2)*	1.411	0.345	0.163	0.035	0.242
1-methyl-4-imidazoleacetate	1.041	0.040	0.140	0.774	0.923
1-methyl-5-imidazoleacetate	0.730	-0.314	0.160	0.050	0.286
1-methyladenosine	1.190	0.174	0.139	0.212	0.538
1-methylhistidine	1.103	0.098	0.171	0.566	0.815
1-methylnicotinamide	1.317	0.276	0.162	0.089	0.376

1-methylurate	1.009	0.009	0.126	0.944	0.975
1-myristoyl-2-arachidonoyl-GPC (14:0/20:4)*	1.496	0.403	0.171	0.019	0.166
1-oleoyl-2-docosahexaenoyl-GPC (18:1/22:6)*	0.664	-0.409	0.146	0.005	0.091
1-oleoyl-2-linoleoyl-GPE (18:1/18:2)*	1.220	0.199	0.143	0.164	0.480
1-oleoylglycerol (18:1)	1.560	0.445	0.161	0.006	0.092
1-oleoylglycerophosphate (18:1)	0.785	-0.242	0.203	0.232	0.553
1-oleoyl-GPC (18:1)	1.026	0.026	0.200	0.896	0.970
1-oleoyl-GPE (18:1)	1.204	0.186	0.157	0.236	0.554
1-oleoyl-GPG (18:1)*	1.574	0.454	0.180	0.012	0.138
1-oleoyl-GPI (18:1)*	1.334	0.288	0.162	0.075	0.343
1-palmitoleoyl-2-linolenoyl-GPC (16:1/18:3)*	1.341	0.293	0.170	0.084	0.361
1-palmitoleoylglycerol (16:1)*	1.374	0.318	0.156	0.042	0.268
1-palmitoleoyl-GPC* (16:1)*	1.267	0.236	0.164	0.150	0.468
1-palmitoyl-2-arachidonoyl-GPC (16:0/20:4n6)	1.049	0.047	0.151	0.753	0.912
1-palmitoyl-2-arachidonoyl-GPE (16:0/20:4)*	1.230	0.207	0.150	0.167	0.482
1-palmitoyl-2-arachidonoyl-GPI (16:0/20:4)*	1.540	0.431	0.157	0.006	0.097
1-palmitoyl-2-docosahexaenoyl-GPC (16:0/22:6)	0.879	-0.129	0.141	0.360	0.678
1-palmitoyl-2-docosahexaenoyl-GPE (16:0/22:6)*	1.198	0.180	0.162	0.266	0.591
1-palmitoyl-2-linoleoyl-GPC (16:0/18:2)	1.548	0.437	0.152	0.004	0.077
1-palmitoyl-2-linoleoyl-GPE (16:0/18:2)	1.527	0.423	0.137	0.002	0.053
1-palmitoyl-2-linoleoyl-GPI (16:0/18:2)	1.522	0.420	0.155	0.007	0.097
1-palmitoyl-2-oleoyl-GPC (16:0/18:1)	1.784	0.579	0.199	0.004	0.073
1-palmitoyl-2-oleoyl-GPI (16:0/18:1)*	1.673	0.514	0.174	0.003	0.068
1-palmitoyl-2-stearoyl-GPC (16:0/18:0)	1.027	0.026	0.146	0.856	0.958
1-palmitoyl-2-stearoyl-GPE (16:0/18:0)*	1.155	0.144	0.151	0.339	0.664
1-palmitoyl-GPC (16:0)	1.297	0.260	0.137	0.057	0.296
1-palmitoyl-GPE (16:0)	1.232	0.208	0.140	0.138	0.442
1-palmitoyl-GPG (16:0)*	1.545	0.435	0.186	0.019	0.167
1-palmitoyl-GPI* (16:0)	1.329	0.285	0.159	0.074	0.342
1-ribosyl-imidazoleacetate*	0.981	-0.019	0.151	0.901	0.970
1-stearoyl-2-arachidonoyl-GPC (18:0/20:4)	0.963	-0.038	0.138	0.781	0.924
1-stearoyl-2-arachidonoyl-GPE (18:0/20:4)	1.168	0.155	0.159	0.331	0.659
1-stearoyl-2-arachidonoyl-GPI (18:0/20:4)	1.210	0.191	0.147	0.195	0.524

1-stearoyl-2-docosahexaenoyl-GPC (18:0/22:6)	0.956	-0.045	0.157	0.774	0.923
1-stearoyl-2-docosahexaenoyl-GPE (18:0/22:6)*	1.374	0.318	0.169	0.061	0.306
1-stearoyl-2-linoleoyl-GPC (18:0/18:2)*	1.274	0.242	0.141	0.085	0.361
1-stearoyl-2-linoleoyl-GPE (18:0/18:2)*	1.454	0.374	0.144	0.009	0.114
1-stearoyl-2-linoleoyl-GPI (18:0/18:2)	1.245	0.219	0.132	0.099	0.388
1-stearoyl-2-oleoyl-GPE (18:0/18:1)	1.434	0.361	0.153	0.019	0.166
1-stearoyl-2-oleoyl-GPI (18:0/18:1)*	1.208	0.189	0.149	0.206	0.535
1-stearoyl-2-oleoyl-GPS (18:0/18:1)	1.289	0.254	0.119	0.033	0.241
1-stearoyl-GPC (18:0)	1.274	0.242	0.156	0.121	0.420
1-stearoyl-GPE (18:0)	1.261	0.232	0.144	0.107	0.400
1-stearoyl-GPG (18:0)	1.546	0.436	0.163	0.007	0.105
1-stearoyl-GPI (18:0)	1.113	0.107	0.145	0.461	0.750
2,2'-Methylenebis(6-tert-butyl-p-cresol)	0.941	-0.060	0.130	0.642	0.861
2,3-dihydroxy-2-methylbutyrate	1.134	0.126	0.151	0.403	0.710
2,3-dihydroxy-5-methylthio-4-pentenoate (DMTPA)*	1.138	0.129	0.151	0.392	0.697
2,3-dihydroxyisovalerate	0.922	-0.081	0.148	0.583	0.824
21-hydroxypregnenolone disulfate	0.965	-0.036	0.153	0.815	0.934
2-aminoadipate	0.615	-0.486	0.228	0.033	0.241
2-aminobutyrate	1.181	0.166	0.182	0.362	0.678
2-aminoheptanoate	1.172	0.158	0.144	0.271	0.598
2-aminooctanoate	1.127	0.119	0.162	0.462	0.751
2-aminophenol sulfate	0.925	-0.078	0.184	0.673	0.876
2-butenoylglycine	0.770	-0.262	0.200	0.191	0.523
2'-deoxyuridine	0.878	-0.130	0.134	0.330	0.659
2-ethylphenylsulfate	0.995	-0.005	0.226	0.981	0.989
2-hydroxy-3-methylvalerate	1.290	0.255	0.137	0.064	0.318
2-hydroxyarachidate*	1.332	0.287	0.137	0.037	0.246
2-hydroxybutyrate/2-hydroxyisobutyrate	1.170	0.157	0.130	0.227	0.551
2-hydroxydecanoate	1.143	0.133	0.148	0.367	0.678
2-hydroxyfluorene sulfate	1.096	0.092	0.203	0.651	0.867
2-hydroxyglutarate	0.951	-0.050	0.194	0.798	0.926
2-hydroxyheptanoate*	1.020	0.019	0.105	0.853	0.957
2-hydroxyhippurate (salicylurate)	0.950	-0.051	0.157	0.743	0.912

2-hydroxylaurate	1.238	0.214	0.155	0.168	0.482
2-hydroxynervonate*	1.683	0.521	0.259	0.044	0.270
2-hydroxyoctanoate	1.558	0.443	0.150	0.003	0.067
2-hydroxypalmitate	1.264	0.234	0.133	0.078	0.349
2-hydroxyphenylacetate	1.006	0.006	0.147	0.967	0.983
2-hydroxystearate	1.299	0.262	0.138	0.057	0.296
2-isopropylmalate	0.741	-0.300	0.149	0.043	0.270
2-keto-3-deoxy-gluconate	1.040	0.039	0.144	0.785	0.924
2-linoleoylglycerol (18:2)	1.292	0.256	0.147	0.080	0.353
2-methylbutyrylcarnitine (C5)	1.228	0.206	0.161	0.201	0.530
2-methylcitrate/homocitrate	0.797	-0.227	0.203	0.264	0.590
2-methylmalonylcarnitine (C4-DC)	1.028	0.028	0.182	0.878	0.970
2-naphthol sulfate	1.326	0.282	0.180	0.117	0.416
2-oleoylglycerol (18:1)	1.343	0.295	0.152	0.053	0.288
2'-O-methylcytidine	0.901	-0.104	0.139	0.452	0.740
2'-O-methyluridine	0.847	-0.166	0.195	0.393	0.697
2-oxoarginine*	1.359	0.307	0.137	0.025	0.201
2-palmitoleoylglycerol (16:1)*	0.970	-0.030	0.151	0.840	0.948
2-palmitoleoyl-GPC* (16:1)*	0.930	-0.073	0.158	0.646	0.865
2-palmitoyl-GPC* (16:0)*	1.121	0.114	0.148	0.441	0.733
2-piperidinone	0.878	-0.130	0.139	0.350	0.677
2-stearoyl-GPE (18:0)*	1.128	0.120	0.148	0.418	0.718
3-(3-amino-3-carboxypropyl)uridine*	1.127	0.120	0.197	0.544	0.799
3-(3-hydroxyphenyl)propionate	1.283	0.249	0.196	0.204	0.532
3-(4-hydroxyphenyl)lactate (HPLA)	1.133	0.125	0.154	0.419	0.718
3-(methylthio)acetaminophen sulfate*	3.982	1.382	0.438	0.002	0.050
3,4-methyleneheptanoate	0.864	-0.146	0.204	0.474	0.760
3,4-methyleneheptanoylcarnitine	1.288	0.253	0.176	0.152	0.469
3-acetylphenol sulfate	1.320	0.278	0.193	0.150	0.468
3-amino-2-piperidone	0.923	-0.080	0.128	0.532	0.790
3-aminoisobutyrate	1.016	0.016	0.143	0.912	0.973
3beta,7alpha-dihydroxy-5-cholestenoate	1.658	0.506	0.210	0.016	0.155
3beta-hydroxy-5-cholestenoate	0.723	-0.324	0.160	0.043	0.269

3b-hydroxy-5-cholenoic acid	0.709	-0.344	0.162	0.034	0.242
3-carboxy-4-methyl-5-pentyl-2-furanpropionate (3-CMPFP)	1.014	0.014	0.186	0.940	0.975
3-carboxy-4-methyl-5-propyl-2-furanpropanoate (CMPF)	1.029	0.029	0.241	0.905	0.970
3-ethylcatechol sulfate (1)	1.648	0.500	0.263	0.057	0.296
3-ethylphenylsulfate	1.311	0.271	0.308	0.379	0.685
3-formylindole	1.299	0.262	0.131	0.045	0.272
3-hydroxy-2-ethylpropionate	1.172	0.159	0.138	0.250	0.577
3-hydroxy-3-methylglutarate	0.748	-0.290	0.155	0.061	0.308
3-hydroxybutyrate (BHBA)	0.905	-0.099	0.129	0.441	0.733
3-hydroxybutyroylglycine	0.771	-0.260	0.154	0.092	0.377
3-hydroxybutyrylcarnitine (1)	1.037	0.036	0.218	0.869	0.966
3-hydroxybutyrylcarnitine (2)	1.018	0.018	0.164	0.913	0.973
3-hydroxydecanoate	1.005	0.005	0.133	0.968	0.983
3-hydroxydodecanedioate*	0.841	-0.173	0.196	0.377	0.682
3-hydroxyhexanoate	1.179	0.164	0.131	0.209	0.536
3-hydroxyhippurate	1.052	0.051	0.144	0.725	0.901
3-hydroxyisobutyrate	1.208	0.189	0.132	0.151	0.468
3-hydroxylaurate	1.060	0.059	0.128	0.648	0.865
3-hydroxyoctanoate	0.946	-0.056	0.143	0.697	0.886
3-hydroxyoleoylcarnitine	1.130	0.122	0.155	0.431	0.731
3-hydroxypalmitoylcarnitine	1.619	0.482	0.215	0.025	0.201
3-hydroxypyridine sulfate	0.982	-0.018	0.154	0.906	0.970
3-hydroxysebacate	0.822	-0.196	0.160	0.221	0.547
3-hydroxystachydrine*	1.041	0.040	0.173	0.815	0.934
3-indoxyl sulfate	0.720	-0.328	0.124	0.008	0.109
3-methoxycatechol sulfate (1)	0.961	-0.040	0.122	0.745	0.912
3-methoxycatechol sulfate (2)	0.905	-0.100	0.198	0.614	0.842
3-methoxytyramine sulfate	1.013	0.013	0.160	0.936	0.975
3-methoxytyrosine	0.980	-0.020	0.135	0.881	0.970
3-methyl catechol sulfate (1)	1.128	0.121	0.179	0.499	0.767
3-methyl-2-oxobutyrate	1.320	0.278	0.155	0.073	0.341
3-methyl-2-oxovalerate	1.334	0.288	0.137	0.036	0.245
3-methyladipate	1.872	0.627	0.310	0.043	0.269

3-methylglutaconate	0.922	-0.082	0.142	0.565	0.814
3-methylglutarylcarnitine (2)	1.045	0.044	0.132	0.739	0.910
3-methylhistidine	0.925	-0.078	0.135	0.563	0.813
3-methylxanthine	0.555	-0.588	0.211	0.005	0.092
3-phenylpropionate (hydrocinnamate)	0.839	-0.176	0.136	0.195	0.524
3-phosphoglycerate	1.011	0.011	0.137	0.938	0.975
3-ureidopropionate	0.809	-0.212	0.188	0.260	0.586
4-acetamidobutanoate	0.951	-0.050	0.150	0.736	0.910
4-acetamidophenylglucuronide	0.692	-0.368	0.354	0.299	0.629
4-acetaminophen sulfate	1.602	0.471	0.311	0.129	0.431
4-acetylphenyl sulfate	0.805	-0.217	0.140	0.120	0.420
4-allylphenol sulfate	1.193	0.176	0.156	0.258	0.584
4-ethylphenyl sulfate	0.791	-0.235	0.141	0.095	0.381
4-guanidinobutanoate	1.246	0.220	0.142	0.122	0.420
4-hydroxy-2-oxoglutaric acid	1.034	0.034	0.227	0.882	0.970
4-hydroxychlorothalonil	0.985	-0.015	0.168	0.928	0.975
4-hydroxyglutamate	1.359	0.307	0.150	0.041	0.265
4-hydroxyhippurate	1.021	0.021	0.139	0.881	0.970
4-hydroxyphenylacetate	0.776	-0.254	0.146	0.083	0.358
4-hydroxyphenylacetylglutamine	0.998	-0.002	0.159	0.989	0.992
4-hydroxyphenylpyruvate	1.076	0.073	0.150	0.627	0.853
4-methoxyphenol sulfate	0.962	-0.039	0.189	0.835	0.945
4-methyl-2-oxopentanoate	1.287	0.253	0.130	0.052	0.287
4-methylcatechol sulfate	0.825	-0.192	0.146	0.190	0.522
4-methylguaiacol sulfate	1.261	0.232	0.164	0.159	0.474
4-vinylguaiacol sulfate	1.119	0.112	0.172	0.513	0.774
4-vinylphenol sulfate	1.362	0.309	0.156	0.048	0.285
5-(galactosylhydroxy)-L-lysine	1.019	0.018	0.159	0.908	0.971
5,6-dihydrothymine	1.284	0.250	0.143	0.082	0.355
5,6-dihydrouracil	1.070	0.067	0.154	0.660	0.871
5,6-dihydrouridine	0.939	-0.063	0.167	0.706	0.888
5-acetylamino-6-formylamino-3-methyluracil	0.594	-0.522	0.231	0.024	0.195
5alpha-androstan-3alpha,17beta-diol monosulfate (1)	1.479	0.391	0.201	0.052	0.287

5alpha-androstan-3alpha,17beta-diol monosulfate (2)	1.415	0.347	0.246	0.159	0.474
5alpha-androstan-3beta,17alpha-diol disulfate	1.045	0.044	0.176	0.801	0.927
5alpha-androstan-3beta,17beta-diol disulfate	2.216	0.796	0.271	0.003	0.069
5alpha-androstan-3beta,17beta-diol monosulfate (2)	1.735	0.551	0.223	0.013	0.142
5alpha-pregnan-3beta,20alpha-diol disulfate	1.037	0.037	0.140	0.793	0.926
5alpha-pregnan-3beta,20alpha-diol monosulfate (2)	0.788	-0.239	0.158	0.131	0.431
5alpha-pregnan-3beta,20beta-diol monosulfate (1)	0.993	-0.007	0.153	0.966	0.983
5alpha-pregnan-diol disulfate	0.789	-0.237	0.228	0.297	0.628
5-dodecenoate (12:1n7)	0.925	-0.078	0.134	0.558	0.810
5-dodecenoylcarnitine (C12:1)	1.128	0.120	0.139	0.387	0.693
5-HEPE	2.225	0.800	0.435	0.066	0.323
5-hydroxyhexanoate	0.966	-0.035	0.122	0.775	0.923
5-hydroxylysine	1.016	0.015	0.149	0.918	0.973
5-methylthioadenosine (MTA)	1.130	0.122	0.171	0.477	0.760
5-methyluridine (ribothymidine)	0.877	-0.131	0.137	0.338	0.664
5-oxoproline	0.897	-0.109	0.133	0.412	0.718
6-bromotryptophan	1.420	0.350	0.184	0.056	0.296
6-hydroxyindole sulfate	0.698	-0.359	0.131	0.006	0.097
6-oxopiperidine-2-carboxylate	0.833	-0.182	0.134	0.172	0.492
7-HOCA	1.302	0.264	0.160	0.100	0.389
7-methylguanine	1.322	0.279	0.157	0.075	0.343
8-hydroxyoctanoate	0.925	-0.078	0.174	0.656	0.868
9,10-DiHOME	0.978	-0.022	0.161	0.890	0.970
9-hydroxystearate	1.076	0.073	0.152	0.629	0.853
acesulfame	0.787	-0.239	0.264	0.366	0.678
acetylcarnitine (C2)	1.133	0.125	0.139	0.369	0.678
acisoga	1.126	0.119	0.150	0.429	0.731
aconitate [cis or trans]	1.039	0.038	0.148	0.798	0.926
adenine	1.141	0.132	0.145	0.363	0.678
adipoylcarnitine (C6-DC)	1.450	0.372	0.197	0.059	0.303
adrenate (22:4n6)	1.124	0.117	0.123	0.344	0.671
adrenoylcarnitine (C22:4)*	1.026	0.026	0.184	0.890	0.970
alanine	1.242	0.217	0.145	0.134	0.435

allantoin	1.178	0.164	0.142	0.249	0.577
alliin	1.338	0.291	0.167	0.081	0.353
alpha-hydroxycaproate	1.449	0.371	0.151	0.014	0.145
alpha-hydroxyisocaproate	1.407	0.342	0.138	0.014	0.142
alpha-hydroxyisovalerate	1.366	0.312	0.148	0.035	0.242
alpha-ketobutyrate	1.318	0.276	0.155	0.074	0.342
alpha-ketoglutaramate*	0.833	-0.183	0.181	0.313	0.647
alpha-ketoglutarate	1.098	0.093	0.173	0.590	0.824
alpha-tocopherol	1.423	0.353	0.130	0.007	0.097
AMP	1.108	0.103	0.152	0.498	0.766
andro steroid monosulfate C19H28O6S (1)*	1.587	0.462	0.175	0.008	0.109
androstenediol (3alpha, 17alpha) monosulfate (2)	1.076	0.074	0.227	0.746	0.912
androstenediol (3alpha, 17alpha) monosulfate (3)	1.049	0.048	0.243	0.845	0.952
androstenediol (3beta,17beta) disulfate (1)	1.989	0.688	0.224	0.002	0.053
androstenediol (3beta,17beta) disulfate (2)	1.379	0.322	0.256	0.209	0.536
androstenediol (3beta,17beta) monosulfate (1)	1.345	0.297	0.187	0.113	0.409
androsterone glucuronide	1.285	0.251	0.206	0.223	0.547
androsterone sulfate	1.156	0.145	0.214	0.497	0.766
arabinose	1.127	0.120	0.154	0.437	0.732
arabitol/xylitol	0.914	-0.090	0.136	0.509	0.771
arabonate/xylonate	1.018	0.018	0.147	0.903	0.970
arachidate (20:0)	1.133	0.125	0.150	0.405	0.711
arachidonate (20:4n6)	0.941	-0.061	0.141	0.666	0.871
arachidonoylcarnitine (C20:4)	0.991	-0.010	0.175	0.957	0.980
arachidonoylcholine	1.040	0.039	0.144	0.784	0.924
arachidoylcarnitine (C20)*	1.269	0.238	0.177	0.179	0.500
argininate*	1.251	0.224	0.144	0.119	0.420
arginine	1.112	0.106	0.144	0.461	0.750
asparagine	0.875	-0.134	0.111	0.228	0.551
aspartate	1.402	0.338	0.145	0.019	0.167
azelate (nonanedioate; C9)	1.203	0.185	0.152	0.222	0.547
behenoyl sphingomyelin (d18:1/22:0)*	1.680	0.519	0.213	0.015	0.150
behenoylcarnitine (C22)*	1.397	0.334	0.159	0.035	0.243

benzoate	1.055	0.053	0.122	0.662	0.871
beta-alanine	1.234	0.210	0.165	0.201	0.530
beta-citrylglutamate	1.533	0.427	0.144	0.003	0.065
beta-cryptoxanthin	1.356	0.305	0.137	0.026	0.207
beta-hydroxyisovalerate	1.286	0.252	0.134	0.060	0.303
betaine	0.729	-0.315	0.154	0.040	0.262
beta-sitosterol	0.844	-0.170	0.166	0.308	0.638
bilirubin	1.101	0.096	0.142	0.501	0.768
bilirubin (E,E)*	0.866	-0.144	0.146	0.324	0.654
bilirubin (E,Z or Z,E)*	1.070	0.068	0.142	0.632	0.855
biliverdin	0.896	-0.110	0.157	0.486	0.763
bradykinin	14.807	2.695	3.126	0.389	0.693
bradykinin, des-arg(9)	1.194	0.177	0.487	0.716	0.892
caffeic acid sulfate	0.870	-0.139	0.155	0.372	0.678
caffeine	0.892	-0.115	0.162	0.478	0.760
campesterol	0.982	-0.019	0.156	0.905	0.970
caprate (10:0)	1.083	0.080	0.135	0.553	0.807
caproate (6:0)	1.113	0.107	0.159	0.502	0.768
caprylate (8:0)	1.176	0.162	0.126	0.196	0.526
carboxyethyl-GABA	0.827	-0.190	0.135	0.158	0.474
carnitine	1.481	0.392	0.155	0.011	0.134
carotene diol (1)	1.472	0.387	0.154	0.012	0.140
carotene diol (2)	1.197	0.180	0.147	0.220	0.547
carotene diol (3)	1.195	0.178	0.201	0.374	0.678
catechol sulfate	0.869	-0.140	0.140	0.315	0.649
ceramide (d16:1/24:1, d18:1/22:1)*	1.338	0.291	0.266	0.273	0.600
ceramide (d18:1/17:0, d17:1/18:0)*	1.178	0.164	0.146	0.263	0.590
ceramide (d18:1/20:0, d16:1/22:0, d20:1/18:0)*	1.519	0.418	0.150	0.005	0.092
ceramide (d18:2/24:1, d18:1/24:2)*	1.300	0.263	0.185	0.157	0.474
cerotoylcarnitine (C26)*	1.869	0.625	0.205	0.002	0.057
C-glycosyltryptophan	1.142	0.133	0.173	0.443	0.733
chenodeoxycholate	1.063	0.061	0.166	0.715	0.892
cholate	1.178	0.164	0.151	0.276	0.603

cholesterol	1.426	0.355	0.137	0.010	0.120
choline	0.911	-0.093	0.153	0.542	0.798
cinnamoylglycine	0.686	-0.378	0.147	0.010	0.124
cis-4-decenoate (10:1n6)*	1.063	0.061	0.141	0.666	0.871
cis-4-decenoylcarnitine (C10:1)	1.122	0.115	0.146	0.431	0.731
citramalate	1.070	0.068	0.203	0.739	0.910
citrate	0.808	-0.213	0.130	0.103	0.393
citrulline	0.948	-0.054	0.120	0.655	0.868
corticosterone	0.908	-0.097	0.152	0.524	0.784
cortisol	0.873	-0.136	0.121	0.259	0.585
cortisone	1.085	0.082	0.125	0.513	0.774
cotinine	1.073	0.070	0.267	0.792	0.926
creatine	1.234	0.210	0.165	0.203	0.532
creatinine	0.661	-0.415	0.203	0.041	0.264
cys-gly, oxidized	0.854	-0.158	0.148	0.285	0.617
cystathionine	1.188	0.172	0.166	0.301	0.631
cysteine	1.398	0.335	0.135	0.013	0.141
cysteine s-sulfate	1.126	0.119	0.134	0.373	0.678
cysteine sulfinic acid	1.220	0.199	0.132	0.130	0.431
cysteinylglycine	0.914	-0.090	0.165	0.588	0.824
cysteinylglycine disulfide*	0.946	-0.055	0.151	0.714	0.892
cystine	1.304	0.266	0.183	0.147	0.463
cytidine	1.140	0.131	0.128	0.306	0.635
daidzein sulfate (2)	1.190	0.174	0.154	0.259	0.585
decanoylcarnitine (C10)	1.111	0.105	0.129	0.414	0.718
dehydroisoandrosterone sulfate (DHEA-S)	1.082	0.079	0.204	0.699	0.887
delta-CEHC glucuronide*	0.920	-0.083	0.260	0.749	0.912
delta-CEHC*	0.985	-0.015	0.192	0.938	0.975
delta-tocopherol	1.084	0.081	0.307	0.792	0.926
deoxycarnitine	0.607	-0.499	0.199	0.012	0.140
deoxycholate	0.816	-0.203	0.159	0.201	0.530
diacylglycerol (14:0/18:1, 16:0/16:1) [1]*	1.515	0.416	0.177	0.019	0.167
diacylglycerol (16:1/18:2 [2], 16:0/18:3 [1])*	1.464	0.381	0.177	0.031	0.231

dihomolinoleate (20:2n6)	1.005	0.005	0.119	0.968	0.983
dihomolinolenate (20:3n3 or 3n6)	1.166	0.153	0.122	0.210	0.537
dihomo-linolenoylcarnitine (C20:3n3 or 6)*	1.254	0.226	0.176	0.199	0.529
dihomo-linolenoyl-choline	1.273	0.241	0.160	0.131	0.431
dihomo-linoleoylcarnitine (C20:2)*	1.013	0.013	0.165	0.939	0.975
dihydrocaffeate sulfate (2)	1.028	0.027	0.124	0.826	0.939
dihydroferulate	1.242	0.217	0.188	0.250	0.577
dihydroferulic acid sulfate	1.131	0.123	0.174	0.480	0.760
dihydroorotate	0.880	-0.128	0.164	0.435	0.731
dimethylarginine (ADMA + SDMA)	1.095	0.091	0.171	0.596	0.827
dimethylglycine	0.893	-0.113	0.146	0.437	0.732
docosadienoate (22:2n6)	1.227	0.204	0.178	0.252	0.577
docosadioate (C22-DC)	1.376	0.320	0.175	0.068	0.325
docosahexaenoate (DHA; 22:6n3)	0.973	-0.028	0.155	0.857	0.959
docosahexaenoylcarnitine (C22:6)*	1.123	0.116	0.151	0.441	0.733
docosahexaenoylcholine	1.024	0.024	0.147	0.871	0.966
docosapentaenoate (DPA; 22:5n3)	1.122	0.115	0.142	0.418	0.718
docosapentaenoate (n6 DPA; 22:5n6)	0.940	-0.061	0.131	0.639	0.861
docosapentaenoylcarnitine (C22:5n3)*	1.224	0.202	0.186	0.278	0.605
docosatrienoate (22:3n3)	0.979	-0.021	0.137	0.880	0.970
docosatrienoate (22:3n6)*	0.830	-0.187	0.169	0.269	0.597
dodecadienoate (12:2)*	1.109	0.104	0.138	0.451	0.740
dodecanedioate (C12)	0.952	-0.049	0.134	0.715	0.892
dodecenedioate (C12:1-DC)*	0.872	-0.137	0.132	0.300	0.629
dopamine 3-O-sulfate	1.020	0.020	0.141	0.889	0.970
DSGEGDFXAEGGGVR*	0.260	-1.346	0.561	0.016	0.156
ectoine	0.896	-0.110	0.154	0.476	0.760
EDTA	1.147	0.137	0.200	0.494	0.766
eicosanedioate (C20-DC)	1.208	0.189	0.150	0.208	0.536
eicosapentaenoate (EPA; 20:5n3)	1.165	0.152	0.153	0.319	0.649
eicosenamide (20:1)*	0.884	-0.123	0.135	0.361	0.678
eicosenedioate (C20:1-DC)*	0.931	-0.071	0.201	0.724	0.900
eicosenoate (20:1n9 or 1n11)	1.073	0.070	0.185	0.706	0.888

eicosenoylcarnitine (C20:1)*	1.100	0.095	0.240	0.692	0.884
epiandrosterone sulfate	0.979	-0.021	0.203	0.918	0.973
equol sulfate	0.764	-0.269	0.289	0.353	0.677
ergothioneine	1.056	0.054	0.160	0.734	0.909
erucate (22:1n9)	1.297	0.260	0.294	0.376	0.681
erucoylcarnitine (C22:1)*	1.340	0.293	0.257	0.254	0.578
erythritol	0.916	-0.088	0.139	0.525	0.784
erythronate*	1.178	0.164	0.154	0.288	0.620
ethylmalonate	1.292	0.256	0.190	0.178	0.498
etiocholanolone glucuronide	0.740	-0.301	0.196	0.124	0.423
eugenol sulfate	0.872	-0.137	0.179	0.442	0.733
FAD	1.161	0.149	0.193	0.441	0.733
ferulic acid 4-sulfate	1.191	0.175	0.179	0.328	0.659
formiminoglutamate	1.652	0.502	0.175	0.004	0.079
fructose	1.156	0.145	0.160	0.364	0.678
fumarate	0.864	-0.146	0.133	0.271	0.598
galactonate	0.942	-0.060	0.179	0.739	0.910
gamma-carboxyglutamate	1.083	0.079	0.151	0.601	0.832
gamma-CEHC	1.107	0.101	0.160	0.526	0.784
gamma-CEHC glucuronide*	1.087	0.083	0.170	0.626	0.852
gamma-glutamyl-2-aminobutyrate	0.994	-0.006	0.156	0.972	0.983
gamma-glutamylalanine	0.988	-0.012	0.155	0.939	0.975
gamma-glutamyl-alpha-lysine	1.065	0.063	0.138	0.649	0.865
gamma-glutamylcitrulline*	0.705	-0.350	0.140	0.013	0.141
gamma-glutamyl-epsilon-lysine	0.780	-0.249	0.165	0.131	0.431
gamma-glutamylglutamate	1.409	0.343	0.142	0.016	0.155
gamma-glutamylglutamine	0.698	-0.359	0.130	0.006	0.092
gamma-glutamylglycine	0.786	-0.241	0.135	0.075	0.343
gamma-glutamylhistidine	0.989	-0.011	0.154	0.944	0.975
gamma-glutamylisoleucine*	1.254	0.227	0.124	0.067	0.323
gamma-glutamylleucine	1.277	0.245	0.126	0.053	0.288
gamma-glutamylmethionine	0.856	-0.155	0.150	0.301	0.631
gamma-glutamylphenylalanine	1.252	0.225	0.138	0.103	0.393

gamma-glutamylthreonine	0.922	-0.081	0.148	0.583	0.824
gamma-glutamyltryptophan	1.076	0.073	0.137	0.595	0.827
gamma-glutamyltyrosine	1.189	0.173	0.140	0.215	0.540
gamma-glutamylvaline	1.293	0.257	0.154	0.095	0.381
gamma-tocopherol/beta-tocopherol	1.505	0.409	0.182	0.025	0.201
genistein sulfate*	1.273	0.241	0.158	0.128	0.430
gentisate	1.066	0.064	0.153	0.677	0.878
gluconate	0.945	-0.057	0.142	0.687	0.882
glucose	1.161	0.149	0.136	0.271	0.598
glucuronate	1.026	0.025	0.184	0.890	0.970
glucuronide of C10H18O2 (7)*	1.570	0.450	0.260	0.000	0.052
glutamine	0.919	-0.085	0.122	0.486	0.763
glutarate (C5-DC)	0.920	-0.084	0.148	0.571	0.819
glutaroylcarnitine (C5)	1.024	0.023	0.163	0.886	0.970
glycerate	1.383	0.324	0.153	0.034	0.242
glycerol	1.105	0.100	0.132	0.451	0.740
glycerol 3-phosphate	1.581	0.458	0.195	0.019	0.166
glycerophosphoethanolamine	1.220	0.199	0.144	0.167	0.482
glycerophosphoglycerol	1.157	0.146	0.157	0.351	0.677
glycerophosphorylcholine (GPC)	1.050	0.049	0.160	0.760	0.912
glycine	0.743	-0.298	0.148	0.044	0.270
glycine conjugate of C10H14O2 (1)*	0.981	-0.019	0.185	0.919	0.973
glyco-alpha-muricholate	0.789	-0.237	0.161	0.141	0.449
glyco-beta-muricholate	0.710	-0.342	0.163	0.036	0.244
glycochenodeoxycholate	1.015	0.015	0.141	0.917	0.973
glycochenodeoxycholate 3-sulfate	1.241	0.216	0.149	0.146	0.461
glycochenodeoxycholate glucuronide (1)	1.176	0.162	0.152	0.287	0.620
glycocholate	1.098	0.094	0.142	0.509	0.771
glycocholenate sulfate*	1.039	0.038	0.138	0.780	0.924
glycodeoxycholate	0.817	-0.203	0.155	0.192	0.523
glycodeoxycholate 3-sulfate	0.889	-0.117	0.149	0.432	0.731
glycohyocholate	0.895	-0.111	0.127	0.384	0.691
glycolithocholate	1.115	0.109	0.165	0.508	0.771

glycolithocholate sulfate*	0.792	-0.233	0.143	0.103	0.394
glycosyl ceramide (d18:1/20:0, d16:1/22:0)*	0.957	-0.044	0.156	0.778	0.924
glycosyl ceramide (d18:1/23:1, d17:1/24:1)*	0.749	-0.289	0.229	0.207	0.535
glycosyl ceramide (d18:2/24:1, d18:1/24:2)*	0.687	-0.376	0.176	0.033	0.241
glycosyl-N-(2-hydroxynervonoyl)-sphingosine (d18:1/24:1(2OH))*	1.652	0.502	0.205	0.014	0.145
glycosyl-N-behenoyl-sphingadienine (d18:2/22:0)*	0.637	-0.452	0.166	0.006	0.097
glycosyl-N-behenoyl-sphingosine (d18:1/22:0)*	1.058	0.056	0.147	0.703	0.888
glycosyl-N-nervonoyl-sphingosine (d18:1/24:1)*	0.616	-0.485	0.245	0.047	0.284
glycosyl-N-palmitoyl-sphingosine (d18:1/16:0)	0.888	-0.119	0.137	0.383	0.690
glycosyl-N-stearoyl-sphingosine (d18:1/18:0)	0.946	-0.055	0.143	0.698	0.887
glycosyl-N-tricosanoyl-sphingadienine (d18:2/23:0)*	0.801	-0.222	0.159	0.163	0.480
glycoursodeoxycholate	1.012	0.011	0.139	0.934	0.975
glycoursodeoxycholate glucuronide (2)	1.072	0.069	0.299	0.816	0.935
glycyrrhetinate	0.877	-0.131	0.259	0.613	0.842
guaiacol sulfate	0.898	-0.107	0.161	0.505	0.770
guanidinoacetate	1.013	0.013	0.180	0.941	0.975
guanidinosuccinate	0.833	-0.182	0.249	0.464	0.752
guanosine	0.770	-0.262	0.215	0.223	0.547
gulonate*	1.188	0.172	0.162	0.289	0.622
heme	0.838	-0.177	0.155	0.253	0.577
heneicosapentaenoate (21:5n3)	1.394	0.332	0.217	0.125	0.426
heptadecenamide (17:1)*	1.019	0.019	0.150	0.898	0.970
heptenedioate (C7:1-DC)*	0.879	-0.129	0.183	0.481	0.760
hexadecadienoate (16:2n6)	1.059	0.058	0.147	0.695	0.886
hexadecanedioate (C16)	1.012	0.012	0.157	0.938	0.975
hexadecenedioate (C16:1-DC)*	0.928	-0.075	0.177	0.672	0.876
hexanoylcarnitine (C6)	1.235	0.211	0.133	0.113	0.409
hexanoylglutamine	0.952	-0.049	0.156	0.754	0.912
hexanoylglycine (C6)	0.855	-0.156	0.147	0.286	0.619
hippurate	0.899	-0.107	0.137	0.434	0.731
histidine	0.850	-0.163	0.131	0.215	0.540
histidine betaine (hercynine)*	1.022	0.022	0.152	0.885	0.970
homoarginine	1.220	0.199	0.133	0.134	0.435

homocitrulline	0.843	-0.170	0.153	0.265	0.591
homostachydrine*	1.100	0.095	0.172	0.579	0.822
homovanillate (HVA)	1.010	0.009	0.158	0.952	0.977
HWESASXX*	1.245	0.219	0.198	0.270	0.597
hydantoin-5-propionate	0.903	-0.102	0.145	0.480	0.760
hydroquinone sulfate	1.014	0.014	0.170	0.936	0.975
hydroxyasparagine	1.344	0.296	0.175	0.092	0.376
hydroxy-CMPF*	0.645	-0.438	0.245	0.074	0.342
hydroxycotinine	1.500	0.405	0.239	0.090	0.376
hydroxy-N6,N6,N6-trimethyllysine*	1.016	0.016	0.160	0.922	0.973
hydroxypalmitoyl sphingomyelin (d18:1/16:0(OH))	1.107	0.102	0.148	0.489	0.765
hydroxyproline	1.118	0.111	0.155	0.474	0.760
hyocholate	0.965	-0.036	0.129	0.783	0.924
hypotaurine	0.592	-0.524	0.180	0.004	0.073
hypoxanthine	1.064	0.062	0.150	0.678	0.879
imidazole lactate	0.872	-0.137	0.162	0.399	0.704
imidazole propionate	0.936	-0.066	0.111	0.553	0.807
iminodiacetate (IDA)	1.234	0.210	0.152	0.168	0.482
indole-3-carboxylate	1.427	0.356	0.170	0.037	0.246
indoleacetate	1.132	0.124	0.153	0.419	0.718
indoleacetylcarnitine*	1.251	0.224	0.183	0.222	0.547
indoleacetylglutamine	1.305	0.266	0.174	0.127	0.427
indolelactate	1.039	0.039	0.153	0.800	0.927
indolepropionate	0.872	-0.138	0.138	0.319	0.649
indolin-2-one	0.675	-0.393	0.180	0.029	0.223
inosine	1.035	0.034	0.149	0.820	0.936
isobutyrylcarnitine (C4)	0.659	-0.417	0.158	0.008	0.109
isobutyrylglycine (C4)	1.063	0.061	0.160	0.704	0.888
isoeugenol sulfate	1.462	0.380	0.236	0.108	0.400
isoleucine	1.254	0.227	0.128	0.078	0.348
isoursodeoxycholate	0.902	-0.103	0.154	0.506	0.771
isovalerate (C5)	1.228	0.205	0.152	0.176	0.497
isovalerylcarnitine (C5)	1.259	0.230	0.136	0.090	0.376

isovalerylglycine	0.827	-0.190	0.162	0.242	0.565
I-urobilinogen	1.081	0.078	0.236	0.740	0.910
kynurenate	1.096	0.092	0.139	0.510	0.771
kynurenine	1.105	0.100	0.136	0.466	0.754
lactate	1.082	0.079	0.140	0.574	0.820
lactosyl-N-behenoyl-sphingosine (d18:1/22:0)*	1.114	0.108	0.172	0.529	0.786
lactosyl-N-nervonoyl-sphingosine (d18:1/24:1)*	0.693	-0.367	0.189	0.052	0.287
lactosyl-N-palmitoyl-sphingosine (d18:1/16:0)	0.679	-0.386	0.163	0.018	0.166
lanthionine	0.800	-0.224	0.148	0.131	0.431
laurate (12:0)	1.059	0.057	0.143	0.688	0.882
laurylcarnitine (C12)	1.114	0.108	0.132	0.415	0.718
leucine	1.307	0.268	0.130	0.039	0.256
leukotriene B4	0.853	-0.159	0.166	0.337	0.664
lignoceroyl sphingomyelin (d18:1/24:0)	1.319	0.277	0.192	0.149	0.467
lignoceroylcarnitine (C24)*	1.723	0.544	0.180	0.003	0.059
linoleate (18:2n6)	1.030	0.029	0.138	0.834	0.945
linolenate (18:3n3 or 3n6)	1.088	0.084	0.151	0.579	0.822
linolenoylcarnitine (C18:3)*	1.182	0.167	0.180	0.354	0.677
linoleoyl ethanolamide	0.987	-0.013	0.175	0.943	0.975
linoleoyl-arachidonoyl-glycerol (18:2/20:4) [1]*	1.331	0.286	0.159	0.071	0.338
linoleoyl-arachidonoyl-glycerol (18:2/20:4) [2]*	1.278	0.245	0.162	0.131	0.431
linoleoylcarnitine (C18:2)*	1.033	0.032	0.174	0.853	0.957
linoleoylcholine*	1.201	0.183	0.150	0.221	0.547
linoleoyl-docosahexaenoyl-glycerol (18:2/22:6) [2]*	1.146	0.136	0.229	0.551	0.806
linoleoyl-linolenoyl-glycerol (18:2/18:3) [2]*	1.370	0.315	0.180	0.080	0.353
linoleoyl-linoleoyl-glycerol (18:2/18:2) [1]*	1.194	0.177	0.180	0.323	0.653
lithocholate sulfate (1)	1.057	0.055	0.175	0.753	0.912
L-urobilin	1.164	0.152	0.201	0.451	0.740
lysine	1.086	0.083	0.135	0.540	0.796
malate	0.720	-0.328	0.162	0.043	0.269
maleate	1.227	0.205	0.138	0.138	0.443
mannitol/sorbitol	1.014	0.014	0.126	0.912	0.973
mannonate*	1.225	0.203	0.130	0.118	0.420

mannose	1.239	0.215	0.139	0.122	0.420
margarate (17:0)	1.016	0.016	0.121	0.896	0.970
margaroylcarnitine (C17)*	1.148	0.138	0.138	0.318	0.649
mead acid (20:3n9)	1.047	0.046	0.165	0.780	0.924
methionine	1.010	0.010	0.150	0.948	0.975
methionine sulfone	1.155	0.145	0.126	0.251	0.577
methionine sulfoxide	1.273	0.242	0.152	0.112	0.409
methyl glucopyranoside (alpha + beta)	1.082	0.079	0.148	0.593	0.826
methyl indole-3-acetate	1.355	0.304	0.162	0.061	0.306
methyl-4-hydroxybenzoate sulfate	0.958	-0.043	0.166	0.794	0.926
methylnaphthyl sulfate (2)*	1.232	0.209	0.209	0.318	0.649
methylsuccinate	0.847	-0.166	0.137	0.226	0.550
myo-inositol	1.002	0.002	0.133	0.988	0.992
myristate (14:0)	1.124	0.117	0.137	0.394	0.698
myristoleate (14:1n5)	0.995	-0.005	0.136	0.968	0.983
myristoleoylcarnitine (C14:1)*	1.061	0.059	0.138	0.666	0.871
myristoyl dihydrosphingomyelin (d18:0/14:0)*	1.552	0.439	0.142	0.002	0.052
myristoylcarnitine (C14)	1.188	0.172	0.134	0.200	0.530
myristoyl-linoleoyl-glycerol (14:0/18:2) [1]*	1.631	0.489	0.162	0.003	0.059
N-(2-furoyl)glycine	0.870	-0.139	0.194	0.473	0.760
N,N,N-trimethyl-5-aminovalerate	0.927	-0.075	0.201	0.707	0.888
N,N,N-trimethyl-alanylproline betaine (TMAP)	0.836	-0.179	0.195	0.359	0.678
N1-Methyl-2-pyridone-5-carboxamide	1.270	0.239	0.170	0.158	0.474
N1-methylinosine	0.914	-0.090	0.153	0.558	0.810
N2,N2-dimethylguanosine	1.062	0.060	0.155	0.696	0.886
N2,N5-diacetylornithine	1.008	0.008	0.190	0.968	0.983
N2-acetyl,N6-methyllysine	0.678	-0.389	0.197	0.049	0.286
N4-acetylcytidine	1.105	0.100	0.140	0.475	0.760
N6,N6,N6-trimethyllysine	1.072	0.069	0.154	0.652	0.867
N6,N6-dimethyllysine	0.791	-0.234	0.138	0.091	0.376
N6-acetyllysine	0.982	-0.018	0.144	0.902	0.970
N6-carbamoylthreonyladenosine	1.295	0.258	0.156	0.098	0.386
N6-methyllysine	0.798	-0.225	0.148	0.126	0.427

N-acetyl-1-methylhistidine*	1.388	0.328	0.200	0.102	0.391
N-acetyl-2-aminooctanoate*	1.219	0.198	0.147	0.179	0.500
N-acetylalanine	1.011	0.011	0.138	0.938	0.975
N-acetylalliin	1.406	0.340	0.154	0.027	0.212
N-acetylarginine	1.045	0.044	0.143	0.758	0.912
N-acetylaspartate (NAA)	0.994	-0.006	0.133	0.964	0.983
N-acetyl-aspartyl-glutamate (NAAG)	0.979	-0.021	0.148	0.886	0.970
N-acetyl-beta-alanine	1.221	0.200	0.161	0.216	0.541
N-acetylcarnosine	1.253	0.226	0.172	0.188	0.520
N-acetylcitrulline	0.685	-0.379	0.207	0.067	0.323
N-acetylglucosamine/N-acetylgalactosamine	1.181	0.166	0.157	0.291	0.624
N-acetylglucosaminylasparagine	0.848	-0.165	0.206	0.423	0.723
N-acetylglutamate	1.051	0.050	0.139	0.718	0.895
N-acetylglutamine	0.806	-0.216	0.182	0.236	0.554
N-acetylglycine	0.768	-0.264	0.152	0.082	0.358
N-acetylhistidine	0.939	-0.063	0.159	0.691	0.884
N-acetylisoleucine	1.229	0.206	0.130	0.114	0.409
N-acetyl-isoputreanine*	1.177	0.163	0.151	0.282	0.611
N-acetylleucine	1.191	0.174	0.119	0.142	0.451
N-acetylmethionine	0.668	-0.404	0.149	0.007	0.097
N-acetylneuraminate	0.963	-0.038	0.150	0.800	0.927
N-acetylphenylalanine	1.058	0.056	0.134	0.676	0.878
N-acetylproline	1.143	0.134	0.176	0.448	0.738
N-acetylputrescine	1.290	0.254	0.151	0.092	0.376
N-acetylserine	0.806	-0.216	0.194	0.266	0.591
N-acetyltaurine	0.803	-0.219	0.208	0.292	0.624
N-acetylthreonine	1.103	0.098	0.149	0.509	0.771
N-acetyltryptophan	1.067	0.065	0.128	0.611	0.842
N-acetyltyrosine	0.959	-0.042	0.127	0.740	0.910
N-acetylvaline	1.145	0.136	0.149	0.363	0.678
N-behenoyl-sphingadienine (d18:2/22:0)*	1.675	0.516	0.183	0.005	0.086
N-carbamoylvaline	0.903	-0.101	0.180	0.573	0.820
N-delta-acetylornithine	0.987	-0.013	0.139	0.926	0.975

nervonoylcarnitine (C24:1)*	1.607	0.474	0.256	0.064	0.318
N-formylanthranilic acid	0.839	-0.175	0.160	0.273	0.600
N-formylmethionine	1.047	0.046	0.171	0.788	0.926
N-formylphenylalanine	1.139	0.130	0.114	0.251	0.577
nicotinamide	1.282	0.248	0.153	0.106	0.398
N-linoleoylglycine	0.851	-0.162	0.161	0.316	0.649
N-linoleoylserine*	0.803	-0.219	0.168	0.192	0.523
N-linoleoyltaurine*	0.900	-0.106	0.214	0.622	0.850
N-methylhydroxyproline	1.261	0.232	0.193	0.229	0.552
N-methylpipecolate	0.763	-0.271	0.155	0.081	0.355
N-methylproline	1.140	0.131	0.144	0.363	0.678
N-oleoylserine	1.034	0.034	0.147	0.819	0.936
N-oleoyltaurine	1.175	0.162	0.163	0.322	0.652
nonadecanoate (19:0)	0.991	-0.009	0.148	0.949	0.975
nonanoylcarnitine (C9)	1.081	0.078	0.135	0.561	0.812
N-palmitoylglycine	1.092	0.088	0.128	0.490	0.765
N-palmitoyl-heptadecasphingosine (d17:1/16:0)*	0.940	-0.062	0.200	0.755	0.912
N-palmitoylserine	0.994	-0.006	0.171	0.971	0.983
N-palmitoyl-sphingadienine (d18:2/16:0)*	1.075	0.072	0.158	0.648	0.865
N-palmitoyl-sphinganine (d18:0/16:0)	1.320	0.278	0.133	0.037	0.250
N-palmitoyl-sphingosine (d18:1/16:0)	1.253	0.226	0.151	0.134	0.435
N-stearoylserine*	1.264	0.234	0.175	0.181	0.503
N-stearoyl-sphinganine (d18:0/18:0)*	1.551	0.439	0.142	0.002	0.052
N-stearoyl-sphingosine (d18:1/18:0)*	1.413	0.346	0.140	0.014	0.143
N-stearoyltaurine	1.253	0.226	0.159	0.157	0.474
O-acetylhomoserine	0.959	-0.042	0.121	0.728	0.903
o-cresol sulfate	1.176	0.162	0.179	0.367	0.678
octadecadienedioate (C18:2-DC)*	1.239	0.214	0.142	0.130	0.431
octadecanedioate (C18)	1.144	0.134	0.173	0.438	0.733
octadecanedioylcarnitine (C18-DC)*	1.295	0.258	0.193	0.181	0.503
octadecenedioate (C18:1-DC)*	0.994	-0.006	0.171	0.972	0.983
octadecenedioylcarnitine (C18:1-DC)*	1.182	0.167	0.206	0.418	0.718
octanoylcarnitine (C8)	1.126	0.119	0.129	0.357	0.678

oleate/vaccenate (18:1)	1.085	0.082	0.128	0.522	0.783
oleoyl ethanolamide	1.093	0.088	0.151	0.558	0.810
oleoyl-arachidonoyl-glycerol (18:1/20:4) [1]*	1.311	0.271	0.153	0.077	0.346
oleoyl-arachidonoyl-glycerol (18:1/20:4) [2]*	1.307	0.268	0.153	0.080	0.353
oleoylcarnitine (C18)	1.054	0.053	0.173	0.760	0.912
oleoylcholine	1.196	0.179	0.148	0.225	0.550
oleoyl-linolenoyl-glycerol (18:1/18:3) [2]*	1.226	0.204	0.163	0.212	0.538
oleoyl-linoleoyl-glycerol (18:1/18:2) [1]	1.240	0.215	0.146	0.140	0.447
oleoyl-linoleoyl-glycerol (18:1/18:2) [2]	1.244	0.218	0.144	0.129	0.431
oleoyl-oleoyl-glycerol (18:1/18:1) [1]*	1.402	0.338	0.184	0.067	0.323
oleoyl-oleoyl-glycerol (18:1/18:1) [2]*	1.472	0.386	0.201	0.055	0.289
ornithine	0.957	-0.044	0.120	0.714	0.892
orotate	0.816	-0.203	0.210	0.334	0.663
orotidine	1.361	0.308	0.190	0.105	0.396
O-sulfo-L-tyrosine	0.994	-0.006	0.150	0.967	0.983
oxalate (ethanedioate)	1.316	0.275	0.140	0.050	0.286
palmitate (16:0)	1.090	0.086	0.126	0.496	0.766
palmitoleamide (16:1)*	1.116	0.110	0.151	0.468	0.756
palmitoleate (16:1n7)	1.034	0.034	0.139	0.808	0.932
palmitoleoyl-arachidonoyl-glycerol (16:1/20:4) [2]*	1.578	0.456	0.192	0.017	0.162
palmitoleoylcarnitine (C16:1)*	1.044	0.043	0.149	0.774	0.923
palmitoleoyl-linoleoyl-glycerol (16:1/18:2) [1]*	1.360	0.308	0.158	0.052	0.287
palmitoyl dihydrosphingomyelin (d18:0/16:0)*	0.983	-0.017	0.136	0.899	0.970
palmitoyl sphingomyelin (d18:1/16:0)	0.920	-0.083	0.139	0.551	0.806
palmitoyl-arachidonoyl-glycerol (16:0/20:4) [1]*	1.123	0.116	0.169	0.494	0.766
palmitoyl-arachidonoyl-glycerol (16:0/20:4) [2]*	1.361	0.308	0.146	0.034	0.242
palmitoylcarnitine (C16)	1.444	0.367	0.151	0.015	0.153
palmitoylcholine	1.169	0.156	0.144	0.279	0.607
palmitoyl-linoleoyl-glycerol (16:0/18:2) [1]*	1.509	0.412	0.157	0.009	0.111
palmitoyl-linoleoyl-glycerol (16:0/18:2) [2]*	1.559	0.444	0.157	0.005	0.085
palmitoyl-oleoyl-glycerol (16:0/18:1) [1]*	1.505	0.409	0.165	0.013	0.141
palmitoyl-oleoyl-glycerol (16:0/18:1) [2]*	1.649	0.500	0.159	0.002	0.051
pantothenate (Vitamin B5)	1.067	0.065	0.134	0.629	0.853

paraxanthine	0.930	-0.073	0.182	0.690	0.884
p-cresol glucuronide*	0.713	-0.339	0.166	0.041	0.264
p-cresol sulfate	0.689	-0.373	0.142	0.009	0.111
pentadecanoate (15:0)	1.007	0.007	0.147	0.964	0.983
perfluorooctanesulfonate (PFOS)	1.137	0.128	0.260	0.622	0.850
perfluorooctanoate (PFOA)*	1.062	0.060	0.288	0.835	0.945
phenol glucuronide	0.893	-0.113	0.179	0.527	0.785
phenol sulfate	0.881	-0.126	0.141	0.371	0.678
phenylacetate	0.754	-0.282	0.180	0.116	0.416
phenylacetylcarnitine	0.785	-0.242	0.147	0.100	0.389
phenylacetylglutamate	0.809	-0.212	0.217	0.330	0.659
phenylalanine	1.049	0.048	0.136	0.727	0.902
phenyllactate (PLA)	1.070	0.068	0.163	0.677	0.878
phenylpyruvate	1.084	0.080	0.143	0.574	0.820
phosphate	1.067	0.065	0.125	0.602	0.832
phosphocholine	1.555	0.442	0.157	0.005	0.089
phosphoethanolamine (PE)	1.532	0.426	0.147	0.004	0.074
picolinate	1.063	0.062	0.236	0.794	0.926
pimelate (C7-DC)	0.896	-0.110	0.173	0.525	0.784
pimeloylcarnitine/3-methyladipoylcarnitine (C7-DC)	1.050	0.049	0.197	0.804	0.929
pipecolate	0.919	-0.084	0.119	0.480	0.760
piperine	0.599	-0.513	0.170	0.002	0.059
pregnanediol-3-glucuronide	0.859	-0.152	0.154	0.321	0.652
pregnanolone/allopregnanolone sulfate	1.034	0.033	0.245	0.891	0.970
pregnen-diol disulfate*	1.325	0.281	0.181	0.120	0.420
pregnenediol sulfate (C21H34O5S)*	1.018	0.018	0.148	0.905	0.970
pregnenetriol disulfate*	1.458	0.377	0.194	0.052	0.287
pregnenetriol sulfate*	1.260	0.231	0.192	0.229	0.552
pregnenolone sulfate	0.883	-0.125	0.178	0.483	0.761
proline	1.106	0.101	0.139	0.470	0.758
prolylglycine	1.134	0.126	0.135	0.352	0.677
prolylhydroxyproline	0.954	-0.047	0.152	0.758	0.912
propionylcarnitine (C3)	1.254	0.227	0.143	0.113	0.409

propionylglycine (C3)	0.858	-0.153	0.145	0.291	0.624
propyl 4-hydroxybenzoate sulfate	0.730	-0.315	0.341	0.355	0.677
pseudouridine	0.997	-0.003	0.174	0.984	0.990
pyridoxal	1.079	0.076	0.154	0.623	0.850
pyridoxate	1.022	0.022	0.134	0.869	0.966
pyroglutamine*	0.876	-0.133	0.197	0.502	0.768
pyruvate	1.156	0.145	0.157	0.355	0.677
quinate	0.827	-0.190	0.141	0.176	0.497
quinolinate	1.194	0.177	0.149	0.232	0.553
retinol (Vitamin A)	1.304	0.266	0.171	0.120	0.420
ribitol	1.229	0.207	0.134	0.122	0.420
ribonate (ribonolactone)	1.136	0.127	0.142	0.368	0.678
ribulonate/xylulonate*	1.006	0.006	0.172	0.971	0.983
S-(3-hydroxypropyl)mercapturic acid (HPMA)	0.930	-0.072	0.221	0.744	0.912
S-1-pyrroline-5-carboxylate	1.070	0.068	0.126	0.589	0.824
saccharin	1.644	0.497	0.257	0.053	0.288
S-adenosylhomocysteine (SAH)	1.134	0.126	0.221	0.569	0.817
salicylate	0.890	-0.117	0.169	0.488	0.765
salicyluric glucuronide*	1.361	0.309	0.356	0.387	0.693
S-allylcysteine	1.385	0.326	0.185	0.079	0.352
sarcosine	0.892	-0.114	0.139	0.412	0.718
sebacate (C10-DC)	1.108	0.103	0.142	0.467	0.755
serine	0.769	-0.262	0.146	0.072	0.338
S-methylcysteine	1.131	0.123	0.142	0.388	0.693
S-methylcysteine sulfoxide	1.263	0.234	0.147	0.112	0.409
S-methylmethionine	1.054	0.053	0.161	0.742	0.911
spermidine	1.224	0.202	0.177	0.253	0.577
sphingadienine	1.359	0.307	0.152	0.044	0.270
sphinganine	1.232	0.208	0.131	0.112	0.409
sphinganine-1-phosphate	0.896	-0.110	0.114	0.337	0.664
sphingomyelin (d17:1/14:0, d16:1/15:0)*	1.151	0.141	0.180	0.435	0.731
sphingomyelin (d17:1/16:0, d18:1/15:0, d16:1/17:0)*	1.136	0.128	0.167	0.444	0.734
sphingomyelin (d17:2/16:0, d18:2/15:0)*	1.377	0.320	0.164	0.051	0.287

sphingomyelin (d18:0/18:0, d19:0/17:0)*	1.594	0.466	0.149	0.002	0.051
sphingomyelin (d18:1/14:0, d16:1/16:0)*	1.500	0.405	0.149	0.007	0.097
sphingomyelin (d18:1/17:0, d17:1/18:0, d19:1/16:0)	0.860	-0.151	0.175	0.388	0.693
sphingomyelin (d18:1/18:1, d18:2/18:0)	1.008	0.008	0.130	0.949	0.975
sphingomyelin (d18:1/19:0, d19:1/18:0)*	1.074	0.072	0.141	0.612	0.842
sphingomyelin (d18:1/20:0, d16:1/22:0)*	1.055	0.053	0.143	0.708	0.889
sphingomyelin (d18:1/20:1, d18:2/20:0)*	0.656	-0.421	0.156	0.007	0.101
sphingomyelin (d18:1/20:2, d18:2/20:1, d16:1/22:2)*	0.816	-0.203	0.164	0.214	0.540
sphingomyelin (d18:1/21:0, d17:1/22:0, d16:1/23:0)*	1.593	0.466	0.204	0.023	0.188
sphingomyelin (d18:1/22:1, d18:2/22:0, d16:1/24:1)*	0.964	-0.036	0.182	0.842	0.949
sphingomyelin (d18:1/22:2, d18:2/22:1, d16:1/24:2)*	0.766	-0.267	0.190	0.161	0.478
sphingomyelin (d18:1/24:1, d18:2/24:0)*	1.028	0.028	0.224	0.901	0.970
sphingomyelin (d18:1/25:0, d19:0/24:1, d20:1/23:0, d19:1/24:0)*	1.483	0.394	0.205	0.054	0.289
sphingomyelin (d18:2/14:0, d18:1/14:1)*	1.484	0.394	0.176	0.025	0.201
sphingomyelin (d18:2/16:0, d18:1/16:1)*	1.065	0.063	0.141	0.657	0.870
sphingomyelin (d18:2/18:1)*	0.670	-0.401	0.160	0.012	0.140
sphingomyelin (d18:2/21:0, d16:2/23:0)*	0.987	-0.013	0.175	0.942	0.975
sphingomyelin (d18:2/23:0, d18:1/23:1, d17:1/24:1)*	1.096	0.092	0.169	0.587	0.824
sphingomyelin (d18:2/23:1)*	0.988	-0.012	0.212	0.956	0.980
sphingomyelin (d18:2/24:1, d18:1/24:2)*	0.777	-0.252	0.156	0.106	0.398
sphingomyelin (d18:2/24:2)*	0.781	-0.247	0.243	0.310	0.642
sphingosine	1.140	0.131	0.139	0.347	0.675
sphingosine 1-phosphate	0.901	-0.105	0.131	0.425	0.726
stachydrine	1.202	0.184	0.127	0.149	0.467
stearamide (18:0)	0.851	-0.161	0.133	0.228	0.551
stearate (18:0)	0.982	-0.019	0.130	0.887	0.970
stearidonate (18:4n3)	1.319	0.277	0.166	0.095	0.381
stearoyl sphingomyelin (d18:1/18:0)	0.918	-0.086	0.143	0.549	0.805
stearoyl-arachidonoyl-glycerol (18:0/20:4) [1]*	0.945	-0.057	0.150	0.706	0.888
stearoyl-arachidonoyl-glycerol (18:0/20:4) [2]*	1.117	0.110	0.135	0.414	0.718
stearoylcarnitine (C18)	1.279	0.246	0.190	0.195	0.524
stearoylcholine*	1.254	0.227	0.138	0.100	0.391
suberate (C8-DC)	1.088	0.084	0.136	0.535	0.793

suberoylcarnitine (C8-DC)	1.120	0.113	0.176	0.521	0.782
succinate	0.887	-0.120	0.135	0.373	0.678
succinimide	0.894	-0.112	0.171	0.515	0.776
succinylcarnitine (C4)	0.968	-0.033	0.138	0.811	0.933
sucrose	1.053	0.051	0.169	0.761	0.912
sulfate*	1.022	0.021	0.162	0.895	0.970
syringol sulfate	0.784	-0.243	0.291	0.405	0.711
tartarate	1.058	0.057	0.129	0.661	0.871
tartronate (hydroxymalonate)	1.226	0.204	0.130	0.117	0.416
taurine	1.277	0.245	0.137	0.074	0.342
taurochenodeoxycholate	1.064	0.062	0.144	0.666	0.871
taurochenodeoxycholic acid 3-sulfate	1.068	0.066	0.198	0.740	0.910
taurocholate	1.085	0.082	0.164	0.616	0.844
taurocholenate sulfate*	1.105	0.100	0.134	0.458	0.748
taurodeoxycholate	0.820	-0.198	0.173	0.252	0.577
taurodeoxycholic acid 3-sulfate	0.683	-0.381	0.249	0.126	0.426
taurolithocholate 3-sulfate	0.992	-0.008	0.179	0.964	0.983
tauroursodeoxycholate	0.725	-0.321	0.224	0.151	0.468
tetradecadienedioate (C14:2-DC)*	0.827	-0.191	0.151	0.206	0.535
tetradecadienoate (14:2)*	1.031	0.030	0.132	0.820	0.936
tetradecanedioate (C14)	0.980	-0.021	0.154	0.893	0.970
theanine	1.109	0.103	0.222	0.642	0.861
theobromine	0.845	-0.168	0.173	0.331	0.659
theophylline	0.652	-0.427	0.185	0.021	0.178
thioproline	1.539	0.431	0.159	0.007	0.097
threonate	1.225	0.203	0.145	0.161	0.478
threonine	0.778	-0.251	0.169	0.137	0.442
thymol sulfate	0.995	-0.005	0.142	0.975	0.985
thyroxine	0.799	-0.225	0.161	0.164	0.480
tiglyl carnitine (C5)	0.813	-0.207	0.151	0.171	0.489
trans-urocanate	0.795	-0.229	0.169	0.174	0.495
tricosanoyl sphingomyelin (d18:1/23:0)*	1.404	0.340	0.202	0.093	0.380
tridecenedioate (C13:1-DC)*	0.835	-0.181	0.172	0.294	0.626

trigonelline (N'-methylnicotinate)	0.782	-0.246	0.144	0.086	0.367
trimethylamine N-oxide	0.766	-0.267	0.189	0.157	0.474
tryptophan	1.326	0.282	0.167	0.091	0.376
tryptophan betaine	1.193	0.177	0.151	0.241	0.565
tyramine O-sulfate	0.794	-0.231	0.134	0.084	0.361
tyrosine	1.266	0.236	0.127	0.063	0.314
umbelliferone sulfate	0.723	-0.324	0.249	0.193	0.523
undecanedioate (C11-DC)	0.960	-0.041	0.146	0.778	0.924
uracil	0.948	-0.054	0.142	0.706	0.888
urate	1.635	0.492	0.177	0.005	0.092
urea	0.980	-0.021	0.164	0.899	0.970
uridine	1.086	0.082	0.159	0.606	0.837
ursodeoxycholate	0.976	-0.024	0.145	0.868	0.966
valerate (5:0)	0.932	-0.070	0.147	0.633	0.855
valine	1.422	0.352	0.135	0.009	0.113
valylleucine	0.958	-0.043	0.189	0.822	0.936
vanillactate	1.086	0.082	0.180	0.647	0.865
vanillylmandelate (VMA)	1.039	0.038	0.130	0.767	0.918
X - 07765	1.174	0.160	0.173	0.354	0.677
X - 09789	1.053	0.052	0.129	0.688	0.882
X - 10458	1.027	0.027	0.144	0.853	0.957
X - 11261	1.059	0.057	0.184	0.756	0.912
X - 11308	1.216	0.195	0.204	0.337	0.664
X - 11315	1.455	0.375	0.159	0.018	0.166
X - 11372	1.013	0.013	0.168	0.939	0.975
X - 11378	1.194	0.177	0.190	0.351	0.677
X - 11407	0.988	-0.012	0.162	0.941	0.975
X - 11441	0.945	-0.057	0.138	0.681	0.880
X - 11442	0.963	-0.038	0.138	0.784	0.924
X - 11444	1.168	0.156	0.123	0.207	0.535
X - 11470	0.967	-0.034	0.149	0.821	0.936
X - 11478	0.839	-0.175	0.182	0.336	0.664
X - 11491	1.002	0.002	0.141	0.991	0.993

X - 11522	0.989	-0.011	0.146	0.940	0.975
X - 11530	1.012	0.012	0.150	0.937	0.975
X - 11632	1.100	0.095	0.140	0.499	0.767
X - 11787	1.190	0.174	0.134	0.193	0.523
X - 11795	1.270	0.239	0.155	0.123	0.420
X - 11843	0.712	-0.340	0.149	0.023	0.188
X - 11847	1.145	0.135	0.165	0.412	0.718
X - 11849	1.369	0.314	0.161	0.051	0.287
X - 11852	1.273	0.241	0.203	0.234	0.553
X - 11858	1.114	0.108	0.158	0.493	0.766
X - 11861	1.388	0.328	0.231	0.155	0.474
X - 11880	1.136	0.128	0.182	0.484	0.762
X - 12007	1.002	0.002	0.168	0.991	0.993
X - 12013	0.736	-0.307	0.222	0.167	0.482
X - 12015	1.064	0.062	0.142	0.662	0.871
X - 12026	0.876	-0.132	0.143	0.354	0.677
X - 12063	1.463	0.381	0.158	0.016	0.155
X - 12093 - retired for N2-acetyl,N6-methyllysine	0.679	-0.387	0.197	0.049	0.286
X - 12100	0.971	-0.030	0.159	0.853	0.957
X - 12101	1.061	0.059	0.149	0.692	0.884
X - 12104	0.862	-0.148	0.188	0.432	0.731
X - 12112	0.764	-0.269	0.149	0.071	0.338
X - 12117	1.029	0.029	0.229	0.900	0.970
X - 12127	1.190	0.174	0.137	0.203	0.532
X - 12170	0.960	-0.040	0.156	0.796	0.926
X - 12193	1.102	0.097	0.127	0.442	0.733
X - 12206	1.133	0.125	0.121	0.303	0.632
X - 12212	1.004	0.004	0.133	0.976	0.985
X - 12216	0.672	-0.398	0.129	0.002	0.052
X - 12221	0.565	-0.570	0.216	0.008	0.109
X - 12230	0.867	-0.143	0.154	0.355	0.677
X - 12261	3.165	1.152	0.879	0.190	0.522
X - 12283	0.672	-0.397	0.164	0.016	0.154

X - 12306	0.863	-0.147	0.180	0.414	0.718
X - 12329	0.663	-0.411	0.190	0.031	0.229
X - 12407	0.514	-0.665	0.252	0.008	0.109
X - 12410	0.777	-0.253	0.177	0.155	0.474
X - 12411	0.977	-0.023	0.123	0.852	0.957
X - 12456	1.378	0.320	0.186	0.084	0.361
X - 12462	1.141	0.132	0.126	0.298	0.628
X - 12472	0.841	-0.174	0.205	0.397	0.702
X - 12524	1.467	0.383	0.163	0.018	0.166
X - 12543	1.016	0.016	0.251	0.949	0.975
X - 12544	0.326	-1.120	0.542	0.039	0.256
X - 12680	1.205	0.186	0.142	0.190	0.522
X - 12689	1.184	0.169	0.178	0.342	0.670
X - 12718	0.828	-0.188	0.158	0.233	0.553
X - 12729	0.934	-0.069	0.157	0.663	0.871
X - 12730	0.802	-0.221	0.163	0.176	0.497
X - 12739	0.892	-0.114	0.186	0.538	0.795
X - 12740	0.962	-0.038	0.161	0.812	0.933
X - 12753	1.753	0.561	0.226	0.013	0.141
X - 12812	0.755	-0.281	0.178	0.115	0.412
X - 12822	0.926	-0.077	0.134	0.564	0.813
X - 12844	1.059	0.057	0.133	0.665	0.871
X - 12846	1.258	0.230	0.165	0.163	0.480
X - 12847	0.950	-0.051	0.164	0.753	0.912
X - 12849	0.785	-0.242	0.193	0.208	0.536
X - 12851	1.051	0.050	0.177	0.779	0.924
X - 12879	1.118	0.112	0.182	0.540	0.796
X - 12906	0.755	-0.281	0.169	0.097	0.384
X - 13431	1.245	0.219	0.158	0.165	0.482
X - 13507	0.730	-0.314	0.192	0.101	0.391
X - 13553	0.798	-0.226	0.187	0.227	0.551
X - 13658	1.230	0.207	0.180	0.250	0.577
X - 13684	1.057	0.056	0.147	0.705	0.888

X - 13695	1.162	0.150	0.166	0.368	0.678
X - 13737	1.042	0.041	0.157	0.793	0.926
X - 13835	0.683	-0.381	0.158	0.016	0.154
X - 13844	0.791	-0.235	0.236	0.320	0.650
X - 13866	1.190	0.174	0.182	0.337	0.664
X - 14056	1.336	0.290	0.147	0.049	0.286
X - 14662	1.457	0.376	0.178	0.034	0.242
X - 14939	0.993	-0.007	0.165	0.965	0.983
X - 15220	0.998	-0.002	0.197	0.993	0.994
X - 15245	1.294	0.258	0.175	0.140	0.447
X - 15461	0.984	-0.016	0.127	0.898	0.970
X - 15469	1.096	0.092	0.137	0.503	0.769
X - 15486	0.763	-0.271	0.194	0.162	0.478
X - 15492	1.448	0.370	0.192	0.054	0.289
X - 15503	0.845	-0.168	0.141	0.233	0.553
X - 15664	0.863	-0.148	0.163	0.364	0.678
X - 15666	1.274	0.242	0.172	0.158	0.474
X - 15728	1.071	0.068	0.170	0.687	0.882
X - 16087	1.252	0.225	0.231	0.331	0.659
X - 16124	0.900	-0.105	0.181	0.561	0.812
X - 16397	1.097	0.092	0.171	0.588	0.824
X - 16570	0.778	-0.251	0.154	0.104	0.394
X - 16576	2.054	0.720	0.254	0.005	0.085
X - 16580	1.094	0.090	0.160	0.576	0.821
X - 16649	0.988	-0.012	0.159	0.937	0.975
X - 16654	1.084	0.081	0.180	0.655	0.868
X - 16932	0.656	-0.422	0.214	0.048	0.285
X - 16935	1.185	0.170	0.186	0.362	0.678
X - 16938	1.010	0.010	0.155	0.946	0.975
X - 16944	1.156	0.145	0.178	0.416	0.718
X - 16946	0.938	-0.064	0.142	0.650	0.865
X - 16964	1.302	0.264	0.165	0.110	0.407
X - 17010	0.914	-0.089	0.159	0.575	0.820

X - 17137	0.896	-0.110	0.169	0.516	0.776
X - 17145	0.987	-0.013	0.168	0.939	0.975
X - 17162	0.680	-0.386	0.228	0.091	0.376
X - 17185	1.162	0.150	0.190	0.429	0.731
X - 17269	1.276	0.244	0.149	0.102	0.391
X - 17325	1.152	0.141	0.232	0.543	0.798
X - 17335	1.050	0.048	0.154	0.753	0.912
X - 17337	1.452	0.373	0.170	0.028	0.215
X - 17340	1.280	0.247	0.164	0.132	0.432
X - 17351	0.791	-0.235	0.148	0.112	0.409
X - 17357	1.010	0.010	0.149	0.948	0.975
X - 17359	1.492	0.400	0.171	0.019	0.167
X - 17365	0.837	-0.178	0.257	0.489	0.765
X - 17367	1.369	0.314	0.245	0.199	0.529
X - 17438	0.917	-0.086	0.160	0.588	0.824
X - 17612	1.049	0.048	0.152	0.753	0.912
X - 17653	1.046	0.045	0.185	0.808	0.932
X - 17654	0.984	-0.016	0.162	0.922	0.973
X - 17655	0.624	-0.471	0.228	0.039	0.256
X - 17673	0.532	-0.632	0.213	0.003	0.067
X - 17676	0.635	-0.454	0.170	0.008	0.106
X - 17685	0.709	-0.343	0.182	0.059	0.303
X - 18249	1.131	0.123	0.199	0.536	0.793
X - 18345	0.950	-0.051	0.167	0.758	0.912
X - 18886	1.209	0.190	0.166	0.253	0.577
X - 18888	0.832	-0.184	0.137	0.178	0.498
X - 18899	0.956	-0.045	0.168	0.789	0.926
X - 18901	0.947	-0.055	0.114	0.630	0.854
X - 18913	1.291	0.256	0.142	0.072	0.338
X - 18914	1.073	0.070	0.180	0.697	0.886
X - 18921	1.099	0.095	0.187	0.611	0.842
X - 18922	1.354	0.303	0.182	0.095	0.381
X - 19141	0.990	-0.010	0.141	0.946	0.975

X - 19183	1.674	0.515	0.310	0.096	0.382
X - 19438	1.193	0.177	0.172	0.303	0.632
X - 21258	1.287	0.252	0.163	0.121	0.420
X - 21295	0.775	-0.255	0.180	0.157	0.474
X - 21310	0.784	-0.243	0.114	0.033	0.241
X - 21315	0.665	-0.407	0.328	0.214	0.540
X - 21319	1.040	0.039	0.151	0.797	0.926
X - 21339	1.398	0.335	0.244	0.169	0.485
X - 21342	0.902	-0.103	0.150	0.494	0.766
X - 21353	1.014	0.014	0.136	0.916	0.973
X - 21364	1.437	0.363	0.203	0.074	0.342
X - 21410	1.726	0.546	0.179	0.002	0.057
X - 21411	1.326	0.282	0.147	0.055	0.289
X - 21441	1.352	0.302	0.164	0.065	0.321
X - 21448	0.937	-0.066	0.139	0.638	0.860
X - 21467	1.299	0.261	0.169	0.122	0.420
X - 21470	1.639	0.494	0.179	0.006	0.094
X - 21471	1.296	0.259	0.147	0.077	0.346
X - 21607	1.103	0.098	0.129	0.447	0.737
X - 21661	1.164	0.152	0.175	0.385	0.692
X - 21729	0.880	-0.127	0.141	0.365	0.678
X - 21733	0.519	-0.657	0.209	0.002	0.051
X - 21736	1.236	0.212	0.171	0.215	0.540
X - 21792	1.031	0.031	0.144	0.831	0.943
X - 21796	0.713	-0.338	0.180	0.060	0.303
X - 21803	0.531	-0.632	0.256	0.013	0.142
X - 21807	0.972	-0.029	0.241	0.906	0.970
X - 21821	0.722	-0.326	0.160	0.042	0.265
X - 21829	1.093	0.089	0.166	0.592	0.825
X - 21834	0.867	-0.143	0.160	0.372	0.678
X - 21959	1.144	0.135	0.157	0.392	0.697
X - 22143	1.169	0.156	0.170	0.357	0.678
X - 22162	0.758	-0.278	0.144	0.053	0.288

X - 22519	1.093	0.089	0.164	0.586	0.824
X - 22520	1.408	0.342	0.212	0.107	0.398
X - 22764	1.022	0.022	0.123	0.860	0.960
X - 22771	1.472	0.386	0.167	0.021	0.176
X - 22775	1.233	0.209	0.221	0.343	0.670
X - 22776	1.129	0.121	0.129	0.347	0.675
X - 22834	0.948	-0.053	0.221	0.811	0.933
X - 22918	0.782	-0.245	0.304	0.419	0.718
X - 23160	0.975	-0.025	0.202	0.901	0.970
X - 23196	1.156	0.145	0.144	0.314	0.647
X - 23276	1.077	0.074	0.156	0.635	0.856
X - 23294	1.004	0.004	0.232	0.985	0.990
X - 23295	1.072	0.070	0.163	0.668	0.871
X - 23296	0.774	-0.257	0.215	0.233	0.553
X - 23297	0.985	-0.015	0.139	0.915	0.973
X - 23314	1.122	0.115	0.129	0.371	0.678
X - 23369	1.175	0.161	0.136	0.236	0.554
X - 23498	0.955	-0.046	0.150	0.761	0.912
X - 23507	0.792	-0.233	0.214	0.276	0.603
X - 23583	0.760	-0.274	0.267	0.305	0.634
X - 23585	0.876	-0.132	0.111	0.234	0.553
X - 23587	1.131	0.123	0.182	0.498	0.766
X - 23590	0.985	-0.015	0.136	0.912	0.973
X - 23593	0.936	-0.066	0.126	0.599	0.830
X - 23636	1.200	0.182	0.135	0.177	0.498
X - 23639	0.985	-0.015	0.118	0.901	0.970
X - 23644	1.124	0.117	0.165	0.480	0.760
X - 23648	0.997	-0.003	0.164	0.983	0.990
X - 23662	0.677	-0.390	0.304	0.199	0.529
X - 23665	1.044	0.043	0.181	0.813	0.933
X - 23666	0.899	-0.107	0.138	0.440	0.733
X - 23680	1.237	0.213	0.165	0.199	0.529
X - 23739	0.919	-0.085	0.130	0.517	0.776

X - 23767	1.194	0.177	0.170	0.297	0.628
X - 23780	1.124	0.117	0.204	0.568	0.816
X - 23782	1.108	0.102	0.150	0.494	0.766
X - 23787	1.418	0.349	0.283	0.217	0.543
X - 23974	1.143	0.134	0.157	0.396	0.700
X - 24077	1.053	0.051	0.161	0.749	0.912
X - 24106	0.958	-0.043	0.134	0.750	0.912
X - 24243	1.123	0.116	0.163	0.476	0.760
X - 24293	1.000	0.000	0.161	0.998	0.998
X - 24295	1.212	0.193	0.140	0.168	0.482
X - 24306	1.084	0.080	0.172	0.641	0.861
X - 24307	1.076	0.073	0.171	0.667	0.871
X - 24328	0.847	-0.165	0.165	0.317	0.649
X - 24337	1.130	0.122	0.135	0.367	0.678
X - 24344	1.918	0.651	0.277	0.019	0.166
X - 24411	0.797	-0.227	0.216	0.294	0.626
X - 24418	0.709	-0.345	0.287	0.230	0.552
X - 24422	0.974	-0.026	0.157	0.868	0.966
X - 24431	1.174	0.160	0.134	0.232	0.553
X - 24432	0.769	-0.263	0.211	0.213	0.539
X - 24435	0.950	-0.051	0.141	0.715	0.892
X - 24455	1.017	0.017	0.135	0.899	0.970
X - 24456	1.280	0.247	0.274	0.368	0.678
X - 24473	0.949	-0.053	0.140	0.706	0.888
X - 24475	1.192	0.176	0.144	0.224	0.549
X - 24518	0.681	-0.385	0.178	0.031	0.229
X - 24527	1.040	0.040	0.180	0.826	0.939
X - 24540	1.100	0.095	0.188	0.614	0.842
X - 24542	0.924	-0.079	0.147	0.591	0.824
X - 24544	1.240	0.215	0.165	0.193	0.523
X - 24546	1.237	0.213	0.170	0.210	0.536
X - 24549	1.034	0.033	0.154	0.830	0.943
X - 24556	0.879	-0.129	0.158	0.414	0.718

X - 24565	0.876	-0.133	0.185	0.473	0.760
X - 24588	1.444	0.367	0.166	0.027	0.212
X - 24608	0.969	-0.031	0.123	0.798	0.926
X - 24637	1.080	0.077	0.157	0.623	0.850
X - 24686	1.379	0.321	0.169	0.056	0.296
X - 24699	0.806	-0.215	0.171	0.207	0.535
X - 24727	0.820	-0.198	0.191	0.300	0.629
X - 24728	1.213	0.193	0.172	0.260	0.586
X - 24736	1.167	0.155	0.227	0.495	0.766
X - 24748	1.045	0.044	0.167	0.794	0.926
X - 24765	0.924	-0.079	0.144	0.583	0.824
X - 24812	1.219	0.198	0.177	0.264	0.590
X - 24813	1.004	0.004	0.169	0.983	0.990
X - 24849	0.957	-0.044	0.144	0.759	0.912
X - 24947	1.454	0.374	0.205	0.068	0.325
X - 24949	1.234	0.210	0.142	0.137	0.442
X - 24951	1.763	0.567	0.181	0.002	0.051
X - 24952	1.178	0.164	0.141	0.246	0.574
X - 24972	1.358	0.306	0.181	0.090	0.376
X - 25172	1.061	0.059	0.145	0.686	0.882
X - 25220	1.438	0.363	0.224	0.105	0.397
X - 25265	0.795	-0.230	0.148	0.121	0.420
X - 25266	1.108	0.102	0.155	0.509	0.771
X - 25267	0.959	-0.042	0.137	0.757	0.912
X - 25271	0.870	-0.139	0.153	0.363	0.678
X - 25279	1.151	0.140	0.157	0.372	0.678
X - 25343	0.968	-0.032	0.157	0.837	0.946
X - 25371	1.080	0.077	0.142	0.591	0.824
X - 25388	1.147	0.137	0.192	0.476	0.760
X - 25419	0.754	-0.283	0.214	0.186	0.514
X - 25420	0.961	-0.040	0.169	0.813	0.933
X - 25422	1.172	0.159	0.177	0.371	0.678
X - 25463	1.106	0.100	0.136	0.462	0.751

xanthine	1.364	0.310	0.154	0.044	0.270
xanthurenate	1.163	0.151	0.154	0.327	0.659
ximenoylcarnitine (C26:1)*	1.748	0.559	0.247	0.024	0.194
xylose	1.316	0.274	0.162	0.090	0.376

Supplementary Table 6. Genetic associations of BMI with metabolic biomarkers

Biochemical	IPD				IVW				Cochran's Q	MR-Egger		Weighted median	
	β	SE	p-value	p-BH	β	SE	p-value	p-BH		β	SE	β	SE
1-(1-enyl-palmitoyl)-2-linoleoyl-GPC (P-16:0/18:2)*	-0.420	0.798	0.599	0.903	-0.048	0.052	0.056	0.182	0.556	-0.042	0.401	-0.030	0.141
1-(1-enyl-palmitoyl)-2-oleoyl-GPC (P-16:0/18:1)*	-0.429	0.835	0.608	0.906	-0.088	0.054	0.032	0.128	0.352	-0.082	0.404	-0.097	0.142
1-(1-enyl-palmitoyl)-2-palmitoleoyl-GPC (P-16:0/16:1)*	0.305	0.916	0.739	0.949	-0.040	0.059	0.839	0.927	0.556	-0.130	0.431	-0.041	0.151
1,5-anhydroglucitol (1,5-AG)	-1.150	0.877	0.193	0.652	0.050	0.057	0.437	0.648	0.040	-0.074	0.416	0.362	0.525
1-arachidonylglycerol (20:4)	-0.441	0.897	0.624	0.918	-0.014	0.058	0.300	0.517	0.480	-0.061	0.462	0.030	0.164
1-carboxyethylisoleucine	2.020	1.026	0.051	0.470	0.367	0.065	0.000	0.003	0.750	0.212	0.469	0.545	0.167
1-carboxyethylleucine	2.200	0.942	0.021	0.470	0.277	0.060	0.000	0.004	0.917	0.153	0.440	0.393	0.153
1-carboxyethylphenylalanine	2.090	0.828	0.013	0.470	0.256	0.055	0.001	0.017	0.917	0.123	0.399	0.369	0.140
1-carboxyethylvaline	1.760	0.826	0.035	0.470	0.317	0.054	0.000	0.000	0.657	0.074	0.400	0.521	0.141
1-linolenoylglycerol (18:3)	-0.816	0.792	0.305	0.739	-0.002	0.052	0.867	0.942	0.556	-0.016	0.394	0.042	0.137
1-linoleoylglycerol (18:2)	-0.369	0.811	0.650	0.930	0.045	0.053	0.713	0.858	0.412	0.056	0.407	0.074	0.143
1-linoleoyl-GPG (18:2)*	0.800	0.744	0.284	0.731	0.129	0.048	0.038	0.145	0.833	0.137	0.365	0.183	0.128
1-methylhistidine	0.560	0.853	0.513	0.860	0.182	0.056	0.043	0.151	0.836	0.053	0.391	0.319	0.137
1-methylurate	0.234	0.844	0.782	0.962	0.207	0.056	0.042	0.151	0.742	-0.015	0.402	0.514	0.602
1-myristoyl-2-arachidonoyl-GPC (14:0/20:4)*	-0.879	0.865	0.312	0.744	0.126	0.057	0.012	0.073	0.873	-0.078	0.425	0.337	0.149
1-myristoyl-2-palmitoyl-GPC (14:0/16:0)	0.316	0.840	0.707	0.946	0.211	0.055	0.000	0.005	0.833	-0.020	0.424	0.408	0.149
1-oleoyl-2-docosahexaenoyl-GPC (18:1/22:6)*	-1.000	0.880	0.257	0.705	-0.064	0.058	0.614	0.786	0.283	-0.134	0.410	-0.073	0.144
1-oleoylglycerol (18:1)	0.497	0.796	0.533	0.866	0.122	0.052	0.174	0.375	0.833	0.036	0.412	0.228	0.145
1-palmitoleoylglycerol (16:1)*	0.263	0.899	0.770	0.962	0.195	0.057	0.003	0.027	0.944	-0.041	0.442	0.391	0.155
1-palmitoyl-2-arachidonoyl-GPC (16:0/20:4n6)	-0.598	0.856	0.486	0.849	0.027	0.057	0.612	0.786	0.394	-0.166	0.427	0.107	0.149
1-palmitoyl-2-dihomo-linolenoyl-GPC (16:0/20:3n3 or 6)*	0.095	0.816	0.908	0.987	0.170	0.053	0.000	0.007	0.949	-0.050	0.391	0.278	0.137

1-palmitoyl-2-linoleoyl-GPC (16:0/18:2)	-0.775	0.862	0.370	0.778	0.108	0.056	0.074	0.220	0.833	0.106	0.416	0.196	0.146
1-palmitoyl-2-oleoyl-GPE (16:0/18:1)	1.560	0.827	0.062	0.487	0.256	0.055	0.000	0.007	0.610	0.038	0.405	0.413	0.142
1-palmitoyl-2-palmitoleoyl-GPC (16:0/16:1)*	0.121	0.767	0.874	0.986	0.261	0.051	0.000	0.000	0.348	-0.059	0.392	0.518	0.139
1-palmitoyl-2-stearoyl-GPE (16:0/18:0)*	1.140	0.991	0.253	0.705	0.272	0.064	0.084	0.240	0.836	0.065	0.460	0.447	0.163
1-stearoyl-2-arachidonoyl-GPC (18:0/20:4)	-0.524	0.882	0.554	0.877	0.014	0.059	0.957	0.983	0.556	-0.203	0.430	0.082	0.151
1-stearoyl-2-arachidonoyl-GPE (18:0/20:4)	0.528	0.849	0.535	0.866	0.206	0.056	0.026	0.115	0.937	-0.012	0.429	0.388	0.151
1-stearoyl-2-arachidonoyl-GPI (18:0/20:4)	-0.659	0.885	0.458	0.827	0.132	0.056	0.210	0.421	0.945	-0.021	0.437	0.258	0.154
1-stearoyl-2-docosahexaenoyl-GPE (18:0/22:6)*	0.091	0.800	0.909	0.987	0.126	0.053	0.379	0.594	0.750	0.116	0.391	0.206	0.137
1-stearoyl-2-linoleoyl-GPE (18:0/18:2)*	0.649	0.887	0.465	0.835	0.269	0.057	0.001	0.017	0.663	0.121	0.429	0.469	0.151
1-stearoyl-2-oleoyl-GPE (18:0/18:1)	1.450	0.854	0.092	0.527	0.232	0.055	0.005	0.038	0.951	0.070	0.412	0.373	0.144
1-stearoyl-2-oleoyl-GPS (18:0/18:1)	1.800	0.935	0.056	0.483	0.085	0.059	0.903	0.960	0.412	0.018	0.453	0.068	0.160
2,3-dihydroxy-5-methylthio-4-pentenoate (DMTPA)*	2.150	0.856	0.013	0.470	0.351	0.058	0.000	0.001	0.556	0.171	0.405	0.511	0.143
2-aminoadipate	-0.026	1.440	0.986	0.994	0.290	0.086	0.030	0.123	<0.001	0.000	0.010	0.003	0.738
2-aminoheptanoate	0.340	0.858	0.693	0.940	0.106	0.056	0.290	0.506	0.836	0.071	0.427	0.144	0.150
2-hydroxyarachidate*	0.115	0.873	0.895	0.986	0.042	0.056	0.477	0.683	0.674	0.064	0.421	0.082	0.148
2-hydroxybehenate	-0.162	0.767	0.833	0.973	0.099	0.048	0.168	0.370	0.982	0.104	0.419	0.230	0.148
2-hydroxyphenylacetate	1.910	0.966	0.051	0.470	0.244	0.064	0.079	0.230	0.873	0.064	0.460	0.341	0.159
2-linoleoylglycerol (18:2)	-0.273	0.877	0.756	0.959	0.030	0.056	0.976	0.987	0.663	0.047	0.434	0.065	0.151
2-methylbutyrylcarnitine (C5)	1.190	0.869	0.172	0.631	0.277	0.057	0.000	0.005	0.686	0.115	0.434	0.484	0.156
2-oxoarginine*	1.400	0.854	0.103	0.529	0.146	0.054	0.071	0.216	0.695	0.067	0.428	0.255	0.150
3beta-hydroxy-5-cholestenoate	-0.682	0.798	0.394	0.791	-0.079	0.052	0.207	0.419	0.512	-0.025	0.391	-0.030	0.138
3b-hydroxy-5-cholenoic acid	0.518	1.174	0.660	0.935	-0.071	0.073	0.794	0.905	0.676	0.213	0.523	-0.141	0.182
3-hydroxy-2-ethylpropionate	1.910	0.916	0.039	0.470	0.172	0.062	0.003	0.029	0.556	0.148	0.426	0.206	0.150
3-hydroxyisobutyrate	1.550	0.921	0.096	0.529	0.184	0.060	0.006	0.044	0.873	0.009	0.453	0.322	0.158

3-methyl-2-oxovalerate	1.250	0.884	0.161	0.619	0.224	0.059	0.001	0.014	0.917	0.080	0.440	0.312	0.155
4-cholesten-3-one	-0.989	0.801	0.219	0.685	0.176	0.051	0.008	0.053	0.979	-0.041	0.417	0.366	0.144
4-guanidinobutanoate	1.640	0.774	0.035	0.470	0.092	0.052	0.266	0.486	0.556	0.045	0.399	0.103	0.140
4-hydroxyglutamate	1.490	0.787	0.061	0.487	0.144	0.054	0.033	0.132	0.556	0.034	0.402	0.222	0.139
5-hydroxylysine	1.460	0.864	0.093	0.527	0.013	0.057	0.586	0.771	0.348	0.075	0.429	-0.024	0.151
5-methylthioadenosine (MTA)	1.580	0.800	0.050	0.470	0.264	0.052	0.023	0.106	0.436	0.168	0.434	0.484	0.153
6-oxopiperidine-2-carboxylate	0.530	0.962	0.582	0.895	0.124	0.061	0.388	0.604	0.836	0.119	0.448	0.210	0.156
alanine	0.546	0.860	0.527	0.865	0.128	0.059	0.043	0.151	0.945	-0.036	0.428	0.226	0.150
arabinose	1.380	1.090	0.207	0.666	-0.034	0.066	0.010	0.066	0.244	0.094	0.498	-0.811	0.638
argininate*	0.400	0.884	0.651	0.930	0.206	0.058	0.026	0.114	0.814	0.026	0.420	0.393	0.147
aspartate	2.070	0.788	0.009	0.470	0.062	0.052	0.347	0.559	0.417	0.146	0.398	0.009	0.140
behenoyl dihydrosphingomyelin (d18:0/22:0)*	-0.864	0.810	0.288	0.733	0.111	0.054	0.191	0.398	0.556	-0.076	0.409	0.280	0.144
beta-alanine	1.790	0.837	0.034	0.470	0.093	0.055	0.672	0.829	0.512	0.150	0.431	-0.096	0.429
beta-citrylglutamate	1.380	0.801	0.087	0.526	0.203	0.053	0.010	0.062	0.873	0.079	0.424	0.337	0.149
beta-hydroxyisovalerate	0.640	0.835	0.444	0.823	0.232	0.056	0.071	0.217	0.917	0.103	0.379	0.329	0.133
betaine	0.019	0.787	0.981	0.993	0.021	0.053	0.500	0.703	0.263	-0.065	0.405	0.077	0.142
bilirubin	-0.418	0.891	0.640	0.923	-0.123	0.059	0.205	0.417	0.873	0.005	0.422	-0.116	0.438
bilirubin (E,E)*	0.088	0.867	0.920	0.987	-0.168	0.057	0.189	0.395	0.917	0.095	0.435	-0.094	0.456
bilirubin (E,Z or Z,E)*	-0.297	0.887	0.738	0.949	-0.138	0.059	0.142	0.331	0.896	0.038	0.426	-0.167	0.445
biliverdin	-0.647	0.815	0.429	0.818	-0.156	0.053	0.040	0.147	0.975	0.032	0.407	-0.142	0.476
butyrylcarnitine (C4)	1.270	0.833	0.128	0.570	0.142	0.057	0.020	0.101	0.833	0.005	0.429	0.221	0.150
carotene diol (2)	-2.460	0.811	0.003	0.297	-0.128	0.056	0.088	0.244	0.676	-0.119	0.417	-0.366	0.481
ceramide (d16:1/24:1, d18:1/22:1)*	0.431	0.602	0.476	0.839	0.095	0.041	0.262	0.482	0.917	0.042	0.285	0.208	0.104
ceramide (d18:1/17:0, d17:1/18:0)*	-0.997	0.916	0.278	0.726	0.164	0.061	0.141	0.331	0.886	0.034	0.426	0.324	0.151

ceramide (d18:1/20:0, d16:1/22:0, d20:1/18:0)*	0.183	0.837	0.827	0.973	0.241	0.054	0.000	0.005	0.556	0.052	0.399	0.467	0.140
cortisol	-0.328	0.871	0.707	0.946	0.094	0.057	0.197	0.407	0.057	-0.154	0.428	0.228	0.150
cortisone	-0.444	0.958	0.644	0.925	0.059	0.060	0.166	0.366	0.080	-0.145	0.446	0.117	0.156
cystathionine	1.220	0.790	0.123	0.558	0.260	0.051	0.000	0.000	0.556	0.188	0.393	0.427	0.138
cysteine-glutathione disulfide	0.444	0.879	0.614	0.911	-0.228	0.055	0.022	0.106	0.525	-0.033	0.428	-0.472	0.150
diacylglycerol (14:0/18:1, 16:0/16:1) [2]*	0.049	1.192	0.967	0.993	0.273	0.072	0.033	0.130	0.556	-0.007	0.534	0.528	0.188
diacylglycerol (16:1/18:2 [2], 16:0/18:3 [1])*	-0.238	0.954	0.803	0.970	0.153	0.060	0.007	0.047	0.833	-0.062	0.478	0.312	0.168
dimethylglycine	0.782	0.857	0.363	0.777	0.111	0.058	0.030	0.124	0.514	0.175	0.407	0.041	0.143
ergothioneine	-0.387	0.809	0.633	0.922	-0.097	0.052	0.170	0.373	0.150	0.003	0.401	-0.253	0.397
erythritol	1.920	0.913	0.037	0.470	0.134	0.061	0.459	0.666	0.676	0.095	0.426	-0.049	0.456
formiminoglutamate	1.750	0.820	0.034	0.470	0.297	0.054	0.000	0.008	0.944	0.186	0.387	0.435	0.136
gamma-glutamyl-alpha-lysine	0.073	0.853	0.932	0.992	0.236	0.057	0.000	0.005	0.563	0.060	0.399	0.430	0.141
gamma-glutamylglutamate	1.320	0.803	0.102	0.529	0.141	0.053	0.011	0.070	0.556	0.068	0.371	0.173	0.130
gamma-glutamylglycine	-0.382	0.913	0.676	0.940	0.063	0.058	0.085	0.241	0.145	0.032	0.437	0.125	0.154
gamma-glutamylisoleucine*	1.840	0.837	0.029	0.470	0.373	0.056	0.000	0.000	0.873	0.199	0.390	0.504	0.137
gamma-glutamylleucine	0.876	0.848	0.304	0.739	0.360	0.056	0.000	0.000	0.750	0.167	0.392	0.563	0.138
gamma-glutamylphenylalanine	2.120	0.898	0.020	0.470	0.310	0.059	0.001	0.009	0.869	0.218	0.412	0.432	0.144
gamma-glutamyltyrosine	1.200	0.807	0.139	0.589	0.286	0.054	0.001	0.011	0.512	0.095	0.408	0.504	0.144
gamma-glutamylvaline	1.300	0.825	0.118	0.550	0.286	0.055	0.000	0.001	0.952	0.074	0.380	0.430	0.134
gamma-tocopherol/beta-tocopherol	-0.867	0.720	0.231	0.694	0.110	0.048	0.215	0.430	0.944	0.093	0.356	0.281	0.464
glucose	1.330	0.881	0.132	0.580	0.044	0.059	0.013	0.076	0.380	0.104	0.427	0.620	0.659
glucuronide of C10H18O2 (7)*	1.410	NA	NA	NA	0.029	0.046	0.560	0.753	<0.001	0.000	0.001	-0.091	1.260
glutamate	1.520	0.828	0.068	0.492	0.195	0.053	0.001	0.016	0.556	0.123	0.386	0.280	0.136
glycine	-0.542	0.875	0.536	0.866	-0.069	0.056	0.639	0.804	0.480	0.021	0.442	-0.099	0.155

glycosyl-N-(2-hydroxynervonoyl)-sphingosine (d18:1/24:1(2OH))*	0.579	0.613	0.347	0.777	0.099	0.039	0.718	0.860	0.836	0.040	0.319	0.157	0.112
glycosyl-N-nervonoyl-sphingosine (d18:1/24:1)*	0.086	0.548	0.875	0.986	0.002	0.036	0.081	0.235	0.348	-0.071	0.271	0.002	0.095
gulonate*	1.920	0.907	0.036	0.470	0.327	0.061	0.000	0.002	0.559	0.101	0.419	0.869	0.781
hexadecasphingosine (d16:1)*	0.462	0.878	0.600	0.903	0.122	0.054	0.047	0.161	0.814	0.061	0.409	0.235	0.140
homoarginine	1.300	0.886	0.144	0.589	0.097	0.058	0.548	0.745	0.556	-0.012	0.417	0.133	0.147
homocitrulline	1.290	0.953	0.179	0.640	0.152	0.064	0.018	0.094	0.776	0.114	0.438	0.185	0.154
hydantoin-5-propionate	1.400	1.017	0.172	0.631	0.215	0.064	0.250	0.470	0.917	0.179	0.445	0.300	0.157
hydroxyasparagine	1.060	0.851	0.215	0.679	0.153	0.057	0.065	0.205	0.917	0.056	0.397	0.201	0.140
isoleucine	1.690	0.930	0.071	0.495	0.273	0.062	0.001	0.009	0.873	0.197	0.445	0.359	0.156
isovalerate (C5)	1.170	0.842	0.167	0.628	0.282	0.055	0.045	0.156	0.763	0.044	0.428	0.506	0.151
isovalerylcarnitine (C5)	0.881	0.884	0.321	0.749	0.166	0.058	0.299	0.517	0.706	0.077	0.427	0.295	0.150
lactosyl-N-nervonoyl-sphingosine (d18:1/24:1)*	0.321	0.681	0.638	0.922	-0.021	0.043	0.127	0.314	0.480	-0.024	0.330	-0.037	0.116
lactosyl-N-palmitoyl-sphingosine (d18:1/16:0)	0.046	0.795	0.953	0.993	0.004	0.052	0.106	0.279	0.612	0.004	0.419	-0.069	0.148
leucine	1.110	0.904	0.223	0.685	0.249	0.061	0.001	0.011	0.873	0.148	0.430	0.373	0.151
linoleoyl-arachidonoyl-glycerol (18:2/20:4) [1]*	-0.271	0.823	0.742	0.949	0.088	0.053	0.289	0.506	0.436	-0.058	0.400	0.163	0.140
linoleoyl-arachidonoyl-glycerol (18:2/20:4) [2]*	-0.017	0.813	0.984	0.993	0.058	0.052	0.296	0.515	0.273	-0.003	0.405	0.127	0.142
linoleoyl-docosahexaenoyl-glycerol (18:2/22:6) [2]*	-0.168	0.740	0.821	0.972	-0.034	0.045	0.444	0.654	0.188	-0.033	0.375	-0.046	0.131
linoleoyl-linolenoyl-glycerol (18:2/18:3) [2]*	-0.348	0.841	0.679	0.940	-0.006	0.052	0.556	0.749	0.173	0.014	0.382	-0.044	0.131
linoleoyl-linoleoyl-glycerol (18:2/18:2) [1]*	-0.028	0.701	0.968	0.993	0.004	0.045	0.861	0.939	0.273	0.063	0.349	-0.056	0.122
lysine	-0.376	0.898	0.676	0.940	0.066	0.060	0.026	0.115	0.833	0.035	0.411	0.166	0.145
mannitol/sorbitol	1.690	0.866	0.053	0.483	0.162	0.058	0.021	0.102	0.917	-0.088	0.398	0.352	0.538
mannonate*	1.750	0.909	0.056	0.483	0.249	0.060	0.000	0.005	0.917	0.151	0.427	0.709	0.734
mannose	0.847	0.912	0.355	0.777	-0.042	0.061	0.537	0.737	0.057	0.038	0.428	-0.127	0.446
myristoyl dihydrosphingomyelin (d18:0/14:0)*	-0.894	0.835	0.286	0.732	0.166	0.056	0.004	0.032	0.873	-0.152	0.404	0.369	0.142

myristoyl-linoleoyl-glycerol (14:0/18:2) [1]*	0.285	0.934	0.761	0.961	0.180	0.061	0.037	0.142	0.978	0.067	0.471	0.322	0.168
N6,N6,N6-trimethyllysine	0.745	0.873	0.395	0.791	0.216	0.056	0.001	0.011	0.882	0.066	0.417	0.356	0.146
N-acetylalanine	0.295	0.888	0.741	0.949	0.161	0.058	0.179	0.382	0.949	0.102	0.430	0.268	0.151
N-acetylcarnosine	-0.264	0.719	0.714	0.946	-0.015	0.045	0.406	0.619	0.080	0.036	0.351	0.016	0.123
N-acetylglutamate	0.590	0.785	0.454	0.823	0.169	0.051	0.554	0.749	0.975	0.051	0.377	0.265	0.131
N-acetylglycine	-0.642	0.794	0.420	0.810	0.108	0.052	0.033	0.130	0.040	0.052	0.415	0.218	0.146
N-acetylisoleucine	1.670	0.923	0.073	0.495	0.271	0.058	0.000	0.005	0.917	0.154	0.423	0.403	0.149
N-acetylleucine	1.930	0.914	0.036	0.470	0.362	0.064	0.000	0.000	0.882	0.128	0.415	0.506	0.146
N-acetylphenylalanine	1.440	0.862	0.096	0.529	0.248	0.059	0.025	0.114	0.951	0.126	0.396	0.361	0.139
N-acetylputrescine	1.320	0.926	0.157	0.614	0.255	0.063	0.002	0.018	0.849	-0.028	0.438	0.393	0.154
N-acetyltryptophan	0.647	0.861	0.453	0.823	0.242	0.057	0.039	0.146	0.556	0.010	0.414	0.452	0.146
N-acetyltyrosine	1.620	0.929	0.083	0.523	0.310	0.063	0.000	0.006	0.563	0.095	0.441	0.521	0.157
N-acetylvaline	1.650	0.856	0.056	0.483	0.301	0.054	0.000	0.002	0.556	0.151	0.387	0.452	0.136
N-palmitoyl-sphinganine (d18:0/16:0)	0.607	0.877	0.490	0.850	0.170	0.056	0.172	0.374	0.674	0.050	0.418	0.253	0.147
N-palmitoyl-sphingosine (d18:1/16:0)	-0.735	0.878	0.404	0.795	0.150	0.057	0.015	0.082	0.945	0.076	0.411	0.278	0.145
N-stearoyl-sphinganine (d18:0/18:0)*	-0.812	0.894	0.366	0.777	0.150	0.060	0.064	0.202	0.480	-0.086	0.432	0.305	0.154
N-stearoyl-sphingosine (d18:1/18:0)*	-0.361	0.877	0.681	0.940	0.221	0.058	0.001	0.015	0.991	-0.020	0.400	0.400	0.141
oleoyl-arachidonoyl-glycerol (18:1/20:4) [1]*	-0.251	0.894	0.779	0.962	0.135	0.057	0.058	0.185	0.750	-0.127	0.427	0.290	0.150
oleoyl-arachidonoyl-glycerol (18:1/20:4) [2]*	0.167	0.908	0.854	0.979	0.174	0.057	0.029	0.120	0.833	-0.113	0.433	0.361	0.152
oleoyl-linolenoyl-glycerol (18:1/18:3) [2]*	0.270	0.940	0.775	0.962	0.012	0.063	0.665	0.824	0.436	0.037	0.496	0.012	0.172
oleoyl-linoleoyl-glycerol (18:1/18:2) [1]	0.020	0.934	0.983	0.993	0.114	0.059	0.196	0.407	0.556	0.057	0.449	0.166	0.158
oleoyl-linoleoyl-glycerol (18:1/18:2) [2]	0.233	0.948	0.807	0.970	0.118	0.059	0.101	0.270	0.556	0.067	0.447	0.180	0.157
oleoyl-oleoyl-glycerol (18:1/18:1) [1]*	0.566	0.793	0.477	0.839	0.143	0.051	0.013	0.076	0.836	-0.001	0.372	0.241	0.131
oleoyl-oleoyl-glycerol (18:1/18:1) [2]*	0.653	0.728	0.371	0.778	0.122	0.047	0.072	0.218	0.917	-0.015	0.347	0.226	0.122

palmitoleoyl-arachidonoyl-glycerol (16:1/20:4) [2]*	-0.193	0.898	0.830	0.973	-0.017	0.063	0.728	0.864	0.212	0.040	0.453	-0.075	0.168
palmitoleoyl-linoleoyl-glycerol (16:1/18:2) [1]*	-0.029	0.920	0.975	0.993	0.207	0.059	0.003	0.028	0.917	-0.026	0.456	0.373	0.160
palmitoyl-arachidonoyl-glycerol (16:0/20:4) [1]*	-0.221	1.077	0.838	0.974	0.192	0.071	0.114	0.292	0.975	-0.004	0.533	0.386	0.180
palmitoyl-arachidonoyl-glycerol (16:0/20:4) [2]*	0.219	0.926	0.814	0.970	0.155	0.058	0.107	0.280	0.676	-0.083	0.439	0.302	0.155
palmitoyl-linoleoyl-glycerol (16:0/18:2) [1]*	0.489	0.898	0.587	0.895	0.173	0.058	0.024	0.107	0.677	0.041	0.443	0.255	0.156
palmitoyl-linoleoyl-glycerol (16:0/18:2) [2]*	0.612	0.917	0.505	0.860	0.153	0.059	0.040	0.147	0.556	0.054	0.441	0.216	0.155
palmitoyl-oleoyl-glycerol (16:0/18:1) [1]*	1.600	0.868	0.068	0.492	0.273	0.057	0.000	0.007	0.917	0.081	0.428	0.450	0.150
palmitoyl-oleoyl-glycerol (16:0/18:1) [2]*	0.952	0.874	0.278	0.726	0.191	0.056	0.021	0.102	0.869	-0.004	0.412	0.307	0.145
pantothenate (Vitamin B5)	0.685	0.820	0.404	0.795	0.132	0.053	0.142	0.331	0.917	0.063	0.407	0.407	0.537
phenylalanine	2.630	0.853	0.002	0.297	0.218	0.058	0.020	0.102	0.917	0.240	0.428	0.275	0.151
proline	0.291	0.826	0.726	0.947	0.174	0.054	0.007	0.050	0.873	0.034	0.399	0.265	0.140
prolylglycine	-0.107	0.912	0.906	0.986	0.141	0.059	0.280	0.496	0.944	0.022	0.435	0.251	0.151
prolylhydroxyproline	0.733	0.819	0.373	0.778	0.205	0.054	0.000	0.005	0.674	0.013	0.413	0.378	0.145
propionylcarnitine (C3)	0.647	0.861	0.454	0.823	0.092	0.058	0.020	0.100	0.676	0.163	0.434	0.108	0.153
pyridoxate	1.220	0.985	0.217	0.679	-0.018	0.067	0.034	0.133	0.283	0.037	0.454	-0.724	0.632
retinol (Vitamin A)	-0.192	0.778	0.806	0.970	0.265	0.050	0.000	0.000	0.154	-0.043	0.394	1.186	1.046
S-adenosylhomocysteine (SAH)	0.599	1.209	0.621	0.915	0.270	0.074	0.088	0.244	0.080	0.000	0.005	0.002	0.322
serine	-0.189	0.868	0.828	0.973	-0.120	0.058	0.617	0.789	0.944	0.028	0.433	-0.208	0.152
sphingadienine	0.812	0.804	0.314	0.744	0.049	0.053	0.753	0.880	0.525	0.085	0.384	0.057	0.135
sphingomyelin (d17:2/16:0, d18:2/15:0)*	-1.260	0.737	0.091	0.527	0.123	0.046	0.060	0.191	0.873	-0.013	0.343	0.317	0.121
sphingomyelin (d18:0/18:0, d19:0/17:0)*	-1.160	0.840	0.170	0.629	0.135	0.056	0.082	0.238	0.556	-0.104	0.414	0.307	0.145
sphingomyelin (d18:0/20:0, d16:0/22:0)*	-1.220	0.866	0.160	0.619	0.139	0.057	0.013	0.076	0.556	-0.093	0.425	0.341	0.149
sphingomyelin (d18:1/14:0, d16:1/16:0)*	-0.882	0.820	0.284	0.731	0.187	0.053	0.000	0.008	0.663	-0.018	0.385	0.423	0.136
sphingomyelin (d18:1/18:1, d18:2/18:0)	-1.310	0.794	0.101	0.529	0.115	0.054	0.317	0.529	0.975	-0.080	0.397	0.312	0.140

sphingomyelin (d18:1/21:0, d17:1/22:0, d16:1/23:0)*	-1.070	0.627	0.090	0.527	0.075	0.040	0.066	0.205	0.884	-0.003	0.316	0.235	0.111
sphingomyelin (d18:2/14:0, d18:1/14:1)*	-1.270	0.802	0.117	0.550	0.237	0.051	0.001	0.015	0.480	-0.085	0.391	0.545	0.139
sphingomyelin (d18:2/21:0, d16:2/23:0)*	-0.788	0.685	0.252	0.705	0.057	0.044	0.495	0.700	0.963	0.075	0.363	0.170	0.127
stearoyl-arachidonoyl-glycerol (18:0/20:4) [1]*	-0.232	0.840	0.783	0.962	0.235	0.054	0.004	0.033	0.480	-0.090	0.423	0.496	0.149
stearoyl-arachidonoyl-glycerol (18:0/20:4) [2]*	-0.396	0.894	0.658	0.935	0.184	0.058	0.059	0.189	0.833	-0.043	0.432	0.346	0.151
tyrosine	1.060	0.919	0.252	0.705	0.196	0.062	0.014	0.076	0.991	0.099	0.435	0.324	0.153
urate	0.212	0.756	0.779	0.962	0.090	0.048	0.090	0.248	0.873	0.035	0.378	0.443	0.509
valine	1.270	0.952	0.183	0.645	0.245	0.063	0.000	0.007	0.873	0.123	0.448	0.383	0.157
X - 11442	-0.436	0.925	0.638	0.922	-0.106	0.060	0.153	0.347	0.836	-0.017	0.419	-0.151	0.147
X - 12063	0.088	0.827	0.915	0.987	0.036	0.054	0.161	0.359	0.556	0.009	0.436	0.124	0.151
X - 12170	1.410	0.903	0.120	0.555	0.301	0.061	0.042	0.151	0.512	0.109	0.433	0.548	0.634
X - 12411	1.830	0.898	0.043	0.470	0.156	0.060	0.124	0.310	0.836	0.179	0.426	0.221	0.150
X - 12456	1.390	1.098	0.210	0.672	0.304	0.071	0.283	0.497	0.676	0.135	0.554	0.548	0.188
X - 12524	0.646	0.857	0.452	0.823	0.284	0.055	0.000	0.003	0.436	0.045	0.430	0.565	0.152
X - 12680	1.990	0.882	0.026	0.470	0.277	0.058	0.121	0.303	0.556	0.115	0.443	0.445	0.156
X - 12689	0.036	0.823	0.965	0.993	0.074	0.054	0.975	0.987	0.556	0.030	0.379	0.039	0.409
X - 13737	1.330	0.833	0.111	0.548	0.169	0.057	0.360	0.574	0.917	0.103	0.397	0.209	0.139
X - 13844	3.760	1.959	0.065	0.492	0.227	0.110	0.001	0.014	0.917	0.153	0.857	1.447	1.418
X - 16946	-0.732	0.906	0.420	0.810	-0.093	0.058	0.308	0.521	0.663	0.035	0.423	-0.119	0.148
X - 17340	0.521	1.024	0.612	0.909	0.191	0.065	0.066	0.206	0.882	0.040	0.468	0.412	0.548
X - 18345	3.040	1.364	0.029	0.470	0.327	0.094	0.004	0.035	0.982	0.342	0.616	0.427	0.226
X - 18886	1.030	0.846	0.223	0.685	0.058	0.056	0.759	0.886	0.173	0.212	0.404	-0.059	0.436
X - 18922	0.687	0.760	0.368	0.777	0.078	0.048	0.924	0.965	0.873	0.137	0.407	0.170	0.143
X - 21829	0.812	0.857	0.345	0.777	0.143	0.054	0.117	0.297	0.769	0.106	0.414	0.227	0.147

X - 22775	2.060	0.905	0.025	0.470	0.229	0.065	0.005	0.038	0.991	0.122	0.443	0.674	0.715
X - 23294	0.264	1.303	0.840	0.975	-0.016	0.087	0.225	0.442	0.394	-0.115	0.665	0.649	0.867
X - 23590	1.010	0.921	0.276	0.726	0.186	0.061	0.053	0.176	0.663	0.135	0.417	0.370	0.546
X - 23593	1.390	0.909	0.128	0.570	0.207	0.061	0.023	0.106	0.917	0.050	0.439	0.541	0.593
X - 23680	-0.256	0.728	0.725	0.947	0.009	0.049	0.311	0.523	0.512	0.061	0.378	0.388	0.514
X - 24106	-0.284	0.812	0.727	0.947	0.010	0.053	0.871	0.946	0.352	-0.011	0.407	-0.014	0.440
X - 24328	1.660	0.730	0.025	0.470	0.039	0.048	0.274	0.492	0.273	0.103	0.346	-0.474	0.486
X - 24588	0.574	0.872	0.512	0.860	0.223	0.057	0.020	0.100	0.907	-0.047	0.401	0.581	0.615
X - 24728	1.840	0.792	0.022	0.470	0.246	0.054	0.005	0.041	0.850	0.127	0.388	0.632	0.626
X - 25371	0.700	0.909	0.443	0.823	0.149	0.062	0.830	0.921	0.880	0.086	0.435	-0.010	0.467

Supplementary Table 7. Multivariable analyses of the associations between metabolic biomarkers with risk of NAFLD

Biomarkers	Super-pathway	Sub-pathway	Univariable	Multivariable
			HR (95% CI)	HR (95% CI)
glutamate	Amino Acid	Glutamate Metabolism	0.85 (0.74, 0.99)	0.88 (0.71, 1.08)
cysteine-glutathione disulfide	Amino Acid	Glutathione Metabolism	1.27 (1.74, 1.46)	1.30 (1.09, 1.56)
isovalerylglycine	Amino Acid	Leucine, Isoleucine and Valine Metabolism	1.08 (1.74, 1.19)	1.15 (0.97, 1.37)
alpha-tocopherol	Cofactors and Vitamins	Tocopherol Metabolism	1.31 (1.74, 1.55)	1.09 (0.89, 1.35)
deoxycarnitine	Lipid	Carnitine Metabolism	1.43 (1.74, 1.71)	1.08 (0.85, 1.37)
3-hydroxysebacate	Lipid	Fatty Acid, Monohydroxy	0.74 (0.74, 0.88)	0.77 (0.60, 0.99)
phosphocholine	Lipid	Phospholipid Metabolism	0.78 (0.74, 0.93)	0.81 (0.65, 1.00)
3b-hydroxy-5-cholenoic acid	Lipid	Secondary Bile Acid Metabolism	0.72 (1.74, 0.83)	1.02 (0.81, 1.28)
glucuronide of C10H18O2 (7)*	Partially Characterized Molecules	Partially Characterized Molecules	0.81 (1.74, 0.95)	1.01 (0.84, 1.20)
1H-indole-7-acetic acid	Xenobiotics	Bacterial/Fungal	1.32 (1.74, 1.57)	1.08 (0.86, 1.36)
indolin-2-one	Xenobiotics	Food Component/Plant	0.74 (0.74, 0.86)	0.91 (0.69, 1.20)
saccharin	Xenobiotics	Food Component/Plant	1.46 (1.74, 1.72)	1.10 (0.85, 1.42)
X - 13729	Unknown		0.77 (0.74, 0.92)	0.94 (0.76, 1.16)
X - 17676	Unknown		0.88 (1.74, 0.99)	1.02 (0.84, 1.23)
X - 21785	Unknown		0.85 (0.74, 1.00)	0.86 (0.72, 1.03)
malate	Energy	TCA Cycle	0.92 (0.74, 1.07)	0.87 (0.72, 1.03)
behenoyl dihydrosphingomyelin (d18:0/22:0)*	Lipid	Dihydrosphingomyelins	1.55 (1.74, 1.82)	1.51 (1.15, 1.98)
1-myristoyl-2-palmitoyl-GPC (14:0/16:0)	Lipid	Phosphatidylcholine (PC)	1.28 (1.74, 1.50)	1.21 (0.98, 1.49)
phosphoethanolamine (PE)	Lipid	Phospholipid Metabolism	1.45 (1.74, 1.70)	1.09 (0.90, 1.33)
X - 12216	Lipid	Sphingomyelins	0.78 (1.74, 0.90)	1.22 (0.93, 1.60)
X - 21286	Unknown		0.77 (0.74, 0.90)	0.77 (0.55, 1.08)
carnitine	Lipid	Carnitine Metabolism	1.11 (1.74, 1.32)	1.03 (0.85, 1.24)
2-hydroxyoctanoate	Lipid	Fatty Acid, Monohydroxy	0.66 (0.74, 0.79)	0.86 (0.69, 1.08)
1-palmitoyl-2-oleoyl-GPI (16:0/18:1)*	Lipid	Phosphatidylinositol (PI)	1.27 (1.74, 1.50)	1.29 (1.00, 1.67)
1-(1-enyl-palmitoyl)-2-oleoyl-GPC (P-16:0/18:1)*	Lipid	Plasmalogen	1.47 (1.74, 1.77)	1.03 (0.78, 1.35)
6-hydroxyindole sulfate	Xenobiotics	Chemical	0.80 (1.74, 0.94)	1.19 (0.85, 1.65)
X - 18913	Unknown		1.26 (1.74, 1.51)	1.21 (0.93, 1.57)
X - 24951	Unknown		1.10 (0.74, 1.31)	0.87 (0.65, 1.15)

The univariable estimates were adjusted for age, age squared, sex, area, smoking, education, and fasting time.
The multivariable estimates were additionally adjusted for other metabolic biomarkers.

Supplementary Table 8. Previous studies on metabolic biomarkers with NAFLD

Ascertainment	Reference	Participants	Calsses/pathways	Traits
Liver biopsy	Gaggini/2018/Italy[1]	NAFLD (n=44), healthy controls (n=20)	Animo acids	↑: **alanine, glutamate, isoleucine, valine, tyrosine**, the GSG-index
			Lipids	↓: lysoPC C16:0, SM C16:0, PCae C32:1
	Lake/2015/US[1]	normal (n=19), steatosis (n=10), NASH (n=9), NASH not fatty liver (n=7)	BCAAs	↑: **leucine, isoleucine, valine**
			Carnitines	↑: **propionyl, hexanoyl, lauryl, acetyl, butyryl carnitine**
	Oresic/2013/Finland[1]	NAFLD (n=306), healthy controls (n=373)	Lipids	↑: triacylglycerols with low carbon number and double-bond content
				↓: lysoPC C16:0, C18:0, PC aa C34:2, C40:7, SM C36:0, 42:2, 34:1
	Tokushige/2013/Japan[1]	initial cohort: NAFLD (n=44); validation cohort: NAFLD (n=105), primary biliary cirrhosis (n=26), controls (n=48)	Sex hormone	↑: 16-OH-DHEA-S, 16-OH-DHEA-S/DHEA-S, 16-OH-DHEA-S/etiocholanolone-S
				↓: DHEA-S, etiocholanolone-S
	Kalhan/2011/US[1]	NAFLD/NASH (n=24), healthy controls (n=25)	Carnitines	↑: free carnitine, **butyrylcarnitine, methylbutyrylcarnitine**
			BCAAs & dipeptides	↑: **glutamyl dipeptides, branched-chain amino acids**
			Others	↓: **cysteine-glutathione**, long-chain fatty acids
	Soga/2011/Japan[1]	248 cases and controls including 11 NAFLD cases	A biomarker panel	↑: **gamma-glutamyl dipeptides**, transaminases, **methionine sulfoxide**
Ultrasound	Feldman/2018/Austria[1]	lean healthy (n=69), obese healthy (n=50), obese NAFLD (n=62)	Animo acids	↑: **alanine, leucine, isoleucine, valine**
				↓: **glycine**
	Pirola/2018/UK[2]	3048 participants of age 17 (70 NAFLD cases)	Lipids	↑: all XL- to S-VLDL, most cholesterols
			Inflammation	↑: glycoprotein acetyls
	Kaikkonen/2017/Finland[3]	5086 participants	Lipids	↑: VLDL, IDL, LDL, small HDL, VLDL size, ApoB, **glycerol**
				↓: large HDL, LDL size, HDL size
			Fatty acids	↑: total FA, MUFA%, SFA%
				↓: PUFA%, n-3%, DHA%, n-6%, linoleic acid%, n-6/n-3, PUFA/SFA
			Animo acids	↑: **isoleucine, leucine, valine, phenylalanine, tyrosin, alanine**
			Glycolysis	↑: **pyruvate**
			Others	↑: glycoprotein acetyls, CRP, GGT, ALT
MRI	Koch/2017/Germany[2]	555 participants (219 NAFLD cases)	Glutathione	↓: **cysteine-glutathione disulfide**
			Glutamate	↑: **glutamate**
			BCAAs	↑: **isoleucine, valine, leucine,** 3-methyl-2-oxovalerate
			Glutamyl dipeptides	↑: **γ-glutamylvaline, γ-glutamylisoleucine, γ-glutamylleucine**
			Others	↑: **propionylcarnitine, tyrosine, urate, cycol(leucylprolyl)**
	Boone/2019/Netherlands[2]	149 participants	Lipids	↑: lysoPC C14:0, PC aa C32:1, PC aa C36:1, PC aa C40:5

[1] Case-control, [2] cross-sectional, [3] prospective.

Supplementary Table 9. Previous prospective studies on metabolic risk factors with NAFLD

Study	Description	Ascertainment	HR
**BMI per 1 kg/m²**			
2016 Li	Meta-analysis of 21 studies	20 ultrasound, 1 biopsy	1.20 (1.14, 1.26)
2013 Musso	Included in 2016 Li et al.	Biopsy	1.32 (1.02, 2.13)
2019 Pang		Hospital record	1.23 (1.21, 1.25)
**Diabetes (yes vs no)**			
2018 Mantovani	Meta-analysis of 16 studies	14 ultrasound, 2 CT	2.22 (1.84, 2.60)
2018 Ma	Included in 2018 Mantovani et al.	CT	2.66 (1.20, 5.70)
2015 Shah	Included in 2018 Mantovani et al.	CT	2.06 (1.50, 2.80)
2019 Pang		Hospital record	1.76 (1.47, 2.16)
